# Comparative effectiveness and safety of interventions for acute diarrhea and gastroenteritis in children: A systematic review and network meta-analysis

**DOI:** 10.1371/journal.pone.0207701

**Published:** 2018-12-05

**Authors:** Ivan D. Florez, Areti-Angeliki Veroniki, Reem Al Khalifah, Juan J. Yepes-Nuñez, Javier M. Sierra, Robin W. M. Vernooij, Jorge Acosta-Reyes, Claudia M. Granados, Giordano Pérez-Gaxiola, Carlos Cuello-Garcia, Adriana M. Zea, Yuan Zhang, Naghmeh Foroutan, Gordon H. Guyatt, Lehana Thabane

**Affiliations:** 1 Department of Health Research Methods, Evidence & Impact; McMaster University, Hamilton, Canada; 2 Department of Pediatrics, Universidad de Antioquia, Medellín, Colombia; 3 Li Ka Shing Knowledge Institute, St. Michaels Hospital, Toronto, Canada; 4 Department of Primary Education, School of Education, University of Ioannina, Ioannina, Greece; 5 Department of Pediatrics, King Saud University, Riyadh, Saudi Arabia; 6 Iberoamerican Cochrane Centre, Biomedical Research Institute Sant Pau (IIB Sant Pau), Barcelona, Spain; 7 Department of Research, Netherlands Comprehensive Cancer Organisation (IKNL), Utrecht, Netherlands; 8 Department of Public Health, Universidad del Norte, Barranquilla, Colombia; 9 Department of Clinical Epidemiology & Biostatistics, Pontificia Universidad Javeriana, Bogotá, Colombia; 10 Hospital Pediátrico de Sinaloa, Culiacán, Mexico; 11 Department of Pediatrics, McMaster University, Hamilton, Canada; 12 School of Nutrition and Dietetics, University of Antioquia, Medellin, Colombia; 13 Programs for Assessment of Technology in Health (PATH), St. Joseph Health Care Hamilton, Hamilton, Canada; 14 Department of Medicine, McMaster University, Hamilton, Canada; 15 Department of Anaesthesia, McMaster University, Hamilton, Canada; TNO, NETHERLANDS

## Abstract

**Background:**

Many interventions have shown effectiveness in reducing the duration of acute diarrhea and gastroenteritis (ADG) in children. Yet, there is lack of comparative efficacy of interventions that seem to be better than placebo among which, the clinicians must choose. Our aim was to determine the comparative effectiveness and safety of the pharmacological and nutritional interventions for reducing the duration of ADG in children.

**Methods:**

Data sources included Medline, Embase, CENTRAL, CINAHL, LILACS, and Global-Health up to May 2017. Eligible trials compared zinc (ZN), vitamin A, micronutrients (MN), probiotics, prebiotics, symbiotics, racecadotril, smectite(SM), loperamide, diluted milk, lactose-free formula(LCF), or their combinations, to placebo or standard treatment (STND), or among them. Two reviewers independently performed screening, review, study selection and extraction. The primary outcome was diarrhea duration. Secondary outcomes were stool frequency at day 2, diarrhea at day 3, vomiting and side effects. We performed a random effects Bayesian network meta-analysis to combine the direct and indirect evidence for each outcome. Mean differences and odds ratio with their credible intervals(CrI) were calculated. Coherence and transitivity assumptions were assessed. Meta-regression, subgroups and sensitivity analyses were conducted to explore the impact of effect modifiers. Summary under the cumulative curve (SUCRA) values with their CrI were calculated. We assessed the evidence quality and classified the best interventions using the Grading of Recommendations, Assessment, Development & Evaluation (GRADE) approach for each paired comparison.

**Results:**

A total of 174 studies (32,430 children) proved eligible. Studies were conducted in 42 countries of which most were low-and middle-income countries (LMIC). Interventions were grouped in 27 categories. Most interventions were better than STND. Reduction of diarrhea varied from 12.5 to 51.1 hours. The combinations *Saccharomyces boulardii* (SB)+ZN, and SM+ZN were considered the best interventions (i.e., GRADE quality of evidence: moderate to high, substantial superiority to STND, reduction in duration of 35 to 40 hours, and large SUCRA values), while symbiotics (combination of probiotics+prebiotics), ZN, loperamide and combinations ZN+MN and ZN+LCF were considered inferior to the best and better than STND [Quality: moderate to high, superior to STND, and reduction of 17 to 25 hours]. In subgroups analyses, effect of ZN was higher in LMIC and was not present in high-income countries (HIC). Vitamin A, MN, prebiotics, kaolin-pectin, and diluted milk were similar to STND [Quality: moderate to high]. The remainder of the interventions had low to very-low evidence quality. Loperamide was the only intervention with more side effects than STND [Quality: moderate].

**Discussion/Conclusion:**

Most interventions analyzed (except vitamin A, micronutrients, prebiotics, and kaolin-pectin) showed evidence of superiority to placebo in reducing the diarrhea. With moderate-to high-quality of evidence, SB+ZN and SM+ZN, demonstrated the best combination of evidence quality and magnitude of effect while symbiotics, loperamide and zinc proved being the best single interventions, and loperamide was the most unsafe. Nonetheless, the effect of zinc, SB+ZN and SM+ZN might only be applied to children in LMIC. Results suggest no further role for studies comparing interventions against no treatment or placebo, or studies testing loperamide, MN, kaolin-pectin, vitamin A, prebiotics and diluted milk.

**PROSPERO registration:**

CRD42015023778.

## Introduction

Diarrheal diseases remain the third cause of death among children younger than 5 years old[1,2]. Most of these deaths occur in low- and middle-income countries (LMIC). Although in high-income countries (HIC) diarrhea is rarely fatal, it is a leading cause of hospitalizations [3]. Acute diarrhea is defined by the World Health Organization (WHO) as the passage of three or more loose or liquid stools per day, for three or more days, and for less than 14 days[4]. The American Academy of Pediatrics, defines acute gastroenteritis as diarrheal disease of rapid onset, with or without additional symptoms and signs, such as nausea, vomiting, fever, or abdominal pain[5]. Although acute diarrhea and gastroenteritis (ADG) are based on two different definitions, they are usually related to the same disease: a gastrointestinal infection caused by microorganisms such as rotavirus (RV), norovirus, *Salmonella*, *E*. *coli*, *Campylobacter*, among others[6].

According to the WHO, the main goal of treatment in ADG is to prevent dehydration, and the strategies to achieve the goal include the use of oral rehydration solution (ORS), continuing oral feeding and zinc supplementation^4^. Therefore, in addition to the ORS, the only intervention recommended by the WHO for ADG is ZN, which is commonly used in many LMIC[7]. Conversely, some guidelines, mostly from HIC, have either recommended additional interventions such as probiotics, racecadotril (an enkaphalinase inhibitor) and smectite (a natural clay formed from sheets of aluminium and magnesium silicate) and/or have discouraged the use of zinc[8,9]. Investigators have conducted many comparative trials of interventions for ADG, as well as several systematic reviews and pairwise meta-analysis with comparisons to placebo or no treatment[10–16]. These reviews have showed that interventions such as smectite, racecadotril, zinc, probiotics, yogurt, and lactose-free formula are effective for reducing the diarrhea duration, in comparison to placebo. However, to date, the summaries have not established the best interventions, or those that clinicians should avoid. To provide such information, clinicians require an analysis that simultaneously addresses all the available treatment options.

One review performed direct and indirect comparisons to address this issue[17]. This review did not, however, include all the available interventions; provided limited information regarding the search strategies, included and excluded studies, and the risk of bias assessment; did not assess for potential effect modifiers; and did not address the overall quality of evidence. We have therefore, through a network meta-analysis (NMA), addressed the effectiveness and safety of the available interventions for the treatment of the ADG in children.

## Materials and methods

This systematic review and NMA protocol was registered with PROSPERO (CRD42015023778), and published in an open access journal[18]. Since methods were reported previously, they are described only briefly here. In the supporting information (File A in S1 Appendix) we describe differences between the published protocol and the final protocol. This report complies with the recommendations of the Preferred Reporting Items for Systematic Reviews and Meta-Analyses (PRISMA) Extension statement for reporting of systematic reviews incorporating NMA (File B in S1 Appendix)[19].

### Search strategy and eligibility criteria

MEDLINE, Embase, Global Heath, CINAHL, LILACS, and Cochrane Central Register of Controlled Trials (CENTRAL) were searched from inception to May 1st, 2017. We screened the grey literature through trial registries and dissertation databases. We considered RCTs and quasi-RCTs assessing interventions for reducing diarrhea in children. Our previously published protocol^18^ and the supporting information (File C in S1 Appendix) provide additional information regarding the search strategy.

Interventions of interest were diluted milk, lactose-free formula, loperamide, micronutrients, prebiotics, probiotics, racecadotril, smectite, symbiotics (defined as the combination of any probiotic strain with any prebiotic), vitamin A, yogurt, and zinc. We did not consider antiemetics, antimicrobials, alternative or non-mainstream interventions (such as herbal medicines), neither we considered trials of interventions for rehydration. However, we did not exclude studies if antibiotics or antiemetics were used as co-interventions. We created clusters of interventions that analyzed into the same group based on their characteristics (see Results section). We created a group called standard treatment (STND), which included the interventions that did not provide an active treatment: intervention arms labeled as placebo, no treatment, “only oral rehydration solution (ORS)” (irrespective of the osmolarity), standard, or regular treatment. In trials comparing lactose free-formula to regular formula, we also classified the latter as STND.

Zinc is widely used in many LMIC, and therefore, following WHO recommendations, the standard treatment of ADG cases in these countries is ORS plus zinc supplementation^4^. In studies in which zinc was part of the standard treatment, the arm receiving only zinc was labeled as zinc, not STND; the other arm was labelled as zinc plus an added intervention. For instance, if a study evaluated standard treatment plus smectite vs. standard treatment (which included zinc + ORS), the comparison was considered as smectite plus zinc vs zinc. We defined combinations of interventions as the simultaneous use of two or more interventions, except for probiotics (commonly, they include preparations of different strains) and symbiotics (combination of probiotics and prebiotics), which were considered single interventions. Our primary outcome was mean diarrhea duration (hours) from the time of recruitment. Secondary outcomes were stool frequency at day 2, diarrhea at day 3, vomiting, and side effects.

### Study selection, data extraction and descriptive analysis

Reviewers, in pairs, independently screened abstracts and full-text articles, extracted the data in a pre-specified extraction form, and assessed the risk of bias with a modified version of the Cochrane tool[20,21]. We extracted patients’ baseline information (country, clinical status, hydration, nutritional status, etiology, days with diarrhea), and studies characteristics (risk of bias, funding source, interventions and their characteristics, number of patients, and outcomes results). Reviewers resolved disagreements by discussion or, if necessary, by a third reviewer (IDF,GHG). We summarized the main characteristics of the studies using descriptive statistics. Categorical variables were summarized using frequencies and proportions, and continuous variables using the means, Standard deviation and ranges. We calculated grand means, by calculating the mean of the means of continuous variables from each study.

### Pairwise and network meta-analysis

We performed pairwise meta-analysis of the available direct comparisons using a Bayesian random-effects model to obtain direct evidence. Treatment effects were estimated using odds ratio (OR) for dichotomous outcomes and mean difference (MD) for continuous outcomes: in hours for diarrhea duration and in number of stools for stool frequency, with their corresponding 95% credible intervals (CrI). Credible intervals provide a range of values for the parameter of interest (e.g., OR) that are plausible given the observed data. We used vague priors, i.e. distributions that play a minimal role in the posterior distribution[22], for all model parameters and a common half-normal prior distribution for the between-study standard deviation (τ~Ν(0,1), τ>0) across all treatment comparisons per outcome[23]. We used a common between-study standard deviation across all treatment comparisons in each network to increase accuracy in τ estimation, especially when a single study was available in a treatment comparison where τ was not estimable. In a Bayesian framework analysis, a prior distribution, or “prior”, of a model parameter refers to the probability distribution that expresses a prior belief about this parameter before new evidence is taken into account. A prior can be vague or “non-informative” in which case it will have little or no impact on the inferences or, alternatively, informed. We used non-informative priors in our analyses[23].

Heterogeneity in pairwise meta-analysis for all the direct comparisons (i.e., head-to-head trial evidence) was assessed with the I^2^ statistic, measuring the percentage of variability that cannot be attributed to sampling error expressed as a percentage (%)[24]. I^2^ values between 0–40% may represent not important heterogeneity; 30–60% may represent moderate heterogeneity; 50–90%: may represent substantial heterogeneity; 75–100% may represent considerable heterogeneity[25]. All analyses were performed using the Markov Chain Monte Carlo method. For each outcome and a connected network of studies, we performed a Bayesian random-effects NMA. In contrast to regular pairwise meta-analysis, which only allows comparisons of two interventions that have been directly compared in head-to-head studies, NMA is a special technique that allows simultaneous comparison between several treatments even when direct comparisons (i.e., head-to-head studies) are unavailable. NMA can produce summary of relative effectiveness among all treatments being compared in the network.

In the NMA, in the absence of direct evidence for given comparisons, the indirect comparisons (i.e., comparisons of interventions using data across different studies through a common comparator intervention) provided the estimates. In the presence of both direct and indirect evidence, the NMA provided a NMA estimate[26]. Heterogeneity, for the whole network, which considers the extent of inconsistency of results within each of the direct comparisons, was measured with the Tau^2^ statistic. Transitivity is the assumption that an indirect comparison is a valid method to compare two treatments, because the studies are sufficiently similar in important clinical and methodological characteristics; in other words, that they are similar in their distributions of effect modifiers[27,28]. Coherence (also called consistency in NMA) is the assumption that the direct effect estimates and the calculated indirect estimates for a given comparison are similar[28,29]. We explored potential causes of important heterogeneity, transitivity and incoherence via meta-regression assuming common fixed coefficient across comparisons for interventions vs. placebo/standard treatment (STND), sensitivity analyses and subgroup analysis.

Variables for transitivity assumption assessment are described in the supporting information (File A in S1 Appendix). The presence of incoherence between the direct and indirect estimates was assessed with both the global test random-effects design-by-treatment interaction model[30] and the local test, the node-splitting method[31]. Meta-regression was performed for the independent variables age, year of publication, etiology (% of children with rotavirus), and days with diarrhea before recruiting. Sensitivity analyses were conducted excluding quasi-RCTs, studies that had high risk of bias because of allocation concealment and because outcome assessment blinding. We performed subgroup analyses by country income classification based on the World Bank classification at 2016 (HIC vs LMIC)[32] and by clinical setting (inpatients and outpatients).

Similar to the pairwise meta-analysis model, we fitted a Bayesian hierarchical NMA model with vague priors adjusting for correlation of multi-arm trials and assuming a common-within network heterogeneity variance (τ~Ν(0,1),τ>0). After discarding the first 10,000 iterations, 100,000 simulations were drawn with thinning of 10, i.e., storing every 10^th^ iteration. The model convergence was checked by visual inspection of the evaluation of the mixing of two chains. The analysis was performed in OpenBUGs (version 3.2.3)[33] When 10 or more studies were available for an outcome, we addressed small-study effects and publication bias using the comparison-adjusted funnel plots[34]. Funnel plots were used to inform the quality of evidence assessment with GRADE. We calculated the Surface Under the Cumulative Ranking (SUCRA) curve values and to capture uncertainty in the parameter values informed treatment rankings, the corresponding CrI[35,36]. The closer to the 100% a SUCRA value for an intervention is the higher the rank in the intervention’s effectiveness (or safety) compared to the interventions assessed in the NMA. We graphically depicted SUCRA values for all outcomes in a rank-heat plot (http://rh.ktss.ca/)[37].

### Rating the quality of the evidence in the estimates

We assessed the quality of evidence according to the GRADE criteria for direct estimates[38]. For indirect and NMA estimates, the approach by Puhan et al.[39] complemented with the approach by Brignardello-Petersen et al.[40] was used. Direct estimates were evaluated with the traditional five GRADE criteria (risk of bias, imprecision, indirectness, inconsistency and publication bias)^38^. Additionally, in second step for each comparison, we removed the imprecision criterion to create direct GRADE quality assessments based only on four criteria (not rating down if imprecision was present, because this criterion was applied to the NMA estimate assessment, as is detailed below) to be used to inform the NMA estimates quality assessments[40]. The indirect estimates assessment was based on the direct estimates quality that informed the indirect in first order loops, with the lowest variance (specifically, the lowest quality of both direct comparisons, will be the quality of the indirect estimate), and rated down the quality if intransitivity was judged as serious[27,29]. The NMA quality estimates were based on the direct and indirect estimates quality (specifically, the higher of the qualities between direct and indirect, was chosen as the quality of the NMA estimate), and rating down if incoherence[39] or imprecision[40] were present. The protocol publication provides additional information on the quality assessment process[18].

### Summary of more and less preferred treatments

For the primary outcome, we developed a system to summarize the results, establishing different groups of interventions (from the best to the worst interventions groups) based on the effect estimates obtained from the NMA, their associated evidence certainty, and their SUCRA (ranking) values. First, we separated moderate-to-high quality (which we define as: high certainty) and low-to-very-low (which we defined as: low certainty) bodies of evidence (based on the GRADE). Then, within each group, we separated treatments based on the magnitude of effect estimates (i.e., based on the magnitude of the effect on reduction in diarrhea duration), as follows: 1)Amongst the best interventions: based on the effect estimates these interventions were all better than STND (they had the largest reduction in diarrhea duration), and also superior to interventions that at the same time, were better than STND (i.e., credible interval of estimates between interventions from group 2 excluded the null value); 2)Inferior to the best but better than the worst interventions: based on the effect estimates these interventions were superior to STND (credible interval of estimates between the intervention and STND excluded the null value), but inferior to interventions from group (1); and 3)Amongst the worst interventions: these interventions showed no differences in effect estimates when compared to STND).

## Results

### Selection, characteristics and risk of bias of studies

**Fig 1:**
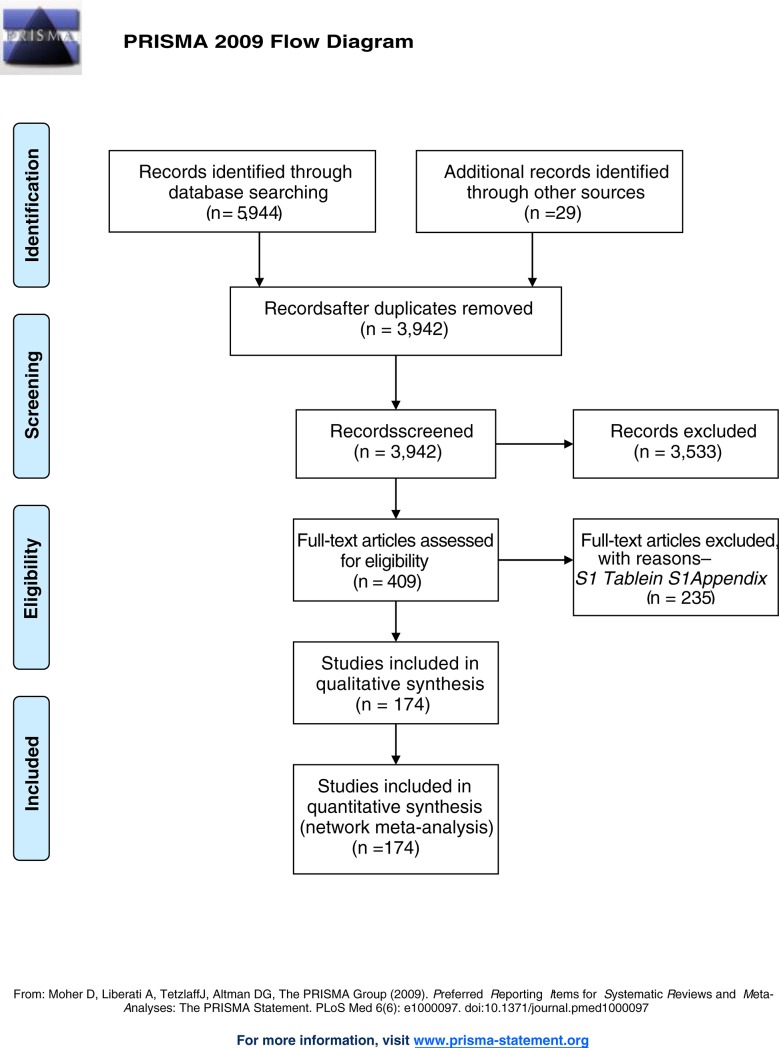
Article selection flowchart.

**Fig 2:**
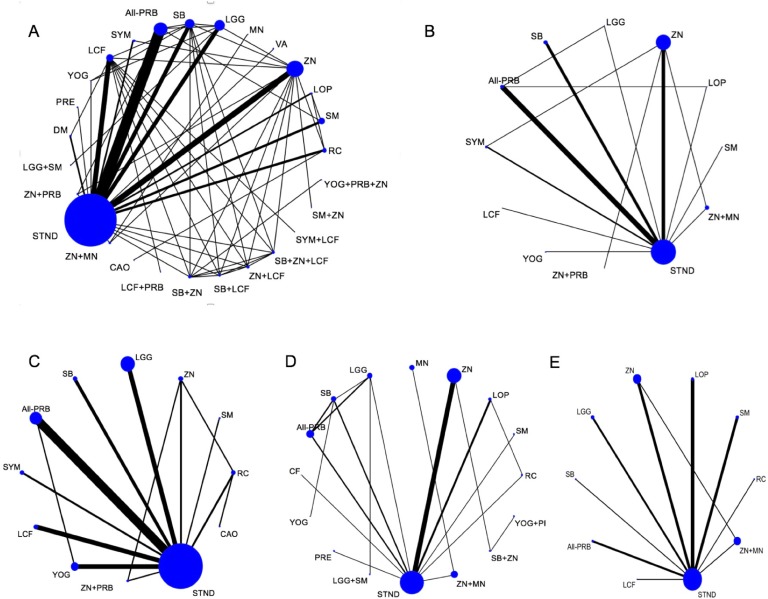
Network meta-analysis plots. (A) Diarrhea duration, (B) Stool Frequency at day 2, (C) Diarrhea at day 3, (D) Vomiting and (E) Side effects. Nodes are proportional to the number of patients included in the corresponding treatments, and edges are weighted according to the number of studies included in the respective comparisons. Interventions' abbreviations: ALL-PRB: All probiotics, except LGG and SB; CAO: Kaolin-Pectin; DM: Diluted Milk; LCF: lactose-free formula; LCF+PRB: Lactose Free Formula + Probiotics; LGG: *L*. *rhamnosus*-GG; LGG+SM: LGG + Smectite; LOP: Loperamide; MN: Micronutrients; PRE: prebiotics; RC: Racecadotril; SB: *Saccharomyces boulardii*; SB+LCF: *S*. *boulardii* + Lactose-Free Formula; SB+ZN: *S*. *boulardii* + Zinc; SB+ZN+LCF: *S*. *boulardii* + Zinc + Lactose free Formula; SM: Smectite; SM+ZN: Smectite + Zinc; STND: Standard treatment or Placebo; SYM: Symbiotics; SYM+LCF: Symbiotics + Lactose-Free Formula; VA: Vitamin A; YOG: Yogurt; YOG+PRB+ZN: Yogurt + Probiotics + Zinc; ZN: Zinc; ZN+LCF: Zinc + Lactose Free Formula; ZN+MN: Zinc + micronutrients; ZN+PRB: Zinc + Probiotics.

Of 5,944 titles identified, 409 proved potentially eligible (Fig 1). Of these, we excluded 235 studies (Table A in S1 Appendix) and included 174 studies in 32,430 children (Table B in S1 Appendix). Studies were conducted in 42 countries: 119 (68.4%) in LMIC, 51 (29.3%) in HIC, and 4(2.3%) in both. The grand mean of days with diarrhea, or the mean days with diarrhea before recruiting was 2.19 days (SD = 1.01, range 0.35–6.58) and the age of children across the studies was 16.9 months (SD = 10.88, range: 0.6–85.7). Studies were conducted in inpatients (100; 57.5%), and outpatients (34; 19.5%); the setting was mixed in 22 studies (12.6%) and was not clear in 20 (11.5%). In 108 studies (62.1%) the etiology of the ADG was clearly stated. Among them, the grand mean of proportion of rotavirus detection was 67.6% (range 0–100%). The pharmaceutical industry funded 29 studies (16.7%); non-for-profit organizations funded 45 (25.8%); investigators obtained funds from both in 16 (9.2%); in 80 (46%), authors did not report funding source, and in 4 (2.3%) authors stated the study had no funding. In the supporting information (Table C in S1 Appendix), we present the studies’ risk of bias ratings. The number of studies that had high or probably high Risk of bias in sequence generation, allocation concealment and outcome blinding were, respectively: 60 (34.5%), 94(54%) and 69(39.6%). The descriptive analysis of variables considered for transitivity assessment are presented in the Table D in S1 Appendix. Trials studied 51 treatments that were grouped in 27 categories (Table 1): 16 single interventions, including STND, and 11 combinations of interventions. Fig 2 shows the network geometry plots with the available direct comparisons for the five outcomes.

**Table 1:** Classification of interventions.

Abbreviations	Name	Additional Information
**All-PRB**	All Probiotics (All probiotics different from LGG and SB)	Different preparations of *Lactobacillus reuteri* (including DSM17938), *Lactobacillus acidophilus*, *Escherichia coli-*Nissle1917, *Lactobacillus paracasei*, *Lactobacillus rhamnosus*-Rosell, *Lactobacillus sporogenes*, *Bifidobacterium longum*, *Enterococcus faecalis*, *Clostridium butyricum*, *Bifidobacterium lactis*, *Lactobacillus helveticus*, *Bifidobacterium bifidum*, *Enterococcus faecalis*, *Lactobacillus bifidus*, *Streptococcus*. *thermophilus*, and *Bacillus mesentericus*, or combinations of them
**CAO**	Kaolin-pectin	Kaolin-pectin
**DM**	Diluted Milk	Diluted formula or milk at 50% or at 25%
**LCF**	Lactose-Free Formula	Lactose-Free formula. Also includes: Soy and Lactose free formula
**LCF+PRB**	Lactose-Free Formula + Probiotics	Combination of Lactose Free Formula **plus** probiotics. One probiotic mixture studied: *Bifidobacterium lactis* **plus** *S*. *thermophilus*
**LGG**	LGG	*Lactobacillus rhamnosus GG*
**LGG+SM**	*Lactobacillus* LGG + Smectite	Combinations of *Lactobacillus rhamnossus-*GG + smectite
**LOP**	Loperamide	Loperamide
**MN**	Micronutrients	Preparations including different doses of vitamin A, zinc, Vitamin D, Vitamin B1, riboflavin, Vitamin B6, nicotinamide, calcium. All micronutrients supplemented 1 RDA (Recommended dietary Allowance)
**PRE**	Prebiotics	Includes: Preparations with soy polysaccharide, alfa-cellulose, gum arabic, fructo-oligosaccharides, and inulin
**RC**	Racecadotril	Racecadotril (acetorphan)
**SB**	*S*. *boulardii*	*Saccharomyces boulardii*
**SB+LCF**	*S*. *boulardii* + Lactose-Free Formula	Combinations of *Saccharomyces boulardii* **plus** lactose-free formula
**SB+ZN**	*S*. *boulardii* + Zinc	Combinations of *Saccharomyces boulardii* **plus** zinc
**SB+ZN+LCF**	*S*. *Boulardii* + Zinc + Lactose free Formula	Combinations of *Saccharomyces boulardii* **plus** zinc **plus** lactose-free formula
**SM**	Smectite	Smectite (or diosmectite)
**SM+ZN**	Smectite+ Zinc	Combinations of s mectite **plus** Zinc
**STND**	Placebo or Standard (no treatment)	Includes:—Standard treatment (no intervention in addition to hydration) - Placebo - Regular Milk of formula (in comparisons vs. lactose-free/soy formula or vs yogurt)
**SYM**	Symbiotics	Includes the following 4 preparations of Probiotics **plus** prebiotics: - *Lactobacillus acidophilus*, *Lactobacillus rhamnosus*, *Bifidobcterium bifidum*, *Bifidobacterium longum*, *Enterococcus faecium* **plus** fructo-oligosaccharides - *Lactobacillus paracasei* B21060 **plus** arabinogalactan and xilooligosaccharides, - *Streptococcus thermophilus*, *Lactobacillus rhamnosus*, *Lactobacillus acidophilus*, *Bifidobacterium infantis*, *Bifidobacterium lactis* **plus** fructo-oligosacharides - *Lactobacillus casei*, *Lactobacillus rhamnosus*, *Lactobacillus plantarum*, *Bifidobacterium lactis* **plus** fructo- and galacto-oligosaccharides and polydextrose and thiamine *- Bifdobacterium*. *lactis* B94 **plus** inulin
**SYM+LCF**	Symbiotics + Lactose-Free Formula	Combinations of symbiotics **plus** lactose-free formula
**VA**	Vitamin A	Vitamin A
**YOG**	Yogurt	Yogurt or fermented milk preparations with one of the following: *- Lactobacillus*. *acidophillus* **plus** *Bifidobacterium*. *lactis* *- Lactobacillus casei (*DN-1140001) *- Lactobacillus rhamnosus* GG (2) *- Lactobacillus bulgaris* **plus** *Streptococcus thermophilus* *- Lactobacillus acidophilus* *- Lactobacillus rhamnosus* **plus** *Lactobacillus bulgaricus*
**YOG+PRB+ZN**	Yogurt + Probiotics + Zinc	Combinations of Yogurt **plus** *L*. *bulgaricus* **plus** *S*. *thermophilus* **plus** zinc
**ZN**	Zinc	Zinc (acetate or sulfate)
**ZN+LCF**	Zinc + Lactose-Free Formula	Combinations of zinc **plus** lactose-free formula
**ZN+MN**	Zinc + Additional Micronutrients	Combinations of zinc (Dose: 2 RDA-recommended dietary allowance, in syrup or tablets plus additional MN preparations including different doses of vitamin A, zinc, Vitamin D, Vitamin B1, riboflavin, Vitamin B6, nicotinamide, calcium. All micronutrients supplemented 1 RDA (Recommended dietary Allowance)
**ZN+PRB**	Combination of Zinc + Probiotics	Combinations of zinc (sulfate or acetate) + probiotics. Including one of the following: *Lactobacillus rhamnossus (R0011)*, *Lactobacillus sporogenes*, *Lactobacillus rhamnossus GG*, *Clostridium butyricum*

### Diarrhea duration

**Fig 3:**
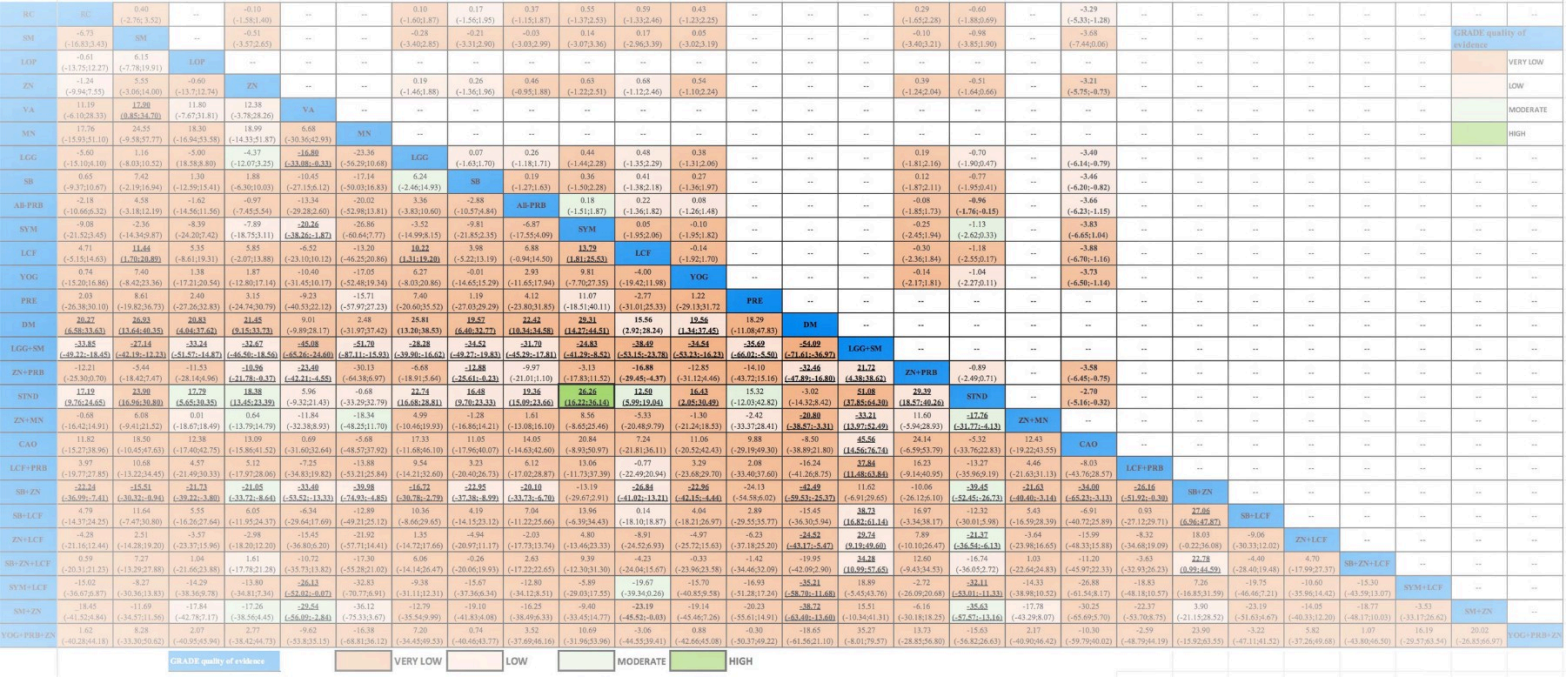
League table with NMA estimates for diarrhea duration and stool frequency at day 2. Comparisons should be read from left to right. The effectiveness estimate is located at the intersection of the column- defining treatment and the row-defining treatment. Diarrhea duration (bottom part of the table) effect estimate is presented in mean difference in hours with the 95% CrI, an MD below 0 favors the column- defining treatment (fewer hours of diarrhea). Stool frequency at day 2 (upper part of the table) effects estimate is presented in mean difference in the number of stools with the 95% CrI, an MD below 0 favours the column-defining treatment (fewer stools per day). To obtain MDs for comparisons in the opposing direction, negative values should be converted into positive values and vice versa. Significant results are in bold and underlined. Cells filling represent the GRADE quality of evidence assessment: Green: High quality; Light Green: Moderate quality; Light Orange: Low; Darker Orange: Very Low. Blank cells: not available interventions and comparisons MD: Mean difference; 95CrI%: 95% Credible Intervals; NMA: Network Meta- analysis. Interventions' abbreviations are described in Table 1.

From the pairwise meta-analyses, we obtained 62 comparisons (20,256 patients) that reported the outcome diarrhea duration (Table E in S1 Appendix). The heterogeneity measured with I^2^ was found moderate to high in most of direct comparisons that had two or more studies (Table E in S1 Appendix); in these instances, in the GRADE assessment, we rated down the quality of evidence of inconsistency. In the NMA, we obtained 351 paired estimates of which 62 had direct and indirect evidence, and 289 had only indirect evidence (Table E in S1 Appendix). VA, DM, and STND were the interventions more frequently inferior to other interventions. Among the single interventions, SYM showed the largest effect in comparison to STND [Mean difference (MD) = -26.26; Credible Interval (CrI) = -36.14 to -16.22]. Since the CrI of the Tau^2^ did not include the null value, between-study heterogeneity was considered significant [Tau^2^ = 99.6; CrI 82.15 to119.70]. No incoherence was found either with the global (p = 0.83), nor with the local assessment (Table F in S1 Appendix). Fig 3 presents the league table with the NMA estimates and GRADE assessments. Much of the evidence was judged low- or very-low quality, mostly because of risk of bias, inconsistency, and imprecision (Table E in S1 Appendix). Table 2 displays a summary of the primary outcome results integrating the effect estimates of diarrhea duration compared to STND and their quality. With high certainty, SB+ZN and SM+ZN were among the best interventions, while SYM, ZN+LCF, ZN, LOP ad ZN+MN were inferior to the best, yet better than the worst interventions, and PRE was among the worst, along with STND. The rest of interventions had low certainty.

**Table 2:** Summary of results for diarrhea duration in hours.

*Certainty on the evidence*	*Classification*	*Intervention*	*Intervention vs*. *Standard/placebo* *MD (95%CrI)*	*SUCRA (95%CrI)*
**High Certainty** *(Moderate- to High-quality evidence)*	**GROUP 1:** **Amongst the best interventions**	***S*. *boulardii* + Zinc (M)**	-39.4 (-52.4; -26.7)	0.92 (0.77; 1.00)
		**Smectite + Zinc (M)**	-35.6 (-57.6; -13.2)	0.88 (0.35; 1.00)
	**GROUP 2:** **Inferior to the best / Better than the worst interventions**	*Zinc (Inpatients)	-29.0 (-35.9; -22.1)	—
		**Symbiotics (H)**	-26.3 (-36.1; -16.2)	0.77 (0.38; 0.92)
		**Zinc + LCF (M)**	-21.4 (-36.5; -6.1)	0.61 (0.19; 0.92)
		**Zinc (All) (M)**	-18.4 (-23.4; -13.4)	0.50 (0.27; 0.69)
		*Zinc (LMIC)	-20.0 (-25.0; -15.0)	—
		**Loperamide (M)**	-17.8 (-30.3; -5.6)	0.46 (0.15; 0.85)
		**Zinc + Micronutrients (M)**	-17.8 (-31.8; -4.1)	0.46 (0.15; 0.85)
	**GROUP 3:** **Amongst the worst interventions**	**Prebiotics (M)**	-15.3 (-12.0; 42.8)	0.38 (0.00; 0.96)
		*Zinc (Outpatients)	-12.4 (-18.4; -6.5)	—
		*Zinc (HIC)	-11.4 (-3.3; 26.1)	—
**Low Certainty** *(Low- to Very Low-quality evidence)*	**GROUP 1:** **Amongst the best interventions**	**LGG + Smectite (VL)**	-51.1 (-64.3; -37.8)	1.00 (0.92; 1.00)
		*LGG (HIC)	-38.0 (-45.4; -30.5)	—
		**Zinc + Probiotics (L)**	-29.4 (-40.3; -18.6)	0.81 (0.5; 0.96)
	**GROUP 2:** **Inferior to the best / Better than the worst interventions**	**Symbiotics + LCF**	-32.1 (-53.0; -11.3)	0.85 (0.27; 1.00)
		**Smectite (VL)**	-23.9 (-30.8; -17.0)	0.69 (0.42; 0.88)
		**LGG (All) (L)**	-22.7 (-28.8; -16.7)	0.65 (0.38; 0.85)
		**All Probiotics (L)**	-19.4 (-23.7; -15.1)	0.54 (0.31; 0.73)
		**Racecadotril (L)**	-17.2 (-24.6; -9.8)	0.46 (0.23; 0.73)
		***S*. *boulardii (L)***	-16.5 (-23.3; -9.7)	0.42 (0.19; 0.69)
		**Yogurt (VL)**	-16.4 (-30.5; -2.0)	0.42 (0.11; 0.85)
		**LCF (VL)**	-12.5 (-19.0; -6.0)	0.31 (0.15; 0.54)
		*LGG (LMIC)	-11.7 (-19.7; -3.8)	—
	**GROUP 3:** **Amongst the worst interventions**	***S*. *boulardii* + Zinc + LCF (L)**	-16.7 (-36.0; 2.7)	0.42 (0.08; 0.88)
		**Yogurt + Probiotics + Zinc (VL)**	-15.6 (-56.8; 26.6)	0.38 (0.00; 1.00)
		**LCF + Probiotics (VL)**	-13.3 (-36.0; 9.2)	0.31 (0.00; 0.88)
		***S*. *boulardii* + LCF (VL)**	-12.3 (-30.0; 6.0)	0.27 (0.04; 0.81)
		**Vitamin A (VL)**	-5.9 (-21.4; 9.3)	0.19 (0.00; 0.61)
		**Kaolin-Pectin (VL)**	-5.3 (-33.8; 22.8)	0.15 (0.00; 0.89)
		**Micronutrients (L)**	-0.7 (-33.3; 32.8)	0.08 (0.00; 0.85)
		**Standard treatment/placebo**	—	0.08 (0.00; 0.19)
		**Diluted milk (VL)**	3.02 (-14.3; 8.4)	0.04 (0.00; 0.23)

**Classifications:** The two large groups (High and Low Certainty), are based on the GRADE quality of the evidence for the comparison of the intervention vs standard or placebo. **High certainty** (White part of the table; includes comparisons with moderate (M) to High quality (H) of evidence); Low **Certainty** (Grey part of the table; includes comparisons with low(L) to very low (VL) quality of evidence). Within each big groups there are 3 groups, clustered on the basis of their NMA effect estimates; **1) Group 1: Amongst the best interventions:** Interventions that were better than standard/placebo and better than the interventions that at the same time were better than standard/placebo; **2) Group 2: Inferior to the best and better than the worst interventions**: Includes interventions that were better than standard/placebo, but inferior to interventions from group (1); and **3) Group 3: Amongst the worst interventions:** includes standard/placebo and interventions with no differences in comparison to it. Interventions within each group showed no differences in the effect among them in the NMA.

*Results for two interventions (ZN, LGG), which showed differences in subgroups analyses; ZN(HIC), ZN (LMIC), LGG(HIC) and LGG/LMIC) effect estimates and classifications are displayed as well as the overall effect estimates: ZN (ALL) and LGG (ALL).

**-Acronyms: SUCRA**: surface under the *cumulative* ranking curve; **95%CrI**: 95% Credible Intervals; **MD:** Mean difference (in hours); **LCF**: Lactose Free formula; LMIC: Low- and Middle-Income countries; **HIC:** High-Income countries; LGG. *Lactobacillus-GG*

**Fig 4:**
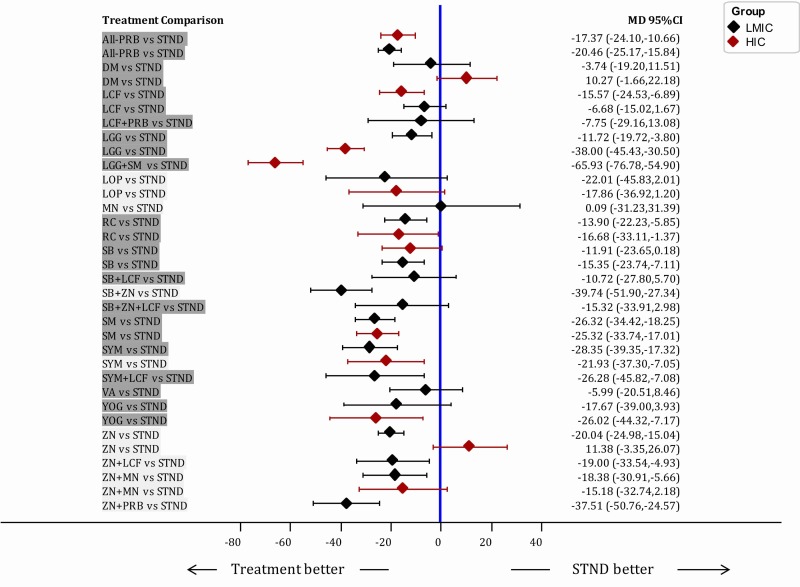
Forest plot of subgroup analyses by country income classification. All interventions are compared to STND. Subgroup analyses were performed based on the country income classification (based on the World Bank classification at 2016) Comparisons are highlighted in Dark Grey if the GRADE Quality was Low or Very Low, and in Light grey, when Quality was High or Moderate. When an intervention has no effect estimate in HIC means that there were no studies in HIC for the mentioned interventions. MD: Mean difference; 95CrI%: 95% Credible Intervals; LMIC: Low- and Middle-Income countries; HIC: High-income countries. Interventions' abbreviations are described in Table 1.

Sensitivity analyses showed similar between-study heterogeneity (Tau^2^) when excluding quasi-RCTs but the heterogeneity did decrease when analysing low risk of bias’ studies (Table G in S1 Appendix). The subgroup analyses by country classification (LMIC: 86 studies vs HIC: 34 studies) showed that LGG had a smaller effect on patients in LMIC, and higher effect in HIC patients (MD -38.0; CrI -45.43; -30.50) than in LMIC [MD -11.72; -19.72; -3.80], while ZN alone had no effect in HIC studies [MD 11.38; CrI .3.35; 26.07], and larger effect in LMIC [MD -20.04; CrI -24.98; -15.04] (Fig 4). Analyses by clinical setting (Inpatients: 68 studies, outpatients: 24 studies) showed some differences. Some interventions showed lower [LGG+SM; MD -16.27; CrI -32.57; -0.24] or no effect [YOG; MD -4.45; CrI -23.33; 14.48 and LA+LCF+ZN; MD -15.05; CrI -33.45; 3.05] when comparing to STND, in inpatients. ZN showed a larger effect in inpatients [MD = -29.0; CrI -35.9; -22.13] (Fig C and Fig D in S1 Appendix). Heterogeneity was reduced when pooling separately studies in LMIC, HIC, inpatients and outpatients (Table H in S1 Appendix) suggesting that part of the heterogeneity might be explained by these variables.

In the meta-regression, only publication year (not age, nor proportion of children with rotavirus) showed a significant coefficient [ß = -0.347; CrI -0.64 to -0.05] (Table I in S1 Appendix). This means that the older the publication of the study the less the impact on reducing the diarrhea duration; for each later year of publication of the study, the reduction in duration of diarrhea increased by 0.3 days.

### Stool frequency at day 2

NMA for stool frequency at day 2 included 33 studies (4,348 patients; 12 interventions). In the NMA (Fig 3) only ALL-PRB was slightly more effective than STND in reducing the number of stools [MD: -0.96; CrI -1.76 to -0.15] (Low quality of evidence). The evidence was low or very low quality in all cases, except for SYM vs STND (Moderate quality of evidence) (Fig E and Table J in S1 Appendix). There was not local or global incoherence (Table K in S1 Appendix). The heterogeneity was considered important since the CrI of the Tau^2^ included the null value (Tau^2^ = 0.89; CrI 0.38 to 2.03).

### Diarrhea at day 3

**Fig 5:**
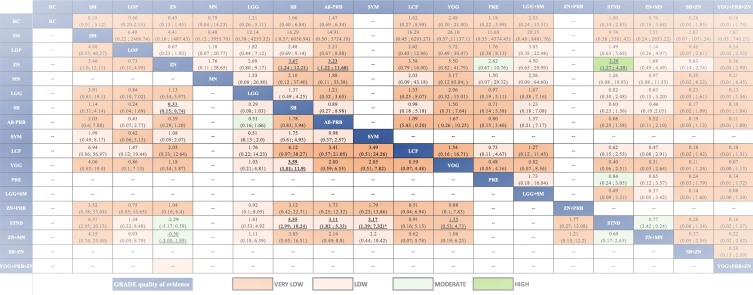
League table with NMA estimates for diarrhea at day 3 and vomiting. Comparisons should be read from left to right. The effectiveness estimate is located at the intersection of the column-defining treatment and the row-defining treatment. Diarrhea at day 3 (bottom part of the table) effect estimates are presented in odds ratio (OR) with the 95% CrI. A OR below 1.0 favours the column- defining treatment (less presence of diarrhea at day 3). Vomiting (upper part of the table) effect estimates are presented in Odds ratio (OR) with the 95% CrI, an OR below 1.0 favors the column-defining treatment (less presence of vomiting). To obtain ORs for comparisons in the opposing direction, reciprocals should be taken. Significant results are in bold and underlined font. Cells filling represent the GRADE quality of evidence assessment: Green: High quality; Light Green: Moderate quality; Light Orange: Low; Darker Orange: Very Low. Blank cells: not available interventions and comparisons. OR: Odds Ratio; 95CrI%: 95% Credible Intervals; NMA: Network Meta-analysis. Interventions' abbreviations are described in Table 1.

NMA for diarrhea at day 3 included 46 studies (10,339 patients; 12 interventions). SYM, SB, ZN, SM and ALL-PRB were more effective than STND, the rest were similar to STND. Fig 5 presents NMA estimates and GRADE assessment. In supporting information (Fig F in S1 Appendix) is displayed the forest plot for diarrhea at day 3 of all the interventions vs. STND, and the direct, indirect and NMA results of all the comparisons with their quality (Table L in S1 Appendix). One comparison (SYM vs STND) was found incoherent in the node-splitting assessment (Table M in S1 Appendix). The heterogeneity was considered important since the CrI of the Tau^2^ did not include the null value [Tau^2^ = 0.77; CrI 0.41 to 1.43].

### Vomiting

Twenty-three studies provided the proportion of children that had any episode of vomiting during the study follow-up (16 interventions; 5,671 patients). Fig 5 presents the NMA estimates and GRADE assessments. Only ZN produced more vomiting than STND [ZN vs STND: OR = 2.20; 1.27 to 4.28; high quality of evidence]. In supporting information (Fig G in S1 Appendix) is displayed the forest plot for vomiting of all the interventions vs STND, and the direct, indirect and NMA results of all the comparisons with their quality (Table N in S1 Appendix). The heterogeneity was considered important since the CrI of the Tau^2^ did not include the null value [Tau^2^ = 0.19; CrI 0.03 to 0.92]. No global or local incoherence was found (Table O in S1 Appendix).

### Side effects

**Fig 6:**
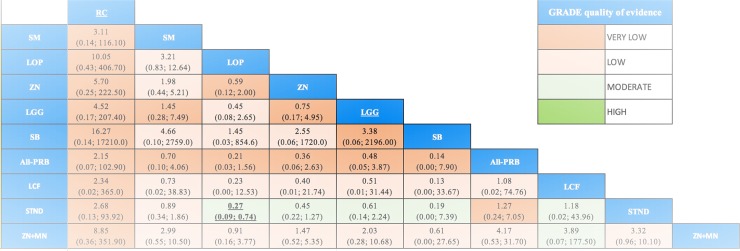
League table with NMA estimates for side effects. Comparisons should be read from left to right. The effectiveness estimate is located at the intersection of the column-defining treatment and the row-defining treatment. Side effects estimates are presented in odds ratio (OR) with the 95% CrI, an OR below 1.0 favors the row-defining treatment (less presence of side effects). To obtain ORs for comparisons in the opposing direction, reciprocals should be taken. Significant results are in bold and underlined. Cells filling represent the GRADE quality of evidence assessment: Green: High quality; Light Green: Moderate quality; Light Orange: Low; Darker Orange: Very Low. OR: Odds Ratio; 95CrI%: 95% Credible Intervals. Interventions' abbreviations are described in Table 1.

Ninety-five studies reported the measurement of side effects. Most studies failed to provide definitions of side effects and whenever presented, they differed. Thirty-six studies reported that no side effects were found, or that the interventions were well-tolerated, and 17 studies reported at least one patient with a side effect (4,419 patients; 10 interventions). Loperamide was the only intervention that showed more side effects than STND [OR = 0.27; CrI 0.09 to 0.74; moderate quality of evidence] (Fig 6). Although the wide CrI of the effect estimate for ZN+MN included the null value, the results do suggest the presence of side effects.

In supporting information (Fig H in S1 Appendix) we display the forest plot for side effects of all the interventions vs STND, and we present the direct, indirect and NMA results of all the comparisons with their quality (Table P in S1 Appendix). We found some heterogeneity in the results since the CrI of the Tau^2^ included the null value [Tau^2^ = 0.19; CrI 0.00 to 1.48]. The global chi-square test suggested the presence of incoherence (p = 0.009) and the local assessment showed incoherence in 2 comparisons (p = 0.04) (Table Q in S1 Appendix).

### Additional analyses

SUCRA values and CrI for all studies and only RCTs for secondary outcomes (Table R in S1 Appendix), the rank-heat plot, which summarizes the interventions SUCRAs across all the outcomes (Fig I in S1 Appendix), as well as the information for diarrhea at day 7 (Table S in S1 Appendix), and treatment failure (Table T in S1 Appendix), and the funnel plots for each outcome (Fig J in S1 Appendix) are detailed in the supporting information.

## Discussion

In this systematic review and NMA of all the standard pharmacological and nutritional interventions for reducing the duration of the ADG in children, we combined direct and indirect evidence from 180 studies in 32,832 children. We found that the interventions ALL-PRB, LGG, LCF, LOP, RC, SB, SM, SYM, YOG, ZN, and the combinations SB+ZN, ZN+LCF, ZN+LCF, SM+ZN, SYM+LCF, ZN+MN, ZN+PRB, and LGG+ZN, were better than placebo or standard treatment for reducing the diarrhea duration (12.5–26.3 hours and 17.8–51.1 hours less, single interventions and combinations, respectively). With high certainty, the combinations SB+ZN, SM+ZN were the only two interventions that, on the basis of high or moderate quality evidence, proved superior to both STND and to all other interventions. SYM, ZN, LOP, ZN+MN, ZN+LCF, demonstrated moderate quality evidence of superiority to STND and did not differ convincingly from one another. PRE proved, on the basis of moderate quality evidence, to be no better than STND. Additionally, with high certainty, we found that the effect of ZN was higher in LMIC, absent in HIC, and higher in inpatients. Therefore, the results for ZN and for SM+ZN and SB+ZN (because the results from SB+ZN and SM+ZN depend on the evidence that comes from the studies comparing ZN vs STND) might only be applied to patients in LMIC.

We found global statistical between-study heterogeneity (measured by Tau^2^), which was partially explained by country income, risk of bias, and hospital setting, but not by etiology. The lack of information about the infectious causes in many studies and the fact that our analysis was only based on the differences in % of children with rotavirus may be the reasons why the etiology did not explain the variability. The effect of some interventions may differ depending on the microorganism causing the disease and the specific contexts and countries. Heterogeneity at the level of the direct comparisons level was also moderate to high in many comparisons. This had an impact on the GRADE quality assessment on many of the direct comparisons, and partially explains why the majority of the comparisons were not of high or moderate quality. Indeed, Table 2 highlights the trustworthy comparisons (those with high or moderate quality) and distinguishes from the less trustworthy (low or very low quality).

Diarrhea duration in hours is a straightforward outcome from a clinical point of view. However, the extent to which a difference of reducing the duration in hours is important is open to question. To date there is no clear threshold to determine how many hours of difference in reduction patients and families are likely to consider important. We could hypothesize that a difference of less that 12 hours might not be important, but more evidence, particularly including the parents’ points of view is needed. Efforts in the characterization and standardization of these outcomes have been proposed, such as the inclusion of quality of life and functional status; they have not, however, been consistently implemented[41]. Further research to define not only what reduction in diarrhea duration parents consider important, but which other outcomes are the most important, is warranted.

Based on the meta-regression, the year of publication may have had an impact on the effect estimates. We included trials from 1968 to 2017, and it is well-know that the severity, the management of the ADG and the availability of interventions have changed substantially in last decades. Therefore, the potential impact of the year of publication on the estimates might not apply to all the interventions. However, the impact of the year of publication on the effect estimates may also be explained by publication bias. It has been described that the publication bias may be larger in meta-analysis that include older studies in comparison to those that include more recent studies[42].

Regarding secondary outcomes, with moderate to high quality evidence: SYM was similar to STND (stool frequency at day 2); and SYM and ZN were the only interventions better than STND (Diarrhea at day 3); ZN was the only one worse than STND (vomiting); and LOP was the only one worse than STND (side effects). Also, ZN+MN results suggested the possibility of important side effects, while LOP and RC were the only interventions with reported significant side effects (narrative summary). Results from secondary outcomes may be considered similar to the primary outcome, and the differences may be explained by differences in the power of the analyses, imprecision for these outcomes (considering the lower number of trials and patients for these outcomes), and because diarrhea duration would be a more comprehensive measure than measuring stool frequency or presence of diarrhea at a specific point in time.

The only previous NMA concluded that RC followed by smectite and *L*. *reuteri* were the best interventions for reducing the duration of diarrhea[43]. We found RC was also found better than STND but inferior to the best interventions, and its evidence quality was judged as low. Additionally, RC was one of the interventions for which at least one significant SE was reported (transaminases elevation). The differences between their results and ours may be explained by several reasons: the larger number of interventions and studies we analyzed, the inclusion of more outcomes, the analysis of the uncertainty around wide SUCRAs’ CrIs, the assessment of coherence and transitivity assumptions, and the GRADE quality of evidence assessment.

Several published or ongoing pairwise meta-analyses have summarized, the evidence of interventions in comparison to STND: all[12,44,45] or single probiotics[46–48], ZN[10,11,49], LCF[16], SM[14,50,51], RC[13,52–54], YOG[55] and LOP[56]. All, except by DM[16], were effective in reducing the diarrhea duration. Consequently, all the previous interventions have been recommended for use in different contexts around the world[57–60]. Although we found similar effects for many interventions, this NMA provides new results (symbiotics and 13 combinations that had not been summarized before), reaffirms the lack of safety of loperamide in children, reports the impact of several variables that may play a role as effect modifiers, presents effect estimates of the interventions not only in comparison to STND but also to one another, identifies research gaps, and is the first source of evidence of all the available interventions and comparisons in one study.

Lastly, consistent with previous reviews, ZN was associated with vomiting[61]. ZN is the only intervention recommended by the WHO the ADG treatment[4] and most of its use has been implemented in LMIC, where micronutrient deficiencies are prevalent[62]. ZN showed an important effect in LMIC studies and no effect in HIC studies, which may be associated with higher prevalence of zinc deficiency in these countries. ZN showed effectiveness with moderate quality of evidence, is inexpensive, has shown cost-effectiveness in LMIC contexts[63–66], and therefore, it may continue being used in these contexts. The increase in vomiting may limit its use, particularly in patients who present with significant vomiting in the emergency department. Unfortunately, based on the information provided by the studies. it is not possible to determine how significant the vomiting episodes might be. Considering that the use of ZN is common and widely implemented in children with ADG all around the world, particularly in LMIC, the increase in vomiting episodes with ZN supplementation may not be a major barrier.

Strengths of this review include the consideration of all the interventions, pharmacological and non-pharmacological (this is the first review that considered all the interventions), tested in RCTs, including some not previously summarized: symbiotics, prebiotics, vitamin A, micronutrients, as well as combinations. In addition to providing effect estimates and CrI for each paired comparison, we used the GRADE approach to assess their quality which includes evaluation of the main assumptions of a NMA, transitivity and coherence–for these assessments, we used both global and local tests. Further, we have explored possible effect modifiers including patients’ age, publication year, extent of Risk of bias, country income, and clinical setting. Lastly, we followed the Cochrane Handbook^24^ and ISPOR guidelines for developing rigorous NMA[67].

The review might also have some limitations. First, our results should be interpreted with caution given the high heterogeneity that we found, which was not completely explained by the variables that we considered in our analyses. Many other variables should be considered in future RCTs to make the studies more homogeneous, such as conducting studies in children in specific subgroups based on the etiology, clinical status, and context, and different combinations of these factors.

Although we found that two combinations (SB+ZN and SM+ZN) seem to be amongst the best interventions in comparison to the rest, both interventions have only been studied in patients in LMIC, in which ZN has shown being effective presumably in relation with higher prevalence of this micronutrient deficiency in children from these countries. The potential advantage of these two interventions in comparison to the rest, in HIC, should be studied in further RCTs.

Furthermore, the evidence included a limited number of direct comparisons–most network estimates relied on less trustworthy indirect comparisons. The scarcity of direct evidence partially explains the low or very low quality of evidence in most of comparisons (335 out of 351), which limits the conclusions about interventions classified as in the low certainty group. Additionally, to deal with the large number of interventions, we created clusters, taking the risk that effects would differ across treatment within clusters (A possibility we were not able to address statistically) which may have increased the heterogeneity or may have caused incoherence. Probiotics, for example, were clustered in three groups: LGG, SB and the rest of probiotics (ALL-PRB). The latter includes a very heterogeneous list of probiotics’ strains. This approach facilitated the analysis but however, it might hide differences among different less studied strains. Further research focused on all the strains that have been tested so far, might help to find differences in effectiveness, if they indeed exist.

Meta-regression results should be interpreted with caution. Because meta-regression is underpowered to detect small associations, negative results do not completely rule out the potential effect of the analyzed variables. Therefore, our findings that age, days with diarrhea, and etiology showed no impact on the treatments’ effects may be due to low power to detect them. Also, in most direct comparisons it was not possible to assess the risk of publication bias since there were few studies. Yet, in those comparisons that had a high number of studies (STND in comparison to ZN, probiotics, LCF, SM, and RC), we could evaluate the risk of publication bias as part of the GRADE quality of evidence assessment.

Lastly, the SUCRA values were extremely imprecise; they are thus of limited value, a problem some authors have highlighted[36,68]. Thus, in deciding on the relative merits of treatment, we have focused on the effect estimates and the evidence quality, with SUCRA rankings as a secondary factor.

Our review may be useful for clinicians, decision-makers, trialists and guidelines’ developers. It is surprising that with so many interventions available most of the studies are comparing interventions vs. STND. Researchers should focus on directly comparing the most effective interventions among them, instead of comparing against placebo and should make efforts to design trials of higher quality. Also, with the aim of reducing the heterogeneity, further trials focused on ADG caused by specific microorganisms, in specific contexts (e.g., studies specifically with inpatients with rotavirus infection in LMIC, inpatients with non-rotavirus disease in HIC, outpatients with non-rotavirus disease in LMIC, or outpatients with rotavirus disease in LMIC, etc.) should be encouraged. Moreover, efforts in the characterization and standardization of the outcomes to be measured in ADG trials have been proposed, such as the inclusion of quality of life and functional status, but they have not been consistently implemented[41]. Further research to define which outcomes are the most important for parents in different contexts, is warranted.

The interventions considered the best were all combinations. Single interventions, although superior to no treatment/placebo, were inferior to the best combinations and similar when compared one to each other. The use of combinations in LMIC settings might not be considered feasible or cost-effective. More research on the cost-effectiveness of these interventions in specific contexts, is warranted. In the meantime, since there were no differences among the single interventions for reducing the duration, clinicians who wish to use only one intervention may make their choice among the interventions that were in group 2 and had high certainty on the evidence, on the basis of issues other than effectiveness such as convenience, costs or safety. Clinicians should probably restrict themselves to the specific intervention studies–for instance, the five symbiotics chosen by trial investigators in HIC (Table 1), or, in LMIC, to ZN. Clinicians should probably avoid interventions that were similar to no treatment or that had low certainty (Bottom half of Table 2), and loperamide, which although was effective (moderate quality) was harmful. Although widely used in adults, loperamide is not recommended for use in young children[56,69] and our findings support this recommendation.

## Conclusions

All the interventions analysed, except vitamin A, micronutrients, prebiotics, kaolin-pectin, and selected combinations, were better than placebo to reduce the duration of the ADG in children. However, the effects are modest, differences among the interventions are small or absent, and the quality of evidence is mostly low or very low. With high certainty, only *S*. *boulardii* + zinc, and smectite + zinc, were considered amongst the best interventions; and symbiotics and zinc, were better than the worst and inferior to the best interventions. Zinc showed an important effect in LMIC. More confident judgements require additional high-quality RCTs comparing interventions that have shown being better than placebo, to one another. Results should be interpreted with caution given the high heterogeneity we found. New studies comparing interventions against no treatment or placebo or STND, studies with loperamide, micronutrients, kaolin-pectin, vitamin A, prebiotics and diluted milk should be discouraged in children.

## Supporting information

S1 Appendix Supporting information files. (DOCX)
