# Supplementary material for: Comparative effectiveness and safety of interventions for acute diarrhea and gastroenteritis in children: A systematic review and network meta-analysis
Source: PLoS One. 2018 Dec 5;13(12):e0207701. doi: 10.1371/journal.pone.0207701 (PMC6281220; doi:10.1371/journal.pone.0207701)
Supplement: S1 Appendix — (DOCX) [file pone.0207701.s001.docx]

**S1 Appendix**

Table of Contents

[File A: Methods and Protocol information 2](#_Toc529651124)

[File B: PRISMA NMA-Checklist 4](#_Toc529651125)

[File C: Search strategy and results 7](#_Toc529651126)

[Table A: Excluded Studies and Reasons 15](#_Toc529651127)

[Table B: Included Studies (N=174) 23](#_Toc529651128)

[Table C: Risk of Bias of Included Studies 45](#_Toc529651129)

[Table D: Descriptive transitivity analyses 49](#_Toc529651130)

[Table E: Direct, indirect, and NMA estimates for diarrhea duration with the GRADE Assessment 52](#_Toc529651131)

[Table F: Assessment of Incoherence for indirect comparisons 62](#_Toc529651132)

[Table G: Sensitivity analyses and SUCRA Values for Diarrhea duration 64](#_Toc529651133)

[Table H: Subgroup analyses and SUCRA values for each intervention for Diarrhea duration 65](#_Toc529651134)

[Fig A: NMA Forest Plots of interventions vs STND for subgroups analyses of all RCTs vs LMIC* 66](#_Toc529651135)

[Fig B: NMA Forest Plots of interventions vs STND for subgroups analyses of all RCTs vs HIC* 67](#_Toc529651136)

[Fig C: NMA Forest Plots interventions vs STND for subgroups analyses all RCTs vs Inpatients* 68](#_Toc529651137)

[Fig D: NMA Forest Plots interventions vs STND for subgroups analyses all RCTs vs outpatients* 69](#_Toc529651138)

[Table I: Meta-regression analyses and SUCRA values for each intervention for Diarrhea duration 70](#_Toc529651139)

[Fig E: NMA Forest plots: interventions vs. STND for Stool Frequency at day 2 71](#_Toc529651140)

[Table J: Direct, Indirect and NMA estimates for Stool frequency at day 2 and GRADE Assessment 72](#_Toc529651141)

[Table K: Assessment of Incoherence for Indirect Comparisons (Stool frequency day 2) 75](#_Toc529651142)

[Fig F: NMA Forest plot of all interventions vs. STND for diarrhea at day 3 76](#_Toc529651143)

[Table L: Direct, Indirect and NMA Estimates for Diarrhea at Day 3 with the GRADE Assessment 77](#_Toc529651144)

[Table M: Assessment of incoherence for indirect comparisons (Diarrhea day 3) 79](#_Toc529651145)

[Fig G: NMA Forest plot of all interventions vs. STND for Vomiting 80](#_Toc529651146)

[Table N: Direct, Indirect, and NMA Estimates for Vomiting with the GRADE Assessment 81](#_Toc529651147)

[Table O: Assessment of Incoherence for indirect comparisons (Vomiting) 85](#_Toc529651148)

[Fig H: NMA Forest plots of all interventions vs. STND for Side effects 86](#_Toc529651149)

[Table P: Direct, Indirect, and NMA Estimates for Side effects with the GRADE Assessment 87](#_Toc529651150)

[Table Q: Assessment of Incoherence for Indirect Comparisons (Side Effects) 89](#_Toc529651151)

[Table R: Sensitivity Analyses & SUCRA for Secondary outcomes 90](#_Toc529651152)

[Fig I: Rank Heat Plot 91](#_Toc529651153)

[Table S: Results for Diarrhea at day 7 92](#_Toc529651154)

[Table T: Results for Treatment Failure 93](#_Toc529651155)

[Fig J: Funnel plots for all the outcomes (A, B, C, D, E) 95](#_Toc529651156)

[File D: Full references of included studies 97](#_Toc529651157)

# File A: Methods and Protocol information

This review and NMA was registered in PROSPERO (CRD42015023778), and the protocol was published (Florez et al. 2016): <https://systematicreviewsjournal.biomedcentral.com/articles/10.1186/s13643-016-0186-8>

**I. Interventions and Classifications**

We included studies that evaluated loperamide. We did not initially consider this intervention because it has been contraindicated in children under 3 years old. However, we discussed that this intervention should be part of the review, since it may be used in older children and there was evidence from RCT available to summarize its effectiveness and safety.

We defined combinations of intervention the simultaneous use of two or more interventions, except for probiotics (commonly, they include preparations of different strains) and symbiotics (combination of probiotics and prebiotics), which were considered single interventions.

**II. Outcomes**

Main outcome: For our main outcome (diarrhea duration), we required studies to provide means and standard deviations. Although 144 publications provided the outcome diarrhea duration, eleven studies were excluded; Four compared different strains of probiotics among them and were included in the same intervention group, and seven studies presented effect using geometric means, which cannot be converted to absolute differences in means (eTable 1). Thus, we included 137 publications providing 142 registries (5 studies provided data split in subgroups by age groups, or country), were analyzed for this outcome (eTable 2).

Side effects: We aimed to summarize side effects as one outcome. However, we found high heterogeneity in definitions of the adverse events. As a post-hoc analysis, we: (1) performed separated analyses for vomiting and for the rest of side effects; (2) we narratively described side effects that we considered as significant side effects. We defined significant side effects as those events that could be related to the need for additional interventions or tests, with the potential to require hospital admission or, if already admitted, with the potential to extend the hospital stay.

The events that met our definition for SSE were constipation, abdominal distension, abdominal pain, ileus, somnolence, transaminases elevation, lower respiratory infection, skin infection, measles, and septicemia. Ten studies reported at least one of the previous events (references # 7,12,46,55,69,86,98,150,160,169). Abdominal pain was more common with STND than with ALL-PRB group in the only study (Henker 2008) that explicitly described this event (4/21 vs. 1/22). Constipation was more with SM group than with STND in 2 studies (Lexombon 1994, Freedman 2015) and was also more common with LOP than with RC (Turck 1999). Abdominal distension was found in one child (N=203 children) of the LOP group vs. no cases in the STND group (Anonymous 1984). One study described as SSE: lower respiratory infections, vulval abscess, and measles. These were found in 4 of 65 children with ALL-PRB group, and in 1 out of 59 children with STND (Sindhu 2014). An additional study reported SSE but did not describe what type of events were considered (Dupont 2009). Somnolence was described in one study in 2 children with LOP in comparison to 1 child with STND (Kaplan 1999). In one study an elevation of transaminases was described in one child with RC, and none with STND (Santos 2009). One study (Basu 2009) reported septicemia (LGG in 4 patients and STND, 3 patients).

Additional outcomes: Mortality, diarrhea at day 7, hospitalizations (Dichotomous outcome) and treatment failure were planned a priori as outcomes of interest. Nevertheless, the information obtained for them was scarce or very heterogeneous and they were summarized narratively as explained below.

Diarrhea at day 7 (16 studies, 6 interventions), had very heterogeneous definitions among the included studies, and therefore were not pooled together. Some studies defined the outcome as: diarrhea at day 7 post-randomization, others as diarrhea >7 days post-randomization, and others as diarrhea at day 7^th^ after the diarrhea onset (not post-randomization). Table 18 summarizes these results.

Regarding treatment failure, 37 studies reported very heterogeneous definitions, ranging from diarrhea duration (diarrhea > 5, >7 or more than 14 days), hydration status, vomiting, need for IV, lactose malabsorption, or stool output, among others). We decided not to perform NMA with this data considering the huge heterogeneity of the clinical definitions. eTables 19 summarizes the findings.

Only 5 studies reported deaths (Awasthu 2006, Basu 2007, Basu 2009, Karrar 1987, Patel 2009); references #9,11,12,88,127): Four in LMIC and one in a HIC. Two studies analyzing ZN, 2 studies LGG, and one study LOP, in comparison to STND. In the LGG studies, one death in each one occurred in STND groups, in one ZN study, there was one death in the ZN group and none in the STND group, and the other ZN study there was one death, but authors did not describe in which group. In the LOP study, the only death was with LOP. Studies did not describe deaths causes (only one death was attributed to septicaemia by *Salmonella* no-*typhi –*LOP group*)* but in all the cases the deaths were considered not associated to any of the interventions.

**II. Additional Analyses**

Transitivity assumption assessment: We assessed the transitivity assumption for each outcome *a priori* using the following effect modifiers: year of publication, country income, age, days with diarrhea, clinical status, and etiology (measured as proportion of rotavirus: %RV). If intransitivity was found in any given comparison, we rated down the quality of the evidence for that indirect estimate (see manuscript the section: *Rating the quality of the evidence in the estimates*).

Subgroups analyses and Meta-regression: We proposed a priori, a subgroup analysis based on the nutritional status or the micronutrient deficiencies. However, we did not find enough information to perform this analysis.

We performed a post-hoc meta-regression analysis based on the year of publication trying to explore additional sources of heterogeneity, since we observed published studies from the late sixties to date.

We decided to exclude trials of zinc in infants below 6 months of age. We did this because this is very specific population of infants that are usually breastfed, in which zinc supplementation will not be expected to have an effect, and indeed the evidence has shown that there were no differences in diarrhea duration between zinc and placebo (Lazzerini et al. 2016; reference #10 in manuscript). Including these studies would be related to lower effect of zinc and not a reflect of the clinical use. Thus, conclusions about zinc in this study will only be applicable to children older than 6 months.

# File B: PRISMA NMA-Checklist

***(PRISMA NMA Checklist of Items to Include When Reporting A Systematic Review Involving a Network Meta-analysis)***

| **Section/Topic** | **Item #** | **Checklist Item** | **Reported on Page #** |
| --- | --- | --- | --- |
| **TITLE** |  |  |  |
| Title | 1 | Identify the report as a systematic review *incorporating a network meta-analysis (or related form of meta-analysis).* | **1** |
| **ABSTRACT** |  |  |  |
| Structured summary | 2 | Provide a structured summary including, as applicable:  **Background:** main objectives  **Methods:** data sources; study eligibility criteria, participants, and interventions; study appraisal; and *synthesis methods, such as network meta-analysis.*  **Results:** number of studies and participants identified; summary estimates with corresponding confidence/credible intervals; *treatment rankings may also be discussed. Authors may choose to summarize pairwise comparisons against a chosen treatment included in their analyses for brevity.*  **Discussion/Conclusions:** limitations; conclusions and implications of findings.  **Other:** primary source of funding; systematic review registration number with registry name. | 2 |
|  |  |  |  |
| **INTRODUCTION** |  |  |  |
| Rationale | 3 | Describe the rationale for the review in the context of what is already known*, including mention of why a network meta-analysis has been conducted.* | **3,4** |
| Objectives | 4 | Provide an explicit statement of questions being addressed, with reference to participants, interventions, comparisons, outcomes, and study design (PICOS). | 4 |
|  |  |  |  |
| **METHODS** |  |  |  |
| Protocol and registration | 5 | Indicate whether a review protocol exists and if and where it can be accessed (e.g., Web address); and, if available, provide registration information, including registration number. | 5 |
| Eligibility criteria | 6 | Specify study characteristics (e.g., PICOS, length of follow-up) and report characteristics (e.g., years considered, language, publication status) used as criteria for eligibility, giving rationale. *Clearly describe eligible treatments included in the treatment network, and note whether any have been clustered or merged into the same node (with justification).* | **Pages 4,5**  **&**  Protocol publication (Florez 2016) |
| Information sources | 7 | Describe all information sources (e.g., databases with dates of coverage, contact with study authors to identify additional studies) in the search and date last searched. | 4 |
| Search | 8 | Present full electronic search strategy for at least one database, including any limits used, such that it could be repeated. | Page 4,  Protocol publication (Florez et al. 2016)  &  S1 Appendix (page 7) |
| Study selection | 9 | State the process for selecting studies (i.e., screening, eligibility, included in systematic review, and, if applicable, included in the meta-analysis). | Page 4,5  &  Protocol publication (Florez et al. 2016) |
| Data collection process | 10 | Describe method of data extraction from reports (e.g., piloted forms, independently, in duplicate) and any processes for obtaining and confirming data from investigators. | Pages 5,6  &  Protocol publication (Florez et al. 2016) |
| Data items | 11 | List and define all variables for which data were sought (e.g., PICOS, funding sources) and any assumptions and simplifications made. | Page 4,5  &  Protocol publication (Florez et al. 2016) |
| **Geometry of the network** | **S1** | Describe methods used to explore the geometry of the treatment network under study and potential biases related to it. This should include how the evidence base has been graphically summarized for presentation, and what characteristics were compiled and used to describe the evidence base to readers. | **Page 9**  **& Figure 2** |
| Risk of bias within individual studies | 12 | Describe methods used for assessing risk of bias of individual studies (including specification of whether this was done at the study or outcome level), and how this information is to be used in any data synthesis. | -Page 6 & Protocol Publication /Florez 2016 (RoB tool)  -Page 8 & Protocol Publication-Florez et al. 2016 (How RoB was used in the analysis: Using GRADE)  -Protocol publication (Florez et al. 2016) (How RoB was used to inform the GRADE assessment) |
| Summary measures | 13 | State the principal summary measures (e.g., risk ratio, difference in means). *Also describe the use of additional summary measures assessed, such as treatment rankings and surface under the cumulative ranking curve (SUCRA) values, as well as modified approaches used to present summary findings from meta-analyses.* | Pages 6,7,8  &  Protocol publication (Florez et al. 2016) |
| Planned methods of analysis | 14 | Describe the methods of handling data and combining results of studies for each network meta-analysis. This should include, but not be limited to:   - *Handling of multi-arm trials;* - *Selection of variance structure;* - *Selection of prior distributions in Bayesian analyses; and* - *Assessment of model fit.* | Pages 6,7, and 8  &  Protocol publication (Florez et al. 2016) |
| **Assessment of Inconsistency** | **S2** | Describe the statistical methods used to evaluate the agreement of direct and indirect evidence in the treatment network(s) studied. Describe efforts taken to address its presence when found. | -Page 7 (Inconsistency/incoherence assessment and how we handled it);  -Page 8 (Incoherence as a criterion for GRADE quality assessment- Based on Puhan 2014)  &  -Protocol publication (Florez et al. 2016) in methods |
| Risk of bias across studies | 15 | Specify any assessment of risk of bias that may affect the cumulative evidence (e.g., publication bias, selective reporting within studies). | Page 8 (Use of GRADE to assess the risk of bias across the studies)  &  Protocol publication (Florez et al. 2016) |
| Additional analyses | 16 | Describe methods of additional analyses if done, indicating which were pre-specified. This may include, but not be limited to, the following:   - Sensitivity or subgroup analyses; - Meta-regression analyses; - *Alternative formulations of the treatment network; and* - *Use of alternative prior distributions for Bayesian analyses (if applicable).* | S1 Appendix (page 71)  &  Protocol publication (Florez et al. 2016) |
| **RESULTS†** |  |  |  |
| Study selection | 17 | Give numbers of studies screened, assessed for eligibility, and included in the review, with reasons for exclusions at each stage, ideally with a flow diagram. | Page 9 ;  Figure 1 ;  S1 Appendix (Page 15 and page 23) |
| **Presentation of network structure** | **S3** | Provide a network graph of the included studies to enable visualization of the geometry of the treatment network. | **Figure 2** |
| **Summary of network geometry** | **S4** | Provide a brief overview of characteristics of the treatment network. This may include commentary on the abundance of trials and randomized patients for the different interventions and pairwise comparisons in the network, gaps of evidence in the treatment network, and potential biases reflected by the network structure. | **Page 9** |
| Study characteristics | 18 | For each study, present characteristics for which data were extracted (e.g., study size, PICOS, follow-up period) and provide the citations. | S1 Appendix (Page 23) |
| Risk of bias within studies | 19 | Present data on risk of bias of each study and, if available, any outcome level assessment. | S1 Appendix (Page 46) |
| Results of individual studies | 20 | For all outcomes considered (benefits or harms), present, for each study: 1) simple summary data for each intervention group, and 2) effect estimates and confidence intervals. *Modified approaches may be needed to deal with information from larger networks.* | This is a Large network that makes difficult to present all the estimates for each study. As an alternative approach, we summarized the information from studies in Supplemental table 2 and the results from all the direct comparisons (#62) across appendices (for each outcome) |
| Synthesis of results | 21 | Present results of each meta-analysis done, including confidence/credible intervals. *In larger networks, authors may focus on comparisons versus a particular comparator (e.g. placebo or standard care), with full findings presented in an appendix. League tables and forest plots may be considered to summarize pairwise comparisons.* If additional summary measures were explored (such as treatment rankings), these should also be presented. | **NMA estimates (league tables and regular tables)**  Figure 3  Figure 4  Figure 5  S1 Appendix (Pages 53, 73, 78, 82, 88)  **Rankings**  S1 Appendix (Page 65, 92) |
| **Exploration for inconsistency** | **S5** | Describe results from investigations of inconsistency. This may include such information as measures of model fit to compare consistency and inconsistency models, *P* values from statistical tests, or summary of inconsistency estimates from different parts of the treatment network. | **Pages 11-14**  S1 Appendix (Pages 6, 11, 13, 15, 17) |
| Risk of bias across studies | 22 | Present results of any assessment of risk of bias across studies for the evidence base being studied. | S1 Appendix (Pages 96-97) |
| Results of additional analyses | 23 | Give results of additional analyses, if done (e.g., sensitivity or subgroup analyses, meta-regression analyses*, alternative network geometries studied, alternative choice of prior distributions for Bayesian analyses,* and so forth). | S1 Appendix (Page 65, 66, 67, 71) |
|  |  |  |  |
| **DISCUSSION** |  |  |  |
| Summary of evidence | 24 | Summarize the main findings, including the strength of evidence for each main outcome; consider their relevance to key groups (e.g., healthcare providers, users, and policy-makers). | Figures 3,4,5  Table 2  Discussion Pages 15-20 |
| Limitations | 25 | Discuss limitations at study and outcome level (e.g., risk of bias), and at review level (e.g., incomplete retrieval of identified research, reporting bias). *Comment on the validity of the assumptions, such as transitivity and consistency. Comment on any concerns regarding network geometry (e.g., avoidance of certain comparisons).* | Page 18 |
| Conclusions | 26 | Provide a general interpretation of the results in the context of other evidence, and implications for future research. | Page 20 |
|  |  |  |  |
| **FUNDING** |  |  |  |
| Funding | 27 | Describe sources of funding for the systematic review and other support (e.g., supply of data); role of funders for the systematic review. This should also include information regarding whether funding has been received from manufacturers of treatments in the network and/or whether some of the authors are content experts with professional conflicts of interest that could affect use of treatments in the network. | Title page (page 1) |

PICOS = population, intervention, comparators, outcomes, study design.

* Text in italics indicates wording specific to reporting of network meta-analyses that has been added to guidance from the PRISMA statement. † Authors may wish to plan for use of appendices to present

***Source:*** *Hutton B, Salanti G, Caldwell DM, Chaimani A, Schmid CH, Cameron C. The PRISMA extension statement for reporting of systematic reviews incorporating network meta-analyses of health care interventions: checklist and explanations. Ann Intern Med. 2015;162.*

# File C: Search strategy and results

Ovid MEDLINE(R) In-Process & Other Non-Indexed Citations, Ovid MEDLINE(R) Daily and Ovid MEDLINE(R) 1946 to Present

1. exp Diarrhea/

2. diarrh$.mp.

3. exp Gastroenteritis/

4. gastroenteritis.mp.

5. gastrointestinal infection$.mp.

6. enteritis.mp.

7. dysenter$.mp.

8. or/1-7

9. pr$biotic$.mp. or exp Probiotics/

10. exp Lactobacillus/

11. lactobacteri$.mp.

12. lactobacill$.mp.

13. reuteri.mp.

14. exp Saccharomyces/

15. exp Bifidobacterium/

16. saccharomyc$.mp.

17. boulardii.mp.

18. exp Bacillus/ or Bacillus.mp.

19. Subtilis.mp.

20. clausii.mp.

21. exp Enterococcus faecium/

22. enterococcus faecium.mp.

23. bifidobact$.mp.

24. rhamnosus.mp.

25. casei.mp.

26. thermophilus.mp.

27. acidophilus.mp.

28. plantarum.mp.

29. bulgaricus.mp.

30. lgg.mp.

31. bifidum.mp.

32. symbiotic$.mp.

33. or/9-32

34. exp Silicates/

35. silicate$.mp.

36. diosmectite.mp.

37. smectite.mp.

38. smecta.mp.

39. or/34-38

40. racecadotril.mp.

41. acetorphan.mp.

42. enkephalinase inhibitor$.mp.

43. exp Thiorphan/

44. thiorphan.mp.

45. tiorfan.mp.

46. tiorfix.mp.

47. hidrasec.mp.

48. or/40-47

49. exp Loperamide/

50. loperamide.mp.

51. imodium.mp.

52. or/49-51

53. exp Antidiarrheals/

54. antidiarrh$.tw.

55. or/53-54

56. exp Zinc/

57. exp Zinc Compounds/

58. exp Zinc Acetate/

59. zinc.mp.

60. gluconate.mp.

61. cinc.mp.

62. Trace Elements/

63. or/56-62

64. exp Vitamin A/

65. retinol.mp.

66. vitamin A.mp.

67. or/64-66

68. exp Yogurt/

69. exp Cultured Milk Products/

70. yogurt$.tw.

71. yogh?urt$.tw.

72. k?umis$.tw.

73. Kumys$.mp.

74. k?efir$.tw.

75. kephir.tw.

76. bulgaricus.tw.

77. doog.tw.

78. lassi.tw.

79. Matso?n$.tw.

80. Da?hi.tw.

81. viili.tw.

82. (Fermented adj2 milk).tw.

83. delbruecki$.tw.

84. (sour adj1 milk).tw.

85. or/68-84

86. exp Milk Substitutes/

87. exp Lactose Intolerance/

88. (Soy adj3 milk).tw.

89. (Soy adj3 formula).tw.

90. (lactose adj1 intolerance).mp.

91. (lactose$ adj2 formula).tw.

92. or/86-91

93. 33 or 39 or 48 or 52 or 55 or 63 or 67 or 85 or 92

94. 8 and 93

95. randomized controlled trial.pt.

96. randomized.mp.

97. blind$.mp.

98. placebo.mp.

99. or/95-98

100. 94 and 99

101. (Infan$ or newborn$ or new-born$ or perinat$ or neonat$ or baby or baby$ or babies or toddler$ or minors or minors$ or boy or boys or boyfriend or boyhood or girl$ or kid or kids or child or child$ or children$ or schoolchild$ or schoolchild).mp. or schoolchild.tw. or schoolchild$.tw. or adolescen$.mp. or juvenil$.mp. or youth$.mp. or teen$.mp. or under$age$.mp. or pubescen$.mp. or exp Pediatrics/ or pediatric$.mp. or paediatric$.mp. or peadiatric$.mp. or school.tw. or school$.tw. or prematur$.mp. or preterm$.mp.

102. 100 and 101

103. limit 102 to human

Embase 1974 to April 2017

1. exp Diarrhea/

2. diarrh$.mp.

3. exp Gastroenteritis/

4. gastroenteritis.mp.

5. gastrointestinal infection$.mp.

6. enteritis.mp.

7. dysenter$.mp.

8. or/1-7

9. pr?biotic$.mp. or exp Probiotics/

10. exp Lactobacillus/

11. lactobacteri$.mp.

12. lactobacill$.mp.

13. exp Saccharomyces/

14. exp Bifidobacterium/

15. saccharomyc$.mp.

16. boulardii.mp.

17. exp Bacillus/ or Bacillus.mp.

18. Subtilis.mp.

19. clausii.mp.

20. exp Enterococcus faecium/

21. enterococcus faecium.mp.

22. bifidobact$.mp.

23. rhamnosus.mp.

24. casei.mp.

25. reuteri.mp.

26. acidophilus.mp.

27. thermophilus.mp.

28. plantarum.mp.

29. bulgaricus.mp.

30. bifidum.mp.

31. LGG.mp.

32. symbiotic$.mp.

33. or/9-31

34. exp Silicates/

35. silicate$.mp.

36. diosmectite.mp.

37. smectite.mp.

38. smecta.mp.

39. or/34-38

40. racecadotril.mp.

41. acetorphan.mp.

42. enkephalinase inhibitor$.mp.

43. exp Thiorphan/

44. thiorphan.mp.

45. tiorfan.mp.

46. tiorfix.mp.

47. hidrasec.mp.

48. or/40-47

49. exp loperamide/

50. loperamide.mp.

51. imodium.mp.

52. or/49-51

53. exp Antidiarrheal agent/

54. antidiarrh$.tw.

55. or/53-54

56. exp Zinc/

57. exp Zinc Compounds/

58. exp Zinc Acetate/

59. zinc.mp.

60. gluconate.mp.

61. cinc.mp.

62. Trace Elements/

63. or/56-62

64. exp Retinol/

65. retinol.mp.

66. vitamin A.tw.

67. (vitamin adj A).tw.

68. or/64-67

69. exp Yoghurt/

70. exp Kefir/

71. exp Fermented product/

72. yogurt$.tw.

73. yogh?urt$.tw.

74. k?umis$.tw.

75. Kumys$.mp.

76. k?efir$.tw.

77. kephir.tw.

78. bulgaricus.tw.

79. doog.tw.

80. lassi.tw.

81. Matso?n$.tw.

82. Da?hi.tw.

83. viili.tw.

84. (Fermented adj2 milk).tw.

85. delbruecki$.tw.

86. (sour adj1 milk).tw.

87. or/69-86

88. exp Artificial milk/

89. exp Soy milk/

90. (Soy adj3 milk).tw.

91. (Soy adj3 formula).tw.

92. (lactose adj1 intolerance).mp.

93. (lactose$ adj2 formula).tw.

94. or/88-93

95. 33 or 39 or 48 or 52 or 55 or 63 or 68 or 87 or 94

96. 8 and 95

97. random:.tw.

98. blind$.tw.

99. placebo:.mp.

100. or/97-99

101. 96 and 100

102. (Infan$ or newborn$ or new-born$ or perinat$ or neonat$ or baby or baby$ or babies or toddler$ or minors or minors$ or boy or boys or boyfriend or boyhood or girl$ or kid or kids or child or child$ or children$ or schoolchild$ or schoolchild).mp. or schoolchild.tw. or schoolchild$.tw. or adolescen$.mp. or juvenil$.mp. or youth$.mp. or teen$.mp. or under$age$.mp. or pubescen$.mp. or exp Pediatrics/ or pediatric$.mp. or paediatric$.mp. or peadiatric$.mp. or school.tw. or school$.tw. or prematur$.mp. or preterm$.mp.

103. 101 and 102

104. limit 103 to human

Global health: 1973 to 2017 Week15

1. exp Diarrhoea/

2. diarrh$.mp.

3. exp Gastroenteritis/

4. gastroenteritis.mp.

5. gastrointestinal infection$.mp.

6. enteritis.mp.

7. dysenter$.mp.

8. or/1-6

9. pr$biotic$.mp. or exp Probiotics/

10. exp Lactobacillus/

11. lactobacteri$.mp.

12. lactobacill$.mp.

13. exp Saccharomyces/

14. exp Bifidobacterium/

15. saccharomyc$.mp.

16. boulardii.mp.

17. exp Bacillus/ or Bacillus.mp.

18. Subtilis.mp.

19. clausii.mp.

20. exp Enterococcus faecium/

21. enterococcus faecium.mp.

22. bifidobact$.mp.

23. rhamnosus.mp.

24. casei.mp.

25. reuteri.mp.

26. acidophilus.mp.

27. thermophilus.mp.

28. plantarum.mp.

29. bulgaricus.mp.

30. bifidum.mp.

31. LGG.mp.

32. symbiotic$.mp.

33. or/9-32

34. exp Silicates/

35. silicate$.mp.

36. diosmectite.mp.

37. smectite.mp.

38. smecta.mp.

39. or/34-38

40. racecadotril.mp.

41. acetorphan.mp.

42. enkephalinase inhibitor$.mp.

43. thiorphan.mp.

44. or/40-43

45. exp Loperamide/

46. loperamide.mp.

47. imodium.mp.

48. or/45-47

49. exp Antidiarrhoea agents/

50. antidiarrh$.tw.

51. or/49-50

52. exp Zinc/

53. zinc.mp.

54. gluconate.mp.

55. cinc.mp.

56. Trace Elements/

57. or/52-56

58. exp Retinol/

59. vitamin A.tw.

60. or/58-59

61. exp Yoghurt/

62. exp cultured milks/

63. exp Fermented foods/

64. exp Kefir/

65. exp Sour milk/

66. yogurt$.tw.

67. yogh?urt$.tw.

68. k?umis$.tw.

69. Kumys$.tw.

70. k?efir$.tw.

71. kephir.tw.

72. bulgaricus.tw.

73. doog.tw.

74. lassi.tw.

75. Matso?n$.tw.

76. Da?hi.tw.

77. viili.tw.

78. (Fermented adj2 milk).tw.

79. delbruecki$.tw.

80. (sour adj1 milk).tw.

81. or/61-80

82. exp Lactose Intolerance/

83. exp Soya Milk/

84. (Soy$1 adj3 milk).tw.

85. (Soy$1 adj3 formula).tw.

86. (lactose adj1 intolerance).mp.

87. (lactose$ adj2 formula).tw.

88. or/82-87

89. 33 or 39 or 44 or 48 or 51 or 57 or 60 or 81 or 88

90. 8 and 89

91. random$.tw.

92. blind$.tw.

93. placebo.tw.

94. (pragmatic adj2 trial).tw.

95. (pragmatic adj2 study).tw.

96. or/91-94

97. 90 and 96

98. (infan$ or newborn$ or new-born$ or perinat$ or neonat$ or baby or baby$ or babies or toddler$ or minors or minors$ or boy or boys or boyfriend or boyhood or girl$ or kid or kids or child or child$ or children$ or schoolchild$ or schoolchild).mp. or schoolchild.tw. or schoolchild$.tw. or adolescen$.mp. or juvenil$.mp. or youth$.mp. or teen$.mp. or under$age$.mp. or pubescen$.mp. or exp Paediatrics/ or pediatric$.mp. or paediatric$.mp. or peadiatric$.mp. or school.tw. or school$.tw. or prematur$.mp. or preterm$.mp.

99. 97 and 98

CENTRAL

#1 MeSH descriptor: [Diarrhea] explode all trees

#2 MeSH descriptor: [Gastroenteritis] explode all trees

#3 MeSH descriptor: [Dysentery] explode all trees

#4 diarrh*:ti,ab,kw (Word variations have been searched)

#5 gastroenteritis:ti,ab,kw (Word variations have been searched)

#6 gastrointestinal infection*:ti,ab,kw (Word variations have been searched)

#7 enteritis:ti,ab,kw (Word variations have been searched)

#8 dysenter*

#9 #1 or #2 or #3 or #4 or #5 or #6 or #7 or #8

#10 MeSH descriptor: [Saccharomyces] explode all trees

#11 MeSH descriptor: [Bifidobacterium] explode all trees

#12 MeSH descriptor: [Bacillus] explode all trees

#13 MeSH descriptor: [Enterococcus faecium] explode all trees

#14 MeSH descriptor: [Lactobacillus] explode all trees

#15 saccharomyc*

#16 boulardii

#17 subtilis

#18 clausii

#19 enterococcus faecium

#20 bifidobact*

#21 rhamnosus

#22 casei

#23 lgg

#24 acidophilus

#25 thermophilus

#26 plantarum

#27 bulgaricus

#28 bifidum

#29 reuteri

#30 lactobacteri*

#31 lactobacill*

#32 MeSH descriptor: [Probiotics] explode all trees

#33 probiotic*

#34 prebiotic*

#35 symbiotic*

#36 #10 or #11 or #12 or #13 or #14 or #15 or #16 or #17 or #18 or #19 or #20 or #21 or #22 or #23 or #24 or #25 or #26 or #27 or #28 or #29 or #30 or #31 or #32 or #33 or #34 or #35

#37 MeSH descriptor: [Silicates] explode all trees

#38 silicate*

#39 diosmectite

#40 smectite

#41 smecta

#42 #37 or #38 or #39 or #40 or #41

#43 MeSH descriptor: [Thiorphan] explode all trees

#44 racecadotril

#45 acetorphan

#46 enkephalinase inhibitor*

#47 hidrasec

#48 #43 or #44 or #45 or #46 or #47

#49 MeSH descriptor: [Loperamide] explode all trees

#50 loperamide (Word variations have been searched)

#51 imodium (Word variations have been searched)

#52 #49 or #50 or #51

#53 MeSH descriptor: [Antidiarrheals] explode all trees

#54 antidiarrh* (Word variations have been searched)

#55 #53 or #54

#56 MeSH descriptor: [Zinc] explode all trees

#57 MeSH descriptor: [Zinc Compounds] explode all trees

#58 zinc (Word variations have been searched)

#59 #56 or #57 or #58

#60 MeSH descriptor: [Vitamin A] explode all trees

#61 retinol

#62 retinoic

#63 vitamin a

#64 #60 or #61 or #62 or #63

#65 MeSH descriptor: [Cultured Milk Products] explode all trees

#66 MeSH descriptor: [Yogurt] explode all trees

#67 yogurt* (Word variations have been searched)

#68 yoghurt* (Word variations have been searched)

#69 kumis* (Word variations have been searched)

#70 kefir* (Word variations have been searched)

#71 bulgaricus

#72 lassi

#73 matson (Word variations have been searched)

#74 dahi (Word variations have been searched)

#75 viili

#76 fermented milk

#77 delbruecki*

#78 Sour milk (Word variations have been searched)

#79 #65 or #66 or #67 or #68 or #69 or #70 or #71 or #72 or #73 or #74 or #75 or #77 or #78

#80 MeSH descriptor: [Milk Substitutes] explode all trees

#81 MeSH descriptor: [Lactose Intolerance] explode all trees

#82 soy milk (Word variations have been searched)

#83 soy formula (Word variations have been searched)

#84 lactose intolerance (Word variations have been searched)

#85 lactose-free

#86 lactose formula

#87 #80 or #81 or #82 or #83 or #84 or #85 or #86

#88 #36 or #42 or #48 or #52 or #55 or #59 or #64 or #79 or #87

#89 #9 and #88

#90 CHILD* or INFAN* or ADOLESCEN* or NEWBORN* or PRESCHOOL* or KINDERGARTEN* or NURSERY SCHOOL or ELEMENTARY SCHOOL or TEEN or TEENS or TEENAGE* or UNDERAGE* or PREEMIE* or NEONAT* or YOUTH or YOUTHS ORUNDERAGE* or BABY or BABIES or PREPUBESCEN* or PUBESCEN* or SCHOOLCHILD* or DAYCARE* or SCHOOLAGE* or BOY* or GIRL* or OFFSPRING or PAEDIATRIC* or PEDIATRIC* or JUVENIL* or TODDLER* or NURSERY or NURSERIES or HIGH SCHOOL* OT HIGHSCHOOL* or PRIMARY SCHOOL* or SECONDARY SCHOOL*

#91 MeSH descriptor: [Pediatrics] explode all trees

#92 #90 or #91

#93 #89 and #92

#94 #89 and #92 in Trials

CINAHL

| S30 | S28 AND S29 |
| --- | --- |
| S29 | (pediatric* or paediatric* or child* or newborn* or congenital* or infan* or baby or babies or neonat* or “pre-term” or preterm or “premature birth*” or NICU or preschool* or “pre- school*” or kindergarten* or “elementary school*” or “nursery school*” or schoolchild* or toddler* or boy or boys or girl* or “middle school*” or pubescen* or juvenile* or teen* or youth* or “high school*” or adolesc*or prepubesc* or “pre-pubesc*” or "(MH "Child+") OR (MH "Adolescence+") OR (MH "Minors (Legal)") or "(MH "Child Abuse, Sexual") OR (MH "Child Behavior Disorders+") OR (MH "Child, Medically Fragile") OR (MH "Child Day Care") OR (MH "Child Behavior+") OR (MH "Child Mortality") OR (MH "Child Passenger Safety") OR (MH "Child Development Disorders, Pervasive+") OR (MH "Child Custody") OR (MH "Child Abuse+") OR (MH "Child Nutritional Physiology+") OR (MH "Child Behavior Checklist") ) OR SO ( child* or pediatric* or paediatric* or adolescent ) |
| S28 | S4 AND S27 |
| S27 | S12 OR S17 OR S23 OR S26 |
| S26 | S24 OR S25 |
| S25 | (MM "Lactose Intolerance") OR "lactose" OR "lactose-free" |
| S24 | (MM "Soy Milk") OR "soy* milk" OR "soy formula" |
| S23 | S18 OR S19 OR S20 OR S21 OR S22 |
| S22 | fermented milk OR lassi OR matson OR viili OR dahi OR kefir OR sour milk |
| S21 | (MH "Cultured Milk Products") |
| S20 | (MH "Yogurt+") OR "yogur" OR "yoghurt" |
| S19 | (MH "Vitamin A") OR "vitamin a" OR "retinol" OR "retinoic" |
| S18 | (MH "Zinc+") OR "zinc" OR (MH "Zinc Sulfate+") OR "cinc" |
| S17 | S13 OR S14 OR S15 OR S16 |
| S16 | (MH "Antidiarrheals") OR "antidiarrh" |
| S15 | loperamide |
| S14 | "racecadotril" or "acetorphan" |
| S13 | smectite or smecta or dismectite |
| S12 | S5 OR S6 OR S7 OR S8 OR S9 OR S10 OR S11 |
| S11 | (MH "Bifidobacterium+") OR "bifidobacterium" |
| S10 | "subtilis" or "clausii" |
| S9 | "thermophilus" |
| S8 | (MH "Enterococcus Faecium+") OR "enterococcus faecium" |
| S7 | "saccharomyces boulardii" |
| S6 | (MH "Lactobacillus+") OR "lactobacill*" OR (MH "Lactobacillus Acidophilus+") OR "reuteri" OR "casei" OR "LGG" OR"rhamnosus" |
| S5 | (MH "Probiotics") OR "probiotic*" OR "prebiotic" OR "symbiotic" |
| S4 | S1 or S2 or S3 or S4 |
| S3 | (MH "Dysentery, Bacillary") OR "dysenter*" |
| S2 | (MH "Gastroenteritis+") OR "gastroenteritis" OR "enteritis" |
| S1 | (MH "Diarrhea") OR "diarrh*" |

LILACS

(tw:((tw:(diarrea)) OR (tw:(gastroenteritis)) OR (tw:(enteritis)) OR (tw:(disentería)) OR (tw:(enfermedad diarreica aguda)) )) OR (tw:(diarr*)) 211.289

(tw:((tw:(probioticos)) OR (tw:(Lactobacillus)) OR (tw:(Saccharomyces)) OR (tw:(Bifidobacterium)) OR (tw:(Enterococcus faecium)) OR (tw:(simbioticos )) )) OR (tw:(probiot*)) OR (tw:(simbiot*)) 167.893

(tw:(Esmectita)) OR (tw:(esmect*)) OR (tw:(diosmectita)) 15

(tw:(racecadotril )) OR (tw:(racecad*)) OR (tw:(acetorphan)) 197

(tw:(loperamida)) OR (tw:(loperamide)) 1.686

(tw:(zinc)) OR (tw:(cinc)) OR (tw:(Zinc*)) 113.797

(tw:(vitamina A)) OR (tw:(retinol)) 196.583

(tw:(vitamina A)) OR (tw:(vitamin A)) OR (tw:(retinol )) 243.221

(tw:(yogur)) OR (tw:(yogurt)) OR (tw:(yogur*)) OR (tw:(kumis)) OR (tw:(kumys)) OR (tw:(leche fermentada)) OR (tw:(fermented milk)) 4.652

(tw:((tw:((tw:(probioticos)) OR (tw:(Lactobacillus)) OR (tw:(Saccharomyces)) OR (tw:(Bifidobacterium)) OR (tw:(Enterococcus faecium)) OR (tw:(simbioticos )) )) OR (tw:(probiot*)) OR (tw:(simbiot*)) )) OR (tw:((tw:(Esmectita)) OR (tw:(esmect*)) OR (tw:(diosmectita)) )) OR (tw:((tw:(racecadotril )) OR (tw:(racecad*)) OR (tw:(acetorphan)) )) OR (tw:((tw:(loperamida)) OR (tw:(loperamide)) )) OR (tw:((tw:(zinc)) OR (tw:(cinc)) OR (tw:(Zinc*)) )) OR (tw:((tw:(vitamina A)) OR (tw:(retinol)) )) OR (tw:((tw:(vitamina A)) OR (tw:(vitamin A)) OR (tw:(retinol )) )) OR (tw:((tw:(yogur)) OR (tw:(yogurt)) OR (tw:(yogur*)) OR (tw:(kumis)) OR (tw:(kumys)) OR (tw:(leche fermentada)) OR (tw:(fermented milk)) )) 519.146

#1 AND #10 9.043

filtro:Lilacs 280

filtro: ensayo clínico 32

**Grey literature Search**

- Clinical Trials.gov: <https://clinicaltrials.gov/>
- World Health Organization WHO International Clinical Trials Registry Platform Search Portal <http://apps.who.int/trialsearch/>
- Controlled-trials.com <http://www.isrctn.com/>
- Web of Science databases (Conference proceedings)
- AAP conferences: [**http://aapexperience.org/**](http://aapexperience.org/)
- ESPGHANP conferences: <http://www.espghan.org/meetings-and-events/annual-meetings/previous-meetings/>

**Citations retrieved per database**

| **Databases** | Number retrieved |
| --- | --- |
| Medline | 1270 |
| Embase | 2078 |
| CENTRAL | 1342 |
| Global Health | 766 |
| CINAHL | 672 |
| LILACS | 30 |
| Web of science | 126 |
| **TOTAL** | **6,284** |

Total

| **Trial registries** | | Number retrieved |
| --- | --- | --- |
| US (Clinicaltrials.gov) | | 247 |
| UN (ISRCTN) | | 83 |
| WHO (ICTRP) | | 305 |
| **TOTAL** | **635** | |

Table A: Excluded Studies and Reasons **(N=235)**

| **First Author** | **Year** | **Title** | **Reason for exclusion** |
| --- | --- | --- | --- |
| Abbaskhanian | 2012 | The effect of fermented yogurt on rotavirus diarrhea in children | Comparing 2 interventions that were categorized as the same intervention (YOG1 vs YOG2) |
| Agarwal | 1995 | Vitamin A administration and preschool child mortality | Different Population: RCT of prophylactic vitamin A to reduce mortality. Not of children with diarrhea. |
| Agarwal | 2002 | Feasibility studies to control acute diarrhoea in children by feeding fermented milk preparations Actimel and Indian Dahi | Comparing 2 interventions that were categorized as the same intervention (YOG1 vs YOG2) |
| Agustina | 2012 | Probiotics, calcium and acute diarrhea: a randomized trial in Indonesian children | Different design (not a RCT) |
| Agustina | 2012 | Randomized trial of probiotics and calcium on diarrhea and respiratory tract infections in Indonesian children | Different Population: Prevention RCT. |
| Alam | 1994 | Enteric protein loss and intestinal permeability changes in children during acute shigellosis and after recovery: Effect of zinc supplementation | Different Population and Outcomes; RCT, children with shigellosis, but given charcoal before being randomised to zinc or no zinc, and outcomes are of intestinal permeability |
| Alam | 2011 | Zinc treatment for 5 or 10 days is equally efficacious in preventing diarrhea in the subsequent 3 months among Bangladeshi children | Different Outcome: Zinc supplementation for Diarrhea prevention, started during diarrheal episode, but both groups received zinc 20mg/d. One groups for 5 days, and the other for 10 days (to measure recurrence of future episodes). |
| Alarcon | 1991 | Clinical trial of home available, mixed diets versus a lactose-free, soy-protein formula for the dietary management of acute childhood diarrhea | Different Intervention: Comparison of lactose-free milk with milk and diet that contained low lactose |
| Alvarez Calatayud | 2009 | Efectividad de racecadotrilo en el tratamiento de la gastroenteritis aguda | Different Design (Observational Study) |
| Amery | 1975 | A multicentre double-blind study in acute diarrhoea comparing loperamide (R 18553) with two common antidiarrhoeal agents and a placebo | Different Population |
| Anonymous | 1996 | Effect of zinc supplementation on diarrhea severity in children | Different design (not a RCT) |
| Anonymous | 2001 | Racecadotril: An antidiarrhoeal suitable for use in infants and young children | Different design (not a RCT) |
| Anonymous | 2001 | Studies confirm efficacy of probiotics for diarrhea | Different design (not a RCT) |
| Anonymous | 2004 | Oral rehydration therapy is the mainstay of treatment for infectious diarrhoea in children | Different design (not a RCT) |
| Anonymous | 2004 | Probiotics for gastrointestinal disorders | Different design (not a RCT) |
| Anonymous | 2007 | Bugs for bugs | Different design (not a RCT) |
| Anonymous | 1994 | [Placebo-controlled, double-blind clinical trial of smectite in acute pediatric diarrhea] | Duplicate of Madkour 1993, #1549 |
| Anonymous | 1994 | Placebo-controlled, double-blind clinical trial of smectite in acute pediatric diarrhea. [German] | Duplicate (in German) of Madkour #1549 |
| Anonymous | 2015 | Probiotic Yogurt for Antibiotic Diarrhea | Different design (not a RCT) |
| Atobe | 2003 | Estudo do efeito do leite fermentado contendo Lactobacillus casei Shirota na microbiota intestinal de crianÃ§as sob terapia antimicrobiana/Study of the effect of fermented milk contend Lactobacillus casei Shirote in the intestinal microflora of children under antimicrobial therapy | Different Population: RCT of probiotics to prevent antibiotic associated diarrhea |
| Bala | 1979 | Evaluation of efficacy and safety of lomotil in acute diarrhoeas in children | Different Intervention: Diphenoxylate |
| Baqui | 2006 | Zinc supplementation and serum zinc during diarrhea | Different design (not a RCT) |
| Baqui | 2002 | Effect of zinc supplementation started during diarrhoea on morbidity and mortality in Bangladeshi children: Community randomised trial | Different Design: Cluster, community RCT. |
| Barry | 1999 | Is soy formula with sucrose better than soy formula with lactose for infants with acute diarrhea? | Different design (not a RCT) |
| Barry | 2002 | Is lactobacillus effective in the treatment of acute infectious diarrhea? | Different design (not a RCT) |
| Baumer | 1992 | [Action of acetorphan, an enkephalinase inhibitor, in acute diarrhea] | Different design (not a RCT) |
| Baumer | 1992 | Effects of acetorphan, an enkephalinase inhibitor, on experimental and acute diarrhoea | Different Population /Adults. Diarrhea was induced with castor oil) |
| Becker | 2006 | Double-blind, randomized evaluation of clinical efficacy and tolerability of an apple pectin-chamomile extract in children with unspecific diarrhea | Different intervention |
| Bequey | 2013 | Comparison of preventive and therapeutic zinc supplementation program effects on diarrhea and febrile illnesses among young children: A randomized trial | Different Population (Diarrhea Prevention RCT) |
| Bergston | 1986 | Symptomatic treatment of acute infectious diarrhoea: loperamide versus placebo in a double-blind trial | Different population: Adults |
| Bhandari | 2008 | Effectiveness of zinc supplementation plus oral rehydration salts compared with oral rehydration salts alone as a treatment for acute diarrhea in a primary care setting: A cluster randomized trial | Different Intervention: Cluster trial; Intervention: education and training of Health care workers in using zinc + ORS |
| Bhan | 1988 | Comparison of a lactose-free cereal-based formula and cow's milk in infants and children with acute gastroenteritis | Different Comparison: (No milk vs milk free formula). |
| Brooks | 2005 | Efficacy of zinc in young infants with acute watery diarrhea | Different Population (Children younger than 6 months) |
| Brown | 1993 | Effect of dietary fiber (soy polysaccharide) on the severity, duration, and nutritional outcome of acute, watery diarrhea in children | Different Intervention: Soy milk vs Soy+Fiber. Milk |
| Burke | 2008 | Journal club. Oral zinc alleviates diarrhea | Different design (not a RCT) |
| Burke | 2011 | Journal club. ORS with zinc/prebiotics limits diarrhea duration | Different design (not a RCT) |
| Cao | 2012 | Clinical study on retention enema of Smecta combined with oral Zinc preparations for treatment of diarrhea in children | Different Intervention (smectite in Enema) |
| Chai | 2009 | Effects of Clostridium butyricum powder with antibiotics on children with acute bacterial diarrhea | Different intervention |
| Chandra | 2002 | Effect of Lactobacillus on the incidence and severity of acute rotavirus diarrhoea in infants. A prospective placebo-controlled double-blind study | Different Population: (Prevention RCT) |
| Chandyo | 2004 | Zinc and childhood infections | Different Population (Diarrhea prevention RCT) |
| Chandyo | 2010 | Two weeks of zinc administration to Nepalese children with pneumonia does not reduce the incidence of pneumonia or diarrhea during the next six months | Different Population (Children with pneumonia) |
| Chang | 2010 | Supplementing iron and zinc: Double blind, randomized evaluation of separate or combined delivery | Different Population (Prevention of diarrhea) |
| Chapoy | 1985 | Treatment of acute infantile diarrhea: controlled trial of *Saccharomyces boulardii* | Different Outcome |
| Chen | 2014 | Influence and curative effect of Saccharomyces boulardii powder on serum interleukin-6 and tumor necrosis factor- alpha levels of children with rotavirus enteritis | Different Intervention (Use Of Antimicrobial interventions in combination owth other interventions) |
| Chowdhury | 2001 | The efficacy of bismuth subsalicylate in the treatment of acute diarrhoea and the prevention of persistent diarrhoea | Different Intervention: Bysmuth |
| Chugh | 1996 | Zinc therapy in acute diarrhea | Different design (not a RCT) (Synopsis of RCT that has been included) |
| Clemente-Yapo | 1993 | [Lactose-free formula versus adapted formula in acute infantile diarrhea] | No access to full text, not possible to determine eligibility. Not possible to contact authors |
| Clin Research Collb Group | 2013 | [A multicenter randomized parallel-controlled study on the efficacy and safety of er xie ting granules in children with acute diarrhea] | Different Intervention |
| Crisinel | 2014 | Demonstration of the effectiveness of zinc in diarrhea of children aged 2 months to 5 years in Lausanne Childhood Hospital | Duplicate of Crisinel 2015, #574 |
| Dagan | 1980 | Lactose-free formulae for infantile diarrhoea | Different Design (Preliminary report of study included Dagan 1984 (#3009)) |
| Dagan | 1980 | Lactose-free formulae for infantile diarrhoea | Different Design |
| Dalby-payne | 2008 | Gastroenteritis in children | Different design (not a RCT) |
| Dalby-Payne | 2002 | Acute gastroenteritis in children | Different design (not a RCT) |
| Darling | 1995 | Improved energy intakes using amylase-digested weaning foods in Tanzanian children with acute diarrhea | Different intervention: Porridge, 3 groups, None of interventions of interests |
| Darmon | 1997 | Zinc in the treatment of diarrhea | Different design (not a RCT) (Sinopsis of RCT that has been included) |
| Datta | 2002 | Zinc or vitamin A reduced diarrhea in young, poor Bangladeshi children | Different design (not a RCT) |
| Deleze | 1983 | Pharmacotherapy of acute diarrhea in children. [French] | Different design (not a RCT) |
| Dinleyici | 2011 | Clinical efficacy of Saccharomyces boulardii or metronidazole in symptomatic children with Blastocystis hominis infection | Different Population: Gastrointestinal symptoms more than 2 weeks. |
| Dom | 1974 | Loperamide (R 18 553), a novel type of antidiarrheal agent. Part 8: Clinical investigation. Use of a flexible dosage schedule in a double-blind comparison of loperamide with diphenoxylate in 614 patients suffering from acute diarrhea | Different Intervention: Diphenoxylate |
| Donnen | 1998 | Randomized placebo-controlled clinical trial of the effect of a single high dose or daily low doses of vitamin A on the morbidity of hospitalized, malnourished children | Different Population: Prevention RCT |
| Donohoe | 1999 | Lactobacillus casei-fortified yoghurt reduces the severity and duration of diarrhoea in healthy children | Different Population: Prevention RCT |
| Dover | 2015 | Rapid cessation of acute diarrhea using a novel solution of bioactive polyphenols: A randomized trial in Nicaraguan children | Different Intervention: Polyphenol |
| Dreverman | 1995 | Loperamide oxide in acute diarrhoea: a double-blind, placebo-controlled trial. The Dutch Diarrhoea Trialists Group | Different Population: 16-75 years. Most of patients: Adults |
| Duan | 2013 | Clinical observation of zinc supplementation in infants with diarrhea | Different Intervention (Co-interventions: antivirals, antimicrobials) |
| Dubey | 2008 | Use of VSL[sharp]3 in the treatment of rotavirus diarrhea in children: preliminary results | Different Outcome (Only Diarrhea of More than 4 days and Treatment failure (but did not provide any definition of the failure). Diarrhea duration was defined in methods but not provided) |
| Dupont | 1992 | Effect of diosmectite on intestinal permeability changes in acute diarrhea: A double-blind placebo-controlled trial | Different Outcome (Smectite vs Placebo, But Different Outcome: Only Permeability outcomes) |
| El-Mougi | 1994 | Is a low-osmolarity ORS solution more efficacious than standard WHO ORS solution? | Different Population: Rehydration RCT |
| Espinoza | 1992 | Rice-based formulas for rapid refeeding of infants with acute diarrhoea. A field trial | Different Interventions: Three interventions lactose free compared, No lactose containing arm |
| Estrada | 2004 | Probiotics in children: An update | Different design (not a RCT) |
| F. Walker | 2011 | Is there a real benefit to zinc and prebiotic fortified ORS in children under the age of 3 years? | Different design (not a RCT) (Sinopsis of RCT) |
| Fang | 2009 | Dose-dependent effect of Lactobacillus rhamnosus on quantitative reduction of faecal rotavirus shedding in children | Different Outcomes: no outomes of interest |
| Faruque | 1999 | Double-blind, randomized, controlled trial of zinc or vitamin A supplementation in young children with acute diarrhoea | Different Population: Zinc results have included children with Zinc Alone and children with Zinc+ vit A, while Vitamin A results have the results mixed of children with Vit A Alone, and children with Vitamin A + zinc |
| Figueroa-Quintanilla | 1993 | A controlled trial of bismuth subsalicylate in infants with acute watery diarrheal disease | Different Intervention (Bysmuth) |
| Fischer Walker | 2008 | Does age affect the response to zinc therapy for diarrhoea in Bangladeshi infants? | Different Design (Secondary Analysis of Fischer-Walker #216) |
| Fischer Walker | 2006 | Zinc supplementation for the treatment of diarrhea in infants in Pakistan | Different Population (Children younger than 6 months) |
| Fischer Walker | 2007 | Zinc during and in convalescence from diarrhea has no demonstrable effect on subsequent morbidity and anthropometric status among infants <6 mo of age | Different Outcomes: Zinc, During Episode + After, and measuring Prevention (incidence and prevalence of diarrhea) |
| Fitzpatrick | 2010 | Effects of acute gastroenteritis | Different design (not a RCT) |
| Franks | 2011 | Probiotics: Probiotics and diarrhea in children | Different design (not a RCT) |
| Freedman | 2014 | Impact of emergency department probiotic treatment of pediatric gastroenteritis: study protocol for the PROGUT (Probiotic Regimen for Outpatient Gastroenteritis Utility of Treatment) randomized controlled trial | RCT Protocol |
| Furmaga | 2012 | The role of lactobacillus rhamnosus GG supplementation on the eradication of pathogenic intestinal flora in infants | Different Outcome (Conference Abstract. Not sure if eligible) |
| Ghisofi | 1987 | [Effects of loperamide on fecal electrolyte excretion in acute diarrhea in infants] | Different outcomes |
| Gibson | 2000 | Zinc supplementation for infants | Different design (not a RCT) |
| Gilbert | 1991 | A study of the effectiveness of smectite versus placebo or loperamide in acute infantile diarrhea. [French] | Duplicate of Gilbert 1991, #914 |
| Gilbert | 1991 | [The efficacy of smectite in acute infantile diarrhea, compared to a placebo and loperamide] | Duplicate of Gilbert 1991, #914 |
| Girola | 1995 | Efficacy of probiotic preparation with living, freere-dried lactic acid bacteria and yeast on child diarrhoea. [Italian] | Different Population: Mixed gastroenteritis + Antibiotic-Associated Diarrhea, imposible to extract info for each group |
| Goepp | 1997 | Comparison of two regimens of feeding and oral electrolyte solutions in infants with diarrhea | Different Intervention: Rice-based feeding regime + ORS in different compositions |
| Grange | 1994 | Evaluation of cassava-salt suspension in the management of acute diarrhoea in infants and children | Different Intervention: Soy vs Maize diet |
| Grange | 1994 | Evaluation of a maize-cowpea-palm oil diet for the dietary management of Nigerian children with acute, watery diarrhea | Different Population: Rehydration Trial |
| Grenov | 2016 | The effect of two probiotic strains BB-12 and LGG on diarrhea in children with severe acute malnutrition in Uganda Journal of Pediatric Gastroenterology and Nutrition | Different Population (children with severe malnutrition) |
| Guarino | 2001 | Smectite in the treatment of acute diarrhea: a nationwide randomized controlled study of the Italian Society of Pediatric Gastroenterology and Hepatology (SIGEP) in collaboration with primary care pediatricians. SIGEP Study Group for Smectite in Acute Diarrhea | Duplicate of Guarino 2001, #989 |
| Guo | 2009 | Effect of medilac-vita combined with ribavirin and smecta in treatment of infantile rotavirus enteritis | Different Intervention: Use of Antimicrobial interventions in combination owth other interventions |
| Gutierrez-Castrellon | 2016 | Randomized, double blind, placebo controlled, clinical trial on the safety, efficacy and pharmacoeconomic analysis of racecadotril in children with acute diarrhea. | No access to full text, not possible to determine eligibility. Authors contacted, no response to request of information. Possibly a duplicate of the next reference (Gutierrez 2015) |
| Gutiérrez-Castrellón | 2015 | Ensayo clínico aleatorizado y análisis farmaco económico del impacto de racecadotrilo (Hidrasec®) como coadyuvante en el tratamiento de la gastroenteritis aguda sobre la reducción de los gastos hospitalarios relacionados en lactantes menores de 24 meses en México. Gac Med Mex. 2014 (in press). | No access to full text, not possible to determine eligibility. Authors contacted, no response to request of information. Possibly a duplicate of the previous reference (Gutierrez 2016) |
| Gutierrez-Castrellon | 2014 | Diarrhea in preschool children and lactobacillus reuteri: A randomized controlled trial | Different Population: Prevention RCT |
| Romer | 1991 | Realimentation of dehydrated children with acute diarrhea: Comparison of cow's milk to a chicken-based formula | Different Intervention |
| Habib | 2010 | Effect of zinc in tablet and suspension formulations in the treatment of acute diarrhoea among young children in an emergency setting of earthquake affected region of Pakistan | Different Intervention: (Implementation of a package for Diarrhea treatment in Community and Physicians) |
| Hanauer | 2007 | Randomized, double-blind, placebo-controlled clinical trial of loperamide plus simethicone versus loperamide alone and simethicone alone in the treatment of acute diarrhea with gas-related abdominal discomfort | Different Population: Adults |
| Hendrickse | 1984 | Loperamide in acute diarrhoea in childhood: Results of a double blind, placebo controlled multicentre clinical trial | Duplicate of Anonymous 1984, #165 |
| Henker | 2008 | Acute diarrhoea in infants and small children. Successful adjuvant therapy with the probiotic Mutaflor. [German] | Duplicate of Henker 2007, #1077 |
| Heredia-Diaz | 1979 | [Evaluation of the safety and effectiveness of WHR-1142A in the treatment of non-specific acute diarrhea] | Different Intervention. |
| Hidayat | 1998 | The effect of zinc supplementation in children under three years of age with acute diarrhea in Indonesia Medical Journal of Indonesia | Different population (Patients were enrolled more than once) |
| Hoghton | 1996 | Effects of immediate modified feeding on infantile gastroenteritis | Different Intervention: immediate modified feeding vs Fasting |
| Hossain | 1998 | Single dose vitamin A treatment in acute shigellosis in Bangladeshi children: Randomised double blind controlled trial | Different Population: Only Children with Shigellosis |
| Hoy-Schulz | 2016 | Safety and acceptability of Lactobacillus reuteri DSM 17938 and *Bifidobacterium longum* subspecies infantis 35624 in Bangladeshi infants: A phase I randomized clinical trial | Different population (Healthy children) |
| Hu | 2014 | Curative effect of Montmorillonite Powder combined with Bifidobacterium Tetravaccine Tablet in treatment of infantile diarrhea and detection of related factors | Different Intervention. |
| Huntley | 2002 | Probiotics show promise for prevention and treatment of diarrhoea | Different Intervention: Prevention RCT |
| Jasinski | 2002 | Efficacy of Lactobacillus GG in oral rehydration solution | Different Intervention (Diluted in ORS) |
| Jiang | 2015 | Influence of Saccharomyces boulardii powder on intestinal microecology of children with rotavirus enteritis | Different Intervention (Co-interventions: antivirals, antimicrobials) |
| Jiang | 2015 | Influence of Saccharomyces boulardii powder on intestinal microecology of children with rotavirus enteritis | Different Intervention (Co-interventions: antivirals, antimicrobials) |
| Jiao | 2015 | Effect of zinc supplementation at different dosage on change of serum zinc level in children with rotavirus enteritis and low zinc level | Different Intervention: Use Of Antimicrobial interventions in combination owth other interventions |
| Jin | 2016 | Probiotics combined with montmorillonite for treatment of diarrhea in children: Effect on clinical symptoms | Different intervention |
| Jin | 2013 | Significance of zinc supplementation in infants with rotavirus enteritis. [Chinese] | Different Intervention (Co-interventions: antivirals, antimicrobials) |
| Jin | 2013 | Significance of zinc supplementation in infants with rotavirus enteritis. [Chinese] | Different Intervention (Co-interventions: antivirals, antimicrobials) |
| Kaila | 1992 | Enhancement of the circulating antibody secreting cell response in human diarrhea by a human Lactobacillus strain | Comparing 2 interventions that were categorized as the same intervention (LGG1 vs LGG2) |
| Kaila | 1994 | Immune responses evoked by cow milk products in health and during rotavirus diarrhea | Different Outcome (Immune responses) |
| Kaila | 1995 | Viable versus inactivated lactobacillus strain GG in acute rotavirus diarrhoea | Comparing 2 interventions that were categorized as the same intervention (YOG1 vs YOG2) |
| Kaila | 1998 | Fecal recovery of a human Lactobacillus strain (ATCC 53103) during dietary therapy of rotavirus diarrhea in infants | Different Outcome: Only fecal bacteria recovery |
| Karan | 1976 | Assessment of safety of Lomotil in infants | Different intervention |
| Kowalska-Duplaga, | 2004 | Efficacy of Trilac in the treatment of acute diarrhoea in infants and young children - A multicentre, randomized, double-blind placebo-controlled study. [Polish] | No access to full text, not possible to determine eligibility. Not possible to contact authors |
| Kolader | 2013 | An oral preparation of Lactobacillus acidophilus for the treatment of uncomplicated acute watery diarrhoea in Vietnamese children: Study protocol for a multicentre, randomised, placebo-controlled trial | Different Design (RCT not finished) |
| Kukuruzovic | 2002 | Milk formulas in acute gastroenteritis and malnutrition: A randomized trial | Different Population: : Mixed: diarrhea and Malnutrition without Diarrhea, info not separated |
| Kwon | 2017 | Effect of Low Lactose Special Formula (MF-1) for the Treatment of Acute Diarrhea in Infants. | Different outcome |
| L. Hohenauer | 1983 | [Dietary treatment of acute gastroenteritis in infants] | Different Intervention: Lactose free vs Low Lactose, not compared with Lactose Containing |
| Lachaux | 1986 | Acute infantile diarrhoea. Role of treatment with smectite as com- plement to rehydration. Randomised double-blind study. Int Rev Pediatr 1986; 163: 29–31. | No access to full text, not possible to determine eligibility. Not possible to contact authors |
| Laudano | 1974 | Comparative assessment of two antidiarrhoeal preparations and placebo in the treatment of acute, non-specific enterocholitis | Different Interventions: Neomycin |
| Lazzerini | 2011 | Oral zinc for treating diarrhoea in children | Different design (not a RCT) |
| Leber | 1988 | A new suspension form of smectite (Liquid 'Diasorb') for the treatment of acute diarrhoea: a randomized comparative study | Different Population (adults) |
| Leber | 1988 | A new suspension form of smectite (Liquid 'Diasorb') for the treatment of acute diarrhoea: a randomized comparative study | Different Population |
| Lee | 1968 | Comparative trial of Lomotil-with-neomycin and kaolin-and-morphine mixture in general practice | Different Intervention (lomotil-diphenoxylate + neomicina) |
| Lee | 2001 | Oral bacterial therapy promotes recovery from acute diarrhea in children | No access to full text, not possible to determine eligibility. Not possible to contact authors |
| Laxmi | 2006 | Effect of vitamin 'A' supplementation on vitamin A status of children suffering from diarrhoea | No access to full text, not possible to determine eligibility. Not possible to contact authors |
| Li | 2008 | Effects of treating infantile rotavirus enteritis with potassium sodium dehydroandroan drographolide succinate and cemitidine | Different intervention |
| Li | 2014 | Clinical research of using Saccharomyces boulardii to prevent secondary diarrhea in hospitalized neonates | Different population (prevention diarrhea in neonates) |
| Li | 2009 | Effect of si-lian-kang on rotavirus enteritis of infants and young children | Different intervention |
| Li | 3850 | Effect of probiotics treatment in children with diarrheal disease | Different Intervention: Use of Antimicrobial interventions in combination owth other interventions |
| Li | 2014 | S. boulardii sachets and Clostridium butyricum treating rotavirus viral enteritis in infants: a comparison of efficacy and safety | Different Intervention: Use Of Antimicrobial interventions in combination owth other interventions |
| Li | 2011 | Effect of Combined Clostridium Butyricum and Bifidobacterium Capsules on various type of diarrhea in children | Different Intervention (Co-interventions: antivirals, antimicrobials) |
| Li | 2011 | Effect of Combined Clostridium Butyricum and Bifidobacterium Capsules on various type of diarrhea in children | Different Intervention (Co-interventions: antivirals, antimicrobials) |
| Lifschitz | 1985 | Absorption and tolerance of lactose in infants recovering from severe diarrhea | Different Outcomes (weigth gain, breath H2, caloric intake) |
| Limaye | Excluded | Comparison of Lomotil with Pectokab in furazolidone-treated infective diarrhoea | Different intervention: Diphenoxylate |
| Lin | 2010 | Effect of zinc gluconate tablets on serum zinc and diarrhea of children with rotavirus enteritis | Different Intervention: Use of Antimicrobial interventions in combination owth other interventions |
| Liu | 2010 | Observation of curative effect with oral zinc treatment of 80 cases of infants with acute diarrhea | Different Intervention (Co-interventions: antivirals, antimicrobials) |
| Long | 2007 | Vitamin A supplementation has divergent effects on norovirus infections and clinical symptoms among Mexican children | Different Population: patients without diarrhea |
| Long | 2007 | The comparative impact of iron, the B-complex vitamins, vitamins C and E, and selenium on diarrheal pathogen outcomes relative to the impact produced by vitamin A and zinc | Different Population: patients without diarrhea, outcomes was prevalence of diarrhea |
| Lonnermark | 2015 | Effects of probiotic intake and gender on nontyphoid salmonella infection | Different Population: Salmonellosis and Adults |
| Losonsky | 1992 | Diarrhea and gastroenteritis | Different design (not a RCT) |
| Lou | 2013 | A multicentre randomized study on the efficacy of Er Xie Ting in children with acute diarrhea | Different Intervention |
| Macedo-Prietsch, 2000 | 1999 | Comparative study of a soy-based lactose-free formula, versus a lactose- containing formula, during an acute phase of diarrheal illness in infants [Estudo comparativo de uma fórmula não láctea à base de soja, sem lactose, versus fórmula láctea com lactose, durante a fase aguda da doença diarréica em lactentes] | Different Outcome |
| Malik | 2013 | Short-course prophylactic zinc supplementation for diarrhea morbidity in infants of 6 to 11 months | Different Population: healthy children with no diarrhea, incidence of diarrhea |
| Manish | 2010 | Evaluation of the efficacy and safety of probiotic formulation with zinc enriched yeast in children with acute diarrhea | Different design (not a RCT) |
| Maragkoudaki, | 2016 | Efficacy of an ORS enriched with L. reuteri DSM 17938 and zinc in infants with acute gastroenteritis: A double blind, placebo controlled trial | Different intervention |
| Marchetti | 2017 | Oral rehydration and the use of ondansetron and of domperidone in acute gastroenteritis: An Italian multicentric trial. [Italian] Medico e Bambino | Different intervention & Different population |
| Maragkoudaki | 2016 | Efficacy of an ORS enriched with L. reuteri DSM 17938 and zinc in infants with acute gastroenteritis: A double blind, placebo controlled trial | Different Intervention: Intervention diluted in ORS |
| Margolis | 1990 | Effects of unrestricted diet on mild infantile diarrhea. A practice-based study | Different Intervention (Compared Lactose containing and lactose free children mixed with Usual management. Not possibl to have effect for Lactose free) |
| Matheson | 2000 | Racecadotril | Different design (not a RCT) |
| Maulen-Radovan | 1994 | Comparison of a rice-based, mixed diet versus a lactose-free, soy-protein isolate formula for young children with acute diarrhea | Different intervention (Mixed Diet intervention) |
| Mazumder | 2010 | Effectiveness of zinc supplementation plus oral rehydration salts for diarrhoea in infants aged less than 6 months in Haryana state, India. [French] | Different Design (Secondary analysis from another trial that was excluded: Bhandari 2008) |
| McFarland | 2010 | Probiotics and diarrhea | Different design (not a RCT) |
| Meadows-Oliver | 2009 | Use of probiotics in pediatrics | Different design (not a RCT) |
| Mehta | 2012 | A comparative study of racecadotril and single dose octreotide as an anti-secretory agent in acute infective diarrhoea | Different Population: patiens >15 years |
| Menor | 2016 | Clinical evaluation of a synbiotic for children between 6 months and 2 years with acute viral diarrhea | No access to full text, Not possible to determine eligibility. Not possible to contact authors |
| Mita | 1995 | Hyperimmune cow colostrum reduces diarrhoea due to rotavirus: A double-blind, controlled clinical trial | Different Intervention: hyperimmune cow´s colostrum was the intervention |
| Mouzaki | 2014 | Lactose avoidance shortens symptom duration for young children with acute diarrhoea | Different design (not a RCT) |
| Nabulsi | 2015 | Lactose-free milk for infants with acute gastroenteritis in a developing country: Study protocol for a randomized controlled trial | Different Design (RCT not finished) |
| Naheed | 2009 | Zinc therapy for diarrhoea improves growth among bangladeshi infants 6 to 11 months of age | Different Design (it was a secondary analysis from a previous trial, outcomes were: growth) |
| Narayanappa | 2009 | A randomized double-blind placebo controlled trial to evaluate the efficacy and safety of bifilac in children with acute rotaviral diarrhea | Duplicate of Narayanappa 2008, #1779 |
| Nasir | 2011 | Bifidogenicity of galacto-oligosaccharides in diarrhea management of acute malnourished infants and young children | Different intervention: Special Formula for the management of Severe Malnutrition |
| Neu | 2005 | Probiotics: protecting the intestinal ecosystem? | Different design (not a RCT) |
| Ninger | 2008 | Zinc reduces diarrhea in children | Different design (not a RCT) (Sinopsis of RCT that has been included) |
| Nizami | 1996 | Efficacy of traditional rice-lentil-yogurt diet, lactose free milk protein-based formula and soy protein formula in management of secondary lactose intolerance with acute childhood diarrhoea. | Comparing 2 interventions that were categorized as the same intervention (Lactose-free formula vs lactose-free formula) |
| Noah | 1979 | Soy-based formula for infantile diarrhoea a controlled trial | Different design (not a RCT) |
| Noguera | 2014 | Resolution of acute gastroenteritis symptoms in children and adults treated with a novel polyphenol-based prebiotic | Different Population (study in adults, only a few children under 14, but the results can´t be differentiated) |
| Osman | 1992 | An adsorbent! Role in the management of acute diar- rhea in infants and children. Ain Shams Med J 1992; 13: 10, 11 and 12665–76. | No access to full text, not possible to determine eligibility. Not possible to contact authors |
| Palma | 1997 | Acute diarrhea: stool water loss in hospitalized infants and its correlation with etiologic agents and lactose content in the diet. [Portuguese] | Different Outcome (Diarrhea Duration provided by Etiology Gropus (EPEC), not per arm. No Additional outcomes of interest) |
| Passariello | 2011 | Efficacy of a new hypotonic oral rehydration solution containing zinc and prebiotics in the treatment of childhood acute diarrhea: A randomized controlled trial | Different intervention: Zinc in Hypotonic ORS, compared with a different ORS Solution |
| Patel | 2005 | Therapeutic evaluation of zinc and copper supplementation in acute diarrhea in children: Double blind randomized trial | Different outcomes (No outcome of interest) |
| Patel | 2013 | Therapeutic zinc and copper supplementation in acute diarrhea does not influence short-term morbidity and growth: Double-blind randomized controlled trial | Different Design (Secondary analysis of #1930 (Patel 2009)) |
| Patel | 2010 | Influence of zinc supplementation in acute diarrhea differs by the isolated organism | Different Design (Secondary analysis of #1930 (Patel 2009)) |
| Patman | 2014 | Diarrhoea: Lactobacillus reuteri reduces episodes of diarrhoea in healthy children | Different design (not a RCT) |
| Penny | 1995 | Zinc in the management of diarrhea in young children | Different design (not a RCT) |
| Phavichitr | 2012 | Cost-effectiveness of probiotic combination (Lactobacillus acidophilus plus Bifidobacterium bifidum) in treating acute childhood diarrhea in hospitalized patients | Duplicate of Pavichitr 2013, # 1987 |
| Pinto | 2016 | Lactobacillus acidophilus Mixture in Treatment of Children Hospitalized With Acute Diarrhea Clinical Pediatrics | Different design (cohort) |
| Pleea | 2016 | Xyloglucan for the treatment of acute gastroenteritis in children: Results of a randomized, controlled, clinical trial. | Different intervention |
| Pu | 2012 | Curative effects of infantile tuina combined with smecta retention enema on rotavirus enteritis | Different intervention |
| Rahman | 2005 | Effects of zinc supplementation as adjunct therapy on the systemic immune responses in shigellosis | Different Outcomes: no clinical outcomes |
| Rajah | 1988 | The effect of feeding four different formulae on stool weights in prolonged dehydrating infantile gastroenteritis | Different outcomes: stool weigth |
| Raqib | 2004 | Effect of zinc supplementation on immune and inflammatory responses in pediatric patients with shigellosis | Different outcomes: linfocites, interleukins, antibody titers |
| Rautanen | 1998 | Management of acute diarrhoea with low osmolarity oral rehydration solutions and Lactobacillus strain GG | Different Intervention (RCT of Hydration Solutions + Randomized to receive LGG vs placebo, but information for Placebo was not described, only early vs late LGG, and these patients received Hydration with Low and standard osmolarity, which makes not possible to obtain the effect of LGG vs No treatment/placebo) |
| Raza | 1995 | Lactobacillus GG in acute diarrhea | Duplicate of Raza 1995, #2102 |
| Rhoads | 1994 | Earth, wind, and fiber: is there a drug to treat acute diarrhea? | Different design (not a RCT) (Sinopsis of RCT) |
| Riera | 2011 | Zinco oral para o tratamento de diarreia em crianÃ§as/Zinc mouth for the treatment of diarrhea in children | Different design (not a RCT) |
| Rio | 2004 | Influence of nutritional status on the effectiveness of a dietary supplement of live lactobacillus to prevent and cure diarrhoea in children. [Spanish] | Different Population (Diarrhea prevention RCT) |
| Rollins | 2000 | Vitamin A supplementation of South African children with severe diarrhea: Optimum timing for improving biochemical and clinical recovery and subsequent vitamin A status | Different population (Population Mixed: Mixed Chronic and Acute Diarrhea) |
| Romer | 1991 | Realimentation of dehydrated children with acute diarrhea: Comparison of cow's milk to a chicken-based formula | Different Intervention |
| Roy | 1992 | Impact of zinc supplementation on intestinal permeability in Bangladeshi children with acute diarrhoea and persistent diarrhoea syndrome | Different Outcome: lactulose and manitol excretion |
| Roy | 2008 | Zinc supplementation in the management of shigellosis in malnourished children in Bangladesh | Different Population: Only Children with Shigellosis |
| Ruz | 1995 | Fecal excretion of endogenous zinc during oral rehydration therapy for acute diarrhea: Nutritional implications | Different population and Outcomes |
| S. McRae | 2009 | Oral zinc for treating diarrhoea in children in the developing world | Different design (not a RCT) |
| Salazar-Lindo | 2001 | Racecadotril was effective for severe watery diarrhoea in children | Different design (not a RCT) (Sinopsis of RCT that has been included) |
| Salvatore | 2007 | Probiotics and zinc in acute infectious gastroenteritis in children: are they effective? | Different design (not a RCT) |
| Sandhu | 1997 | A multicentre study on behalf of the European Society of Paediatric Gastroenterology and Nutrition Working Group on Acute Diarrhoea. Early feeding in childhood gastroenteritis | Different Intervention (Early vs Late fedding, Not Lactose Avoidance) |
| Santosham | 1990 | A comparison of rice-based oral rehydration solution and 'early feeding' for the treatment of acute diarrhea in infants | Diffeent Intervention: Soy, compared with Different rice preparations and Rice formula |
| Santosham | 1991 | Role of a soy-based lactose-free formula in the outpatient management of diarrhea | Different Intervention: Early refeeding vs delayed refeeding |
| Santosham | 1985 | Role of soy-based, lactose-free formula during treatment of acute diarrhea | Different Intervention: Soy Formula compared to 24 hours fasting |
| Savas-Erdeve | 2009 | Efficacy and safety of Saccharomyces boulardii in amebiasis-associated diarrhea in children | Different Population (Amebiasis) |
| Savitha | 2006 | Racecadotrial – a novel drug for treatment of acute watery diarrhoea in Indian children. Karnataka Pedicon 2005 – Conference Abstracts. Pediatric Oncall [serial online]; 2006 [cited 01 January 2006]; 3. | No access to full text, Not possible to determine eligibility. Authors contacted, no response to request of information |
| Sharif | 2017 | The Role of Probiotics in the Treatment of Dysentery: a Randomized Double-Blind Clinical Trial Probiotics and Antimicrobial Proteins | Different population (dysentery) |
| Sheen | 1995 | Short report: A placebo-controlled study of Lactobacillus GG colonization in one-to-three-year-old Peruvian children | Different intervention and Outcome: Zn for 10 days T all patients, then randomized for additional supplementation or placebo to evaluate effects on Blood cells count and Immune responses |
| Sugita | 1994 | Efficacy of Lactobacillus preparation bioloactis powder in children with rotavirus enteritis. | No access to full text, not possible to determine eligibility. Authors contacted, no response to request of information |
| Sheikh | 2010 | Zinc influences innate immune responses in children with enterotoxigenic Escherichia coli-induced diarrhea | Different intervention and Outcome: Zn for 10 days T all patients, then randomized for additional supplementation or placebo to evaluate effects on Blood cells count and Immune responses |
| Sudarmo | 2003 | Management of infant diarrhea with high-lactose probiotic-containing formula | Different Intervention (Compared High lactose formula + Probiotic vs Control. Not clear about what was High-lactose, how was the concentration, and not clear whether the Control received either High, Normal lactose or Low-lactose) |
| Sun | 2012 | Efficacy of ganciclovir and Smecta at treating 56 infants with rotavirus enteritis | Different intervention |
| Szymanski | 2006 | Colonisation of the gastrointestinal tract by probiotic L. rhamnosus strains in acute diarrhoea in children | Different Outcome (Only colonization outcome) |
| Thibault | 2004 | Effects of long-term consumption of a fermented infant formula (with Bifidobacterium breve c50 and Streptococcus thermophilus 065) on acute diarrhea in healthy infants | Different Population (Prevention RCT) |
| Tian | 2012 | Observation on curative effect of Medilac-Vita and Smecta in treatment of infant rotavirus enteritis | Different Intervention: Use Of Antimicrobial interventions in combination owth other interventions |
| Tikhomirova | 2009 | Clinical and immunological efficiency of anaferon (pediatric formulation) in calicivirus infection in children | Different Intervention: RCT about IFN for Calicivirus |
| Tran | 2015 | Zinc-Fortified Oral Rehydration Solution Improved Intestinal Permeability and Small Intestinal Mucosal Recovery | Different Intervention (Diluted in ORS) |
| Tran | 2015 | Zinc-Fortified Oral Rehydration Solution Improved Intestinal Permeability and Small Intestinal Mucosal Recovery | Different Intervention: ORS + Zinc enriched |
| Urbanska | 2016 | Systematic review with meta-analysis: Lactobacillus reuteri DSM 17938 for diarrhoeal diseases in children Alimentary Pharmacology & Therapeutics | Different design |
| Valery | 2005 | Zinc and vitamin A supplementation in Australian indigenous children with acute diarrhoea: A randomised controlled trial | Different Population: Results presented mixed; Zinc group included children with Zinc alone and with Zinc+Vitamin A. It was not possible to have the information separated for zinc, Vit A and Combination |
| Van den Eynden | 1995 | New approaches to the treatment of patients with acute, nonspecific diarrhea: A comparison of the effects of loperamide and loperamide oxide | Different Population: Adults |
| Vandenplas | 2012 | Cost/benefit of synbiotics in acute infectious gastroenteritis: Spend to save | Different Design (Secondary Analysis of Vandenplas 2011, #2660) |
| Vanderhoof | 1997 | Use of soy fiber in acute diarrhea in infants and toddlers | Different Intervention: Soy milk vs Soy Fiber Milk |
| Vesikari | 1985 | A comparative trial of cholestyramine and loperamide for acute diarrhoea in infants treated as outpatients | Different interventions Cholestiramine |
| Wadhwa | 2011 | ORS containing zinc does not reduce duration or stool volume of acute diarrhea in hospitalized children | Different Intervention: ORS + Zinc enriched |
| Wang | 2012 | Analysis on the effect of Smecta and synbiotics probiotics on treatment of pediatric non-infectious diarrhea | Different Intervention (Co-interventions: antivirals, antimicrobials) |
| Wang | 2008 | The comparison of the therapeutic effects smecta and siliankang in treatment of rotavirus enteritis in young children | Different Intervention (Co-interventions: antivirals, antimicrobials) |
| Wemmer | 1977 | [Nutrition in infant enteritis] | Different Intervention (Unclear the intervention, although they seemed to be comapring Low lactose vs Lactose full containng. Not clear inclusioncriteria, in terms of Diarrhea) |
| Willumsen | 1997 | Dietary management of acute diarrhoea in children: effect of fermented and amylase-digested weaning foods on intestinal permeability | Different outcome: permeability. |
| Xie | 2013 | Therapeutic effect of probiotics and oral IgY as supplementary drugs in the treatment of pediatric rotavirus enteritis: A comparative study. [Chinese] | Different Intervention (Co-interventions: antivirals, antimicrobials) |
| Xu | 2009 | Efficiency of lactose-free formula feeding as an adjunctive therapy in infants with acute diarrhea. [Chinese] | Different Intervention (Co-interventions: antivirals, antimicrobials) |
| Xu | 2016 | A double-blinded randomized trial on growth and feeding tolerance with Saccharomyces boulardii CNCM I-745 in formula-fed preterm infants Jornal de Pediatria | Different population (Healthy children) |
| Yu | 2011 | Clinical study on racecadotril combined with montmorillonite powder in treatment with infantile rotavirus enteritis | Different Intervention (Racecadotril mixed with montmorillonite) |
| Yuan | 2014 | Influence and curative effect of Saccharomyces boulardii Sachets combined with Smectite Powder on cellular immune function of children with rotavirus enteritis | Different Intervention (Co-interventions: antivirals, antimicrobials) |
| Zaman | 2014 | Antisecretory factor effectively and safely stops childhood diarrhoea: A placebo-controlled, randomised study | Different intervention. Egg Yolk |
| Zhou | 2014 | Clinical efficacy of zinc gluconate and reduning injection in the treatment of infantile autumn diarrhea | Different Intervention (Co-interventions: antivirals, antimicrobials) |

# Table B: Included Studies (N=174)

** Full reference list in Appendix 4*

| **Author/year** | **Country** | **Age (months)** | **Setting** | **Interventions (n per arm)** | **Dose/frequency** | **Duration of intervention** | **Funding** |
| --- | --- | --- | --- | --- | --- | --- | --- |
| Agustina 2007^1^ | Indonesia | 3-60 | Inpatients | 1. Low lactose formula with precooked rice + *Lactobacillus rhamnosus* LMG P-22799 + Prebiotic (inulin) + dietary fiber (soya polysaccharides) + Zinc and Iron (+0.4 and +0.6 mg/100mL, respectively) n=30 2. Low lactose infant formula with precooked rice  n=28 | Both: Formula ad libitum on demand (up to 140 mL/kg/day) | 7 days | Private |
| Al-Sonboli 2003^2^ | Brazil | 3-60 | inpatients | 1. Zinc n= 37 2. vitamin C (Redoxon, flavourless), Roche Consumer Health) n=37 | 1. 3-6 months=22.5 mg/day 7-60 months=45mg/day QD 2. 3-6 months 250 mg/day 7-60 months=500mg | 5 days | Not specified |
| Allen 1994^3^ | Canada | 2-12 | Inpatients | 1. Soy-based formula, Isomil (Ross Laboratories, Montreal, Canada n=39 2. Lactose-containing formula, SMA (Wyeth Limited, Toronto, Canada) n=34 | Both ad libitum during the first 24 h at a time determined by the attending pediatrician | 14 days | Private |
| Anderson 1984^4^ | Mexico | 3-120 | Recruited at ED | 1. Loperamide n=26 2. Placebo n=30 | 1. <20kg: 2mg  >20kg: 4mg/day 2. NA | NR | Not specified |
| Anonymous 1984^5^ | U.K. | 3-36 | Inpatients | 1**.** Loperamide syrup n=102 2. Loperamide n=101 3. Placebo syrup n=100 | 1. 0.8 mg/kg/day 2. 0,4 mg/kg/day 3. NA | Until diarrhea resolved or for 7 days | Not specified |
| Armitstead 1989^6^ | UK | 1-9 | outpatients | 1. Quarter-strenght adapted milk formula (SMA-Wyeth or Premium-Cow & Gate), diluted with *GEM. n=22 2. Alfaré Formula n=24 *GEM Glucose Electrolite Mixture 3. Full-strength adapted milk. n=22 | 1. Increased in quarter-strength steps at 24h intervals to full-strenght feedings.  2. Half-strength in GEM for 24h, followed by 4 days of full-strength Alfaré, then was returned to the usual formula.  3. NA | 14 days | Private |
| Awasthi 2006^7^ | Brazil, Ethiopia, Egypt, India, Philippines | 2-59 | outpatients | 1. Zinc tablets (Nutriset; manufactured by Rodael, Malaunay, France) + education to promote adherence n=1010 2. Standard (only ORS) n=992 | 1. 10-20 mg/day  QD to BID 2. NA | 14 days | Not for profit |
| Bahl 2002^8^ | India | 6-35 | outpatients | 1. Zinc gluconate syrup (GK Pharma ApS, Køge, Denmark) n=404 2**.** Zinc+ORS (mixed) n=402 3. Placebo n=401 | **1.** *Children 12 to 35 months: 30mg/day (12 ml) *Children 6 to 11 months: 15 mg/day (15 ml) **2.** 40mg mixed in 1 liter of water 40mg zinc gluconate was mixed in 1 liter of solution10mL/kg 3. NA | 14 days | Not for profit |
| Basu 2007^9^ | India | NR | Inpatients | *1. Lactobacillus rhamnosus -GG* (LGG)  n=330 2. No treatment (Standard) n=332 | 1. 60 million cells dissolved in 100 mL of ORS, BID  2. NA | 7 days or till diarrhea ceased | Not specified |
| Basu 2009^10^ | India | NR | Inpatients | 1**.** ORS+ *Lactobacillus rhamnosus-* LGG  n=196 2**.** ORS + *Lactobacillus rhamnosus- LGG*  n=196 3. No treatment (Standard) n=196 | **1.** powder 10 exp10 CFU in 100mL of ORS BID **2.** powder 10 exp12 CFU dissolved in 100mL of ORS BID 3. NA | 7 days or until diarrhea ceased. | Not specified |
| Bhandari 1997^11^ | India | 12-60 | Outpatients | 1. Vitamin A capsules n=451 2. Placebo n=444 | 1. 60 mg once 2. NA | Not clear. It seems it was one day | Not for profit |
| Bhatnagar 1998^12^ | India | 4-48 | Recruited at ED | 1. Yogurt: 67 cal/100 Combination of *Streptococcus thermophilus* and *Lactobacillus bulgaricus*  n=47 2. Regular formula: 67 cal/100 ml (Lactogen-2; Nestle India Ltd., New Delhi, India) n=49 | 1. probiotics added to the to the formula milk 90 gm of a pre-prepared standard 2. No more information | 72 hours or until recovery | Not for profit |
| Bhatnagar 2004^13^ | India | 3-36 | Recruited at ED | 1. Zinc + vitamin B complex  n=143 2. Placebo + vitamin B complex n=144 | 1. 1mg/ml of elemental zinc *<12 months=15ml/d divided in 3 doses  *>12 months=30ml/day divided in 3 doses zinc sulfatate Both: Placebo and zinc  mixed vitamin B complex (5 mL contained: B1, 2.5 mg; B2, 2.5 mg; B6, 1.0 mg; B12, 3#g; C, 50 mg, D-panthenol, 12.5 mg niacinamide, 25 mg | 14 days | Not for profit |
| Billoo 2006^14^ | Pakistan | 2-144 | outpatients | 1. Probiotic *Saccharomyces boulardii* n=50 2. No treatment (Standard) n=50 | 1. 250 mg bid 2. NA | 5 days | Private |
| Boran 2006^15^ | Turkey | 6-60 | outpatients | 1. Zinc syrup  n=150 2. No treatment (Standard) n=130 | 1. 6 to 12 months:15 mg/day 12-60 months: 30mg/day QD syrup zinc sulphate 2. NA | 14 days | Private |
| Boudraa 2001^16^ | Algeria | 3-24 | Recruited at ED | 1. Yogurt (Fermented from Formula Adding: *Streptococcus thermophylus + Lactobacillus Bulgaricus* (YALACTA).  Co-intervention: vegetables with fat added in the form of butter and cereals (Sinlac, Nestlé, Vevey, Switzerland) n=56 2. Regular formula (ENAP AL-SOP AD, Nestlé, Courbevoie, France) Co-intervention: vegetables with fat added in the form of butter and cereals (Sinlac, Nestlé, Vevey, Switzerland) n=56 | 1. 180ml/kg/day in 6 meals 2. NA | NR | Not specified |
| Boulloche 1994^17^ | France | 1-48 | Inpatients | 1. *Lactobacillus acidophillus* n=38 2. Loperamide n=32 3. Placebo n=33 | 1. SACHET 3First, 1 BID 2. TID 10drop/k/day 3. NA | 5 days | Not specified |
| Bowie 1995^18^ | South Africa | 3-18 | Recruited at ED | 1. Loperamide n=100 2. Placebo n=100 | 0.8mg/kg/day TID | NR | Private |
| Brown 1991^19^ | Peru | 3-24 | Inpatients | **1. Group HM:** lactose hydrolyzed milk (Lactaid, sugar-Lo, Pleasantville, NJ, U.S.A) n= 30 2. **Group N-M:** mixture wheat noodles + regular formula with lactose. N=29 3. **Group N-HM:** mixture wheat noodles + lactose hydrolyzed milk n=29 4. **Group M: comparator** (Modified whole milk): Regular formula with lactose (Nido, Perulac, S.A, Lima, Peru) n=28 | All GROUPS:  First 2 days: 150ml/kd/d All subsequent study days: >110kcal/kg/d | NR | Not for profit |
| Burande 2013^20^ | India | NR | outpatients | 1*. Saccharomyces boulardii* + Zinc n=35 2. Zinc n=35 | 1. 250 mg BID 2. Zinc: 10 mg/day in a child of <6 months and 20 mg/day for a child >6 months a day for 14 days | 5 days | Not for profit |
| Canani 2007^21^ | Italy | 3-36 | outpatients | **1.** *Lactobacillus casei rhamnossus* (Dicoflor 60) n=100 **2.** *Saccharomyces boulardii* (Codex) n=91 **3**. *Bacillus clausii* (Enterogermina) n=100 **4.** *Lactobacillus delbrueckii (bulgaricus) + Lactobacillus acidophilus + Streptococcus thermophilus + Bifodobacterium bifidum* (Lactogermina) n=97 **5**. *Enterococcus faecium* (Bioflorin) n=91 6. No treatment: Standard: oral rehydratation solution alone n=92 | **1**.6×10exp9 CFU in 20 ml water BID **2.** 5x10exp9 in 20 ml water BID **3**. 10exp9 CFU, in 20 ml water BID **4.** 10exp9 CFU, 10exp9 CFU, 10exp9CFU, 5x10exp8 CFU, in 20 ml water BID **5**. 7.5x10exp7 CFU in 20 ml water, BID | 5 days | Not specified |
| Carrague-Orendain 1999^22^ | Philippines | NR | mixed | 1. *Lactobacillus acidophilus + Lactobacillus bifidu*s (Infloran Berna; dose and duration not stated). N=35 2. Placebo (no details) n=35 | NR | NR |  |
| Cetina-Sauri 1994^23^ | Mexico | 3-36 | NR | 1. *Saccharomyces boulardii*  n=65 2. Placebo n=65 | 1. 600 mg/day 2. NA | NR | Not specified |
| Cezard 2001^24^ | France | 3-48 | Inpatients | 1. Racecadotril n=89 2. Placebo n=83 | 1. 1.5 mg/kg TID 2. NA | 5 days | Not specified |
| Chen 2010^25^ | Taiwan | 3-72 | Inpatients | 1. *Bifidobacterium mesentericus, Enterococcus faecalis, Clostridrium butyricum* (Bio-three) n=150 2. Placebo n=143 | 1. 2.5x10exp7CFU TID 2. NA | 7 days | Not for profit |
| Chew 1993^26^ | Guatemala | 0.5-6 | Inpatients | 1. Full-strength milk formula n= 80 2. Progressive reintroduction of full-strength milk formula (half-strength first 24H, 2/3 strength for the second 24H, and full-strength thereafter) n= 79 | 150ml/kg/day, divided into eight feedings of equal volume. | over 48 hours | Not for profit |
| Clemente-Yago 1993^27^ | Spain | 1-12 | Inpatients | 1. Cow’s milk (lactose-containing) formula N = 32 2. Lactose-free formula  N = 28 | NR | NR | Not specified |
| Cojocaru 2002^28^ | France | 3-36 | Recruited at ED | 1. Racecadotril n=81 2. No treatment (Standard) n=83 | 1. <9kg: 10mg/day >9kg: 20mg/day 2. NA | NR | Not specified |
| Conway 1989^29^ | UK | 1.5-12 | Inpatients | 1. 24 hours of ORS (Dextrolyte, Cowand Gate), followed by 24 hours of half strength and 24 hours of three quarter strength (SMAGoldCap Wyeth) before continued feeding with the full strength formula milk n=50 2. Especial formula HN25 feed (Milupa) until two days after the stools returned to normal followed on successive days by replacement of one, three, and then all HN25 feeds by full strength SMA Gold Cap n=50 3. Full strenght SMA Gold Cap from the time of admission n=50 4. Formula S (Cow and gate) from the time of admission n= 50 | NR | NR | Not specified |
| Cordier 1987^30^ | France | 6-30 | Inpatients | 1. Loperamide n=23 2. Placebo n=27 | 1. 7 drops/kg TID 2. NA | NR | Not specified |
| Correa 2011^31^ | Brazil | 6-48 | Inpatients | 1. *Sacccharomyces boulardii* (Floratil; Merck S.A., Rio de Janeiro, Brazil) n=95 2. Placebo n=91 | 1. 200mg BID 2. NA | 5 days | Not for profit |
| Costa-Ribeiro 2003^32^ | Brazil | 0-24 | Inpatients | 1. *Lactobacillus casei rhamnosus*(LGG) + small amount of inulin (320 mg) n=61 2. Placebo (inulin alone) n=63 | 1. 10 billionCFU QD 2. NA | until cessation of diarrhea (passage of 2 semi-formed or formed stools or no stool for 24 hours) or day 7, whichever came first | Not for profit |
| Crisinel 2015^33^ | Switzerland | 2-60 | Recruited at ED | 1. Zinc (Nutriset SAS, France) n=74 2. Placebo n=74 | 1. <6months:10 mg QD >6months: 20mg QD 2. NA | 10 days | Not for profit |
| Czerwionka-Szaflarska 2009^34^ | Poland | 2-36 | Recruited at ED | 1. LGG n=50 2. Standard n=50 | 1. 50 ml/kg body mass 2. NA | NR | Not specified |
| Dagan 1984^35^ | Israel | 0-12 | Inpatients | 1. Soy formula (Hyprovit; Hayes Ltd., Ashdod, Israel): soy protein 20%, fat 13% and carbohydrates (glucose and corn starch) 55%; 79 calories/100 ml.  n=40  2. Regular formula: cow's milk with 5% glucose added. n=35 | NR | NR | Not specified |
| Dalgic 2011^36^ | Turkey | 3-36 | Inpatients | **1**. *Saccharomyces boulardii* n=60 **2**. Zinc n=60 **3.** Lactose free (Bebelac Lactose Free Formula, 400 g; Nutricia, Istanbul, Turkey) n=60 **4**. *Saccharomyces boulardii* + Zinc n=60 **5**. *Saccharomyces boulardii i*+ Lactose free formula n=60 **6.** Zinc+ Lactose free n=60 **7**. Zinc + Lactose free formula + *Saccharomyces boulardii* n=60 **8**. Standard: oral and/or parenteral rehydration solutions n=60 | 1.250 QD 2. < 6 months: 10 mg BID > 6 months: 20 mg/day 3. NR 4. 250mg +10/20mg 5. NR 6. NR 7. NR | NR | Not specified |
| Das 2016^37^ | India | 3-60 | Inpatients | 1. *Saccharomyces boulardii* (lyophilized powdered form, (Econorm, Dr Reddy’s Laboratories) in sachets n=30 2. Placebo (similar to intervention) n=30 | 1. 250 mg/day 2. NA | NR | Not specified |
| Dewan 1995^38^ | India | 6-60 | NR | 1. Vitamin A n=108 2. No treatment (Standard) n=108 | 1. <10kg:100000 UI >10kg: 200000 UI 2. NA | NR | Not specified |
| Dinleyici 2013^39^ | Turkey | 3-120 | Inpatients | 1. Symbiotic:  *Lactobacillus acidophilus, Lactobacillus rhamnosus, Bifidobacterium bifidum, Bifidobacteriums longum, Enterococcus faecium +* 625 mg fructooligosaccharide, and vitamins A, B1, B2, B6, E, and C NBL Probiotic Gold® (Nobel, Turkey)  n=113 2. No treatment (Standard) n=96 | 1.  Probiotic: 2.5×109CFU probiotics prebiotic: 625 mg 2. NA | 5 days | Not specified |
| Dinleyici 2014^40^ | Turkey | 3-60 | Inpatients | 1*. Lactobacillus reuteri (BioGaia drops, BioGaia AB, Sweden; distributed by Eczacibasi in Turkey)* n=70 2. No treatment (Standard) n=70 | 1. 1x10exp8 CFU QD 2. NA | 5 days | Not specified |
| Dinleyici 2015^41^ | Turkey | 3-60 | outpatients | 1. *Lactobacillus reuteri* DSM17938  (BioGaia®, Stockholm, Sweden) n=32 2. No treatment (Standard) n=32 | 1. 1x10exp8 CFU 2. NA | 5 days | Not for profit |
| Dinleyici 2015^42^ | Turkey | 3-60 | NR | *1. Saccharomyces boulardii*  n=240 2. No treatment (Standard) oral rehydration solution and/or intravenous therapy n=160 | 1. 5 ×10exp9 cfu/250 mg 2. NA | 5 days | Not specified |
| Dugdale 1982^43^ | Australia | >6 | Inpatients | 1. Diluted Milk: half-strength full-cream milk for 24hours n=32  2. Regular formula: Immediate resumption of normal milk after rehydration n=28 | NR | NR | Not specified |
| Dupont 2009^44^ | Peru, Malaysia | 1-36 | Recruited at ED | 1. Smectite n=299 (in 2 countries) 2. Placebo n=303 (in 2 countries) | 1. <12mo: 6gr/day for 3d, then 3 g/day.  >13mo: 12g/day 2. NR | NR | Private |
| Dutta 2000^45^ | India | 3-24 | Inpatients | 1. Zinc sulphate  n=44 2. Placebo n=36 | 1. (177mg/day in three divided dosis ) 40 mg QD 2. NR | NR | Not specified |
| Dutta 2011^46^ | India | 6-24 | Inpatients | **1.** Zinc + single oral dose of vitamin A n=41 **2**. Micronutrient combination + single oral dose of vitamin A  n=39 **3.** Zinc + single oral dose of vitamin A n=44 **4.** Placebo n=43 | **1. Elemental zinc**: 20 mg QD 2. **Micronutrients combination:** zinc 20mg, iron 10 mg, copper 2 mg, selenium 40 mg, vitamin B12 1.4 mg, folate 100 mg. BID 3. **Vitamina A:** <1 year, 100 000 IU; >1 year, 200 000 IU. BID 4. NR | 14 days | Not for profit |
| Dutta 2011^47^ | India | 6-24 | Inpatients | 1. *Lactobacillus sporogenes (Bacillus. coagulans*) n=80 2. Placebo n=80 | 1. 2 TAB, BID 2. NR | 5 days | Mixed |
| El-Soud 2015^48^ | Egypt | 1-23 | Inpatients | 1. *Bifidobacterium lactis* n=25 2. Regular formula n=25 | 1. 14.5 x 106 CFU/100 ml daily  2. NA | 7 days | Not specified |
| Eren 2010^49^ | Turkey | 5-192 | Mixed | 1. *Saccharomyces boulardii* (Reflor Sanofi-Aventis, Turkey) + zinc supplement n=28 2. Yogurt (a fluid extracted from Pinar® yogurt made by a ferment containing *Lactobacillus bulgaricus* and *Streptococcus thermophilus*, 10exp7 microorganism/100 mL, provided by manufacturer) supplement n=27 | 1. <2 years:125mg BID >2 years 250 BID + ZINC as WHO Doses (10-20mg/d) 2. ≤ 6mo: 10mg/d  >6mo: 20mgd | NR | Not specified |
| Fajolu 2008^50^ | Nigeria | 6-24 | Recruited at ED | 1. Zinc sulphate monohydrate n=30 2. Placebo n=30 | 1. > 1 year: 20 mg/day  <1 year: 10mg/day  once a day 2. NA | 14 days | Not for profit |
| Fayad 1999^51^ | Egypt | 3-18 | Recruited at ED | 1. Soy based formula with sucrose: Nursoy (Wyeth Nutritionals International, Philadelphia, Pa) n=100 2. Soy based formula with lactose: Nursoy (Wyeth Nutritionals International) + lactose n=100 | BOTH:  150ml/kg/day in 8 equal volumes until resolution of diarrhea | 7 days or until diarrhea ceased. | Private |
| Francavilla 2012^52^ | Italy | 6-36 | Inpatients | 1. *Lactobacillus reuteri* DSM 17938 (4 × 10exp8 CFU/day) in a mixture of sunflower oil and medium-chain triglyceride oil  n=37 2. Placebo n=37 | 1. 5 drops BID 2. NR | NR | Not specified |
| Freedman 2015^53^ | Canada | 4-48 | Recruited at ED | 1. *Lactobacillus helveticus* Rosell-52 [5%] + *Lactobacillus rhamnosus* Rosell-11 [95%]) along with maltodextrin, magnesium stearate, and ascorbic acid n=66 2. Placebo n=66 | ND | NR | Private |
| Gharial 2017^54^ | Kenya | 3-60 | Inpatients | 1. Racecadotril + zn n=60 2. Zinc + placebo n=60 | 1. 10 mg per dose < 12 months; and 30 mg > 12 months 2. 10-20mg/day | 1. 3 days 2. 10 days | Not specified |
| Gilbert 1991^55^ | France | 2-24 | Recruited at ED | 1. Smectite n=18 2. Placebo n=18 | 1. <12 months: 3g/day >12months:6g/day. 2. NR | NR | Not specified |
| Grandy 2010^56^ | Bolivia | 1-23 | Inpatients | 1. *Saccharomyces boulardii* n=25 2. *Lactobacillus acidophilus, Lactobacillus rhamnosus, Bifidobacterium longum and Saccharomyces boulardii* n=26 3. Placebo n=25 | ALL GROUPS: BID dissolved in 20 ml of water | 5 days | Not for profit |
| Grandy 2012^57^ | Bolivia | 10-36 | Inpatients | 1. Yogurt + *Lactobacillus rhamnosus*+ *Lactobacillus bulgaricus*  n=37 2. *Saccharomyces boulardii*  n=37 | 1. 4.9 × 10exp8 CFU/g + 5 × 109 CFU/g  2. 5 × 10exp9 CFU | 5 days | Not for profit |
| Groothuis 1986^58^ | USA | <12 | outpatients | 1. Soy-based carbohydrate-free concentrate formula + lactose n=19 2. Soy-based carbohydrate-free concentrate formula + sucrose n=20 3. Soy-based carbohydrate-free concentrate formula+ polycose n=19 4. Soy-based carbohydrate-free concentrate formula + sucrose-polycose mixture n= 20 5. Soy n=20 | NR | 14 days | Mixed |
| Gu 2011^59^ | China | 1-36 | NR | *1. Bifidobacterium sp.+ Lactobacillus + Streptococcus thermophilus* n=100 2. Smectite n=60 | 1. <6 months: 1 tablet each time, twice to three times daily; 6 months - 3 years: 2 tablets each time, twice to three times daily. 2. <12 months:1.5 g, 3 times per day >12 months: 3 g, 3 times per day n=60 | NR | Not specified |
| Guandalini 2000^60^ | Italy, Egypt, Portugal, Croatia, Slovenia, Greece, UK, Poland, Israel, The Netherlands | 1-36 | mixed | ***1.*** *Lactobacillus GG* n=147 2. Placebo n=140 | 1. (10exp10 CFU/250 ml) 2. NR | NR | Not specified |
| Guarino 1997^61^ | Italy | 3-36 | outpatients | 1. *Lactobacillus casei strain GG* n=52 2. Placebo n=48 | 1. 3x10exp9 CFU; Dicoflor 30, Dicofarm SpA; Rome, Italy) BID 2. NR | 5 days | Not for profit |
| Guarino 2001^62^ | Italy | 3-60 | outpatients | 1.ORS + Dioctahedral smectite (Diosmectal; Malesci S.p.A., Florence, Italy) n=406 2. No treatment - ORS alone n=398 | 1. <12months: 3g/d >12mos: 6g/d BID 2. NA | 5 days | Private |
| Hafeez 2002^63^ | Pakistan | 6-60 | Inpatients | 1. *Saccharomyces boulardii* + ORS + nutrition appropiate for age n=55 2. ORS + nutrition appropiate for age n= 51 | 1. 250 mg BID 2. NA | 6 days | Not specified |
| Haffejee 1990^64^ | South Africa | <28 | Recruited at ED | 1. Cow's milk-based formula n= 120 2. Breast milk n= 79 3. Breast plus supplement n= 35 4. Soy formula n=75 | ALL GROUPS:  ad libitum | NR | Not for profit |
| Hegar 2015^65^ | Indonesia | 6-36 | outpatients | 1. *Lactobacillus rhamnosu*s R0011 +  *Lactobacillus acidophilus* R0052 u/d + zinc n= 56 2. Zinc + placebo n=56 | 1. 1.9×10exp9 +0.1 × 10exp9 CFU/day + zinc 20mg/day 2. zinc 20mg/day + placebo | 7 days probiotics  and 10 days for zinc | Private |
| Henker 2007^66^ | Ukraine | <48 | outpatients | 1. Non-pathogenic *Eschericchia coli s*train Nissle 1917 (Mutaflor suspension; Ardeypharm, Herdecke, Germany, with  n=55 2. Placebo n=58 | 1. 10exp8 viable microorganisms per millilitre;1, 2, 3 ml QD (1, 2, 3 years) 2. NR | NR | Private |
| Henker 2008^67^ | Ukraine | 1-47 | Inpatients | 1. Suspension for oral use containing nonpathogenic *Escherichia coli* strain Nissle 1917 (Mutaflor Suspension, Ardeypharm, Herdecke, Germany; 108 viable micro- organisms per mL)  n=75 2. Placebo n=76 | 1. *Infants <1 year: 1 mL QD *Toddlers >= 1 to < 3 years 1 mL BID  *Toddlers >3 to <4 years 1 mL 3 TID 2. NA | 21 days | Not specified |
| Henning 1992^68^ | Bangladesh | 12-60 | Inpatients | 1. Vitamin A (retinyl palmitate equivalent to 200 000UI along with 25UI of vit E) n=46 2. Placebo contained only vit E n= 37 Both supplied by Hoffman La Roche | 1. 1ml 2. Same | NR | Not for profit |
| Heydarian 2010^69^ | Iran | 6-144 | Inpatients | 1. Probiotic yogurt consisted of 10exp9 CFU/dose with *Lactobacillus bulgaricus, Lactobacillus acidophilus Bifid bacterium* and *Streptococcus thermophilus n= 50 2. Placebo: traditional yogurts n= 50* | 1. 100cc/day 2. NA | 5 days | Not specified |
| Hoekstra 2004^70^ | Egypt | 1-36 | Inpatients | 1. SRO + mixture of non-digestible carbohydrates: Soy polysaccharide, Alfa-cellulose, Gum Arabic, Fructo-oligosaccharides, Inulin, Resistant starch (NV Nutricia, Wageningen, The Neth- erlands). 100 ml of oral rehydration solution contained 1 g of this mixture. n=75 2. Placebo n=79 | NR | Until diarrhea stopped | Mixed |
| Hoque 2005^71^ | Bangladesh | 3-59 | outpatients | 1. Zinc sulphate tablet (Rodael Pharma) n=534 2. Placebo n=533 | 1. 20mg (NR frequency) 2. NA | NR | Not for profit |
| Htwe 2008^72^ | Myanmar | 3-120 | Inpatients | 1. *Saccharomyces boulardii*  n=50 2. No treatment (Standard): SRO n=50 | 1. 250 mg BID 2. NA | 5 days | Not specified |
| Huang 2012^73^ | Taiwan | 3-168 | Inpatients | 1. *Enterococcus faecalis* + *Clostridium butyricum* + *Bifidobacterium mesentericus* n=82 2. No treatment (Standard) n=77 | 1. 3.48 x 10exp8 CFU of a mixture of *E. faecalis* (3.17 108 CFU), *C. butyricum* (2.0 107 CFU), and *B. mesentericus* (1.1 107 CFU 2. NA | NR | Not for profit |
| Huang 2014^74^ | Taiwan | 3-168 | Inpatients | 1. BIO-THREEE tablet: (TOA Pharmaceutical Co. Ltd., Tokyo, Japan) contains a total of 3.48 10exp8 CFU of a mixture of *E. faecalis* (3.17 10exp8 CFU), *Clostridium butyricum* (2.0 x 10exp7 CFU), and *Bacillus mesentericus* (1.1 x 10exp7 CFU)  n=82 2. No treatment: Control: intravenous fluid, oral rehydration solutions, oral rice, and half-strength milk formula n=77 | 1. *<6 years: 1 tablet tid  *6 - 12 years:2 tablets tid *>12 years 3 tablets tid 2. NA | 7 days | Not for profit |
| Islek 2014^75^ | Turkey | 2-60 | mixed | 1. Symbiotic group:  *Bifidobacterium lacti*s B94 + 900 mg inulin containing preparation (Maflor® sachet, Mamsel, Turkey) n=90 2. Placebo group: maltodextrin n=89 | 1. QD (no more info) 2. NR | 5 days | Not for profit |
| Isolauri 1986^76^ | Finland | 6-34 | Inpatients | 1. Lactose free (milk free group: all milk products removed from diet) n=27   2. Regular formula: Milk containing group (including milk products) n=38 | NR | 1month | Not for profit |
| Isolauri 1991^77^ | Finland | 2-17 | Inpatients | 1. *Lactobacillus casei* sp strain GG -fermented milk, 125g product, Lactose-free milk n=24 2. *Lactobacillus* GG  n=23 3. Placebo (fermented-pasteurized) yogurt with an insignificant amount of lactic acid bacteria. | 1. 10exp10-11 CFU BID 2. 10exp10-11 BID 3. 125g BID | 5 days | Not for profit |
| Isolauri 1994^78^ | Finland | 1-36 | Inpatients | 1*. Lactobacillus casei* GG  n=21 2. No treatment (Standard) n=21 | 1. 10exp10 CFU, BID 2. NA | 5 days | Not specified |
| Jiang 2016^79^ | China | 3-36 | NR | 1. Zinc N=51 2. No treatment n=52 | 1. <6m: 10 mg/day >6m: 20 mg/day 2. NA | NR | Not specified |
| Kang 2016^80^ | India | 3-60 | Inpatients and Outpatients | 1. Racecadotril n=167 2. Placebo n=162 | 1. 1.5mg/kg/dose TID 2. The same dosis stated | 3 days | Not for profit |
| Kaplan 1999^81^ | USA, Mexico | 24-132 | outpatients | 1. Loperamide HCL 0.5 mg/5mL n=130 2. Placebo n=128 | 1. 2-5 years: 3.0 mg  6- 11 Years: 4.0 mg  9-11 years: 6.0mg *Doses of approximately 0.2mg/kg (range 0.14mg/kg/ day-0.28mg/kg/day). 2. NA | 48 hours | Private |
| Karamyyar 2013^82^ | Iran | 9-60 | Inpatients | 1. Zinc sulfate + ORS n=188 2. ORS alone n=191 | 1. 1 mg/kg/day TID 2. NA | 5 days | Not for profit |
| Karrar 1987^83^ | Saudi Arabia | 3-36 | Inpatients | 1. Rehydration + Loperamide syrup n=17 2. Rehydration + Loperamide syrup n=18 3. Rehydration plus placebo syrup  n= 18 | 1. 0.8mg/kg/day 2. 0.4 mg/kg/day 3. NA | 7 days or until diarrhea resolved | Mixed |
| Kassem 1983^84^ | Egypt | <24 | outpatients | 1. Loperamide n=50 2. Placebo n=50 | 1. 0.24 mg/kg/day (one drop/kg/dose) TID 2. Placebo | Until termination of the diarrheal episode and for a minimal period of 48 hours. | Not for profit |
| Khan 2012^85^ | Pakistan | 2-60 | Recruited at ED | 1. *Saccharomyces boulardii*  n=210 2. No treatment (Standard): ORS alone n=210 | 1. 250mg BD 2. NA | 5 days | Not specified |
| Khanna 2005^86^ | india | 6-144 | Inpatients | 1. Packets of puffed rice powder + *Lactobacillus acidophillus* (LA) (Lactrol, Raptakos) n=48 2. Puffed rice powder alone n=50 | BOTH 1 packet daily (15 billion tyndalized LA cells) | 3 days | Not specified |
| Kianifar 2009^87^ | Iran | 6-36 | Inpatients | 1. *L. acidophilus* + one billion *Bifidobacterium bifidum*, (Lab. Farmaceutico SIT S.r.I., Mede, Pavia, Italy) in the form of a powder  n=34 2. Placebo n=34 | Powder reconstituted by parents with 5–10 ml of water and administered as a suspension in 5–10 ml of water TID | 5 days | Not for profit |
| Kowalska-Duplaga 1999^88^ | Poland | 0-24 | NR | *1. Lactobacillus acidophilus, Bifidobacterium bifidum, Lactobacillus bulgaricus*  n=86 2. Placebo (identical) n=86 | 1. 3.2 x 109 CFU/day for 5 days 2. NA | 5 days | Not specified |
| Kurugol 2005^89^ | Turkey | 3-84 | Inpatients | 1. *Saccharomyces boulardii* (Sanofi, Turkey) n=100 2. Placebo n=100 | 1. 250mg BID 2. NA | 5 days | Not specified |
| Leake 1974^90^ | USA | 0.25-8 | Inpatients | 1. Soy-based (lactose-free) formula (Isomil®):  n=11 2. Regular formula: Cow’s milk formula N = 11 | NR | NR | Private |
| Lee 2015^91^ | Korea | 3-84 | Recruited at ED | 1. Six types of probiotics:  *Bifidobacterium longum (IBG), Bifidobacterium lactis (BL), Lactobacillus acidophilus (LA), Lactobacillus rhamnosus (LRH), Lactobacillus plantarum, Pediococcus pentosaceu*s  n=24 2. Placebo n=24 | 1. 10exp8 CFU/each strain per sachet (1 g). BID 2. NA | 7 days | Not for profit |
| Lei 2006^92^ | Ghana | <60 | mixed | 1. KokoSour Water (live lactic acid bacteria mainly *Weissella confusa* + *Lactobacillus fermentum*) n=97 2. No treatment n=93 | 1. 10exp8 CFU. Dose: 300ml day | 5 days | Not for profit |
| Lexomboon 1994^93^ | Thailand | 1-24 | Outpatients | 1. Smectite n=34 2. No treatment (standard) n= 34 | 1. 1.5gr q 12h 2. NA | NR | Private |
| Lifshitz 1991^94^ | Brazil | <12 | Inpatients | 1. Diluted (2/3) cow’s milk:  N = 10 2. Cow’s milk formula (Nanon®):  N = 10 3. Lactose-free milk formula (Portagen®):  N = 10 4. Lactose-free milk formula (Pregestimil®): N = 10 5. Lactose-free milk formula (Prosobee®):  N = 10  COMPARISON 1. Group 1 + 2 versus Group 3 + 4 + 5  COMPARISON 2. Not eligible since Group 1 dilution < 50% | ND | NR | Not specified |
| Lopez-Hernandez 1998^95^ | Mexico | NR | NR | 1. *Saccharomyces boulardii* + SRO n=25 2. SRO + placebo n=25 | 1. 200 mg TID 2. NA | 5 days | Not specified |
| Lozano 1994^96^ | Colombia | 1-24 | Inpatients | 1. Lactose free formula (AL- 110 Nestlé Lab) n=28 2. Regular formula (NAN 1 & NAN 2) n=24 | NR | NR | Not specified |
| Madkour 1993^97^ | Egypt | 3-24 | Inpatients | 1.Dioctahedral smectite (by Bcaufour-Ipsen, France, via the WHO) n=45 2. Placebo n=45 | 1. 1.5g sachets QID 2. NA | 3 days | Mixed |
| Majamaa 1995^98^ | Finland | 4-35 | Inpatients | 1.Freeze-Dried LGG  n=16 2. *Lactobacillus casei* subsp. *rhamnosus (Lactophilus) LGG* n=14 3. Combination of: *Streptococcus thermophilus + Lactobacillus delbrückii* subsp. *Bulgaricus + Lactobacillus casei subsp. rhamnosus* (Yalacta) n= 19 | Children received one of the three lactic acid bacteria preparation BID no more info | 5 days | Not for profit |
| Manyal 2015^99^ | India | 1-72 | Inpatients | 1. *Lactobacillus sporogenes* + Zinc  n= 31 2. Fluids + zinc n=31 | 1. Sachets with 150 million spores TID 2. 10-20 mg/day | 3 days | Not specified |
| Mao 2008^100^ | China | 6-36 | Inpatients | 1. Milk-based lactose-free formula + *Bifidobacterium lactis* Bb 12 + *Streptococcus thermophilu*s TH4 n=71 2. Lactose Free formula + *Bifidobacterium lacti*s + *Streptococcus thermophillus* n=70 3. Milk-based lactose-free formula n=71 | 1. 10exp8 CFU/g / & 5x10exp7 CFU/g 2. 10exp9 CFU/g / & 5x10exp8 CFU/g | NR | Not specified |
| Maudgal 1985^101^ | UK | NR | Inpatients | 1. Diluted Milk: “Graduated” re-feeding with milk or cow’s milk formula: 1⁄4 strength for 24 hrs, 1⁄2 strength for further 24 hrs, then full strength:  n=86 2. Regular formula: Immediate re-feeding with full strength milk or cow’s milk formula n=89 | NR | NR | Not specified |
| Melendez-García 2007^102^ | Guatemala | 3-71 | outpatients | 1. Racecadotril (Hidrasec-Ferrer) n=25 2. Kaolin Pectin (Ind. Bioquimicas S.A) n= 25 | 1. 10mg 2. Kaolin 3g and Pectin 66mg/15ml | NR | Private |
| Michael 2014^103^ | Egypt | 24-72 | mixed | **Group I** (hospitalized, n=60)  -Treatment group: racecadotril + ORS n= 30 -Control group: ORS only n= 30 **Group II** (Outpatient, n=90) -Group II A: treatment group: racecadotril + nitazoxanide  n=15 -Control group: nitazoxanide only  n=15  -Group II B:  -Treatment group: racecadotril + metronidazole  n=15 -Control group: metronidazole only  n=15 Group II C -  -Treatment group racecadotril + ORS n= 15 -Control group: ORS only n=15 | -Racecadotril: 1.5 mg/kg TID -Nitazoxanide: 100mg/5ml oral suspension TID -Metronidazole: 40mg/ml syrup TID | 7 days | Not specified |
| Milocco 1999^104^ | Italy | 0-60 | Inpatients | 1. Smectite n=16 2. Standard (no treatment) n=19 | 1. <3 year:1.5g q12  >3 year: 3g q12  2. NA | 3 days | Not specified |
| Misra 2009^105^ | India | 1-36 | Inpatients | 1. *Lactobacillus rhammosus GG* n=111 2. Placebo n=118 | 1. 10exp9 live every day 2. NA | 10 days | Not for profit |
| Moal 2007^106^ | Ecuador | 1-24 | Inpatients | 1.  *Lactobacillus acidophilus LB* + ORS (Laboratoire du Lacte ́ ol) n=42 2. Placebo sachets + ORS n=38 | 1.Initial dose of 2 sachets (L acidophilus LB-ORS or placebo-ORS) and subsequently 1 sachet every 12 hours. 10 billion heat- killed 2. NA | 72 hours | Not specified |
| Movahedi 2008^107^ | Iran | 3-12 | outpatients | 1. Zinc n=64 2. No treatment (Standard) n=89 | 1. 5 mg BID 2. NA | 14 days | Not for profit |
| Mujawar 2012^108^ | India | 24-60 | outpatients | 1. Dioctahedral smectite n=58 2. No treatment: ORS therapy only n=59 | 1. 1.5 gr 3 TID 2. NA | 5 days | Not specified |
| Naidoo 1981^109^ | South Africa | NR | Inpatients | 1. Lactose-free soy-based formula (Isomil®): n=56 2. Standard (lactose-containing) cow’s milk formula n+56 | NR | NR | Not specified |
| Narayanappa 2008^110^ | India | 3-36 | Inpatients | 1. Standard therapy + probiotic (Bifilac): *Bacillus mesentericus, Clostridium butyricum, Lactobacillus sporegens + Streptococcus faeecalis  n=40* 2. Standard therapy + placebo n=40 | 1. 1 sachet TID 2. NA | 14 days | Private |
| Narkeviciute 2002^111^ | Lithuania | 6-48 | Inpatients | 1. Dioctahedral smectite n=28 2. Placebo n=26 | 1. 3g at the beginning of rehydratation, then: <10kg: 1.5g TID  10-20kg: 1.5g QID 2. NA | until 24 hrs after resolution of the stools | Private |
| Negi 2015^112^ | india | 60-144 | Recruited at ED | 1. Zinc n= 67 2. Placebo n= 67 | 1. 20 mg BID 2. NA | 14 days | Not for profit |
| Nixon 2012^113^ | USA | 6-72 | outpatients | 1. *Lactobacillus* GG (LGG) powder in capsules form n=77 2. Placebo capsules n=78 | 1. BID, No colony number provided 2. NA | 5 days | Mixed |
| Noreen 2016^114^ | Pakistan | 1-12 | Inpatients | 1. Lactose free formula n=35 2. Lactose containing formula  n=34 | NR | NR | Not specified |
| Oandasan 1999^115^ | Philippines | NR | Inpatients | 1. *Lactobacillus acidophilius + Lactobacillus bifidus* (Infloran, Berna);  n=47 2. Placebo n=47 | 1. 3 x 109 of each organism/day  2. NA | NR | Not specified |
| Owens 1981^116^ | England, Lybia | 1-48 | Inpatients | 1. Loperamide n=48 2. Placebo n=45 | 1. 0.2mg/kg/day 2. NA | 5 days | Not specified |
| Ozkan 2007^117^ | Turkey | 6-120 | mixed | 1. *Saccharomyces boulardii* n=16 2. Placebo n=11 | 1. 250 mg dissolved in 5 ml of water orally BID 2. NA | 7 days | Private |
| Pant 1996^118^ | Thailand | 1-24 | Inpatients | 1. *Lactobacillus GG* n=20 2. Placebo n=19 | 1. 10exp10 to 10exp11 CFU as freeze-dried preparation mixed in 10ml of ORS, BID  2. NA | 2 days | Not specified |
| Pashapour 2006^119^ | Iran | 6-24 | Inpatients | 1. Yogurt with: fat 2.5%, sugar 3%, lactic acid 1%, water 74%, *Lactobacillus bulgaris* 50,000/ml, *Streptococcus thermophilus* 50,000/ml and pH=4.7 n= 40 2. Placebo n= 40 | 1. 15ml/Kg /d 2. NA | NR | Not specified |
| Passariello 2012^120^ | Italy | 6-36 | outpatients | 1. Symbiotic  *Lactobacillus paracasei,* arabinogalactan, xilooligosaccharides (Flortec Bracco, Milan, Italy). n=52 Placebo n=55 | 1. one sachet dissolved in 50 mL of water containing *Lactobacillus paracasei* B21060, 2.5 x 10exp9 CFU, + arabinogalactan, 500 mg + xilooligosaccharides, 700 mg b.d. (Flortec Bracco, Milan, Italy). BID 2. NA | 5 days | Mixed |
| Patel 2009^121^ | India | 6-59 | Recruited at ED | 1. Zinc sulfate n=264 2. Zinc + Copper: copper sulfate equivalent to 2 mg/5 ml elemental copper in addition to zinc in the aforementioned dose. n=273 3. Placebo n=271 (supplements and placebo were prepared by Universal Medicaments Pvt. Ltd, Nagpur, India) | 1. 0.5 ml/kg/ day of the syrup (the dose of zinc was 2 mg/kg/day and of copper was 0.2 mg/kg/day) 20 mg/5 ml of elemental zinc. 2, Zinc same dose + copper 0.2 mg/kg/day 3. NA | 14 days | Not for profit |
| Patel 2015^122^ | India | 1-60 | mixed | 1. Zinc sulphate n=47 2. Standard: ORS, intravenous fluid and antibiotics n=53 | 1. >6 months:10mg/day >6 months: 20mg/day 2. NA | 14 days | Not for profit |
| Patro 2010^123^ | Poland | 3-48 | mixed | Zinc n=81 2. Placebo n=79 | 1. <6 months: 10mg/day >6 months: 20 mg/day 2 doses 2. NA | 10 days | Mixed |
| Phavichitr 2013^124^ | Thailand | 3-72 | Inpatients | 1. *Lactobacillus acidophilus + Bifidobacterium bifidum* viv. lyophilisat with lactose + magnesium stearate as excipients. n=53 2. Placebo: containing only the excipients.  n=53 | 1. Each probiotic capsule contained a minimum of one billion organism: < 1 year: one capsule BID >1 year: one capsule TID 2. NA | 7 days | Mixed |
| Pieacik-Lech 2013^125^ | Poland | 4-60 | Inpatients | 1. *Lactobacillus GG*: LGG (Dicoflor 30, Vitis Pharma, Poland) + Smectite (Smecta, Beaufour, Ipsen, France) 3g daily n=44 2. Placebo: glucose n=44 | 1. LGG: 6x10exp9 of QD  + Smectite: 3g QD 2. NA | 7 days or until the diarrhea stopped | Not for profit |
| Placzek 1984^126^ | UK | <18 | Inpatients | 1. Diluted Milk: “Graduated” re-feeding with milk or cow’s milk formula: 1⁄4 strength for 24 hrs, 1⁄2 strength for 24 hrs, 3⁄4 strength for 24 hrs, then full strength: N = 25 2. Regular Milk: Immediate re-feeding with full strength cow’s milk formula:  N = 23 | NR | NR (until discharge) | Not specified |
| Pociecha 1998^127^ | Poland | 6-36 | Recruited at ED | 1. Smectite + *Lactobacillus rhamnossus* n= 104 2. *Lactobacillus rhamnossus* n=110 3. Placebo n=108 | 1. 6-12 months: 1.5 g, 2 time day 13-36 months: 3 g, 3 times day 2. The same dose of *Lactobacillus rhamnossus*, no Smectite 3. NA | 6 days | Not specified |
| Polat 2003^128^ | Turkey | 2-29 | Recruited at ED | 1. Zinc sulfate  n=92 2. Placebo n=79 | 1. (20 mg/day elemental zinc in 3 doses during diarrhea); 6.7 mg zinc TID, diluted in 5 mL of liquid. 2. 250 mg glucose TID h diluted in 5 mL of liquid. | 7 days after recovery | Not specified |
| Quak 1989^129^ | Singapore | NR | Inpatients | 1. Lactose-containing formula:  n=25 2. Full strength lactose-containing formula from the start of the refeeding period.  n=25 3. Disaccharide-free soy formula in full strength from the beginning of refeeding. n=25 4. Full strength cow's milk formula with lactose and low fat.  n=25 | 1/4 strength formula first 12 hours, followed by 1/2 strength for the next 12-hours. Subsequently 3/4 strength and full-strength formula. In all 4 groups, the total volume of feed was calculated according to body weight and age. Equal volume 3-hourly feeds were given. | 48 hours | Not specified |
| Rafeey 2008^130^ | Iran | NR | Inpatients | 1. *Lactobacillus acidophilus* yogurt n=40 2. *Lactobacillus acidophilus* supplement (made by Tabriz Drug Research Center) n=40 3. Conventional Yogurth n=40 4. Placebo n=40 | 1. 300 gr/day, semi-fluid 2. 5x10exp10 CFU 2 capsules/day  3. 300 gr/day, semi-fluid 4. NR | NR | Not for profit |
| Ransome 1984^131^ | South Africa | 3-36 | Inpatients | 1. MILK  Full-strength cow's milk FCM n= 29 2. MILK *First day of admission: half-strength milk *2nd and 3rd day: 2/3 strength milk *4th day FCM n= 32 | NR | NR | Not specified |
| Raza 1995^132^ | Pakistan | 1-24 | Inpatients | 1. *Lactobacillus* GG (supplied by Scientific Hospital Supplies, Liverpool, United Kingdom) n=21 2. Placebo n=19 | 1. 10 exp10 CFU, BID Mixed in 10ml of ORS 2. NA | 2 days | Mixed |
| Rehman 2013^133^ | Pakistan | 6-24 | Inpatients | 1. Smectite + zinc sulfate n=99 2. Zinc + placebo n=97 | 1. Diosmectite:  <12mo 1.0 g 12-24mo 1.5 g TID  diluted in water 2. NA | 5 days | Not specified |
| Rerksuppaphol 2010^134^ | Thailand | 2-84 | Inpatients | 1. *Lactobacillus acidophilus + Bifidobacterium bifidum* (InfloranH, Berna, Switzerland) AT 4·Celsius n=23 2. *Lactobacillus acidophilus* + *Bifidobacterium bifidum* (InfloranH, Berna, Switzerland) At room temperature n=22 3. Placebo n=22 | 1. A minimum of 10exp9 /capsule, both probiotics) 2. 1. A minimum of 10exp9 /capsule, both probiotic (Difference in storage temperature) 3. NA | NR | Not for profit |
| Riaz 2012^135^ | India | 3-59 | Inpatients | 1. *Saccharomyces boullardii* (SB) (Econorm, Dr Reddy’s Laboratories) mixed with puffed rice powder.  n=54 2. Placebo n=54 | 1. 250 mg BID 2. NA | 5 days or until the recovery. | Not specified |
| Ritchie 2010^136^ | Australia | 4-24 | Inpatients | 1. *Lactobacillus casei strain GG* n=33 2. Placebo n= 31 | 1. 5x10exp9 TID 2. NA | 3 days | Not specified |
| Rosenfeldt 2002^137^ | Denmark | 6-36 | Inpatients | 1. *Lactobacillus rhamnosus* 19070-2 + *Lactobacillus reuteri* DSM 12246, Lyophilized n=30 2. Placebo: skim milk powder and dextrose anhydrate n=39 | 1. 10exp10 CFU of each strain BID  2. NA | 5 days | Not specified |
| Rosenfeldt 2002^138^ | Denmark | NR | outpatients | *Lactobacillus rhamnosus* 19070-2 + *Lactobacillus reuteri* DSM 12246 n=24 2. Placebo n=19 | 1. 10exp10 CFU of each strain BID  2. NA | 5 days | Not specified |
| Roy 1997^139^ | Bangladesh | 3-24 | Recruited at ED | 1. Zinc acetate + Multivitamin syrup n=57 2. Placebo: Multivitamin syrup n=54 | 1. 20 mg/day zinc elemental in 3 doses (1 ml of zinc syrup: 1.3 mg of elemental zinc) prepared by Square Pharmaceuticals Co, Bangladesh, and contained in each 5 ml:  vitamin A 3000 IU, --vitamin D 600 IU vitamin B-1 1.2 mg, riboflavin 2.0 mg, vitamin B-6 0.6 mg, nicotinamide 6.0 mg calcium d-pantothenate 6.0 mg  2. Same multivitamin presentation (without zinc) | 2 weeks | Not for profit |
| Sachdev 1998^140^ | India | 6-18 | Inpatients | 1. Zinc  n=25 2. Placebo: glucose n=25 | 1. (55mg zinc sulfate)  20mg BID 2. NA | NR | Not specified |
| Salazar 2000^141^ | Peru | 3-35 | Inpatients | 1. Racecadotril n=68 2. Placebo n=67 | 1. 1.5mg/kg 2. NA | 5 days or until the diarrhea stopped, whichever came first. | Private |
| Salazar 2004^142^ | Peru | 6-36 | Inpatients | 1. Milk formula (Valio Ltd, Helsinki, Finland) as milk powder to reconstitute with water containing *Lactobacillus casei* strain *GG (LGG)*  n=90 2. Similar milk formula not containing LGG n= 89 | 1. 150 ml/kg/day to a maximum of 1000 ml/day (10exp9 CFU/ml) On average, each serving of 100 ml will supply 1011 cfu of LGG for those receiving the formula containing LGG. Calculated based on Weight 2. NA | 5 days | Mixed |
| Saneian 2012^143^ | Iran | 1-24 | Inpatients | 1. Lactose-free formula N = 37 2. Regular formula: Lactose-containing formula N = 37 No more information | NR | 7 days | Not for profit |
| Santos 2009^144^ | Spain | 3-36 | outpatients | 1. Racecadotril n=91 2. Placebo n=88 | 1. <9kg: 10mg TID 9-13kg: 20 mg TID >13kg: 30mg TID 2. NA | 7 days | Private |
| Sarker 2005^145^ | Bangladesh | 4-24 | Inpatients | 1. *Lactobacillus paracasei*  n=115 2. Placebo: whey-protein/skim-milk powder blend n=115 | 1. 5x10exp9. Lyophilized BID 2.NA | 5 days | Mixed |
| Sazawal 1995^146^ | India | 6-35 | outpatients | 1. Zinc gluconate + Multiple micronutrients (Sandoz, India (Bombay) n=456 2. Multiple micronutrients alone n= 481 | 1. Elemental zinc: 20 mg/day zinc elemental Micronutrients: Each daily 10-ml dose contained: vitamins A (1600 units),  B1 (1.2 mg),  B2 (1.0 mg),  B6 (1.0 mg),  D3 (200 IU), E (6 mg) and  niacinamide (20 mg) 2. Micronutrients same doses. Without zinc | NR | Not for profit |
| Shan 1997^147^ | China | 2-36 | Inpatients | 1. Smectite n=20 2. Probiotic: *Lactobacillus helveticus* n=10" | 1. <1 years: 1/3 bag 2 to 3 times daily 1-3 years: 1/2 bag 3 times daily 2. <1 years: 0.4g 3 times dayly 1-3 years:0,8g 3 times daily | NR | Not specified |
| Shamir 2005^148^ | Israel | 6-12 | Inpatients | 1. Cereals (Baby-Biocal, Remedia, Israel) with probiotics: *Streptococcus thermophilus, Bifidobacterium lactis, Lactobacillus acidophilus* + zinc, in a lactose free, soy protein-based formula (Remedia Tsimchit, Remedia, Israel). n=33 2. Cereals (Baby-Biocal, Remedia, Israel) without probiotics and zinc, in a lactose free, soy protein-based formula n= 32 | 1. 2x10exp9 each strain *10 mg of zinc/day *0.3 grams of fructo-oligosaccharides 7x10exp9 CFU/100g cereal 2. Cereal, similar content, whiout Probiotic, and without zinc | 7 days | Private |
| Shornikova 1997^149^ | Finland | 6-36 | Inpatients | 1. *Lactibacillus reuteri* SD2112 by Bio-Gaia Biologics, Inc. (Raleigh, NC, U.S.A.) n=19 2. Placebo n=21 | 1. 10exp10-11 CFU per gram, QD  2. NA | 5 days | Private |
| Shornikova 1997^150^ | Finland | 7-36 | Inpatients | 1. *Lactobacillus* *reuteri* in large and small dosages n=41 2. Placebo n=25 | 1. Large dosage: 10exp10 to 11 CFU QD Small dosage contained 10exp7 CFU QD 2. NA | 5 days | Private |
| Shornikova 1997^151^ | Russia | 1-36 | Inpatients | 1. *Lactobacillus* GG  n=59 2. Placebo n= 64 | 1. 5x10exp9 CFU, BID  2. NA | 5 days | Mixed |
| Simakachorn 2000^152^ | Thailand | 3-24 | Inpatients | 1. *Lactobacillus acidophilus-LB*  n=37 2. Placebo n=36 | 1. 2×10exp10, 160 mg, BID (Each child was assigned a numerically coded package containing six sachets, to be taken in 5 ml of water as follows: first dose of one sachet on admission, then one sachet every 12 hours, with a total of five doses corresponding to a 48-hour treatment) 2. NA | 3 days | Private |
| Simakachorn 2004^153^ | Thailand | 3-24 | Inpatients | 1. Lactose-Free Formula (Dumex®) n=40 2. Lactose-containing formula (Dumex® Infant Formula) n= 40 | 1. 90 ml/kg/day of either a lactose-free formula or a lactose-containing formula 2. NA | 7 days | Mixed |
| Sindhu 2014^154^ | India | 6-60 | outpatients | 1. *Lactobacillus rhamnosus* GG (by i-Health Inc Cromwell, Connecticut) n=65 2. Placebo n= 59 | 1. 1×10exp10 QD  2. NA | 4 weeks | Not for profit |
| Strand 2002^155^ | Nepal | 6-35 | outpatients | 1. Zinc syrup + placebo capsule n=447 2. Zinc syrup + Vitamin A capsule n=450 3. Zinc syrup + placebo capsule n= 448 3. Placebo syrup + placebo capsule n=452 | 1. Zinc: infants: 6ml/day   older: 12ml/day 2. Zinc same + Vitamin A at enrollment (not specified dose) 3. Same dose of Zinc, no Vitamin A 4. Placebo syrup for both. No more information | 7 days | Not for profit |
| Su 2014^156^ | China | 6-24 | outpatients | 1. Zinc n=30 2. Zinc + lactose free n=30 3. Lactose free n=30 | 1. Zinc gluconate: 40mL, once daily 2. Same dose of Zinc. Lactose free, no information  23. No information on Lactose free | 10 days | Not specified |
| Sutton 1968^157^ | Canada | <24 | Inpatients | 1. Lactose-free formula (glucose 6.4%) n = 48 2. Lactose-containing formula (lactose 6.4%)  n= 49 | NR | Until discharge | Not specified |
| Szymanski 2006^158^ | Poland | 2-72 | Mixed | 1. *Lactobacillus rhamnosus* strains  Three: (573L/1; 573L/2; 573L/3) (Lakcid L; Biomed, Lublin, Poland) n=49 2. Placebo n=44 | 1.2x10exp10 CFU BID 2. NA | 5 days | Not specified |
| Teran 2009^159^ | Bolivia | 1-24 | Inpatients | 1. Nitazoxanide (100mg/5ml) n= 29  2.Combination of oral probiotics: *Lactobacillus acidophilus, Lactobacillus rhamnosus, Bifidobacterium longum, Saccharomyces boulardii* n= 30 3. Oral or systemic rehydration solutions alone n= 31 | 1. Nitazoxanide 15mg/kg/day BID 2. Oral probiotics: 1g BID | 1. 3 days  2. 5 days | Not specified |
| Torabi 2011^160^ | Iran | 6-24 | Inpatients | 1. Zinc n=200 2. Placebo n=200 | 5 mg once per day | NR | Not for profit |
| Torrez 2013^161^ | Bolivia | 6-59 | Inpatients | 1. Racecadotril n=55 2. Placebo n=55 | NR | NR | Not specified |
| Touhami 1989^162^ | Algeria | <9 | Inpatients | 1. Diluted Milk n=40 2. Regular formula n=40 | NR | NR | Not for profit |
| Turck 1999^163^ | France | 24-120 | outpatients | 1. Racecadotril + placebo (to keep study blind) n=52 2. Loperamide + placebo (to keep study blind) n=50 | 1. 1.5 mg/kg TID 2. 0.03mg/kg (four drops of a solution containing 0.2 mg/mL loperamide) TID | Until recovery | Private |
| Upadhyay 2014^164^ | India | 6-60 | outpatients | 1. *Lactobacillus GG*  n=100 2. No probiotic medication (Standard) n=100 | 1. 10 billion CFU/day 2. NA | 5 days | Not specified |
| Urganci 2001^165^ | Turkey | 2-29 | Inpatients | 1. *Saccharomyces boulardii* n=50 2. Placebo n=50 | 1. 250 mg QD 2. NA | NR | Not specified |
| Vandenplas 2011^166^ | Belgium | 3-186 | outpatients | 1. Symbiotic by Phacobel (Tinlot, Belgium): *Streptococcus thermophilus* 20mg *Lactobacillus rhamnosus* 28mg *Lactobacillus acidophilus* 28mg *Bifidobacterium infantis* 20 mg  *Bifidobacterium lactis* 20mg Fructo-oligosaccharides 20mg Ascorbic acid 1.2mg n=57 2. Placebo n=54 | 1. 1 capsule/day 2. NA | 7 days | Mixed |
| Villaruel 2007^167^ | Argentina | 3-24 | outpatients | 1. *Saccharomyces boulardii*  n=50 2. Placebo n=50 | 1. Capsules of 250 mg <1 year: QD  >1year: BID 2. NA | 6 days | Not specified |
| Vivatvakin 1992^168^ | Thailand | 1-24 | Inpatients | 1. Smectite by Beaufour Ipsen International (c / 0 Pacific Healthcare, Bangkok, Thailand) + ORS n=32 2. ORS alone n= 30 | 1. <3 Kg: 1.5g BID  4-10 Kg: 1.5g TID 11-15 kg: 1.5g QID 2. NA | 5 days | Private |
| Vivatvakin 2006^169^ | Thailand | 1-24 | Inpatients | *1. Lactobacillus acidophilus + Bifidobacterium bifidum*  n=37 2. No treatment (Standard) n=38 | 1. one capsule BID 2. NA | NR | Mixed |
| Wall 1994^170^ | Australia | 1-24 | Inpatients | 1. Lactose-free cow's milk infant formula containing corn syrup solids, as the carbohydrate (O-LAC, Mead Johnson) n=23 2. Low-lactose cow's milk infant formula containing maltodextrin, >95% of the lactose has been hydrolyzed to galactose and glucose (De-Lact infant, Sharpe lab) n=24 3. Standard cow's milk infant formula containing lactose (Enfalac, Mead Johnsons) n= 23 | NR | NR | Private |
| Widiasa 2009^171^ | Indonesia | 6-12 | Inpatients | 1. Smectite n=34 2. Placebo n= 34 | NR | NR | Private |
| Xu 2014^172^ | China | 6-24 | NR | 1. Zinc  n=38 2. *Glostridium Butyricum*  n=38 | 1. <1 year: 5 mL for children 1-2 year: 10 mL once daily 2. 0.5g each time, BID | NR | Not specified |
| Yazar 2016^173^ | Turkey | 6-120 | outpatients | 1. Symbiotic: Lactobacillus *casei, Lactobacillus rhamnosus, Lactobacillus plantarum plantarum, Bifidobacterium lactis* + fructose and galacto-oligosaccharides and polydextrose n=55 2. Zinc 15mg/day n=55 | 1. 4.5×10exp9 CFU 2. NA | 5 days | Not for profit |
| Yurdakok 2000^174^ | Turkey | 6-12 | Inpatients | 1. Vitamin A n= 57 2. Placebo n= 58 | 1. 100.000 IU single oral dose 2. NA | 1 day | Not specified |

CFU: Colony formed units; QD: Once a day; BID; Two times a day; TID: Three times a day; QID: Four times a day.

ORS: Ora rehydration solution; NA; Non-applicable; NA (when describing dose/freq of a placebo): Non-applicable, because no information provided for the placebo; NR: Not reported

**** Full reference list of included studies in page 94***

Table C: Risk of Bias of Included Studies **(N=174)**

| **Author & Year** | **Sequence generation** | **Allocation concealment** | **Blinding Participants and Patients** | **Blinding Outcome** | **Incomplete Outcome data** | **Selective outcome reporting** | **Other biases** | **Comparisons** |
| --- | --- | --- | --- | --- | --- | --- | --- | --- |
| Agustina, 2007 | Probably Low | Probably Low | Low | Probably Low | Low | Probably Low | Low | 25-11 |
| Al-Sonboli, 2003 | Probably Low | Probably High | Probably Low | Low | High | Low | Low | 4-17 |
| Allen, 1994 | Low | Probably Low | Low | Low | Probably Low | Low | Probably High | 11-17 |
| Anderson, 1984 | Probably Low | Probably High | Probably Low | Probably High | High | Low | Low | 3-17 |
| Anonymous, 1984 | Low | Low | Low | Low | Low | Probably Low | Low | 3-17 |
| Armitstead, 1989 | Probably High | Probably High | Probably Low | Probably High | Low | Low | Probably High | 11-14, 11-17, 14-17 |
| Awasthi, 2006 | High | Low | High | High | Low | Low | Low | 4-17 |
| Bahl, 2002 | Low | Low | Low | Low | Low | Probably Low | Low | 4-17 |
| Basu, 2007 | Low | Low | Low | Low | Low | Low | Probably Low | 7-17 |
| Basu, 2009 | Low | Low | Low | Probably Low | Low | Probably Low | Probably Low | 7-17 |
| Bhandari, 1997 | Probably High | Probably High | Probably Low | Probably Low | Low | Probably Low | Low | 5-17 |
| Bhatnagar, 1998 | Low | Probably High | Probably Low | Probably High | Low | Low | Low | 17-12 |
| Bhatnagar,2004 | Low | Low | Low | Low | Low | Low | Low | 18-17 |
| Billoo, 2006 | Probably Low | High | High | High | Low | Probably Low | Low | 8-17 |
| Boran, 2006 | Low | Probably High | High | High | Low | Probably Low | Low | 4-17 |
| Boudraa, 2001 | Probably High | Probably High | Low | Probably Low | Probably Low | Low | Low | 17-12 |
| Boulloche, 1994 | Low | Probably High | Probably Low | Probably High | Low | Probably Low | Low | 9-3, 3-17, 9-17 |
| Bowie, 1995 | Probably Low | Low | Low | Low | Probably Low | Probably Low | Low | 3-17 |
| Brown, 1991 | Low | Probably Low | Probably Low | Probably Low | Low | Low | Low | 11-17 |
| Burande, 2013 | Low | Probably High | Probably Low | Probably High | Low | Probably Low | Low | 21-4 |
| Canani, 2007 | Low | Low | Low | Low | Probably High | Probably Low | Low | 7-8,7-9,7-17,8-9,8-17,9-17 |
| Carague-Orendain, 1999 | Probably High | Probably High | Probably Low | Probably High | Probably High | Low | Low | 9-17 |
| Cetina-Sauri, 1994 | Low | Probably High | Probably Low | Probably High | Low | Low | Low | 8-17 |
| Cezard, 2001 | Probably High | High | Low | Probably Low | Probably Low | Low | Low | 1-17. |
| Chen 2010 | Low | Probably Low | Low | Low | Low | Probably Low | Low | 9-17 |
| Chew, 1993 | Low | Low | Low | Low | Low | Low | Low | 17-14 |
| Clemente-Yago, 1993 | Low | Probably High | Probably Low | Probably High | Low | Low | Low | 11-17 |
| Cojocaru, 2002 | High | High | High | High | Low | Low | Low | 1-17 |
| Conway, 1989 | Probably High | Probably High | Probably Low | Probably High | Low | Low | Low | 14-11, 14-17, 11-17 |
| Cordier, 1987 | High | High | Probably Low | Probably Low | Probably High | Low | Low | 3-17 |
| Correa, 2011 | Low | Probably Low | Low | Low | High | Probably Low | Low | 8-17 |
| Costa-Ribeiro, 2003 | Probably Low | Probably High | Low | Probably Low | Low | Probably High | Low | 7-17 |
| Crisinel, 2015 | Low | Probably Low | Low | Low | Low | Low | Low | 7-17 |
| Czerwionka-Szaflarska, 2009 | Probably High | Probably High | Probably Low | Probably High | Low | Low | Low | 4-17 |
| Dagan, 1984 | High | High | Probably Low | Probably High | Low | Low | Low | 11-17 |
| Dalgic, 2011 | Low | Probably High | Probably Low | Probably High | Low | Probably Low | Low | 8-4,8-11,8-21,8-22,8-23,8-24,8-17,4-11,4-21,4-22,4-23,4-24,4-17, 11-21,11-22,11-23,11-24,11-17,21-22,21-23,21-24,21-17,22-23,22-24,22-17,23-24,23-17,24-17 |
| Das, 2016 | Probably High | Probably High | Low | Probably Low | Probably Low | Low | Low | 8-17 |
| Dewan, 1995 | Probably High | High | Probably Low | Probably Low | Probably High | Probably Low | Low | 5-17 |
| Dinleyici, 2013 | Low | Probably Low | Probably Low | Probably High | Probably High | Low | Low | 10-17 |
| Dinleyici, 2015A | Low | Probably High | Probably Low | Probably High | Probably Low | Probably Low | Low | 9-17 |
| Dinleyici, 2015B | Low | Probably Low | High | Probably High | Low | Low | Low | 8-17 |
| Dinleyici,2014 | Low | Low | Low | Low | Probably High | Low | Low | 9-17 |
| Dugdale, 1982 | Probably High | Probably High | High | High | Low | Low | Low | 14-17 |
| Dupont, 2009a,b | Probably Low | Low | Low | Low | Low | Low | Low | 2-17 |
| Dutta, 2000 | Low | Low | Low | Low | Low | Probably Low | Low | 4-17 |
| Dutta, 2011A | Low | Low | Low | Low | Probably Low | Probably Low | Low | 4-18 |
| Dutta, 2011B | Low | Low | Low | Low | Low | Probably Low | Low | 9-17 |
| El-Soudi, 2015 | Probably High | Low | Probably Low | Probably Low | Low | Low | Low | 9-17 |
| Eren, 2010 | High | High | High | High | Probably Low | Probably Low | Low | 21-27 |
| Fajolu, 2008 | Probably Low | Probably High | Low | Low | Low | Low | Low | 4-17 |
| Fayad, 1999 | Low | Probably High | Probably Low | Probably High | Low | Low | Probably High | 17-11 |
| Francavilla, 2012 | Low | Probably High | Probably Low | Probably Low | Probably High | Low | Probably Low | 9-17 |
| Freedman, 2015 | Low | Low | Probably Low | Probably Low | Probably Low | Low | Low | 9-17 |
| Gharial, 2016 | Low | Probably Low | Probably Low | Probably Low | High | Low | Low | 1-4 |
| Gilbert, 1991 | Probably High | High | Probably Low | Probably High | Low | Probably High | Low | 2-17, 3-17, 2-3 |
| Grandy, 2010 | Probably Low | Probably Low | Probably Low | Probably Low | Low | Probably Low | Low | 9-8,8-17 |
| Grandy, 2012 | Probably High | Low | Low | Low | Probably Low | Low | Low | 12-8 |
| Groothuis, 1986 | High | Low | Low | Probably Low | Probably High | Low | Probably Low | 17-11 |
| Gu, 2011 | Probably Low | Probably High | Probably Low | Probably High | Probably High | High | Low | 9-2 |
| Guandalini, 2000 | Probably High | Probably High | Probably Low | Probably Low | Probably Low | Low | Probably Low | 7-17 |
| Guarino, 1997 | Low | Probably High | Probably Low | Probably High | Probably Low | Low | Probably Low | 7-17 |
| Guarino, 2001 | High | High | High | High | Low | Low | Low | 2-17 |
| Hafeez, 2002 | High | High | Probably Low | Probably High | Probably Low | Probably Low | Low | 8-17 |
| Haffejee, 1990 | High | Low | Probably Low | Probably High | Probably Low | Low | Low | 17-11 |
| Hegar, 2015 | Low | Low | Low | Low | Low | Low | Low | 16-4 |
| Henker, 2007 | Low | Low | Probably Low | Probably Low | Probably Low | Probably Low | Low | 9-17 |
| Henker, 2008 | Low | Probably Low | Low | Low | Low | Probably Low | Low | 9-17 |
| Henning, 1992 | Probably Low | Probably High | Probably Low | Probably Low | Probably Low | Probably Low | Low | 5-17 |
| Heydarian, 2010 | Low | Probably Low | Probably Low | Probably Low | Probably Low | Probably Low | Low | 12-17 |
| Hoekstra, 2004 | Low | Low | Low | Low | Probably Low | Probably Low | Low | 13-17 |
| Hoque, 2005 | Low | Low | Low | Low | High | Low | Low | 4-17 |
| Htwe, 2008 | High | High | High | High | High | Probably Low | Low | 8-17 |
| Huang, 2012 | Low | High | High | High | Low | Low | Low | 9-17 |
| Huang, 2014 | Low | Probably High | High | High | Low | High | Low | 9-17 |
| Islek, 2014 | Low | Probably Low | Probably Low | Probably Low | Low | Probably Low | Low | 10-17 |
| Isolauri, 1984 | Probably High | Probably High | Probably Low | Probably High | Low | Low | Low | 12-7 |
| Isolauri, 1986 | Probably High | Probably High | Probably Low | Probably High | Low | Low | Low | 7-17 |
| Isolauri, 1991 | Probably High | Probably High | Probably Low | Probably High | Low | Low | Probably Low | 11-17 |
| Jiang, 2016 | Probably High | Probably High | Probably Low | Probably High | Low | Low | Probably High | 4-17 |
| Kang, 2016 | Low | Probably Low | Low | Low | Probably Low | Low | Low | 1-17 |
| Kaplan, 1999 | Low | Low | Low | Low | Probably Low | Probably Low | Low | 3-17 |
| Karamyyar, 2013 | Low | Low | Low | Low | Low | Low | Low | 4-17 |
| Karrar, 1987 | High | Low | Low | Low | Low | Probably Low | Low | 3-3,3-17 |
| Kassem, 1983 | Probably High | Low | Low | Low | Low | Probably Low | Low | 3-17 |
| Khan, 2012 | Low | Probably High | High | High | Probably Low | Probably Low | Low | 8-17 |
| Khanna, 2005 | Probably Low | Low | Low | Low | Low | Probably Low | Probably High | 9-17 |
| Kianifar, 2009 | Low | Probably Low | Low | Low | High | Probably Low | Probably Low | 9-17 |
| Kowalska, 1999 | Probably High | Probably High | Probably Low | Probably Low | Probably High | Low | Low | 9-17 |
| Kurugol, 2005 | Probably Low | Probably Low | Low | Low | Low | Probably Low | Low | 8-17 |
| Leake 1974 | Low | Probably High | Low | Low | Low | Low | Low | 11-17 |
| Lee, 2015 | Probably Low | Probably High | Probably Low | Probably Low | Probably High | Low | Low | 9-17 |
| Lei, 2006 | Low | Probably High | High | High | Probably Low | Low | Low | 12-17 |
| Lexomboon, 1994 | Probably Low | Probably High | Probably Low | Probably High | Low | Low | Low | 2-17 |
| Lifshitz, 1991 | Low | Probably Low | Probably Low | Probably High | Low | Low | Low | 17-11 |
| Lopez-Hernandez, 1998 | Probably High | Probably High | Low | Probably Low | Low | Probably Low | Low | 8-17 |
| Lozano, 1994 | Low | Probably High | Probably Low | Probably High | Probably Low | Low | Low | 11-17 |
| Madkour, 1993 | Probably Low | Low | Low | Low | Probably Low | Low | Low | 2-17 |
| Majamaa, 1995 | Probably High | Probably High | Probably Low | Probably Low | Low | Low | Low | 7-9 |
| Manyal, 2015 | High | High | High | High | Low | Probably Low | Low | 16-4 |
| Mao, 2008 | Probably High | Probably High | Probably Low | Probably Low | Low | Low | Low | 11-20 |
| Maudgal, 1985 | High | High | High | High | Low | Low | Low | 14-17 |
| Melendez Garcia, 2007 | Low | High | High | High | Low | Low | Low | 1-19 |
| Michael, 2014 | High | High | High | High | Low | Low | Low | 1-17 |
| Milocco, 1999 | High | High | High | High | Probably High | High | Low | 2-17 |
| Misra, 2009 | Low | Probably High | Low | Low | Low | Low | Low | 7-17 |
| Moal, 2007 | Probably Low | Low | Low | Low | Low | Low | Low | 9-17 |
| Movahedi, 2008 | Probably High | Probably High | Probably Low | Probably Low | Low | Low | Low | 4-17 |
| Mujawar, 2012 | High | High | High | High | Low | Low | Low | 2-17 |
| Naidoo, 1981 | Probably High | Probably High | Probably Low | Probably High | Low | Low | Low | 11-17 |
| Narayanappa, 2008 | Probably High | Probably High | Probably Low | Probably Low | High | Low | High | 9-17 |
| Narkeviciute, 2002 | High | High | High | High | Probably Low | Probably Low | Low | 2-17 |
| Negi, 2015 | Low | Probably Low | Low | Low | Low | Low | Low | 4-17 |
| Nixon, 2012 | Low | Low | Low | Low | Probably Low | Low | Low | 7-17 |
| Noreen, 2016 | Probably High | High | High | High | Low | Low | High | 11-17 |
| Oandasan, 1999 | Low | Low | Probably Low | Probably Low | Low | Low | Low | 9-17 |
| Owens, 1981a,b | Probably Low | Probably High | Low | Low | Probably Low | Probably Low | Low | 3-17 |
| Ozkan, 2007 | Probably Low | Probably High | Low | Low | Probably Low | Probably Low | Low | 8-17 |
| Pant, 1996 | Probably Low | Probably High | Low | Low | Low | Low | Low | 7-17 |
| Pashapour, 2006 | Probably Low | Probably High | Probably Low | Probably Low | Probably Low | Probably Low | Low | 12-17 |
| Passariello, 2012 | Low | Probably High | Low | Low | Probably High | Low | Low | 10-17 |
| Patel, 2009 | Low | Low | Low | Low | Low | Low | Low | 4-17 |
| Patel, 2015 | Low | Probably High | High | High | Low | Low | Low | 4-17 |
| Patro, 2010 | Low | Low | Low | Low | Probably High | Probably Low | Low | 4-17 |
| Phavichitr, 2013 | Low | Low | Low | Low | Low | Low | Low | 9-17 |
| Pieacik-Lech, 2013 | Low | Probably Low | Low | Low | Probably High | Low | Low | 15-7 |
| Placzek, 1984 | High | High | High | High | Low | Low | Low | 17-14 |
| Pociecha, 1998a,b | Probably High | Probably High | Probably Low | Probably High | Low | Low | Low | 15-7 |
| Polat, 2003a,b | Low | Probably High | Low | Low | Probably High | Probably High | Probably Low | 4-17 |
| Quak, 1989 | Probably High | Probably High | Probably Low | Probably High | Low | Low | Low | 17-11 |
| Rafeey, 2008 | Low | Probably Low | Probably Low | Probably High | Low | Low | Low | 12-9 |
| Ransome, 1984 | Probably High | Probably High | Low | High | Low | Low | Low | 17-14 |
| Raza, 1995 | Probably Low | Probably Low | Low | Low | High | Low | Low | 7-17 |
| Rehman, 2013 | Probably Low | Probably High | Probably Low | Probably High | Probably High | Low | Low | 26-4 |
| Rerksuppaphol, 2010 | Low | Probably Low | Probably Low | Probably Low | Probably Low | Probably High | Low | 9-17 |
| Riaz, 2012 | Probably Low | Low | Low | Low | Low | Low | Low | 21-4 |
| Ritchie, 2010 | Low | Low | Low | Low | Low | Low | Low | 16-4 |
| Rosenfeldt, 2002A | Probably Low | Probably Low | Low | Probably Low | High | Low | Low | 9-17 |
| Rosenfeldt, 2002B | Probably Low | Probably Low | Low | Probably Low | Low | Low | Low | 9-17 |
| Roy, 1997 | Low | Low | Low | Low | Low | Probably Low | Low | 18-6 |
| Sachdev, 1988 | Probably High | High | Low | Low | Probably Low | Probably Low | Low | 4-17 |
| Salazar-Lindo, 2000 | Probably High | High | Low | Low | Probably Low | Low | Probably High | 7-17 |
| Salazar-Lindo, 2004 | Probably Low | Low | Low | Low | Probably Low | Low | Low | 1-17 |
| Saneian, 2012 | High | High | Probably Low | Probably High | Probably Low | Low | Low | 11-17 |
| Santos, 2009 | Low | High | High | High | Probably Low | Low | Probably Low | 1-17 |
| Sarker, 2005 | Low | Low | Low | Low | Low | Low | Low | 9-17 |
| Sazawal, 1995 | Low | Probably Low | Low | Low | Low | Probably Low | Low | 18-6 |
| Shan, 1997 | Probably Low | Probably High | Probably Low | Probably High | Probably Low | Probably High | Probably Low | 4-17 |
| Shamir, 2005 | Probably Low | Low | Low | Probably Low | Low | Low | Low | 16-17 |
| Shornikova, 1997A | Low | Probably Low | Low | Low | Low | Low | Low | 9-17 |
| Shornikova, 1997B | Probably High | Probably High | Low | Low | Low | Low | Low | 9-17 |
| Shornikova, 1997C | Probably Low | Probably High | Low | Low | Probably Low | Low | Low | 7-17 |
| Simakachorn, 2000 | Low | Low | Low | Low | Low | Low | Low | 9-17 |
| Simakachorn, 2004 | Low | Low | Low | Probably Low | Low | Low | Low | 11-17 |
| Sindhu, 2014 | Low | Probably Low | Low | Low | High | Low | Low | 7-17 |
| Strand, 2002 | Low | Low | Low | Low | Probably Low | Low | Low | 9-4,4-18,4-17,18-17 |
| Su, 2014 | Low | Probably High | Probably Low | Probably High | Probably High | High | Low | 4-11,11-23,4-23 |
| Sutton, 1968 | Probably High | Probably High | Probably Low | Probably High | Low | Low | Low | 11-17 |
| Szymanski, 2006 | Low | Low | Low | Low | Probably Low | Low | Low | 9-17 |
| Teran, 2009 | Low | Probably High | Probably Low | Probably High | Low | Low | Low | 17-9 |
| Torabi, 2011 | Probably Low | Probably Low | Probably Low | Probably Low | Probably Low | Low | Low | 4-17 |
| Torrez, 2013 | Probably High | Probably High | Probably Low | Probably High | Low | Low | Low | 1-17 |
| Touhami, 1989 | Probably High | Probably High | High | High | Probably Low | Probably Low | Low | 14-17 |
| Turck, 1999 | Probably High | Probably High | Low | Low | High | Low | Low | 1-3 |
| Upadhyay, 2014 | Low | Low | High | High | Low | Low | Low | 16-4 |
| Urganci, 2001 | Probably High | Probably High | Probably Low | Probably Low | Low | Probably Low | Low | 8-17 |
| Vandenplas, 2011 | Low | Low | Low | Low | Low | Low | Low | 10-17 |
| Villarruel, 2007 | Low | Low | Low | Probably Low | Probably Low | Probably Low | Low | 8-17 |
| Vivatvakin, 1992 | Probably High | High | High | High | Low | Low | Low | 2-17 |
| Vivatvakin, 2006 | Probably High | Probably High | Probably Low | Probably High | High | Probably High | Low | 9-17 |
| Wall, 1994 | Probably Low | Probably Low | Low | Probably Low | Probably High | Low | Low | 11-17 |
| Widiasa, 2009 | Probably Low | Low | Low | Low | Low | Low | Low | 2-17 |
| Xu, 2014 | Low | Probably High | Probably Low | Probably High | Low | High | Low | 16-9 |
| Yazar 2016 | Low | Probably Low | High | High | Low | Low | Low | 10-4,10-17,4-17 |

Some studies are labeled with letters in addition to the year and author names; Capital letters (e.g., A, B, C) denote different studies, same author. Lower Case letters (e.g., a, b, c) denote the same study but extracted double because they presented the results in subgroups, such as by age, or by country, or by setting (inpatients vs outpatients).

Last column describes the comparisons to which each study applies. Each number denotes a specific intervention which are described in Table 1 on manuscript.

# Table D: Descriptive transitivity analyses

| ***Outcome*** | **Mode_Year of Publication** | | **Mode_Country Income** | **Mean_Age** | **Mean_Diarrhea days** | **Mode_Patients Status** | **Mean_Etiology** |
| --- | --- | --- | --- | --- | --- | --- | --- |
| ***Diarrhea duration*** |  | |  |  |  |  |  |
| STND vs SYM | 2011, 2012. 2013, 2014, 2016 | | 1 | 31.502 | 0.84 | 2 | 0.34 |
| DM vs LCF | 1989 | | 2 | 6.43 | 5 | 1 | 0.225 |
| STND vs LCF | 1994 | | 1, 2 | 9.795 | 2.415 | 1 | 0.4763333 |
| LCF+PRB vs LCF | 2008 | | 1 | 13.07 | NR | 1 | 0.87 |
| SB+ZN vs LCF | 2011 | | 1 | 13.7 | 2.93 | 1 | 1 |
| SB+LCF vs LCF | 2011 | | 1 | 13.7 | 2.93 | 1 | 1 |
| ZN+LCF vs LCF | 2011, 2014 | | 1 | 13.69 | 1.465 | 1, 2 | 1 |
| SB+ZN+LCF vs LCF | 2011 | | 1 | 13.7 | 2.93 | 1 | 1 |
| SYM+LCF vs LCF | 2007 | | 1 | 8.05 | 1.85 | 1 | 0.75 |
| STND vs YOG | 2001 | | 1 | 8.1 | NR | 4 | NR |
| STND vs PRE | 2004 | | 3 | 12.5 | 2.1 | 1 | 0.21 |
| STND vs DM | 1989 | | 1 | 6.6 | 2.3 | 1 | 15.16075 |
| STND vs ZN+PRB | 2005 | | 2 | 9.28 | 2.37 | 1 | NR |
| ZN+MN vs STND | 2004, 2011 | | 1 | 12.075 | 1.67 | 1, 2 | 0.4095 |
| SB+ZN vs STND | 2011 | | 1 | 13.7 | 2.93 | 1 | 1 |
| SB+LCF vs STND | 2011 | | 1 | 13.7 | 2.93 | 1 | 1 |
| ZN+LCF vs STND | 2011 | | 1 | 13.7 | 2.93 | 1 | 1 |
| SB+ZN+LCF vs STND | 2011 | | 1 | 13.7 | 2.93 | 1 | 1 |
| STND vs RC | 2000,2009, 2013, 2014 | | 1 | 22.17 | 1.385 | 1 | 0.4453333 |
| LOP vs RC | 1999 | | 2 | 56.4 | 1.55 | 2 | 0.1 |
| ZN vs RC | 2016 | | 1 |  | NR | 1 | NR |
| SB+LCF vs SB+ZN | 2011 | | 1 | 13.7 | 2.93 | 1 | 1 |
| ZN+LCF vs SB+ZN | 2011 | | 1 | 13.7 | 2.93 | 1 | 1 |
| SB+ZN+LCF vs SB+ZN | 2011 | | 1 | 13.7 | 2.93 | 1 | 1 |
| ZN+LCF vs SB+LCF | 2011 | | 1 | 13.7 | 2.93 | 1 | 1 |
| SB+ZN+LCF vs SB+LCF | 2011 | | 1 | 13.7 | 2.93 | 1 | 1 |
| SB+ZN+LCF vs ZN+LCF | 2011 | | 1 | 13.7 | 2.93 | 1 | 1 |
| STND vs SM | 2009 | | 1 | 10.85 | 1.053333 | 1, 4 | 0.1804 |
| LOP vs SM | 1991 | | 2 | 11.16 | 1.46 | 4 | 0.277 |
| All-PRB vs SM | 1997, 2011 | | 1 |  | NR | 3, 4 | 1 |
| STND vs LOP | 1983, 1991, 1999 | | 1, 2, 3 | 26.37 | 0.4866667 | 2 | 0.219 |
| SYM vs ZN | 2016 | | 1 | 46.93 | NR | 2 | NR |
| LCF vs ZN | 2011, 2014 | | 1 | 13.69 | 1.465 | 1, 2 | 1 |
| ZN+PRB vs ZN | 2014, 2015 | | 1 | 20.8 | 1.515 | 2 | 0.241 |
| STND vs ZN | 2011 | | 1 | 22.18429 | 1.41125 | 1 | 0.345875 |
| ZN+MN vs ZN | 2011 | | 1 | 12.15 | 1.97 | 1 | 0.38 |
| SB+ZN vs ZN | 2011, 2012, 2013 | | 1 | 18.33 | 1.26 | 1 | 0.575 |
| SB+LCF vs ZN | 2011 | | 1 | 13.7 | 2.93 | 1 | 1 |
| ZN+LCF vs ZN | 2011, 2014 | | 1 | 13.69 | 1.465 | 1, 2 | 1 |
| SB+ZN+LCF vs ZN | 2011 | | 1 | 13.7 | 2.93 | 1 | 1 |
| LGG vs ZN | 2003 | | 1 | 15.35 | 1.72 | 4 | NR |
| SB vs ZN | 2011 | | 1 | 13.7 | 2.93 | 1 | 1 |
| STND vs VA | 1992, 1995, 2000 | | 1 | 13.58 | 1.643333 | 1 | 0.168 |
| ZN+MN vs MN | 1997 | | 1 | 11 | 2.7 | 4 | 0.25 |
| YOG vs LGG | 1991 | | 2 | 15.23 | 2.54 | 1 | 0.82 |
| LGG+SM vs LGG | 1998 | | 2 | 18.11 | 1.873333 | 3 | 0.875 |
| STND vs LGG | 1994,1996, 1997, 2000, 2003, 2007, 2009, 2012, 2014 | | 1 | 15.16917 | 2.118333 | 1 | 0.551 |
| SB vs LGG | 2007 | | 2 | 17.69 | 0.64 | 2 | NR |
| All-PRB vs LGG | 1995, 2007 | | 2 | 18.31 | 1.49 |  | 1 |
| LCF vs SB | 2011 | | 1 | 13.7 | 2.93 | 1 | 1 |
| STND vs SB | 2007 | | 1 | 19.50667 | 1.38125 | 1, 2, 4 | 0.7525 |
| SB+ZN vs SB | 2011 | | 1 | 13.7 | 2.93 | 1 | 1 |
| SB+LCF vs SB | 2011 | | 1 | 13.7 | 2.93 | 1 | 1 |
| ZN+LCF vs SB | 2011 | | 1 | 13.7 | 2.93 | 1 | 1 |
| SB+ZN+LCF vs SB | 2011 | | 1 | 13.7 | 2.93 | 1 | 1 |
| All-PRB vs SB | 2007, 2010 | | 1, 2 | 13.055 | 1.485 | 1, 2 | 1 |
| ZN+PRB vs All-PRB | 2014 | | 1 | 19.7 | NR | 3 | NR |
| STND vs All-PRB | 2015 | | 1 | 19.615 | 1.429032 | 1 | 0.5405455 |
| ***Diarrhea Day 3*** |  | |  |  |  |  |  |
| ZN+MN vs STND | 2002 | | 1 | 15.57 | 2.17 | 2 | NR |
| STND vs SM | 1992, 1993, 1994 | | 1 | 8.59 | 1.483333 | 1 | 0.181 |
| STND vs LOP | 1994 | | 1 | 12.4 | NR | 1 | 0.49 |
| All-PRB vs LOP | 1994 | | 1 | 12.4 | NR | 1 | 0.49 |
| ZN+PRB vs ZN | 2010 | | 1 | 8.91 | 3.93 | 1 | 0.14 |
| STND vs ZN | 2015 | | 1 | 29.32143 | 1.106667 | 4 | 0.014 |
| ZN+MN vs ZN | 2002 | | 1 | 15.57 | 2.17 | 2 | NR |
| SYM vs ZN | 2016 | | 1 | 46.93 | NR | 2 | NR |
| STND vs LGG | 1994, 2000 | | 1 | 13.15 | 2.625 | 2 | 0.85 |
| All-PRB vs LGG | 1997 | | 1 | 16.54 | 2.94 | 1 | 0.75 |
| STND vs SB | 1994, 1998, 2005, 2011, 2012, 2015 | | 1 | 24.355 | 0.7383333 | 4 | 0.702 |
| STND vs All-PRB | 1994, 1997, 1999, 2000, 2007, 2008, 2009, 2010, 2012, 2014, 2015 | | 1 | 17.605 | 1.525714 | 1 | 0.5388889 |
| STND vs SYM | 2011, 2012, 2013, 2016 | | 1 | 29.8775 | 0.675 | 2 | NR |
| STND vs LCF | 1981 | | 1 | 7.34 | NR | 1 | NR |
| STND vs YOG | 1998, 2001, 2010 | | 1 | 5.175 | 0.7233334 | 4 | 0.54 |
| ***Vomiting*** | |  |  |  |  |  |  |
| STND vs PRE | 2004 | | 1 | 12.5 | 2.1 | 1 | 0.21 |
| ZN+MN vs STND | 2004 | | 1 | 12 | 1.37 | 4 | 0.439 |
| STND vs RC | 2000 | | 1 | 12.5 | 2.04 | 1 | 0.5 |
| LOP vs RC | 1999 | | 1 | 56.4 | 1.55 | 2 | 0.1 |
| STND vs SM | 1992 | | 1 | 6.84 | 1.85 | 1 | 0.11 |
| STND vs LOP | 1983, 1984, 1999 | | 1 | 29.65 | NR | 2 | 0.19 |
| STND vs ZN | 2002, 2003, 2005, 2015 | | 1 | 28.144 | 1.175 | 4 | 0.014 |
| SB+ZN vs ZN | 2013 | | 1 | NR | NR | 2 | NR |
| ZN+MN vs MN | 1995 | | 1 | NR | 3.4 | 2 | NR |
| LGG+SM vs LGG | 2013 | | 1 | 17.73 | 1.46 | 1 | 0.625 |
| STND vs LGG | 2007 | | 1 | 17.69 | 0.64 | 2 | NR |
| SB vs LGG | 2007 | | 1 | 17.69 | 0.64 | 2 | NR |
| All-PRB vs LGG | 1997, 2007 | | 1 | 17.115 | 1.79 | 1, 2 | 0.75 |
| STND vs SB | 2007, 2010 | | 1 | 13.055 | 1.485 | 1, 2 | 1 |
| All-PRB vs SB | 2007, 2010 | | 1 | 13.055 | 1.485 | 1, 2 | 1 |
| STND vs All-PRB | 2007, 2010 | | 1 | 13.055 | 1.485 | 1, 2 | 1 |
| STND vs LCF | 2004 | | 1 | 12.17 | 2.39 | 1 | 0.5 |
| ***Any Side Effects*** |  | |  |  |  |  |  |
| STND vs RC | 2009 | | 2 | 12.01 | 2.1 | 2 | 0.236 |
| STND vs SM | 2009 | | 1 | 12.77667 | 0.3666667 | 4 | 0.2093333 |
| STND vs LOP | 1984, 1987, 1999 | | 2 | 27.67333 | 2.39 | 1 | 0.233 |
| ZN+MN vs ZN | 2002 | | 1 | 15.57 | 2.17 | 2 | NR |
| STND vs ZN | 2002, 2008, 2010 | | 1 | 18.55 | 1.44 | 2 | 0.431 |
| STND vs LGG | 2009, 2014 | | 1 | 15.82 | 2.73 | 1, 2 | 0.615 |
| STND vs SB | 2005 | | 1 | 42.6 | 1.55 | 1 | 0.83 |
| STND vs All-PRB | 2008, 2015 | | 2, 3 | 23.885 | 2.9 | 1, 3 | 0.5 |
| STND vs LCF | 1994 | | 2 | 7.41 | 3.26 | 4 | 0.082 |
| ZN+MN vs STND | 2002 | | 1 | 15.57 | 2.17 | 2 | NR |
| ***Stool Frequency at Day 2*** |  | |  |  |  |  |  |
| STND vs ZN+PRB | 2005 | | 1 | 9.28 | 2.37 | 1 | NR |
| STND vs RC | 2009, 2014 | | 1 | 27.005 | 1.75 | 2, 3 | 0.418 |
| ZN vs RC | 2016 | | 1 | NR | NR | 1 | NR |
| STND vs SM | 1999 | | 1 | 30.4 | 2.09 | 1 | 0.23 |
| STND vs ZN | 2003 | | 1 | 15.19 | 1.675 | 4 | NR |
| STND vs LGG | 1995, 1996, 2007, 2009 | | 1 | 13.6525 | 2.505 | 1 | 0.43075 |
| STND vs SB | 1994, 2001, 2007 | | 1 | 14.34667 | 0.9833333 | 3 | 1 |
| STND vs All-PRB | 1997, 2005, 2006, 2008, 2010, 2012 | | 1 | 23.94286 | 1.51125 | 1 | 0.6626667 |
| YOG vs All-PRB | 2008 | | 1 | 17.95 | 1.6 | 1 | NR |
| STND vs SYM | 2011, 2013 | | 1 | 26.07 | 1.175 | 1, 2 | NR |
| STND vs LCF | 1968, 1981, 1986, 2004 | | 1 | 9.826667 | 1.41 | 1 | 0.39 |
| STND vs YOG | 2001, 2006, 2008, 2010 | | 1 | 10.075 | 0.95 | 1 | 0.54 |

Variables are described in modes (categorical variables), and means (continuous variables). For Year of publication: Modes of Publication year; For Country income classification: (1) Low-and Middle-income country, (2) High-Income Country; (3) Both type of countries; For Age: Mean of months of age; For diarrhea days: Mean number of days with diarrhea among the children; For Patient Status: (1) Inpatients, (2) Outpatients; (3) recruited at the ED, not clear if hospitalized or not after the recruitment (4): Not stated/not reported; For aetiology: Mean proportion of children with infection by rotavirus.

Interventions abbreviations are described in table 1 of manuscript. NR: Not reported.

# Table E: Direct, indirect, and NMA estimates for diarrhea duration with the GRADE Assessment

| **#** | **Comparison** | **Direct Estimates**  MD (95%CrI) | **I^2^** | **Number of Studies** | **Number of**  **Patients** | **Direct GRADE** | **Indirect**  **Estimates**  MD (95%CrI) | **Indirect GRADE** | **Direct GRADE that informs Indirect** | **NMA estimate**  MD (95%CrI) | **NMA**  **GRADE** |
| --- | --- | --- | --- | --- | --- | --- | --- | --- | --- | --- | --- |
| **1** | **STND vs SYM** | **27.84 (17.3; 38.26)** | 24% | 5 | 692 | **High** | 2.04  (-83.33; 87.42) | Low^6^ | 34/37 | **26.26**  **(16.22;36.14)** | **High** |
| **2** | **DM vs LCF** | 15.09 (-2.3; 32.31) | -- | 1 | 150 | Very Low^1,2,4^ | 15.89  (-12.39; 44.16) | Very Low | 12/3 | **15.56**  **(2.92; 28.24)** | **Low** |
| **3** | **STND vs LCF** | **14.25 (7.39; 21.28)** | 65% | 14 | 1,343 | **Very low^1,2,5^** | -1.15  (-39.53; 37.23) | Moderate | 35/37 | **12.50**  **(5.99; 19.04)** | **Moderate** |
| **4** | **LCF+PRB vs LCF** | -0.83 (-10.11; 8.52) | -- | 1 | 212 | Low^1,4^ | 1.13  (-19.97; 21.94) | Low^1,4^ | -- | -0.77  (-22.49; 20.94) | Low^1,4^ |
| **5** | **SB+ZN vs LCF** | **-32.04 (-45.33;-18.92)** | -- | 1 | 120 | **Very low^1,1,4^** | -22.45  (-58.03; 13.13) | Very Low | 15/3 | **-26.84**  **(-41.02; -13.21)** | **Low** |
| **6** | **SB+LCF vs LCF** | 2.29 (-8.57; 13.36) | -- | 1 | 120 | Very low1^,1,4^ | -0.53  (-66.71; 65.64) | Very Low | 16/3 | 0.14  (-18.10; 18.87) | Low^4^ |
| **7** | **ZN+LCF vs LCF** | -10.94 (-28.89; 7.14) | 94% | 2 | 180 | **Very low^1,2,3,4^** | -0.61  (-60.57; 59.35) | Very Low | 17/3 | -8.91  (-24.52; 6.93) | Very Low^4^ |
| **8** | **SB+ZN+LCF vs LCF** | -2.31 (-15.64; 11.35) | -- | 1 | 120 | Very low^1,1,4^ | -5.17  (-73.61, 63.28) | Very Low | 18/3 | -4.22  (-24.04; 15.67) | Very Low^4^ |
| **9** | **SYM+LCF vs LCF** | **-19.66 (-23.07;-16.27)** | -- | 1 | 58 | High | -19.67  (-39.34; 0.25) | Moderate | -- | -19.67  (-39.34; 0.25) | Moderate |
| **10** | **STND vs YOG** | **17.79 (5.14; 30.33)** | -- | 1 | 112 | Very low^1,1,4^ | 9.63  (-39.77; 59.03) | Low | 50/48 | **16.43**  **(2.05; 30.49)** | **Very Low^4^** |
| **11** | **STND vs PRE** | 14.73 (-4.58; 34.37) | -- | 1 | 144 | Moderate^4^ | 15.62  (-11.28; 42.42) | Moderate^4^ | -- | 15.32  (-12.03; 42.82) | Moderate^4^ |
| **12** | **STND vs DM** | -2.46 (-14.02; 9.24) | 66% | 5 | 548 | Low^1,2^ | -5.48  (-97.65; 86.68) | Very Low | 2/3 | -3.02  (-14.32; 8.41) | Very Low^4^ |
| **13** | **STND vs ZN+PRB** | **15.03 (3.19; 26.96)** | -- | 1 | 65 | Very low^1,1,4^ | **33.17**  **(3.06; 63.29)** | Low | 36/37 | **29.39**  **(18.57; 40.26)** | **Low** |
| **14** | **ZN+MN vs STND** | **-17.82 (-33.15; -2.5)** | 75% | 2 | 392 | Moderate^2^ | -14.42  (-97.85; 69.01) | Moderate | 38/37 | **-17.76**  **(-31.77; -4.13)** | **Moderate** |
| **15** | **SB+ZN vs STND** | **-52.94 (-68.34;-37.33)** | -- | 1 | 120 | **Very low^1,1,4^** | **-35.08**  **(-66.65; -3.50**) | Moderate | 39/37 | **-39.45**  **(-52.45;-26.73)** | **Moderate** |
| **16** | **SB+LCF vs STND** | **-18.68 (-32.21; -5.2)** | -- | 1 | 120 | Very low^1,1,4^ | -4.40  (-64.52; 55.71) | Very Low^6^ | 57/55 | -12.32  (-30.01; 5.98) | **Very Low^4^** |
| **17** | **ZN+LCF vs STND** | **-13.95 (-27.53; -0.33)** | -- | 1 | 120 | Very low^1,1,4^ | -35.15  (-72.57; 2.27) | Very Low | 41/37 | **-21.37**  **(-36.54; -6.13)** | **Moderate** |
| **18** | **SB+ZN+LCF vs STND** | **-23.18 (-38.8; -7.75)** | -- | 1 | 120 | Very low^1,1,4^ | -8.57  (-70.78; 53.64) | Low^6^ | 42/37 | -16.74  (-36.05; 2.71) | Low4 |
| **19** | **STND vs RC** | **20.18 (11.79; 28.69)** | 79% | 7 | 883 | **Low^1,2^** | 2.49  (-34.15; 39.14) | Very Low^6^ | 22/37 | **17.19**  **(9.75; 24.65)** | **Low** |
| **20** | **CAO vs RC** | 12.02 (-7.21; 31.06) | -- | 1 | 50 | **Very low^1,4,4^** | 11.86  (-14.87; 38.52) | Very Low^1,4,4^ | -- | 11.82  (-15.27; 38.96) | Very Low^1,4,4^ |
| **21** | **LOP vs RC** | -1.9 (-7.51; 3.76) | -- | 1 | 97 | Low^1,4^ | -1.86  (-35.39; 31.39) | Very Low^6^ | 33/19 | -1.61  (-14.62; 11.66) | Low^4^ |
| **22** | **ZN vs RC** | -22.77 (-50.24; 5.22) | -- | 1 | 120 | Moderate^1^ | 0.05  (-21.20; 21.30) | Low | 19/37 | -1.23  (-9.94; 7.54) | Low^4^ |
| **23** | **SB+LCF vs SB+ZN** | **34.49 (20.91; 48.37)** | -- | 1 | 120 | **Very low^1,1,4^** | 2.19  (-86.96; 91.34) | Very Low^6^ | 40/39 | **27.06**  **(6.96; 47.87)** | **Very Low^4^** |
| **24** | **ZN+LCF vs SB+ZN** | **39.41 (25.52; 53.12)** | -- | 1 | 120 | **Very low^1,1,4^** | -21.74  (-77.68; 34.19) | Very Low | 41/39 | 18.03  -0.21; 36.08) | Very Low^4^ |
| **25** | **SB+ZN+LCF vs SB+ZN** | **30.04 (14.42; 45.49)** | -- | 1 | 120 | **Very low^1,1,4^** | -3.23  (-95.09; 88.63) | Low^6^ | 42/39 | **22.78**  **(0.99; 44.59)** | **Very Low^4^** |
| **26** | **YOG+PRB+ZNvsSB+ZN** | 22.98 (-12.74; 58.13) | -- | 1 | 55 | **Very low^1,1,4^** | NA | NA | -- | 23.90  (-15.92; 63.55) | Very Low^1,1,4^ |
| **27** | **ZN+LCF vs SB+LCF** | 5.04 (-6.39; 16.47) | -- | 1 | 120 | Very low^1,1,4^ | -108.46  (-215.69; -1.22) | Very Low | 41/40 | -9.06  (-30.33; 12.02) | Very Low^4^ |
| **28** | **SB+ZN+LCF vs SB+LCF** | -4.39 (-18.02; 9.29) | -- | 1 | 120 | Very low^1,1,4^ | NA | NA | 42/40 | -4.40  (-28.40; 19.48) | Very Low^4^ |
| **29** | **SB+ZN+LCF vs ZN+LCF** | -9.12 (-23.16; 4.79) | -- | 1 | 120 | Very low^1,1,4^ | 106.90  (-3.5; 217.3) | Very Low | 42/41 | 4.70  (-17.99;27.37) | Very Low^4^ |
| **30** | **STND vs SM** | **23.41 (15.53; 31.15)** | 96% | 9 | 1,767 | **Very low^1,1,2,5^** | 19.79  (-13.48; 53.06) | Very Low | 62/32 | **23.90**  **(16.96;30.80)** | **Very Low** |
| **31** | **LOP vs SM** | 4.31 (-22.33; 31.19) | -- | 1 | 32 | Very low^1,4^ | 11.32  (-22.39; 45.03) | Very Low | 33/30 | 6.15  (-7.77; 19.91) | Low^4^ |
| **32** | **All-PRB vs SM** | 9.49 (-11.35; 30.14) | 98% | 2 | 190 | Very low^1,2,2^ | 6.49  (-15.16; 28.14) | Very Low | 62/30 | 4.58  (-3.18; 12.19) | Very Low^4^ |
| **33** | **STND vs LOP** | **16.55 (1.18; 31.64)** | 0% | 4 | 423 | **Moderate^1^** | 19.12  (-25.33; 63.57) | Very Low | 31/30 | **17.79**  **(5.65; 30.35)** | **Moderate** |
| **34** | **SYM vs ZN** | 4.75 (-6.32; 15.89) | -- | 1 | 110 | Moderate^1^ | 11.20  (-14.74; 37.14) | Low^6^ | 1/37 | -7.89  (-18.75; 3.11) | Low^4^ |
| **35** | **LCF vs ZN** | 18.04 (-0.04; 36.46) | 96% | 2 | 180 | Very Low^1,1,2,4^ | 0.34  (-17.89; 18.58) | Very Low | 3/37 | 5.85  (-2.07;13.88) | Very Low^4^ |
| **36** | **ZN+PRB vs ZN** | -11.27 (-25.72; 2.74) | 51% | 3 | 374 | Very low^1,2,4^ | -11.13  (-46.64; 24.39) | Very Low^6^ | 13/37 | **-10.96**  **(-21.78; -0.37)** | **Low** |
| **37** | **STND vs ZN** | **15.19 (9.4; 20.9)** | 90% | 17 | 3,871 | **Moderate^2^** | 28.64  (6.49; 50.79) | Very Low^6^ | 3/35 | **18.38**  **(13.45;23.39)** | **Moderate** |
| **38** | **ZN+MN vs ZN** | -2.54 (-10.26; 4.97) | -- | 1 | 124 | Moderate^4^ | 3.33  (39.76; 46.43) | Moderate | 14/37 | 0.64  (-13.79; 14.79) | Moderate^4^ |
| **39** | **SB+ZN vs ZN** | -12.44 (-25.86; 0.72) | 0% | 3 | 298 | Low^1,4^ | -79.80  (-146.99;-12.61) | Low | 15/37 | **-21.05**  **(-33.72; -8.64)** | **Moderate** |
| **40** | **SB+LCF vs ZN** | **27.43 (15.87; 39.28)** | -- | 1 | 120 | Very low^1,1,4^ | -34.09  (-96.58; 28.40) | Very Low^6^ | 16/37 | 6.05  (-11.95;24.37) | Very Low^4^ |
| **41** | **ZN+LCF vs ZN** | 6.23 (-12.03; 24.68) | 97% | 2 | 180 | **Very low^1,1,2,2^** | 27.07  (-28.30; 82.44) | Very Low^6^ | 17/37 | -2.97  (-18.20: 12.20) | Very Low^4^ |
| **42** | **SB+ZN+LCF vs ZN** | **22.89 (8.94; 36.72)** | -- | 1 | 120 | Very low^1,1,4^ | -40.30  (-104.97; 24.36) | Very Low^6^ | 18/37 | 1.61  (-17.78;21.28) | Low4 |
| **43** | **SM+ZN vs ZN** | **-17.49 (-26.75; -8.28)** | -- | 1 | 196 | High | -17.28  (-38.75; 3.78) | High | -- | -17.26  (-38.56; 4.50) | High |
| **44** | **LGG vs ZN** | 12.23 (-1.39; 25.99) | -- | 1 | 106 | Moderate^4^ | -5.83  (-22.62; 10.97) | Low | 50/37 | -4.37  (-12.07; 3.25) | Moderate^4^ |
| **45** | **SB vs ZN** | **32.96 (20.94; 45.18)** | -- | 1 | 120 | **Very low^1,1,4^** | -0.95  (-20.36; 18.45) | Low | 55/37 | 1.87  (-6.30; 10.03) | Very Low^4^ |
| **46** | **STND vs VA** | 5.82 (-9.52; 21.25) | 0% | 3 | 374 | Low^1,4^ | 6.11  (-9.19; 21.42) | Low^1,4^ | -- | 5.95  (-9.32; 21.43) | Low^1,4^ |
| **47** | **ZN+MN vs MN** | -18.57 (-41.57; 4.53) | -- | 1 | 74 | Moderate^4^ | -18.09  (-47.36; 11.08) | Moderate^4^ | -- | -18.34  (-48.25; 11.70) | Moderate^4^ |
| **48** | **YOG vs LGG** | **11.95 (1.05; 22.76)** | -- | 1 | 71 | Very low^1,1,4^ | 4.03  (-45.86; 53.92) | Low | 10/50 | 6.27  (-8.03; 20.86) | Very Low^4^ |
| **49** | **LGG+SM vs LGG** | **-28.49 (-40.13;-16.76)** | 99% | 3 | 153 | **Low^1,2^** | **-28.4**  **(-39.67; -17.0)** | **Low^1,2^** | -- | **-28.28**  **(-39.90; -16.62)** | **Low^1,2^** |
| **50** | **STND vs LGG** | **22.08 (15.29; 28.65)** | 99% | 12 | 2,552 | **Low^1,2^** | 24.59  (-4.24; 53.42) | Very Low | 52/62 | **22.74**  **(16.68; 28.81)** | **Low** |
| **51** | **SB vs LGG** | **26.53 (19.31; 33.85)** | -- | 1 | 191 | Moderate^4^ | 3.92  (-17.21; 25.04) | Low | 55/50 | 6.24  (-2.46; 14.93) | Moderate^4^ |
| **52** | **All-PRB vs LGG** | **16.82 (0.36; 33.09)** | 88% | 2 | 438 | Very low^1,1,2^ | -2.15  (-18.40; 14.10) | Low | 62/50 | 3.35  (-3.82; 10.60) | Very Low^4^ |
| **53** | **LCF vs SB** | -7.44 (-18.99; 4.11) | -- | 1 | 120 | Very low^1,1,4^ | 1.07  (-20.67; 22.82) | Very Low | 3/55 | -3.98  (-5.22; 13.19) | Very Low^4^ |
| **54** | **YOG vs SB** | -7.07 (-26.72; 12.71) | -- | 1 | 42 | Very low^1,1,4^ | 0.42  (-14.39; 15.04) | Very Low^6^ | 10/55 | -0.01  (-14.65; 15.29) | Very Low^4^ |
| **55** | **STND vs SB** | **19.66 (12.07; 26.93)** | 71% | 11 | 1,774 | **Low^1,2^** | 3.29  (-36.55; 43.14) | Very Low^6^ | 60/62 | **16.48**  **(9.69; 23.30)** | **Low** |
| **56** | **SB+ZN vs SB** | **-39.62 (-53.69;-25.49)** | -- | 1 | 120 | **Very low^1,1,4^** | -18.27  (-55.58; 19.04) | Very Low^6^ | 15/55 | **-22.95**  **(-37.38;-8.99)** | **Low** |
| **57** | **SB+LCF vs SB** | -5.31 (-17.36; 6.62) | -- | 1 | 120 | Very low^1,1,4^ | 13.80  (54.87; 82.47) | Very Low^6^ | 16/55 | 4.19  (-14.15; 23.12) | Very Low^4^ |
| **58** | **ZN+LCF vs SB** | -0.56 (-12.52; 11.52) | -- | 1 | 120 | Very low^1,1,4^ | -23.36  (-66.67; 19.96) | Very Low^6^ | 17/55 | -4.94  (-20.97;11.17) | Very Low^4^ |
| **59** | **SB+ZN+LCF vs SB** | -9.77 (-24.26; 4.57) | -- | 1 | 120 | Very low^1,1,4^ | 9.76  (-61.21; 80.72) | Very Low^6^ | 18/55 | -0.26  (-20.06; 19.93) | Very Low^4^ |
| **60** | **All-PRB vs SB** | -1.67 (-17.82; 14.28) | 97% | 2 | 424 | Low^1,2^ | -8.35  (-27.82; 11.11) | Low | 62/55 | -2.88  (-10.57; 4.83) | Very Low^4^ |
| **61** | **ZN+PRB vs All-PRB** | **-21.56 (-29.18;-13.82)** | -- | 1 | 76 | Moderate^1^ | -0.94  (-32.26; 30.38) | Very Low^6^ | 13/62 | -9.97  (-21.01; 1.10) | Low^4^ |
| **62** | **STND vs All-PRB** | **20.68 (16.08; 25.27)** | 89% | 31 | 3,375 | **Very low^1,2,4^** | 13.60  (-11.07; 38.27) | Very Low | 50/52 | **19.36**  **(15.09; 23.66)** | **Low** |
| **63** | **SM vs RC** | NA | NA | NA | NA | NA | -6.74  (-16.83; 3.44) | Very Low^4^ | 30/19/ | -6.74  (-16.83; 3.44) | Very Low^4^ |
| **64** | **ZN vs SM** | NA | NA | NA | NA | NA | 5.56  (-3.06; 14) | Very Low^4^ | 30/37/ | 5.56  (-3.06; 14) | Very Low^4^ |
| **65** | **ZN vs LOP** | NA | NA | NA | NA | NA | -0.6  (-13.7; 12.74) | Very Low^4,6^ | 33/37/ | -0.6  (-13.7; 12.74) | Very Low^4,6^ |
| **66** | **VA vs RC** | NA | NA | NA | NA | NA | 11.19  (-6.1; 28.33) | Very Low^4^ | 19/46/ | 11.19  (-6.1; 28.33) | Very Low^4^ |
| **67** | **VA vs SM** | NA | NA | NA | NA | NA | **17.9  (0.86; 34.7)** | **Very Low** | 30/46/ | **17.9  (0.86; 34.7)** | **Very Low** |
| **68** | **VA vs LOP** | NA | NA | NA | NA | NA | 11.8  (-7.67; 31.81) | Low4 | 33/46/ | 11.8  (-7.67; 31.81) | Low4 |
| **69** | **VA vs ZN** | NA | NA | NA | NA | NA | 12.38  (-3.78; 28.26) | Low4 | 37/46/ | 12.38  (-3.78; 28.26) | Low4 |
| **70** | **MN vs RC** | NA | NA | NA | NA | NA | 17.76  (-15.93; 51.1) | Very Low^4^ | 19/14/47 | 17.76  (-15.93; 51.1) | Very Low^4^ |
| **71** | **MN vs SM** | NA | NA | NA | NA | NA | 24.55  (-9.58; 57.77) | Very Low^4^ | 30/14/47 | 24.55  (-9.58; 57.77) | Very Low^4^ |
| **72** | **MN vs LOP** | NA | NA | NA | NA | NA | 18.3  (-16.94; 53.58) | Very Low^4,6^ | 33/14/47 | 18.3  (-16.94; 53.58) | Very Low^4,6^ |
| **73** | **MN vs ZN** | NA | NA | NA | NA | NA | 18.99  (-14.33; 51.87) | Low4 | 37/14/47 | 18.99  (-14.33; 51.87) | Low4 |
| **74** | **MN vs VA** | NA | NA | NA | NA | NA | 6.68  (-30.36; 42.93) | Very Low^4,6^ | 46/14/47 | 6.68  (-30.36; 42.93) | Very Low^4,6^ |
| **75** | **LGG vs RC** | NA | NA | NA | NA | NA | -5.6  (-15.1; 4.1) | Very Low^4^ | 19/50/ | -5.6  (-15.1; 4.1) | Very Low^4^ |
| **76** | **LGG vs SM** | NA | NA | NA | NA | NA | 1.16  (-8.03; 10.52) | Very Low^4^ | 30/50/ | 1.16  (-8.03; 10.52) | Very Low^4^ |
| **77** | **LGG vs LOP** | NA | NA | NA | NA | NA | -5.0  (-18.58; 8.81) | Very Low^4,6^ | 33/50/ | -5.0  (-18.58; 8.81) | Very Low^4,6^ |
| **78** | **LGG vs VA** | NA | NA | NA | NA | NA | **-16.79  (-33.08; -0.33)** | **Very Low^6^** | 46/50/ | **-16.79  (-33.08; -0.33)** | **Very Low^6^** |
| **79** | **LGG vs MN** | NA | NA | NA | NA | NA | -23.36  (-56.29; 10.68) | Very Low^4^ | 14/50/47 | -23.36  (-56.29; 10.68) | Very Low^4^ |
| **80** | **SB vs RC** | NA | NA | NA | NA | NA | 0.65  (-9.37; 10.67) | Very Low^4^ | 19/55/ | 0.65  (-9.37; 10.67) | Very Low^4^ |
| **81** | **SB vs SM** | NA | NA | NA | NA | NA | 7.42  (-2.19; 16.94) | Very Low^4^ | 30/55/ | 7.42  (-2.19; 16.94) | Very Low^4^ |
| **82** | **SB vs LOP** | NA | NA | NA | NA | NA | 1.3  (-12.59; 15.41) | Very Low^4,6^ | 33/55/ | 1.3  (-12.59; 15.41) | Very Low^4,6^ |
| **83** | **SB vs VA** | NA | NA | NA | NA | NA | -10.45  (-27.15; 6.12) | Very Low^4^ | 46/55/ | -10.45  (-27.15; 6.12) | Very Low^4^ |
| **84** | **SB vs MN** | NA | NA | NA | NA | NA | -17.14  (-50.03; 16.83) | Very Low^4^ | 14/55/47 | -17.14  (-50.03; 16.83) | Very Low^4^ |
| **85** | **All-PRB vs RC** | NA | NA | NA | NA | NA | -2.18  (-10.66; 6.32) | Very Low^4^ | 19/62/ | -2.18  (-10.66; 6.32) | Very Low^4^ |
| **86** | **All-PRB vs LOP** | NA | NA | NA | NA | NA | -1.62  (-14.56; 11.56) | Very Low^4^ | 33/62/ | -1.62  (-14.56; 11.56) | Very Low^4^ |
| **87** | **All-PRB vs ZN** | NA | NA | NA | NA | NA | -0.97  (-7.45; 5.54) | Very Low^4^ | 37/62/ | -0.97  (-7.45; 5.54) | Very Low^4^ |
| **88** | **All-PRB vs VA** | NA | NA | NA | NA | NA | -13.34  (-29.28; 2.6) | Very Low^4,6^ | 46/62/ | -13.34  (-29.28; 2.6) | Very Low^4,6^ |
| **89** | **All-PRB vs MN** | NA | NA | NA | NA | NA | -20.02  (-52.98; 13.81) | Very Low^4^ | 14/62/47 | -20.02  (-52.98; 13.81) | Very Low^4^ |
| **90** | **SYM vs RC** | NA | NA | NA | NA | NA | -9.08  (-21.52; 3.45) | Very Low^4,6^ | 19/1/ | -9.08  (-21.52; 3.45) | Very Low^4,6^ |
| **91** | **SYM vs SM** | NA | NA | NA | NA | NA | -2.36  (-14.34; 9.87) | Very Low^4^ | 30/1/ | -2.36  (-14.34; 9.87) | Very Low^4^ |
| **92** | **SYM vs LOP** | NA | NA | NA | NA | NA | -8.39  (-24.2; 7.42) | Very Low^4,6^ | 33/1/ | -8.39  (-24.2; 7.42) | Very Low^4,6^ |
| **93** | **SYM vs VA** | NA | NA | NA | NA | NA | **-20.26  (-38.26; -1.87)** | **Low^6^** | 46/1/ | **-20.26  (-38.26; -1.87)** | **Low^6^** |
| **94** | **SYM vs MN** | NA | NA | NA | NA | NA | -26.86  (-60.64; 7.77) | Very Low^4,6^ | 14/1/47 | -26.86  (-60.64; 7.77) | Very Low^4,6^ |
| **95** | **SYM vs LGG** | NA | NA | NA | NA | NA | -3.52  (-14.99; 8.15) | Very Low^4,6^ | 50/1/ | -3.52  (-14.99; 8.15) | Very Low^4,6^ |
| **96** | **SYM vs SB** | NA | NA | NA | NA | NA | -9.81  (-21.85; 2.35) | Very Low^4,6^ | 55/1/ | -9.81  (-21.85; 2.35) | Very Low^4,6^ |
| **97** | **SYM vs All-PRB** | NA | NA | NA | NA | NA | -6.87  (-17.55; 4.09) | Very Low^4,6^ | 62/1/ | -6.87  (-17.55; 4.09) | Very Low^4,6^ |
| **98** | **LCF vs RC** | NA | NA | NA | NA | NA | 4.71  (-5.15; 14.63) | Very Low^4^ | 19/3/ | 4.71  (-5.15; 14.63) | Very Low^4^ |
| **99** | **LCF vs SM** | NA | NA | NA | NA | NA | **11.44  (1.7; 20.89)** | **Very Low** | 30/3/ | **11.44  (1.7; 20.89)** | **Very Low** |
| **100** | **LCF vs LOP** | NA | NA | NA | NA | NA | 5.35  (-8.61; 19.31) | Very Low^4^ | 33/3/ | 5.35  (-8.61; 19.31) | Very Low^4^ |
| **101** | **LCF vs VA** | NA | NA | NA | NA | NA | -6.52  (-23.1; 10.12) | Very Low^4^ | 46/3/ | -6.52  (-23.1; 10.12) | Very Low^4^ |
| **102** | **LCF vs MN** | NA | NA | NA | NA | NA | -13.2  (-46.25; 20.86) | Very Low^4^ | 14/3/47 | -13.2  (-46.25; 20.86) | Very Low^4^ |
| **103** | **LCF vs LGG** | NA | NA | NA | NA | NA | **10.22  (1.31; 19.2)** | **Very Low** | 50/3/ | **10.22  (1.31; 19.2)** | **Very Low** |
| **104** | **LCF vs All-PRB** | NA | NA | NA | NA | NA | 6.88  (-0.94; 14.5) | Very Low^4^ | 62/3/ | 6.88  (-0.94; 14.5) | Very Low^4^ |
| **105** | **LCF vs SYM** | NA | NA | NA | NA | NA | **13.79  (1.81; 25.53)** | **Very Low** | 1/3/ | **13.79  (1.81; 25.53)** | **Very Low** |
| **106** | **YOG vs RC** | NA | NA | NA | NA | NA | 0.74  (-15.2; 16.86) | Very Low^4^ | 19/10/ | 0.74  (-15.2; 16.86) | Very Low^4^ |
| **107** | **YOG vs SM** | NA | NA | NA | NA | NA | 7.4  (-8.42; 23.36) | Very Low^4^ | 30/10/ | 7.4  (-8.42; 23.36) | Very Low^4^ |
| **108** | **YOG vs LOP** | NA | NA | NA | NA | NA | 1.38  (-17.21; 20.54) | Very Low^4^ | 33/10/ | 1.38  (-17.21; 20.54) | Very Low^4^ |
| **109** | **YOG vs ZN** | NA | NA | NA | NA | NA | 1.87  (-12.8; 17.14) | Very Low^4^ | 37/10/ | 1.87  (-12.8; 17.14) | Very Low^4^ |
| **110** | **YOG vs VA** | NA | NA | NA | NA | NA | -10.4  (-31.45; 10.17) | Very Low^4,6^ | 46/10/ | -10.4  (-31.45; 10.17) | Very Low^4,6^ |
| **111** | **YOG vs MN** | NA | NA | NA | NA | NA | -17.05  (-52.48; 19.34) | Very Low^4^ | 14/10/47 | -17.05  (-52.48; 19.34) | Very Low^4^ |
| **112** | **YOG vs All-PRB** | NA | NA | NA | NA | NA | 2.93  (-11.65; 17.94) | Very Low^4,6^ | 62/10/ | 2.93  (-11.65; 17.94) | Very Low^4,6^ |
| **113** | **YOG vs SYM** | NA | NA | NA | NA | NA | 9.81  (-7.7; 27.35) | Very Low^4,6^ | 1/10/ | 9.81  (-7.7; 27.35) | Very Low^4,6^ |
| **114** | **YOG vs LCF** | NA | NA | NA | NA | NA | -4  (-19.42; 11.98) | Very Low^4^ | 3/10/ | -4  (-19.42; 11.98) | Very Low^4^ |
| **115** | **PRE vs RC** | NA | NA | NA | NA | NA | 2.03  (-26.38; 30.1) | Very Low^4^ | 19/11/ | 2.03  (-26.38; 30.1) | Very Low^4^ |
| **116** | **PRE vs SM** | NA | NA | NA | NA | NA | 8.61  (-19.82; 36.73) | Very Low^4^ | 30/11/ | 8.61  (-19.82; 36.73) | Very Low^4^ |
| **117** | **PRE vs LOP** | NA | NA | NA | NA | NA | 2.4  (-27.26; 32.83) | Very Low^4,6^ | 33/11/ | 2.4  (-27.26; 32.83) | Very Low^4,6^ |
| **118** | **PRE vs ZN** | NA | NA | NA | NA | NA | 3.15  (-24.74; 30.79) | Very Low^4,6^ | 37/11/ | 3.15  (-24.74; 30.79) | Very Low^4,6^ |
| **119** | **PRE vs VA** | NA | NA | NA | NA | NA | -9.23  (-40.53; 22.12) | Very Low^4,6^ | 46/11/ | -9.23  (-40.53; 22.12) | Very Low^4,6^ |
| **120** | **PRE vs MN** | NA | NA | NA | NA | NA | -15.71  (-57.97; 27.23) | Low4 | 14/11/47 | -15.71  (-57.97; 27.23) | Low4 |
| **121** | **PRE vs LGG** | NA | NA | NA | NA | NA | 7.4  (-20.6; 35.52) | Very Low^4^ | 50/11/ | 7.4  (-20.6; 35.52) | Very Low^4^ |
| **122** | **PRE vs SB** | NA | NA | NA | NA | NA | 1.19  (-27.03; 29.29) | Very Low^4,6^ | 55/11/ | 1.19  (-27.03; 29.29) | Very Low^4,6^ |
| **123** | **PRE vs All-PRB** | NA | NA | NA | NA | NA | 4.12  (-23.8; 31.85) | Very Low^4^ | 62/11/ | 4.12  (-23.8; 31.85) | Very Low^4^ |
| **124** | **PRE vs SYM** | NA | NA | NA | NA | NA | 11.07  (-18.51; 40.11) | Low4,6 | 1/11/ | 11.07  (-18.51; 40.11) | Low4,6 |
| **125** | **PRE vs LCF** | NA | NA | NA | NA | NA | -2.77  (-31.01; 25.33) | Very Low^4^ | 3/11/ | -2.77  (-31.01; 25.33) | Very Low^4^ |
| **126** | **PRE vs YOG** | NA | NA | NA | NA | NA | 1.22  (-29.13; 31.72) | Very Low^4^ | 10/11/ | 1.22  (-29.13; 31.72) | Very Low^4^ |
| **127** | **DM vs RC** | NA | NA | NA | NA | NA | **20.27  (6.58; 33.63)** | **Very Low^6^** | 19/12/ | **20.27  (6.58; 33.63)** | **Very Low^6^** |
| **128** | **DM vs SM** | NA | NA | NA | NA | NA | **26.93  (13.64; 40.35)** | **Very Low** | 30/12/ | **26.93  (13.64; 40.35)** | **Very Low** |
| **129** | **DM vs LOP** | NA | NA | NA | NA | NA | **20.83  (4.04; 37.62)** | **Very Low^6^** | 33/12/ | **20.83  (4.04; 37.62)** | **Very Low^6^** |
| **130** | **DM vs ZN** | NA | NA | NA | NA | NA | **21.45  (9.15; 33.73)** | **Very Low^6^** | 37/12/ | **21.45  (9.15; 33.73)** | **Very Low^6^** |
| **131** | **DM vs VA** | NA | NA | NA | NA | NA | 9.01  (-9.89; 28.17) | Very Low^4^ | 46/12/ | 9.01  (-9.89; 28.17) | Very Low^4^ |
| **132** | **DM vs MN** | NA | NA | NA | NA | NA | 2.49  (-31.97; 37.42) | Very Low^4,6^ | 14/12/ | 2.49  (-31.97; 37.42) | Very Low^4,6^ |
| **133** | **DM vs LGG** | NA | NA | NA | NA | NA | **25.81  (13.2; 38.53)** | **Very Low^6^** | 50/12/ | **25.81  (13.2; 38.53)** | **Very Low^6^** |
| **134** | **DM vs SB** | NA | NA | NA | NA | NA | **19.57  (6.4; 32.77)** | **Very Low^6^** | 55/12/ | **19.57  (6.4; 32.77)** | **Very Low^6^** |
| **135** | **DM vs All-PRB** | NA | NA | NA | NA | NA | **22.42  (10.34; 34.58)** | **Very Low^6^** | 62/12/ | **22.42  (10.34; 34.58)** | **Very Low^6^** |
| **136** | **DM vs SYM** | NA | NA | NA | NA | NA | **29.31  (14.27; 44.51)** | **Very Low^6^** | 1/12/ | **29.31  (14.27; 44.51)** | **Very Low^6^** |
| **137** | **DM vs YOG** | NA | NA | NA | NA | NA | **19.56  (1.34; 37.45)** | **Low** | 10/12/ | **19.56  (1.34; 37.45)** | **Low** |
| **138** | **DM vs PRE** | NA | NA | NA | NA | NA | 18.29  (-11.08; 47.83) | Very Low^4^ | 11/12/ | 18.29  (-11.08; 47.83) | Very Low^4^ |
| **139** | **LGG+SM vs RC** | NA | NA | NA | NA | NA | **-33.85  (-49.22;-18.45)** | **Low** | 19/50/49 | **-33.85  (-49.22;-18.45)** | **Low** |
| **140** | **LGG+SM vs SM** | NA | NA | NA | NA | NA | **-27.14  (-42.19;-12.23)** | **Very Low** | 30/50/49 | **-27.14  (-42.19;-12.23)** | **Very Low** |
| **141** | **LGG+SM vs LOP** | NA | NA | NA | NA | NA | **-33.24  (-51.57;-14.87)** | **Low** | 33/50/49 | **-33.24  (-51.57;-14.87)** | **Low** |
| **142** | **LGG+SM vs ZN** | NA | NA | NA | NA | NA | **-32.67  (-46.5; -18.56)** | **Low** | 37/50/49 | **-32.67  (-46.5; -18.56)** | **Low** |
| **143** | **LGG+SM vs VA** | NA | NA | NA | NA | NA | **-45.08  (-65.26; -24.6)** | **Very Low^6^** | 46/50/49 | **-45.08  (-65.26; -24.6)** | **Very Low^6^** |
| **144** | **LGG+SM vs MN** | NA | NA | NA | NA | NA | **-51.7  (-87.11;-15.93)** | **Low** | 14/50/47 | **-51.7  (-87.11;-15.93)** | **Low** |
| **145** | **LGG+SM vs SB** | NA | NA | NA | NA | NA | **-34.52  (-49.27;-19.83)** | **Low** | 55/50/49 | **-34.52  (-49.27;-19.83)** | **Low** |
| **146** | **LGG+SM vs All-PRB** | NA | NA | NA | NA | NA | **-31.7  (-45.29;-17.81)** | **Low** | 62/50/49 | **-31.7  (-45.29;-17.81)** | **Low** |
| **147** | **LGG+SM vs SYM** | NA | NA | NA | NA | NA | **-24.83  (-41.29; -8.52)** | **Very Low^6^** | 1/50/49 | **-24.83  (-41.29; -8.52)** | **Very Low^6^** |
| **148** | **LGG+SM vs LCF** | NA | NA | NA | NA | NA | **-38.49  (-53.15;-23.78)** | **Very Low** | 3/50/49 | **-38.49  (-53.15;-23.78)** | **Very Low** |
| **149** | **LGG+SM vs YOG** | NA | NA | NA | NA | NA | **-34.54  (-53.23;-16.23)** | **Very Low^6^** | 10/50/49 | **-34.54  (-53.23;-16.23)** | **Very Low^6^** |
| **150** | **LGG+SM vs PRE** | NA | NA | NA | NA | NA | **-35.69  (-66.02; -5.5)** | **Low** | 11/50/49 | **-35.69  (-66.02; -5.5)** | **Low** |
| **151** | **LGG+SM vs DM** | NA | NA | NA | NA | NA | **-54.09  (-71.61;-36.97)** | Very Low^6^ | 12/50/49 | **-54.09  (-71.61;-36.97)** | Very Low^6^ |
| **152** | **ZN+PRB vs RC** | NA | NA | NA | NA | NA | -12.21  (-25.3; 0.7) | Very Low^4,6^ | 19/13/ | -12.21  (-25.3; 0.7) | Very Low^4,6^ |
| **153** | **ZN+PRB vs SM** | NA | NA | NA | NA | NA | -5.44  (-18.42; 7.47) | Very Low^4^ | 30/13/ | -5.44  (-18.42; 7.47) | Very Low^4^ |
| **154** | **ZN+PRB vs LOP** | NA | NA | NA | NA | NA | -11.53  (-28.14; 4.96) | Very Low^4,6^ | 33/13/ | -11.53  (-28.14; 4.96) | Very Low^4,6^ |
| **155** | **ZN+PRB vs VA** | NA | NA | NA | NA | NA | **-23.4  (-42.21; -4.55)** | **Low** | 46/13/ | **-23.4  (-42.21; -4.55)** | **Low** |
| **156** | **ZN+PRB vs MN** | NA | NA | NA | NA | NA | -30.13  (-64.38; 4.97) | Very Low^4^ | 14/13/47 | -30.13  (-64.38; 4.97) | Very Low^4^ |
| **157** | **ZN+PRB vs LGG** | NA | NA | NA | NA | NA | -6.68  (-18.91; 5.64) | Very Low^4^ | 50/13/ | -6.68  (-18.91; 5.64) | Very Low^4^ |
| **158** | **ZN+PRB vs SB** | NA | NA | NA | NA | NA | **-12.88  (-25.61; -0.23)** | **Low** | 55/13/ | **-12.88  (-25.61; -0.23)** | **Low** |
| **159** | **ZN+PRB vs SYM** | NA | NA | NA | NA | NA | -3.13  (-17.83; 11.52) | Very Low^4,6^ | 1/13/ | -3.13  (-17.83; 11.52) | Very Low^4,6^ |
| **160** | **ZN+PRB vs LCF** | NA | NA | NA | NA | NA | **-16.88  (-29.45; -4.37)** | **Very Low** | 3/13/ | **-16.88  (-29.45; -4.37)** | **Very Low** |
| **161** | **ZN+PRB vs YOG** | NA | NA | NA | NA | NA | -12.85  (-31.12; 4.46) | Very Low^4^ | 10/13/ | -12.85  (-31.12; 4.46) | Very Low^4^ |
| **162** | **ZN+PRB vs PRE** | NA | NA | NA | NA | NA | -14.1  (-43.72; 15.16) | Very Low^4^ | 11/13/ | -14.1  (-43.72; 15.16) | Very Low^4^ |
| **163** | **ZN+PRB vs DM** | NA | NA | NA | NA | NA | **-32.46  (-47.89; -16.8)** | **Low** | 12/13/ | **-32.46  (-47.89; -16.8)** | **Low** |
| **164** | **ZN+PRB vs LGG+SM** | NA | NA | NA | NA | NA | **21.72  (4.38; 38.62)** | **Low** | 50/13/49 | **21.72  (4.38; 38.62)** | **Low** |
| **165** | **STND vs MN** | NA | NA | NA | NA | NA | -0.68  (-33.29; 32.79) | Low4 | 38/14/ | -0.68  (-33.29; 32.79) | Low4 |
| **166** | **STND vs LGG+SM** | NA | NA | NA | NA | NA | **51.08  (37.85; 64.3)** | **Very Low^6^** | 49/50/49 | **51.08  (37.85; 64.3)** | **Very Low^6^** |
| **167** | **ZN+MN vs RC** | NA | NA | NA | NA | NA | -0.68  (-16.42; 14.91) | Very Low^4^ | 19/14/ | -0.68  (-16.42; 14.91) | Very Low^4^ |
| **168** | **ZN+MN vs SM** | NA | NA | NA | NA | NA | 6.08  (-9.41; 21.52) | Very Low^4^ | 30/14/ | 6.08  (-9.41; 21.52) | Very Low^4^ |
| **169** | **ZN+MN vs LOP** | NA | NA | NA | NA | NA | 0.01  (-18.67; 18.49) | Low4 | 33/14/ | 0.01  (-18.67; 18.49) | Low4 |
| **170** | **ZN+MN vs VA** | NA | NA | NA | NA | NA | -11.84  (-32.38; 8.93) | Low4 | 46/14/ | -11.84  (-32.38; 8.93) | Low4 |
| **171** | **ZN+MN vs LGG** | NA | NA | NA | NA | NA | 4.99  (-10.46; 19.93) | Very Low^4^ | 50/14/ | 4.99  (-10.46; 19.93) | Very Low^4^ |
| **172** | **ZN+MN vs SB** | NA | NA | NA | NA | NA | -1.28  (-16.86; 14.21) | Very Low^4^ | 55/14/ | -1.28  (-16.86; 14.21) | Very Low^4^ |
| **173** | **ZN+MN vs All-PRB** | NA | NA | NA | NA | NA | 1.61  (-13.08; 16.1) | Very Low^4^ | 62/14/ | 1.61  (-13.08; 16.1) | Very Low^4^ |
| **174** | **ZN+MN vs SYM** | NA | NA | NA | NA | NA | 8.56  (-8.65; 25.46) | Very Low^4,6^ | 1/14/ | 8.56  (-8.65; 25.46) | Very Low^4,6^ |
| **175** | **ZN+MN vs LCF** | NA | NA | NA | NA | NA | -5.33  (-20.48; 9.79) | Very Low^4^ | 3/14/ | -5.33  (-20.48; 9.79) | Very Low^4^ |
| **176** | **ZN+MN vs YOG** | NA | NA | NA | NA | NA | -1.3  (-21.24; 18.53) | Very Low^4^ | 10/14/ | -1.3  (-21.24; 18.53) | Very Low^4^ |
| **177** | **ZN+MN vs PRE** | NA | NA | NA | NA | NA | -2.42  (-33.37; 28.41) | Low4 | 11/14/ | -2.42  (-33.37; 28.41) | Low4 |
| **178** | **ZN+MN vs DM** | NA | NA | NA | NA | NA | **-20.8  (-38.57; -3.31)** | **Very Low^6^** | 12/14/ | **-20.8  (-38.57; -3.31)** | **Very Low^6^** |
| **179** | **ZN+MN vs LGG+SM** | NA | NA | NA | NA | NA | **33.21  (13.97; 52.49)** | **Low** | 50/14/49 | **33.21  (13.97; 52.49)** | **Low** |
| **180** | **ZN+MN vs ZN+PRB** | NA | NA | NA | NA | NA | 11.6  (-5.94; 28.93) | Very Low^4^ | 13/14/ | 11.6  (-5.94; 28.93) | Very Low^4^ |
| **181** | **CAO vs SM** | NA | NA | NA | NA | NA | 18.5  (-10.45; 47.63) | Very Low^4^ | 30/19/20 | 18.5  (-10.45; 47.63) | Very Low^4^ |
| **182** | **CAO vs LOP** | NA | NA | NA | NA | NA | 12.38  (-17.4; 42.75) | Very Low^4^ | 33/19/20 | 12.38  (-17.4; 42.75) | Very Low^4^ |
| **183** | **CAO vs ZN** | NA | NA | NA | NA | NA | 13.09  (-15.86; 41.52) | Very Low^4^ | 37/19/20 | 13.09  (-15.86; 41.52) | Very Low^4^ |
| **184** | **CAO vs VA** | NA | NA | NA | NA | NA | 0.69  (-31.6; 32.64) | Very Low^4,6^ | 47/19/20 | 0.69  (-31.6; 32.64) | Very Low^4,6^ |
| **185** | **CAO vs MN** | NA | NA | NA | NA | NA | -5.68  (-48.57; 37.92) | Very Low^4^ | 14/19/47 | -5.68  (-48.57; 37.92) | Very Low^4^ |
| **186** | **CAO vs LGG** | NA | NA | NA | NA | NA | 17.33  (-11.68; 46.1) | Very Low^4^ | 50/19/20 | 17.33  (-11.68; 46.1) | Very Low^4^ |
| **187** | **CAO vs SB** | NA | NA | NA | NA | NA | 11.05  (-17.96; 40.07) | Very Low^4^ | 55/19/20 | 11.05  (-17.96; 40.07) | Very Low^4^ |
| **188** | **CAO vs All-PRB** | NA | NA | NA | NA | NA | 14.05  (-14.63; 42.6) | Very Low^4,6^ | 62/19/20 | 14.05  (-14.63; 42.6) | Very Low^4,6^ |
| **189** | **CAO vs SYM** | NA | NA | NA | NA | NA | 20.84  (-8.93; 50.97) | Very Low^4^ | 1/19/20 | 20.84  (-8.93; 50.97) | Very Low^4^ |
| **190** | **CAO vs LCF** | NA | NA | NA | NA | NA | 7.24  (-21.81; 36.11) | Very Low^4^ | 3/19/20 | 7.24  (-21.81; 36.11) | Very Low^4^ |
| **191** | **CAO vs YOG** | NA | NA | NA | NA | NA | 11.06  (-20.52; 42.43) | Very Low^4^ | 10/19/20 | 11.06  (-20.52; 42.43) | Very Low^4^ |
| **192** | **CAO vs PRE** | NA | NA | NA | NA | NA | 9.88  (-29.19; 49.3) | Very Low^4^ | 11/19/20 | 9.88  (-29.19; 49.3) | Very Low^4^ |
| **193** | **CAO vs DM** | NA | NA | NA | NA | NA | -8.5  (-38.89; 21.8) | Very Low^4,6^ | 12/19/20 | -8.5  (-38.89; 21.8) | Very Low^4,6^ |
| **194** | **CAO vs LGG+SM** | NA | NA | NA | NA | NA | **45.56  (14.56; 76.74)** | **Low** | 50/19/49 | **45.56  (14.56; 76.74)** | **Low** |
| **195** | **CAO vs ZN+PRB** | NA | NA | NA | NA | NA | 24.14  (-6.59; 53.79) | Very Low^4,6^ | 13/19/20 | 24.14  (-6.59; 53.79) | Very Low^4,6^ |
| **196** | **CAO vs STND** | NA | NA | NA | NA | NA | -5.32  (-33.76; 22.83) | Very Low^4^ | 20/19/20 | -5.32  (-33.76; 22.83) | Very Low^4^ |
| **197** | **CAO vs ZN+MN** | NA | NA | NA | NA | NA | 12.43  (-19.22; 43.55) | Very Low^4^ | 14/19/20 | 12.43  (-19.22; 43.55) | Very Low^4^ |
| **198** | **LCF+PRB vs RC** | NA | NA | NA | NA | NA | 3.97  (-19.77; 27.85) | Very Low^4^ | 19/3/4 | 3.97  (-19.77; 27.85) | Very Low^4^ |
| **199** | **LCF+PRB vs SM** | NA | NA | NA | NA | NA | 10.68  (-13.22; 34.45) | Very Low^4^ | 30/3/4 | 10.68  (-13.22; 34.45) | Very Low^4^ |
| **200** | **LCF+PRB vs LOP** | NA | NA | NA | NA | NA | 4.57  (-21.49; 30.33) | Very Low^4^ | 33/3/4 | 4.57  (-21.49; 30.33) | Very Low^4^ |
| **201** | **LCF+PRB vs ZN** | NA | NA | NA | NA | NA | 5.12  (-17.97; 28.06) | Very Low^4^ | 37/3/4 | 5.12  (-17.97; 28.06) | Very Low^4^ |
| **202** | **LCF+PRB vs VA** | NA | NA | NA | NA | NA | -7.25  (-34.83; 19.82) | Very Low^4^ | 47/3/4 | -7.25  (-34.83; 19.82) | Very Low^4^ |
| **203** | **LCF+PRB vs MN** | NA | NA | NA | NA | NA | -13.88  (-53.21; 25.84) | Very Low^4^ | 14/3/47 | -13.88  (-53.21; 25.84) | Very Low^4^ |
| **204** | **LCF+PRB vs LGG** | NA | NA | NA | NA | NA | 9.54  (-14.21; 32.6) | Very Low^4^ | 50/3/4 | 9.54  (-14.21; 32.6) | Very Low^4^ |
| **205** | **LCF+PRB vs SB** | NA | NA | NA | NA | NA | 3.23  (-20.4; 26.73) | Very Low^4^ | 55/3/4 | 3.23  (-20.4; 26.73) | Very Low^4^ |
| **206** | **LCF+PRB vs All-PRB** | NA | NA | NA | NA | NA | 6.12  (-17.02; 28.87) | Very Low^4^ | 62/3/4 | 6.12  (-17.02; 28.87) | Very Low^4^ |
| **207** | **LCF+PRB vs SYM** | NA | NA | NA | NA | NA | 13.06  (-11.73; 37.39) | Very Low^4^ | 1/3/4 | 13.06  (-11.73; 37.39) | Very Low^4^ |
| **208** | **LCF+PRB vs YOG** | NA | NA | NA | NA | NA | 3.29  (-23.68; 29.7) | Very Low^4^ | 10/3/4 | 3.29  (-23.68; 29.7) | Very Low^4^ |
| **209** | **LCF+PRB vs PRE** | NA | NA | NA | NA | NA | 2.08  (-33.4; 37.6) | Very Low^4^ | 11/3/4 | 2.08  (-33.4; 37.6) | Very Low^4^ |
| **210** | **LCF+PRB vs DM** | NA | NA | NA | NA | NA | -16.24  (-41.26; 8.75) | Very Low^4^ | 12/3/4 | -16.24  (-41.26; 8.75) | Very Low^4^ |
| **211** | **LCF+PRB vs LGG+SM** | NA | NA | NA | NA | NA | **37.84  (11.48; 63.84)** | **Very Low** | 50/3/49 | **37.84  (11.48; 63.84)** | **Very Low** |
| **212** | **LCF+PRB vs ZN+PRB** | NA | NA | NA | NA | NA | 16.23  (-9.14; 40.95) | Very Low^4^ | 13/3/4 | 16.23  (-9.14; 40.95) | Very Low^4^ |
| **213** | **LCF+PRB vs STND** | NA | NA | NA | NA | NA | -13.27  (-35.96; 9.19) | Very Low^4^ | 4/3/4 | -13.27  (-35.96; 9.19) | Very Low^4^ |
| **214** | **LCF+PRB vs ZN+MN** | NA | NA | NA | NA | NA | 4.46  (-21.63; 31.13) | Very Low^4^ | 14/3/47 | 4.46  (-21.63; 31.13) | Very Low^4^ |
| **215** | **LCF+PRB vs CAO** | NA | NA | NA | NA | NA | -8.03  (-43.76; 28.57) | Very Low^4^ | 19/3/20 | -8.03  (-43.76; 28.57) | Very Low^4^ |
| **216** | **SB+ZN vs RC** | NA | NA | NA | NA | NA | **-22.24  (-36.99; -7.41)** | **Very Low^6^** | 19/15/ | **-22.24  (-36.99; -7.41)** | **Very Low^6^** |
| **217** | **SB+ZN vs SM** | NA | NA | NA | NA | NA | **-15.51  (-30.32; -0.94)** | **Very Low** | 30/15/ | **-15.51  (-30.32; -0.94)** | **Very Low** |
| **218** | **SB+ZN vs LOP** | NA | NA | NA | NA | NA | **-21.73  (-39.22; -3.8)** | **Very Low^6^** | 33/15/ | **-21.73  (-39.22; -3.8)** | **Very Low^6^** |
| **219** | **SB+ZN vs VA** | NA | NA | NA | NA | NA | **-33.4  (-53.52;-13.33)** | **Low** | 47/15/ | **-33.4  (-53.52; -13.33)** | **Low** |
| **220** | **SB+ZN vs MN** | NA | NA | NA | NA | NA | **-39.98  (-74.93; -4.85)** | **Low** | 14/15/47 | **-39.98  (-74.93; -4.85)** | **Low** |
| **221** | **SB+ZN vs LGG** | NA | NA | NA | NA | NA | **-16.72  (-30.78; -2.79)** | **Very Low^6^** | 50/15/ | **-16.72  (-30.78; -2.79)** | **Very Low^6^** |
| **222** | **SB+ZN vs All-PRB** | NA | NA | NA | NA | NA | **-20.1  (-33.73; -6.7)** | **Low** | 62/15/ | **-20.1  (-33.73; -6.7)** | **Low** |
| **223** | **SB+ZN vs SYM** | NA | NA | NA | NA | NA | -13.19  (-29.67; 2.91) | Very Low^4,6^ | 1/15/ | -13.19  (-29.67; 2.91) | Very Low^4,6^ |
| **224** | **SB+ZN vs YOG** | NA | NA | NA | NA | NA | **-22.96  (-42.15; -4.44)** | **Very Low^6^** | 10/15/ | **-22.96  (-42.15; -4.44)** | **Very Low^6^** |
| **225** | **SB+ZN vs PRE** | NA | NA | NA | NA | NA | -24.13  (-54.58; 6.02) | Very Low^4,6^ | 11/15/ | -24.13  (-54.58; 6.02) | Very Low^4,6^ |
| **226** | **SB+ZN vs DM** | NA | NA | NA | NA | NA | **-42.49  (-59.53;-25.37)** | **Very Low^6^** | 12/15/ | **-42.49  (-59.53;-25.37)** | **Very Low^6^** |
| **227** | **SB+ZN vs LGG+SM** | NA | NA | NA | NA | NA | 11.62  (-6.91; 29.65) | Very Low^4,6^ | 50/15/49 | 11.62  (-6.91; 29.65) | Very Low^4,6^ |
| **228** | **SB+ZN vs ZN+PRB** | NA | NA | NA | NA | NA | -10.06  (-26.12; 6.1) | Very Low^4,6^ | 13/15/ | -10.06  (-26.12; 6.1) | Very Low^4,6^ |
| **229** | **SB+ZN vs ZN+MN** | NA | NA | NA | NA | NA | **-21.63  (-40.4; -3.14)** | **Very Low^6^** | 14/15/47 | **-21.63  (-40.4; -3.14)** | **Very Low^6^** |
| **230** | **SB+ZN vs CAO** | NA | NA | NA | NA | NA | **-34  (-65.23; -3.13)** | **Very Low^6^** | 19/15/20 | **-34  (-65.23; -3.13)** | **Very Low^6^** |
| **231** | **SB+ZN vs LCF+PRB** | NA | NA | NA | NA | NA | **-26.16  (-51.92; -0.3)** | **Very Low** | 3/15/ | **-26.16  (-51.92; -0.3)** | **Very Low** |
| **232** | **SB+LCF vs RC** | NA | NA | NA | NA | NA | 4.79  (-14.37; 24.25) | Very Low^4,6^ | 19/16/ | 4.79  (-14.37; 24.25) | Very Low^4,6^ |
| **233** | **SB+LCF vs SM** | NA | NA | NA | NA | NA | 11.64  (-7.47; 30.8) | Very Low^4^ | 30/16/ | 11.64  (-7.47; 30.8) | Very Low^4^ |
| **234** | **SB+LCF vs LOP** | NA | NA | NA | NA | NA | 5.55  (-16.26; 27.64) | Very Low^4,6^ | 33/16/ | 5.55  (-16.26; 27.64) | Very Low^4,6^ |
| **235** | **SB+LCF vs VA** | NA | NA | NA | NA | NA | -6.34  (-29.64; 17.69) | Very Low^4,6^ | 47/16/ | -6.34  (-29.64; 17.69) | Very Low^4,6^ |
| **236** | **SB+LCF vs MN** | NA | NA | NA | NA | NA | -12.89  (-49.21; 25.12) | Very Low^4^ | 14/16/47 | -12.89  (-49.21; 25.12) | Very Low^4^ |
| **237** | **SB+LCF vs LGG** | NA | NA | NA | NA | NA | 10.36  (-8.66; 29.65) | Very Low^4,6^ | 50/16/ | 10.36  (-8.66; 29.65) | Very Low^4,6^ |
| **238** | **SB+LCF vs All-PRB** | NA | NA | NA | NA | NA | 7.04  (-11.22; 25.66) | Very Low^4^ | 62/16/ | 7.04  (-11.22; 25.66) | Very Low^4^ |
| **239** | **SB+LCF vs SYM** | NA | NA | NA | NA | NA | 13.96  (-6.39; 34.43) | Very Low^4,6^ | 1/16/ | 13.96  (-6.39; 34.43) | Very Low^4,6^ |
| **240** | **SB+LCF vs YOG** | NA | NA | NA | NA | NA | 4.04  (-18.21; 26.97) | Very Low^4,6^ | 10/16/ | 4.04  (-18.21; 26.97) | Very Low^4,6^ |
| **241** | **SB+LCF vs PRE** | NA | NA | NA | NA | NA | 2.89  (-29.55; 35.77) | Very Low^4,6^ | 11/16/ | 2.89  (-29.55; 35.77) | Very Low^4,6^ |
| **242** | **SB+LCF vs DM** | NA | NA | NA | NA | NA | -15.45  (-36.3; 5.94) | Very Low^4,6^ | 12/16/ | -15.45  (-36.3; 5.94) | Very Low^4,6^ |
| **243** | **SB+LCF vs LGG+SM** | NA | NA | NA | NA | NA | **38.73  (16.82; 61.14)** | **Low** | 50/16/49 | **38.73  (16.82; 61.14)** | **Low** |
| **244** | **SB+LCF vs ZN+PRB** | NA | NA | NA | NA | NA | 16.97  (-3.34; 38.17) | Very Low^4^ | 13/16/ | 16.97  (-3.34; 38.17) | Very Low^4^ |
| **245** | **SB+LCF vs ZN+MN** | NA | NA | NA | NA | NA | 5.43  (-16.59; 28.39) | Very Low^4^ | 14/16/ | 5.43  (-16.59; 28.39) | Very Low^4^ |
| **246** | **SB+LCF vs CAO** | NA | NA | NA | NA | NA | -6.91  (-40.72; 25.89) | Very Low^4,6^ | 19/16/20 | -6.91  (-40.72; 25.89) | Very Low^4,6^ |
| **247** | **SB+LCF vs LCF+PRB** | NA | NA | NA | NA | NA | 0.92  (-27.12; 29.71) | Very Low^4^ | 3/16/ | 0.92  (-27.12; 29.71) | Very Low^4^ |
| **248** | **ZN+LCF vs RC** | NA | NA | NA | NA | NA | -4.28  (-21.16; 12.44) | Very Low^4,6^ | 19/17/ | -4.28  (-21.16; 12.44) | Very Low^4,6^ |
| **249** | **ZN+LCF vs SM** | NA | NA | NA | NA | NA | 2.52  (-14.28; 19.2) | Very Low^4^ | 30/17/ | 2.52  (-14.28; 19.2) | Very Low^4^ |
| **250** | **ZN+LCF vs LOP** | NA | NA | NA | NA | NA | -3.57  (-23.37; 15.96) | Very Low^4,6^ | 33/17/ | -3.57  (-23.37; 15.96) | Very Low^4,6^ |
| **251** | **ZN+LCF vs VA** | NA | NA | NA | NA | NA | -15.45  (-36.8; 6.2) | Very Low^4^ | 47/17/ | -15.45  (-36.8; 6.2) | Very Low^4^ |
| **252** | **ZN+LCF vs MN** | NA | NA | NA | NA | NA | -21.92  (-57.71; 14.41) | Very Low^4^ | 14/17/47 | -21.92  (-57.71; 14.41) | Very Low^4^ |
| **253** | **ZN+LCF vs LGG** | NA | NA | NA | NA | NA | 1.35  (-14.72; 17.66) | Very Low^4,6^ | 50/17/ | 1.35  (-14.72; 17.66) | Very Low^4,6^ |
| **254** | **ZN+LCF vs All-PRB** | NA | NA | NA | NA | NA | -2.03  (-17.73; 13.74) | Very Low^4^ | 62/17/ | -2.03  (-17.73; 13.74) | Very Low^4^ |
| **255** | **ZN+LCF vs SYM** | NA | NA | NA | NA | NA | 4.8  (-13.46; 23.33) | Very Low^4,6^ | 1/17/ | 4.8  (-13.46; 23.33) | Very Low^4,6^ |
| **256** | **ZN+LCF vs YOG** | NA | NA | NA | NA | NA | -4.97  (-25.72; 15.63) | Very Low^4^ | 10/17/ | -4.97  (-25.72; 15.63) | Very Low^4^ |
| **257** | **ZN+LCF vs PRE** | NA | NA | NA | NA | NA | -6.23  (-37.18; 25.2) | Very Low^4,6^ | 11/17/ | -6.23  (-37.18; 25.2) | Very Low^4,6^ |
| **258** | **ZN+LCF vs DM** | NA | NA | NA | NA | NA | **-24.52  (-43.17; -5.47)** | **Very Low^6^** | 12/17/ | **-24.52  (-43.17; -5.47)** | **Very Low^6^** |
| **259** | **ZN+LCF vs LGG+SM** | NA | NA | NA | NA | NA | **29.74  (9.19; 49.6)** | **Low** | 50/17/49 | **29.74  (9.19; 49.6)** | **Low** |
| **260** | **ZN+LCF vs ZN+PRB** | NA | NA | NA | NA | NA | 7.89  (-10.1; 26.47) | Very Low^4^ | 13/17/ | 7.89  (-10.1; 26.47) | Very Low^4^ |
| **261** | **ZN+LCF vs ZN+MN** | NA | NA | NA | NA | NA | -3.64  (-23.98; 16.65) | Very Low^4,6^ | 14/17/ | -3.64  (-23.98; 16.65) | Very Low^4,6^ |
| **262** | **ZN+LCF vs CAO** | NA | NA | NA | NA | NA | -15.99  (-48.33; 15.88) | Very Low^4,6^ | 19/17/20 | -15.99  (-48.33; 15.88) | Very Low^4,6^ |
| **263** | **ZN+LCF vs LCF+PRB** | NA | NA | NA | NA | NA | -8.32  (-34.68; 19.09) | Very Low^4^ | 3/17/ | -8.32  (-34.68; 19.09) | Very Low^4^ |
| **264** | **SB+ZN+LCF vs RC** | NA | NA | NA | NA | NA | 0.59  (-20.31; 21.23) | Very Low^4,6^ | 19/18/ | 0.59  (-20.31; 21.23) | Very Low^4,6^ |
| **265** | **SB+ZN+LCF vs SM** | NA | NA | NA | NA | NA | 7.27  (-13.29; 27.88) | Very Low^4^ | 30/18/ | 7.27  (-13.29; 27.88) | Very Low^4^ |
| **266** | **SB+ZN+LCF vs LOP** | NA | NA | NA | NA | NA | 1.04  (-21.66; 23.88) | Very Low^4,6^ | 33/18/ | 1.04  (-21.66; 23.88) | Very Low^4,6^ |
| **267** | **SB+ZN+LCF vs VA** | NA | NA | NA | NA | NA | -10.72  (-35.73; 13.82) | Very Low^4^ | 47/18/ | -10.72  (-35.73; 13.82) | Very Low^4^ |
| **268** | **SB+ZN+LCF vs MN** | NA | NA | NA | NA | NA | -17.3  (-55.28; 21.02) | Very Low^4^ | 14/18/47 | -17.3  (-55.28; 21.02) | Very Low^4^ |
| **269** | **SB+ZN+LCF vs LGG** | NA | NA | NA | NA | NA | 6.06  (-14.14; 26.47) | Very Low^4,6^ | 50/18/ | 6.06  (-14.14; 26.47) | Very Low^4,6^ |
| **270** | **SB+ZN+LCF vs All-PRB** | NA | NA | NA | NA | NA | 2.63  (-17.22; 22.65) | Very Low^4^ | 62/18/ | 2.63  (-17.22; 22.65) | Very Low^4^ |
| **271** | **SB+ZN+LCF vs SYM** | NA | NA | NA | NA | NA | 9.39  (-12.3; 31.3) | Very Low^4,6^ | 1/18/ | 9.39  (-12.3; 31.3) | Very Low^4,6^ |
| **272** | **SB+ZN+LCF vs YOG** | NA | NA | NA | NA | NA | -0.33  (-23.96; 23.58) | Very Low^4^ | 10/18/ | -0.33  (-23.96; 23.58) | Very Low^4^ |
| **273** | **SB+ZN+LCF vs PRE** | NA | NA | NA | NA | NA | -1.42  (-34.46; 32.09) | Very Low^4,6^ | 11/18/ | -1.42  (-34.46; 32.09) | Very Low^4,6^ |
| **274** | **SB+ZN+LCF vs DM** | NA | NA | NA | NA | NA | -19.95  (-42.09; 2.9) | Very Low^4,6^ | 12/18/ | -19.95  (-42.09; 2.9) | Very Low^4,6^ |
| **275** | **SB+ZN+LCF vs LGG+SM** | NA | NA | NA | NA | NA | **34.28  (10.99; 57.65)** | **Low** | 50/18/49 | **34.28  (10.99; 57.65)** | **Low** |
| **276** | **SB+ZN+LCF vs ZN+PRB** | NA | NA | NA | NA | NA | 12.6  (-9.43; 34.53) | Very Low^4^ | 13/18/ | 12.6  (-9.43; 34.53) | Very Low^4^ |
| **277** | **SB+ZN+LCF vs ZN+MN** | NA | NA | NA | NA | NA | 1.03  (-22.64; 24.83) | Very Low^4^ | 14/18/ | 1.03  (-22.64; 24.83) | Very Low^4^ |
| **278** | **SB+ZN+LCF vs CAO** | NA | NA | NA | NA | NA | -11.2  (-45.97; 22.33) | Very Low^4^ | 19/18/20 | -11.2  (-45.97; 22.33) | Very Low^4^ |
| **279** | **SB+ZN+LCF vs LCF+PRB** | NA | NA | NA | NA | NA | -3.63  (-32.93; 26.23) | Very Low^4^ | 3/18/ | -3.63  (-32.93; 26.23) | Very Low^4^ |
| **280** | **SYM+LCF vs RC** | NA | NA | NA | NA | NA | -15.02  (-36.67; 6.87) | Very Low^4^ | 19/3/4 | -15.02  (-36.67; 6.87) | Very Low^4^ |
| **281** | **SYM+LCF vs SM** | NA | NA | NA | NA | NA | -8.27  (-30.36; 13.83) | Very Low^4^ | 30/3/4 | -8.27  (-30.36; 13.83) | Very Low^4^ |
| **282** | **SYM+LCF vs LOP** | NA | NA | NA | NA | NA | -14.29  (-38.36; 9.78) | Very Low^4^ | 33/3/4 | -14.29  (-38.36; 9.78) | Very Low^4^ |
| **283** | **SYM+LCF vs ZN** | NA | NA | NA | NA | NA | -13.8  (-34.81; 7.34) | Very Low^4^ | 37/3/4 | -13.8  (-34.81; 7.34) | Very Low^4^ |
| **284** | **SYM+LCF vs VA** | NA | NA | NA | NA | NA | **-26.13  (-52.02; -0.07)** | Very Low | 47/3/4 | **-26.13  (-52.02; -0.07)** | Very Low |
| **285** | **SYM+LCF vs MN** | NA | NA | NA | NA | NA | -32.83  (-70.77; 6.91) | Very Low^4^ | 14/3/47 | -32.83  (-70.77; 6.91) | Very Low^4^ |
| **286** | **SYM+LCF vs LGG** | NA | NA | NA | NA | NA | -9.38  (-31.11; 12.31) | Very Low^4^ | 50/3/4 | -9.38  (-31.11; 12.31) | Very Low^4^ |
| **287** | **SYM+LCF vs SB** | NA | NA | NA | NA | NA | -15.67  (-37.36; 6.34) | Very Low^4^ | 55/3/4 | -15.67  (-37.36; 6.34) | Very Low^4^ |
| **288** | **SYM+LCF vs All-PRB** | NA | NA | NA | NA | NA | -12.8  (-34.12; 8.51) | Very Low^4^ | 62/3/4 | -12.8  (-34.12; 8.51) | Very Low^4^ |
| **289** | **SYM+LCF vs SYM** | NA | NA | NA | NA | NA | -5.89  (-29.03; 17.55) | Very Low^4^ | 1/3/4 | -5.89  (-29.03; 17.55) | Very Low^4^ |
| **290** | **SYM+LCF vs YOG** | NA | NA | NA | NA | NA | -15.7  (-40.85; 9.58) | Very Low^4^ | 10/3/4 | -15.7  (-40.85; 9.58) | Very Low^4^ |
| **291** | **SYM+LCF vs PRE** | NA | NA | NA | NA | NA | -16.93  (-51.28; 17.24) | Very Low^4^ | 11/3/4 | -16.93  (-51.28; 17.24) | Very Low^4^ |
| **292** | **SYM+LCF vs DM** | NA | NA | NA | NA | NA | **-35.21  (-58.7; -11.68)** | **Very Low** | 12/3/4 | **-35.21  (-58.7; -11.68)** | **Very Low** |
| **293** | **SYM+LCF vs LGG+SM** | NA | NA | NA | NA | NA | 18.89  (-5.45; 43.76) | Very Low^4^ | 50/3/49 | 18.89  (-5.45; 43.76) | Very Low^4^ |
| **294** | **SYM+LCF vs ZN+PRB** | NA | NA | NA | NA | NA | -2.72  (-26.09; 20.68) | Very Low^4^ | 13/3/ | -2.72  (-26.09; 20.68) | Very Low^4^ |
| **295** | **SYM+LCF vs STND** | NA | NA | NA | NA | NA | **-32.11  (-53.01;-11.33)** | **Very Low^4^** | 9/3/ | **-32.11  (-53.01;-11.33)** | **Very Low^4^** |
| **296** | **SYM+LCF vs ZN+MN** | NA | NA | NA | NA | NA | -14.33  (-38.98; 10.52) | Very Low^4^ | 14/3/ | -14.33  (-38.98; 10.52) | Very Low^4^ |
| **297** | **SYM+LCF vs CAO** | NA | NA | NA | NA | NA | -26.88  (-61.54; 8.17) | Very Low^4^ | 19/3/20 | -26.88  (-61.54; 8.17) | Very Low^4^ |
| **298** | **SYM+LCF vs LCF+PRB** | NA | NA | NA | NA | NA | -18.83  (-48.18; 10.57) | Very Low^4^ | 3/3/ | -18.83  (-48.18; 10.57) | Very Low^4^ |
| **299** | **SYM+LCF vs SB+ZN** | NA | NA | NA | NA | NA | 7.26  (-16.85; 31.59) | Very Low^4^ | 15/3/ | 7.26  (-16.85; 31.59) | Very Low^4^ |
| **300** | **SYM+LCF vs SB+LCF** | NA | NA | NA | NA | NA | -19.75  (-46.46; 7.21) | Very Low^4^ | 16/3/ | -19.75  (-46.46; 7.21) | Very Low^4^ |
| **301** | **SYM+LCF vs ZN+LCF** | NA | NA | NA | NA | NA | -10.6  (-35.96; 14.42) | Very Low^4^ | 17/3/ | -10.6  (-35.96; 14.42) | Very Low^4^ |
| **302** | **SYM+LCF vs SB+ZN+LCF** | NA | NA | NA | NA | NA | -15.3  (-43.59; 13.07) | Very Low^4^ | 18/3/ | -15.3  (-43.59; 13.07) | Very Low^4^ |
| **303** | **SM+ZN vs RC** | NA | NA | NA | NA | NA | -18.45  (-41.52; 4.84) | Very Low^4^ | 19/37/ | -18.45  (-41.52; 4.84) | Very Low^4^ |
| **304** | **SM+ZN vs SM** | NA | NA | NA | NA | NA | -11.69  (-34.57; 11.56) | Very Low^4^ | 30/37/ | -11.69  (-34.57; 11.56) | Very Low^4^ |
| **305** | **SM+ZN vs LOP** | NA | NA | NA | NA | NA | -17.84  (-42.78; 7.17) | Low4 | 33/37/ | -17.84  (-42.78; 7.17) | Low4 |
| **306** | **SM+ZN vs VA** | NA | NA | NA | NA | NA | **-29.54  (-56.09; -2.84)** | **Moderate** | 47/37/ | **-29.54  (-56.09; -2.84)** | **Moderate** |
| **307** | **SM+ZN vs MN** | NA | NA | NA | NA | NA | -36.12  (-75.33; 3.67) | Low4 | 14/37/47 | -36.12  (-75.33; 3.67) | Low4 |
| **308** | **SM+ZN vs LGG** | NA | NA | NA | NA | NA | -12.79  (-35.54; 10) | Very Low^4^ | 50/37/ | -12.79  (-35.54; 10) | Very Low^4^ |
| **309** | **SM+ZN vs SB** | NA | NA | NA | NA | NA | -19.1  (-41.83; 4.08) | Very Low^4^ | 55/37/ | -19.1  (-41.83; 4.08) | Very Low^4^ |
| **310** | **SM+ZN vs All-PRB** | NA | NA | NA | NA | NA | -16.25  (-38.49; 6.33) | Very Low^4^ | 62/37/ | -16.25  (-38.49; 6.33) | Very Low^4^ |
| **311** | **SM+ZN vs SYM** | NA | NA | NA | NA | NA | -9.4  (-33.45; 14.77) | Very Low^4,6^ | 1/37/ | -9.4  (-33.45; 14.77) | Very Low^4,6^ |
| **312** | **SM+ZN vs LCF** | NA | NA | NA | NA | NA | **-23.19  (-45.52; -0.03)** | **Very Low** | 3/37/ | **-23.19  (-45.52; -0.03)** | **Very Low** |
| **313** | **SM+ZN vs YOG** | NA | NA | NA | NA | NA | -19.14  (-45.46; 7.26) | Very Low^4^ | 10/37/ | -19.14  (-45.46; 7.26) | Very Low^4^ |
| **314** | **SM+ZN vs PRE** | NA | NA | NA | NA | NA | -20.23  (-55.61; 14.91) | Very Low^4,6^ | 11/37/ | -20.23  (-55.61; 14.91) | Very Low^4,6^ |
| **315** | **SM+ZN vs DM** | NA | NA | NA | NA | NA | **-38.72  (-63.4; -13.6)** | **Very Low^6^** | 12/37/ | **-38.72  (-63.4; -13.6)** | **Very Low^6^** |
| **316** | **SM+ZN vs LGG+SM** | NA | NA | NA | NA | NA | 15.51  (-10.34; 41.31) | Very Low^4^ | 50/37/49 | 15.51  (-10.34; 41.31) | Very Low^4^ |
| **317** | **SM+ZN vs ZN+PRB** | NA | NA | NA | NA | NA | -6.16  (-30.18; 18.25) | Very Low^4^ | 13/37/ | -6.16  (-30.18; 18.25) | Very Low^4^ |
| **318** | **SM+ZN vs STND** | NA | NA | NA | NA | NA | **-35.63  (-57.57;-13.16)** | **Moderate** | 9/37/ | **-35.63  (-57.57;-13.16)** | **Moderate** |
| **319** | **SM+ZN vs ZN+MN** | NA | NA | NA | NA | NA | -17.78  (-43.29; 8.07) | Low4 | 14/37/ | -17.78  (-43.29; 8.07) | Low4 |
| **320** | **SM+ZN vs CAO** | NA | NA | NA | NA | NA | -30.25  (-65.69; 5.7) | Very Low^4^ | 19/37/20 | -30.25  (-65.69; 5.7) | Very Low^4^ |
| **321** | **SM+ZN vs LCF+PRB** | NA | NA | NA | NA | NA | -22.37  (-53.7; 8.75) | Very Low^4^ | 3/37/ | -22.37  (-53.7; 8.75) | Very Low^4^ |
| **322** | **SM+ZN vs SB+ZN** | NA | NA | NA | NA | NA | 3.9  (-21.15; 28.52) | Very Low^4^ | 15/37/ | 3.9  (-21.15; 28.52) | Very Low^4^ |
| **323** | **SM+ZN vs SB+LCF** | NA | NA | NA | NA | NA | -23.19  (-51.63; 4.67) | Very Low^4^ | 16/37/ | -23.19  (-51.63; 4.67) | Very Low^4^ |
| **324** | **SM+ZN vs ZN+LCF** | NA | NA | NA | NA | NA | -14.05  (-40.33; 12.2) | Very Low^4^ | 17/37/ | -14.05  (-40.33; 12.2) | Very Low^4^ |
| **325** | **SM+ZN vs SB+ZN+LCF** | NA | NA | NA | NA | NA | -18.77  (-48.17; 10.03) | Very Low^4^ | 18/37/ | -18.77  (-48.17; 10.03) | Very Low^4^ |
| **326** | **SM+ZN vs SYM+LCF** | NA | NA | NA | NA | NA | -3.53  (-33.17; 26.62) | Very Low^4^ | 3/38/ | -3.53  (-33.17; 26.62) | Very Low^4^ |
| **327** | **YOG+PRB+ZN vs RC** | NA | NA | NA | NA | NA | 1.62  (-40.28; 44.18) | Very Low^4^ | 19/15/ | 1.62  (-40.28; 44.18) | Very Low^4^ |
| **328** | **YOG+PRB+ZN vs SM** | NA | NA | NA | NA | NA | 8.28  (-33.3; 50.62) | Very Low^4^ | 30/15/ | 8.28  (-33.3; 50.62) | Very Low^4^ |
| **329** | **YOG+PRB+ZN vs LOP** | NA | NA | NA | NA | NA | 2.07  (-40.95; 45.94) | Very Low^4,6^ | 33/15/ | 2.07  (-40.95; 45.94) | Very Low^4,6^ |
| **330** | **YOG+PRB+ZN vs ZN** | NA | NA | NA | NA | NA | 2.77  (-38.42; 44.73) | Very Low^4^ | 26/39/ | 2.77  (-38.42; 44.73) | Very Low^4^ |
| **331** | **YOG+PRB+ZN vs VA** | NA | NA | NA | NA | NA | -9.62  (-53.8; 35.15) | Very Low^4^ | 47/15/ | -9.62  (-53.8; 35.15) | Very Low^4^ |
| **332** | **YOG+PRB+ZN vs MN** | NA | NA | NA | NA | NA | -16.38  (-68.81; 36.12) | Very Low^4^ | 14/15/ | -16.38  (-68.81; 36.12) | Very Low^4^ |
| **333** | **YOG+PRB+ZN vs LGG** | NA | NA | NA | NA | NA | 7.2  (-34.45; 49.53) | Very Low^4^ | 50/15/ | 7.2  (-34.45; 49.53) | Very Low^4^ |
| **334** | **YOG+PRB+ZN vs SB** | NA | NA | NA | NA | NA | 0.74  (-40.46; 43.77) | Very Low^4^ | 55/15/ | 0.74  (-40.46; 43.77) | Very Low^4^ |
| **335** | **YOG+PRB+ZN vs All-PRB** | NA | NA | NA | NA | NA | 3.52  (-37.69; 46.16) | Very Low^4^ | 62/15/ | 3.52  (-37.69; 46.16) | Very Low^4^ |
| **336** | **YOG+PRB+ZN vs SYM** | NA | NA | NA | NA | NA | 10.69  (-31.96; 53.96) | Very Low^4,6^ | 1/15/ | 10.69  (-31.96; 53.96) | Very Low^4,6^ |
| **337** | **YOG+PRB+ZN vs LCF** | NA | NA | NA | NA | NA | -3.06  (-44.55; 39.41) | Very Low^4^ | 3/15/ | -3.06  (-44.55; 39.41) | Very Low^4^ |
| **338** | **YOG+PRB+ZN vs YOG** | NA | NA | NA | NA | NA | 0.88  (-42.66; 45.08) | Very Low^4^ | 10/15/ | 0.88  (-42.66; 45.08) | Very Low^4^ |
| **339** | **YOG+PRB+ZN vs PRE** | NA | NA | NA | NA | NA | -0.3  (-50.37; 49.22) | Very Low^4^ | 11/15/ | -0.3  (-50.37; 49.22) | Very Low^4^ |
| **340** | **YOG+PRB+ZN vs DM** | NA | NA | NA | NA | NA | -18.65  (-61.56; 25.1) | Very Low^4,6^ | 12/15/ | -18.65  (-61.56; 25.1) | Very Low^4,6^ |
| **341** | **YOG+PRB+ZN vs LGG+SM** | NA | NA | NA | NA | NA | 35.27  (-8.01; 79.57) | Very Low^4^ | 50/15/49 | 35.27  (-8.01; 79.57) | Very Low^4^ |
| **342** | **YOG+PRB+ZN vs ZN+PRB** | NA | NA | NA | NA | NA | 13.73  (-28.85; 56.8) | Very Low^4^ | 13/15/ | 13.73  (-28.85; 56.8) | Very Low^4^ |
| **343** | **YOG+PRB+ZN vs STND** | NA | NA | NA | NA | NA | -15.63  (-56.82; 26.63) | Very Low^4^ | 26/15/ | -15.63  (-56.82; 26.63) | Very Low^4^ |
| **344** | **YOG+PRB+ZN vs ZN+MN** | NA | NA | NA | NA | NA | 2.17  (-40.9; 46.42) | Very Low^4^ | 14/15/ | 2.17  (-40.9; 46.42) | Very Low^4^ |
| **345** | **YOG+PRB+ZN vs CAO** | NA | NA | NA | NA | NA | -10.3  (-59.79; 40.02) | Very Low^4^ | 19/15/20 | -10.3  (-59.79; 40.02) | Very Low^4^ |
| **346** | **YOG+PRB+ZN vs LCF+PRB** | NA | NA | NA | NA | NA | -2.59  (-48.79; 44.19) | Very Low^4^ | 3/15/ | -2.59  (-48.79; 44.19) | Very Low^4^ |
| **347** | **YOG+PRB+ZN vs SB+LCF** | NA | NA | NA | NA | NA | -3.22  (-47.11; 41.52) | Very Low^4^ | 16/15/ | -3.22  (-47.11; 41.52) | Very Low^4^ |
| **348** | **YOG+PRB+ZN vs ZN+LCF** | NA | NA | NA | NA | NA | 5.82  (-37.26; 49.68) | Very Low^4^ | 17/15/ | 5.82  (-37.26; 49.68) | Very Low^4^ |
| **349** | **YOG+PRB+ZN vs SB+ZN+LCF** | NA | NA | NA | NA | NA | 1.07  (-43.8; 46.5) | Very Low^4^ | 18/15/ | 1.07  (-43.8; 46.5) | Very Low^4^ |
| **350** | **YOG+PRB+ZN vs SYM+LCF** | NA | NA | NA | NA | NA | 16.19  (-29.57; 63.54) | Very Low^4^ | 3/15/ | 16.19  (-29.57; 63.54) | Very Low^4^ |
| **351** | **YOG+PRB+ZN vs SM+ZN** | NA | NA | NA | NA | NA | 20.02  (-26.85; 66.97) | Very Low^4^ | 37/15/ | 20.02  (-26.85; 66.97) | Very Low^4^ |

+ This column displays the direct comparisons GRADE Assessments that informed the indirect estimates GRADE assessment. Numbers are showing the number ID of the direct comparison (which are all between 1-62)

**MD:** Mean difference; **95CrI%:** 95% Credible Intervals; Acronyms for interventions are detailed in table 2 in manuscript; **NMA:** Network Meta-analysis; NA: Non-applicable; In **Bold,** estimates that were statistically significant.

**RC:** Racecadotril; **SM:** Smectite, **LOP:** Loperamide; **ZN**: Zinc; **VA:** Vitamin A; **MN:** Micronutrients; **LGG:** *Lactobacillus rhamnosus* –GG; **SB:** *Saccharomyces boulardii*; **ALL-PRB**: **SYM:** Symbiotics, **LCF:** lactose-free formula; **YOG:** Yogurt; **PRE:** prebiotics; **DM:** Diluted Milk; **LGG+SM**: LGG+Smectite; **ZN+PRB:** Zinc + Probiotics; **STND:** Standard treatment or Placebo**; ZN+MN:** Zinc + micronutrients; **CAO:** Kaolin-Pectin; **LCF+PRB:** Lactose Free Formula +Probiotics; **SB+ZN:** *S. boulardii* + Zinc; **SB+LCF:** *S. boulardii* + Lactose-Free Formula; **ZN+LCF:** Zinc + Lactose Free Formula; **SB+ZN+LCF**: *S. boulardii* + Zinc + Lactose free Formula; **SYM+LCF:** Symbiotics + Lactose Free Formula; **SM+ZN:** Smectite+ Zinc; **YOG+PRB+ZN:** Yogurt + Probiotics + Zinc

**GRADE Assessment:** Reasons for downgrading direct evidence(1 to 5), indirect (6) and Mixed estimates(4, 7): 1. Downgraded because of Risk of Bias; 2. Downgraded because of Inconsistency; 3. Downgraded because of Indirectness; 4. Downgraded because of Imprecision; 5. Downgraded because of Publication Bias; 6. Downgraded because of Intransitivity; 7. Downgraded because of Incoherence; NOTE: When a superscript is more than one for a estimate, means that the criterion was downgraded 2 points (-2), instead on one point (-1)

# Table F: Assessment of Incoherence for indirect comparisons

| **Comparison** | **Z-value** | **P- value** |
| --- | --- | --- |
| STND vs SYM | 0.558 | 0.577 |
| DM vs LCF | 0.030 | 0.976 |
| STND vs LCF | 0.859 | 0.390 |
| SB+ZN vs LCF | 0.325 | 0.745 |
| SB+LCF vs LCF | 0.065 | 0.948 |
| ZN+LCF vs LCF | 0.461 | 0.644 |
| SB+ZN+LCF vs LCF | 0.065 | 0.948 |
| STND vs YOG | 0.226 | 0.821 |
| STND vs DM | 0.097 | 0.923 |
| STND vs ZN+PRB | 0.625 | 0.532 |
| ZN+MN vs STND | 0.074 | 0.941 |
| SB+ZN vs STND | 0.628 | 0.530 |
| SB+LCF vs STND | 0.376 | 0.707 |
| ZN+LCF vs STND | 0.664 | 0.506 |
| SB+ZN+LCF vs STND | 0.376 | 0.707 |
| STND vs RC | -0.710 | 0.481 |
| LOP vs RC | 0.000 | 0.999 |
| SB+LCF vs SB+ZN | 0.625 | 0.532 |
| ZN+LCF vs SB+ZN | 1.618 | 0.106 |
| SB+ZN+LCF vs SB+ZN | 0.625 | 0.532 |
| ZN+LCF vs SB+LCF | 1.890 | 0.059 |
| SB+ZN+LCF vs ZN+LCF | 1.890 | 0.059 |
| STND vs SM | -0.370 | 0.708 |
| LOP vs SM | 0.23 | 0.817 |
| All-PRB vs SM | -0.370 | 0.708 |
| STND vs LOP | 0.170 | 0.869 |
| SYM vs ZN | -0.570 | 0.569 |
| LCF vs ZN | -0.710 | 0.476 |
| ZN+PRB vs ZN | -0.080 | 0.934 |
| STND vs ZN | 1.070 | 0.285 |
| ZN+MN vs ZN | 0.180 | 0.857 |
| SB+ZN vs ZN | -1.810 | 0.071 |
| SB+LCF vs ZN | -1.530 | 0.126 |
| ZN+LCF vs ZN | 0.741 | 0.458 |
| SB+ZN+LCF vs ZN | 1.530 | 0.126 |
| LGG vs ZN | -0.680 | 0.496 |
| SB vs ZN | -1.280 | 0.201 |
| ZN+MN vs MN |  | 0.857 |
| YOG vs LGG | 0.226 | 0.821 |
| STND vs LGG | 0.203 | 0.839 |
| SB vs LGG | 0.858 | 0.391 |
| All-PRB vs LGG | 0.917 | 0.359 |
| LCF vs SB | 0.326 | 0.744 |
| STND vs SB | 0.580 | 0.562 |
| SB+ZN vs SB | 0.698 | 0.485 |
| SB+LCF vs SB | 0.452 | 0.651 |
| ZN+LCF vs SB | 0.686 | 0.493 |
| SB+ZN+LCF vs SB | 0.452 | 0.651 |
| All-PRB vs SB | 0.356 | 0.722 |
| ZN+PRB vs All-PRB | 0.716 | 0.474 |
| STND vs All-PRB | 0.609 | 0.543 |
| Whole Network |  | 0.83 |

Incoherence between the direct and indirect estimates in the network was evaluated using both the global test random-effects design-by-treatment interaction model (Veroniki 2013) for the whole network and with the node-splitting method (Dias 2010) for local assessment.

# Table G: Sensitivity analyses and SUCRA Values for Diarrhea duration

| **Intervention** | **All Studies** | **RCTs** | **Blinding** | **Alloc concealment** |
| --- | --- | --- | --- | --- |
| **RC** | 0.46 (0.23; 0.73) | 0.39 (0.17; 0.70) | 0.46 (0.00; 0.92) | 0.19 (0.00; 0.50) |
| **SM** | 0.69 (0.42; 0.88) | 0.78 (0.48; 0.91) | 0.85 (0.38; 1.00) | 0.81 (0.56; 1.00) |
| **LOP** | 0.46 (0.15; 0.85) | 0.43 (0.13; 0.83) | 0.46 (0.08; 0.92) | 0.56 (0.19; 0.94) |
| **ZN** | 0.5 (0.27; 0.69) | 0.52 (0.30; 0.74) | 0.54 (0.31; 0.77) | 0.38 (0.19; 0.56) |
| **VA** | 0.19 (0.00; 0.61) | 0.17 (0.00; 0.65) | NA | 0.25 (0.00; 0.88) |
| **MN** | 0.08 (0.00; 0.85) | 0.09 (0.00; 0.87) | 0.08 (0.00; 0.85) | 0.06 (0.00; 0.81) |
| **LGG** | 0.65 (0.38; 0.85) | 0.70 (0.43; 0.87) | 0.31 (0.00; 0.69) | 0.94 (0.69; 1.00) |
| **SB** | 0.42 (0.19; 0.69) | 0.39 (0.17; 0.70) | NA | 0.19 (0.00; 0.56) |
| **All-PRB** | 0.54 (0.31; 0.73) | 0.57 (0.35; 0.74) | 0.62 (0.31; 0.85) | 0.56 (0.38; 0.81) |
| **SYM** | 0.77 (0.38; 0.92) | 0.78 (0.43; 0.91) | 0.85 (0.38; 1.00) | 0.88 (0.25; 1.00) |
| **LCF** | 0.31 (0.15; 0.54) | 0.30 (0.13; 0.52) | 1.00 (0.38; 1.00) | 0.69 (0.25; 1.00) |
| **YOG** | 0.42 (0.11; 0.85) | 0.35 (0.04; 0.87) | NA | NA |
| **PRE** | 0.38 (0.00; 0.96) | 0.43 (0.00; 0.96) | NA | 0.56 (0.00; 1.00) |
| **DM** | 0.04 (0.00; 0.23) | 0.04 (0.00; 0.22) | 0.23 (0.00; 0.85) | 0.19 (0.00; 0.75) |
| **LGG+SM** | 1.00 (0.92; 1.00) | 1.00 (0.91; 1.00) | 0.31 (0.00; 0.85) | NA |
| **ZN+PRB** | 0.81 (0.5; 0.96) | NA | 0.23 (0.00; 1.00) | 0.75 (0.38; 1.00) |
| **LA+LCF+ZN** | NA | 0.87 (0.52; 0.96) | NA | NA |
| **STND** | 0.08 (0.00; 0.19) | 0.09 (0.00; 0.17) | 0.15 (0.00; 0.38) | 0.13 (0.00; 0.31) |
| **ZN+MN** | 0.46 (0.15; 0.85) | 0.52 (0.13; 0.87) | 0.77 (0.38; 0.92) | 0.56 (0.25; 0.88) |
| **CAO** | 0.15 (0.00; 0.89) | NA | NA | NA |
| **LCF+PRB** | 0.31 (0.00; 0.88) | 0.30 (0.00; 0.91) | NA | NA |
| **SB+ZN** | 0.92 (0.77; 1.00) | 0.96 (0.78; 1.00) | NA | 0.75 (0.19; 1.00) |
| **SB+LCF** | 0.27 (0.04; 0.81) | 0.30 (0.00; 0.83) | NA | NA |
| **ZN+LCF** | 0.61 (0.19; 0.92) | 0.61 (0.17; 0.91) | NA | NA |
| **SB+ZN+LCF** | 0.42 (0.08; 0.88) | 0.43 (0.04; 0.91) | NA | NA |
| **SYM+LCF** | 0.85 (0.27; 1.00) | 0.87 (0.26; 1.00) | NA | NA |
| **SM+ZN** | 0.88 (0.35; 1.00) | NA | NA | NA |
| **YOG+PRB+ZN** | 0.38 (0.00; 1.00) | NA | NA | NA |
| Heterogeneity (Tau^2^) | 99.60  (82.15;119.70) | 99.34  (82.09;119.90) | 28.33  (17.30; 2.96) | 49.67  (36.08;66.88) |

**All Studies:** Analysis based on al the studies that reported the outcome. **RCTs:** Analysis based on the exclusion of quasi-RCTs; **Blinding:** Based on the exclusion of the studies with High risk of bias for blinding; **Alloc: Concealment:** Analysis based on the exclusion of studies with High RoB for Allocation concealment; Global heterogeneity assessed with the common within-network between-study variance (Tau2; 95%CrI). **RC:** racecadotril; **SM:** Smectite, **LOP:** Loperamide; **ZN**: Zinc; **VA:** Vitamin A; **MN:** Micronutrients; **LGG:** *Lactobacillus rhamnosus* –GG; **SB:** *Sacharomyces boulardii*; **ALL-PRB**: **SYM:** Symbiotics, **LCF:** lactose-free formula; **YOG:** Yogurt; **PRE:** prebiotics; **DM:** Diluted Milk; **LGG+SM**: LGG+Smectite; **ZN+PRB:** Zinc + Probiotics; **STND:** Standard treatment or Placebo**; ZN+MN:** Zinc + micronutrients; **CAO:** Kaolin-Pectin; **LCF+PRB:** Lactose Free Formula +Probiotics; **SB+ZN:** *S. boulardii* + Zinc; **SB+LCF:** *S. boulardii* + Lactose Free Formula; **ZN+LCF:** Zinc + Lactose Free Formula; **SB+ZN+LCF**: *S. boulardii* + Zinc + Lactose free Formula; **SYM+LCF:** Symbiotics + Lactose Free Formula; **SM+ZN:** Smectite+ Zinc; **YOG+PRB+ZN:** Yogurt + Probiotics + Zinc

# Table H: Subgroup analyses and SUCRA values for each intervention for Diarrhea duration

| **Intervention** | **All Studies** | **Country income Classification** | | **Clinical Setting** | |
| --- | --- | --- | --- | --- | --- |
|  |  | **LMIC** | **HIC** | **Inpatients** | **Outpatients** |
| **RC** | 0.46 (0.23; 0.73) | 0.43 (0.14; 0.71) | 0.54 (0.23; 0.85) | 0.33 (0.14; 0.67) | 0.18 (0.09; 0.45) |
| **SM** | 0.69 (0.42; 0.88) | 0.81 (0.52; 0.95) | 0.62 (0.08; 0.92) | 0.81 (0.52; 0.95) | NA |
| **LOP** | 0.46 (0.15; 0.85) | 0.71 (0.05; 1.00) | 0.54 (0.15; 0.85) | NA | 0.36 (0.09; 0.64) |
| **ZN** | 0.5 (0.27; 0.69) | 0.62 (0.43; 0.81) | 0.00 (0.00; 0.15) | 0.81 (0.67; 0.90) | 0.36 (0.09; 0.64) |
| **VA** | 0.19 (0.00; 0.61) | 0.19 (0.00; 0.67) | NA | 0.14 (0.00; 0.62) | NA |
| **MN** | 0.08 (0.00; 0.85) | 0.05 (0.00; 0.86) | NA | NA | NA |
| **LGG** | 0.65 (0.38; 0.85) | 0.33 (0.14; 0.62) | 0.92 (0.77; 0.92) | 0.48 (0.24; 0.71) | 0.73 (0.64; 0.91) |
| **SB** | 0.42 (0.19; 0.69) | 0.48 (0.19; 0.76) | 0.31 (0.15; 0.77) | 0.48 (0.19; 0.76) | 0.27 (0.09; 0.45) |
| **All-PRB** | 0.54 (0.31; 0.73) | 0.62 (0.43; 0.81) | 0.54 (0.31; 0.77) | 0.62 (0.38; 0.76) | 0.55 (0.27; 0.64) |
| **SYM** | 0.77 (0.38; 0.92) | 0.86 (0.52; 1.00) | 0.69 (0.23; 0.92) | 0.90 (0.57; 1.00) | 0.64 (0.27; 0.73) |
| **LCF** | 0.31 (0.15; 0.54) | 0.19 (0.05; 0.43) | 0.46 (0.23; 0.77) | 0.48 (0.24; 0.71) | 0.18 (0.00; 0.91) |
| **YOG** | 0.42 (0.11; 0.85) | 0.52 (0.05; 0.95) | 0.77 (0.23; 0.92) | 0.14 (0.00; 0.67) | NA |
| **PRE** | 0.38 (0.00; 0.96) | NA | NA | 0.43 (0.00; 0.95) | NA |
| **DM** | 0.04 (0.00; 0.23) | 0.00 (0.00; 0.57) | 0.08 (0.00; 0.15) | 0.05 (0.00; 0.19) | NA |
| **LGG+SM** | 1.00 (0.92; 1.00) | NA | 1.00 (1.00; 1.00) | 0.48 (0.10; 0.86) | NA |
| **ZN+PRB** | 0.81 (0.5; 0.96) | 0.95 (0.76; 1.00) | 0.46 (0.15; 0.85) | 0.43 (0.05; 0.90) | 0.82 (0.73; 0.91) |
| **LA+LCF+ZN** | NA | NA | NA | NA | NA |
| **STND** | 0.08 (0.00; 0.19) | 0.05 (0.00; 0.19) | 0.15 (0.08; 0.23) | 0.10 (0.00; 0.19) | 0.00 (0.00; 0.09) |
| **ZN+MN** | 0.46 (0.15; 0.85) | 0.57 (0.19; 0.86) | NA | 0.81 (0.38; 0.95) | NA |
| **CAO** | 0.15 (0.00; 0.89) | NA | NA | NA | NA |
| **LCF+PRB** | 0.31 (0.00; 0.88) | 0.24 (0.00; 0.86) | NA | 0.48 (0.10; 0.90) | NA |
| **SB+ZN** | 0.92 (0.77; 1.00) | 0.95 (0.81; 1.00) | NA | 1.00 (0.86; 1.00) | 0.91 (0.55; 1.00) |
| **SB+LCF** | 0.27 (0.04; 0.81) | 0.33 (0.00; 0.81) | NA | 0.33 (0.05; 0.76) | NA |
| **ZN+LCF** | 0.61 (0.19; 0.92) | 0.57 (0.19; 0.90) | NA | 0.24 (0.00; 0.67) | 1.00 (0.91; 1.00) |
| **SB+ZN+LCF** | 0.42 (0.08; 0.88) | 0.48 (0.05; 0.90) | NA | 0.52 (0.10; 0.90) | NA |
| **SYM+LCF** | 0.85 (0.27; 1.00) | 0.81 (0.24; 1.00) | NA | 0.90 (0.62; 1.00) | NA |
| **SM+ZN** | 0.88 (0.35; 1.00) | NA | NA | NA | NA |
| **YOG+PRB+ZN** | 0.38 (0.00; 1.00) | NA | NA | NA | NA |
| Heterogeneity (Tau^2^) | 99.60  (82.15;119,70) | 79.33  (63.30; 99.05) | 43.67  (31.48; 59.53) | 53.59  (40.60; 69.79) | **2.52**  **(0.00; 14.38)** |

**All Studies:** Analysis based on all the studies that reported the outcome. **LMIC:** Subgroup of studies conducted in low and middle-income countries; **HIC:** Subgroup of studies conducted in high-income countries; **Inpatients:**  Subgroup of studies conducted in in inpatients; **Outpatients:** Subgroup of studies conducted in outpatients; Global heterogeneity assessed with the common within-network between-study variance (Tau2; 95%CrI). **RC:** racecadotril; **SM:** Smectite, **LOP:** Loperamide; **ZN**: Zinc; **VA:** Vitamin A; **MN:** Micronutrients; **LGG:** *Lactobacillus rhamnosus* –GG; **SB:** *Sacharomyces boulardii*; **ALL-PRB**: **SYM:** Symbiotics, **LCF:** lactose-free formula; **YOG:** Yogurt; **PRE:** prebiotics; **DM:** Diluted Milk; **LGG+SM**: LGG+Smectite; **ZN+PRB:** Zinc + Probiotics; **STND:** Standard treatment or Placebo**; ZN+MN:** Zinc + micronutrients; **CAO:** Kaolin-Pectin; **LCF+PRB:** Lactose Free Formula +Probiotics; **SB+ZN:** *S. boulardii* + Zinc; **SB+LCF:** *S. boulardii* + Lactose Free Formula; **ZN+LCF:** Zinc + Lactose Free Formula; **SB+ZN+LCF**: *S. boulardii* + Zinc + Lactose free Formula; **SYM+LCF:** Symbiotics + Lactose Free Formula; **SM+ZN:** Smectite+ Zinc; **YOG+PRB+ZN:** Yogurt + Probiotics + Zinc

# Fig A: NMA Forest Plots of interventions vs STND for subgroups analyses of all RCTs vs LMIC*

All-PRB vs STND

All-PRB vs STND

DM vs STND

DM vs STND

LA+LCF+ZN vs STND

LCF vs STND

LCF vs STND

LCF+PRB vs STND

LCF+PRB vs STND

LGG vs STND

LGG vs STND

LGG+SM vs STND

LOP vs STND

LOP vs STND

MN vs STND

MN vs STND

PRE vs STND

RC vs STND

RC vs STND

SB vs STND

SB vs STND

SB+LCF vs STND

SB+LCF vs STND

SB+ZN vs STND

SB+ZN vs STND

SB+ZN+LCF vs STND

SB+ZN+LCF vs STND

SM vs STND

SM vs STND

SYM vs STND

SYM vs STND

SYM+LCF vs STND

SYM+LCF vs STND

VA vs STND

VA vs STND

YOG vs STND

YOG vs STND

ZN vs STND

ZN vs STND

ZN+LCF vs STND

ZN+LCF vs STND

ZN+MN vs STND

ZN+MN vs STND

ZN+PRB vs STND

-19.37 (-23.66,-15.07)

-20.46 (-25.17,-15.84)

-3.74 (-19.20,11.51)

3.15 (-8.17,14.28)

-30.15 (-42.25,-18.06)

-6.68 (-15.02,1.67)

-11.40 (-18.39,-4.53)

-12.19 (-35.22,9.92)

-7.75 (-29.16,13.08)

-22.45 (-28.51,-16.44)

-11.72 (-19.72,-3.80)

-50.93 (-64.13,-37.70)

-15.64 (-28.16,-3.32)

-22.01 (-45.83,2.01)

0.98 (-32.49,33.02)

0.09 (-31.23,31.39)

-15.35 (-42.74,12.15)

-14.30 (-22.37,-6.32)

-13.90 (-22.23,-5.85)

-15.35 (-23.74,-7.11)

-14.92 (-22.33,-7.38)

-10.72 (-27.80,5.70)

-11.59 (-29.53,6.39)

-38.93 (-51.86,-26.09)

-39.74 (-51.90,-27.34)

-15.32 (-33.91,2.98)

-15.97 (-35.46,3.45)

-25.32 (-33.74,-17.01)

-26.32 (-34.42,-18.25)

-28.35 (-39.35,-17.32)

-26.19 (-36.38,-16.14)

-26.28 (-45.82,-7.08)

-30.86 (-51.67,-10.16)

-5.99 (-20.51,8.46)

-5.89 (-21.48,9.42)

-17.67 (-39.00,3.93)

-13.96 (-30.38,2.54)

-18.03 (-23.21,-12.90)

-20.04 (-24.98,-15.04)

-20.80 (-35.80,-5.87)

-19.00 (-33.54,-4.93)

-18.38 (-30.91,-5.66)

-17.70 (-31.57,-3.73)

-37.51 (-50.76,-24.57)

**MD 95%CI**

**Treatment Comparison**

-60

-40

-20

0

20

40

**Group**

All RCTs

LMIC

STND better

Treatment better

**All subgroup analyses were performed with RCTs*

Each intervention has been compared to STND treatment (Placebo, standard or no treatment), MD: Mean difference; 95%CI: 95% credible interval; HIC: High income Countries; LMIC: Low- and Middle-Income countries; **Interventions:** **RC:** racecadotril; **SM:** Smectite, **LOP:** Loperamide; **ZN**: Zinc; **VA:** Vitamin A; **MN:** Micronutrients; **LGG:** *Lactobacillus rhamnosus* –GG; **SB:** *Saccharomyces boulardii*; **ALL-PRB**: **SYM:** Symbiotics, **LCF:** lactose-free formula; **YOG:** Yogurt; **PRE:** prebiotics; **DM:** Diluted Milk; **LGG+SM**: LGG+Smectite; **ZN+PRB:** Zinc + Probiotics; **STND:** Standard treatment or Placebo**; ZN+MN:** Zinc + micronutrients; **CAO:** Kaolin-Pectin; **LCF+PRB:** Lactose Free Formula +Probiotics; **SB+ZN:** *S. boulardii* + Zinc; **SB+LCF:** *S. boulardii* + Lactose Free Formula; **ZN+LCF:** Zinc + Lactose Free Formula; **SB+ZN+LCF**: *S. boulardii* + Zinc + Lactose free Formula; **SYM+LCF:** Symbiotics + Lactose Free Formula; **SM+ZN:** Smectite+ Zinc; **YOG+PRB+ZN:** Yogurt + Probiotics + Zinc

# Fig B: NMA Forest Plots of interventions vs STND for subgroups analyses of all RCTs vs HIC*

**All subgroup analyses were performed with RCTs*

Each intervention has been compared to STND treatment (Placebo, standard or no treatment), MD: Mean difference; 95%CI: 95% credible interval; HIC: High income Countries; LMIC: Low- and Middle-Income countries; **Interventions:** **RC:** racecadotril; **SM:** Smectite, **LOP:** Loperamide; **ZN**: Zinc; **VA:** Vitamin A; **MN:** Micronutrients; **LGG:** *Lactobacillus rhamnosus* –GG; **SB:** *Saccharomyces boulardii*; **ALL-PRB**: **SYM:** Symbiotics, **LCF:** lactose-free formula; **YOG:** Yogurt; **PRE:** prebiotics; **DM:** Diluted Milk; **LGG+SM**: LGG+Smectite; **ZN+PRB:** Zinc + Probiotics; **STND:** Standard treatment or Placebo**; ZN+MN:** Zinc + micronutrients; **CAO:** Kaolin-Pectin; **LCF+PRB:** Lactose Free Formula +Probiotics; **SB+ZN:** *S. boulardii* + Zinc; **SB+LCF:** *S. boulardii* + Lactose Free Formula; **ZN+LCF:** Zinc + Lactose Free Formula; **SB+ZN+LCF**: *S. boulardii* + Zinc + Lactose free Formula; **SYM+LCF:** Symbiotics + Lactose Free Formula; **SM+ZN:** Smectite+ Zinc; **YOG+PRB+ZN:** Yogurt + Probiotics + Zinc

# Fig C: NMA Forest Plots interventions vs STND for subgroups analyses all RCTs vs Inpatients*

**All subgroup analyses were performed with RCTs*

Each intervention has been compared to STND treatment (Placebo, standard or no treatment), MD: Mean difference; 95%CI: 95% credible interval; HIC: High income Countries; LMIC: Low- and Middle-Income countries; **Interventions:** **RC:** racecadotril; **SM:** Smectite, **LOP:** Loperamide; **ZN**: Zinc; **VA:** Vitamin A; **MN:** Micronutrients; **LGG:** *Lactobacillus rhamnosus* –GG; **SB:** *Saccharomyces boulardii*; **ALL-PRB**: **SYM:** Symbiotics, **LCF:** lactose-free formula; **YOG:** Yogurt; **PRE:** prebiotics; **DM:** Diluted Milk; **LGG+SM**: LGG+Smectite; **ZN+PRB:** Zinc + Probiotics; **STND:** Standard treatment or Placebo**; ZN+MN:** Zinc + micronutrients; **CAO:** Kaolin-Pectin; **LCF+PRB:** Lactose Free Formula +Probiotics; **SB+ZN:** *S. boulardii* + Zinc; **SB+LCF:** *S. boulardii* + Lactose Free Formula; **ZN+LCF:** Zinc + Lactose Free Formula; **SB+ZN+LCF**: *S. boulardii* + Zinc + Lactose free Formula; **SYM+LCF:** Symbiotics + Lactose Free Formula; **SM+ZN:** Smectite+ Zinc; **YOG+PRB+ZN:** Yogurt + Probiotics + Zinc

# Fig D: NMA Forest Plots interventions vs STND for subgroups analyses all RCTs vs outpatients*

All-PRB vs STND

All-PRB vs STND

DM vs STND

LA+LCF+ZN vs STND

LCF vs STND

LCF vs STND

LCF+PRB vs STND

LGG vs STND

LGG vs STND

LGG+SM vs STND

LOP vs STND

LOP vs STND

MN vs STND

PRE vs STND

RC vs STND

RC vs STND

SB vs STND

SB vs STND

SB+LCF vs STND

SB+ZN vs STND

SB+ZN vs STND

SB+ZN+LCF vs STND

SM vs STND

SYM vs STND

SYM vs STND

SYM+LCF vs STND

VA vs STND

YOG vs STND

ZN vs STND

ZN vs STND

ZN+LCF vs STND

ZN+LCF vs STND

ZN+MN vs STND

ZN+PRB vs STND

-19.37 (-23.66,-15.07)

-16.10 (-21.87,-10.38)

3.15 (-8.17,14.28)

-30.15 (-42.25,-18.06)

-11.40 (-18.39,-4.53)

-8.47 (-35.25,17.86)

-12.19 (-35.22,9.92)

-22.45 (-28.51,-16.44)

-26.98 (-33.19,-20.34)

-50.93 (-64.13,-37.70)

-15.64 (-28.16,-3.32)

-10.84 (-18.54,-3.46)

0.98 (-32.49,33.02)

-15.35 (-42.74,12.15)

-14.30 (-22.37,-6.32)

-8.03 (-15.31,-1.09)

-14.92 (-22.33,-7.38)

-8.84 (-14.10,-3.36)

-11.59 (-29.53,6.39)

-31.40 (-46.80,-16.39)

-38.93 (-51.86,-26.09)

-15.97 (-35.46,3.45)

-25.32 (-33.74,-17.01)

-26.19 (-36.38,-16.14)

-18.00 (-25.96,-10.02)

-30.86 (-51.67,-10.16)

-5.89 (-21.48,9.42)

-13.96 (-30.38,2.54)

-12.42 (-18.38,-6.55)

-18.03 (-23.21,-12.90)

-53.19 (-77.28,-29.38)

-20.80 (-35.80,-5.87)

-17.70 (-31.57,-3.73)

-29.85 (-37.91,-22.04)

**MD 95%CI**

**Treatment Comparison**

-80

-60

-40

-20

0

20

40

**Group**

All RCTs

Outpatients

STND better

Treatment better

**All subgroup analyses were performed with RCTs*

Each intervention has been compared to STND treatment (Placebo, standard or no treatment), MD: Mean difference; 95%CI: 95% credible interval; **RCTs:** Randomized controlled tirals; **Interventions:** **RC:** racecadotril; **SM:** Smectite, **LOP:** Loperamide; **ZN**: Zinc; **VA:** Vitamin A; **MN:** Micronutrients; **LGG:** *Lactobacillus rhamnosus* –GG; **SB:** *Saccharomyces boulardii*; **ALL-PRB**: **SYM:** Symbiotics, **LCF:** lactose-free formula; **YOG:** Yogurt; **PRE:** prebiotics; **DM:** Diluted Milk; **LGG+SM**: LGG+Smectite; **ZN+PRB:** Zinc + Probiotics; **STND:** Standard treatment or Placebo**; ZN+MN:** Zinc + micronutrients; **CAO:** Kaolin-Pectin; **LCF+PRB:** Lactose Free Formula +Probiotics; **SB+ZN:** *S. boulardii* + Zinc; **SB+LCF:** *S. boulardii* + Lactose Free Formula; **ZN+LCF:** Zinc + Lactose Free Formula; **SB+ZN+LCF**: *S. boulardii* + Zinc + Lactose free Formula; **SYM+LCF:** Symbiotics + Lactose Free Formula; **SM+ZN:** Smectite+ Zinc; **YOG+PRB+ZN:** Yogurt + Probiotics + Zinc

# Table I: Meta-regression analyses and SUCRA values for each intervention for Diarrhea duration

| **Intervention** | **All Studies** | **Year** | **Age** | **Days** | **Etiology** |
| --- | --- | --- | --- | --- | --- |
| **RC** | 0.46 (0.23; 0.73) | 0.43 (0.14; 0.71) | 0.32 (0.16; 0.63) | 0.55 (0.23; 0.82) | 0.43 (0.17; 0.78) |
| **SM** | 0.69 (0.42; 0.88) | 0.78 (0.43; 0.91) | 0.74 (0.42; 0.89) | 0.82 (0.45; 0.95) | 0.43 (0.17; 0.78) |
| **LOP** | 0.46 (0.15; 0.85) | 0.43 (0.13; 0.83) | 0.47 (0.16; 0.84) | 0.59 (0.14; 0.91) | 0.39 (0.13; 0.78) |
| **ZN** | 0.5 (0.27; 0.69) | 0.57 (0.35; 0.78) | 0.42 (0.21; 0.58) | 0.50 (0.27; 0.68) | 0.57 (0.30; 0.78) |
| **VA** | 0.19 (0.00; 0.61) | 0.17 (0.00; 0.57) | 0.11 (0.00; 0.68) | 0.14 (0.00; 0.73) | 0.13 (0.00; 0.87) |
| **MN** | 0.08 (0.00; 0.85) | 0.04 (0.00; 0.83) | NA | 0.09 (0.00; 0.86) | 0.09 (0.00; 0.83) |
| **LGG** | 0.65 (0.38; 0.85) | 0.70 (0.43; 0.87) | 0.74 (0.53; 0.89) | 0.68 (0.45; 0.82) | 0.61 (0.35; 0.83) |
| **SB** | 0.42 (0.19; 0.69) | 0.43 (0.22; 0.74) | 0.42 (0.16; 0.74) | 0.32 (0.14; 0.59) | 0.52 (0.17; 0.83) |
| **All-PRB** | 0.54 (0.31; 0.73) | 0.61 (0.39; 0.78) | 0.58 (0.37; 0.74) | 0.59 (0.36; 0.77) | 0.65 (0.43; 0.83) |
| **SYM** | 0.77 (0.38; 0.92) | 0.83 (0.48; 0.96) | 0.68 (0.26; 0.89) | 0.82 (0.50; 0.95) | 0.83 (0.26; 1.00) |
| **LCF** | 0.31 (0.15; 0.54) | 0.22 (0.13; 0.43) | 0.26 (0.11; 0.53) | 0.45 (0.23; 0.73) | 0.30 (0.13; 0.57) |
| **YOG** | 0.42 (0.11; 0.85) | 0.43 (0.09; 0.87) | 0.37 (0.11; 0.84) | 0.27 (0.00; 0.86) | 0.22 (0.00; 0.83) |
| **PRE** | 0.38 (0.00; 0.96) | 0.39 (0.00; 0.96) | 0.37 (0.00; 0.89) | 0.45 (0.00; 0.95) | 0.39 (0.00; 0.91) |
| **DM** | 0.04 (0.00; 0.23) | 0.04 (0.00; 0.17) | 0.00 (0.00; 0.16) | 0.14 (0.00; 0.50) | 0.13 (0.00; 0.43) |
| **LGG+SM** | 1.00 (0.92; 1.00) | 1.00 (0.96; 1.00) | 0.95 (0.95; 1.00) | 1.00 (0.91; 1.00) | 1.00 (0.87; 1.00) |
| **ZN+PRB** | 0.81 (0.5; 0.96) | 0.83 (0.48; 0.96) | 0.79 (0.42; 0.89) | 0.73 (0.32; 0.95) | 0.91 (0.52; 1.00) |
| **LA+LCF+ZN** | NA | NA | NA | NA | NA |
| **STND** | 0.08 (0.00; 0.19) | 0.09 (0.04; 0.22) | 0.05 (0.00; 0.16) | 0.09 (0.00; 0.23) | 0.09 (0.00; 0.17) |
| **ZN+MN** | 0.46 (0.15; 0.85) | 0.48 (0.17; 0.87) | 0.47 (0.11; 0.84) | 0.50 (0.18; 0.86) | 0.52 (0.17; 0.83) |
| **CAO** | 0.15 (0.00; 0.89) | NA | NA | NA | NA |
| **LCF+PRB** | 0.31 (0.00; 0.88) | 0.22 (0.00; 0.87) | 0.26 (0.00; 0.89) | NA | 0.30 (0.00; 0.87) |
| **SB+ZN** | 0.92 (0.77; 1.00) | 0.96 (0.78; 1.00) | 0.84 (0.37; 0.95) | 0.91 (0.68; 1.00) | 0.91 (0.52;1.00) |
| **SB+LCF** | 0.27 (0.04; 0.81) | 0.30 (0.04; 0.83) | NA | 0.23 (0.00; 0.73) | 0.30; 0.04; 0.83) |
| **ZN+LCF** | 0.61 (0.19; 0.92) | 0.57 (0.17; 0.91) | 1.00 (0.84; 1.00) | 0.14 (0.00; 0.59) | 0.65; 0.17; 0.87) |
| **SB+ZN+LCF** | 0.42 (0.08; 0.88) | 0.39 (0.04; 0.91) | NA | 0.36 (0.05; 0.86) | 0.48; 0.09; 0.88) |
| **SYM+LCF** | 0.85 (0.27; 1.00) | 0.83 (0.22; 0.96) | NA | 0.91 (0.45; 1.00) | 0.87; 0.30; 1.00) |
| **SM+ZN** | 0.88 (0.35; 1.00) | NA | NA | NA | NA |
| **YOG+PRB+ZN** | 0.38 (0.00; 1.00) | NA | NA | NA | NA |
| Tau2 (95%CrI) | 99.60  (82.15;119,70) | 99.04  (82.05; 119.60) | 89.72  (72.99; 109.80) | 89.72  (72.99; 109.80) | 84.04  (67.95; 103.50) |
| ß Coefficient (95%CrI) | NA | **-0.35 (-0.6;-0.05)** | -0.10 (-0.30;0.09) | -0.32 (-3.4; 2.77) | -0.33 (-0.74;0.07) |

**Analyses based on year of publication, age, days with diarrhea and etiology (% patients with rotavirus infection). All Studies:** Analysis based on al the studies that reported the outcome. **Year:** metaregression based on year of publication of the study; **Age:** metaregression based on age of children; **Days:** metaregression based on days with diarrhea at the time of recruiting; **Etiology:** metaregression based on proportion of children who had rotavirus; Global heterogeneity assessed with the common within-network between-study variance (Tau2; 95%CrI); ß Coefficient with 95%CrI. **RC:** Racecadotril; **SM:** Smectite, **LOP:** Loperamide; **ZN**: Zinc; **VA:** Vitamin A; **MN:** Micronutrients; **LGG:** *Lactobacillus rhamnosus* –GG; **SB:** *Sacharomyces boulardii*; **ALL-PRB**: All probiotics (except LGG and SB); **SYM:** Symbiotics, **LCF:** lactose-free formula; **YOG:** Yogurt; **PRE:** prebiotics; **DM:** Diluted Milk; **LGG+SM**: LGG+Smectite; **ZN+PRB:** Zinc + Probiotics; **STND:** Standard treatment or Placebo**; ZN+MN:** Zinc + micronutrients; **CAO:** Kaolin-Pectin; **LCF+PRB:** Lactose Free Formula +Probiotics; **SB+ZN:** *S. boulardii* + Zinc; **SB+LCF:** *S. boulardii* + Lactose Free Formula; **ZN+LCF:** Zinc + Lactose Free Formula; **SB+ZN+LCF**: *S. boulardii* + Zinc + Lactose free Formula; **SYM+LCF:** Symbiotics + Lactose Free Formula; **SM+ZN:** Smectite+ Zinc; **YOG+PRB+ZN:** Yogurt + Probiotics + Zinc

# Fig E: NMA Forest plots: interventions vs. STND for Stool Frequency at day 2

**– # 4348 patients, # 12 treatments, # 33 studies–**

**MD:** Mean difference; **95%CI:** 95% Credible Interval**; RC:** Racecadotril; **SM:** Smectite, **LOP:** Loperamide; **ZN**: Zinc; **VA:** Vitamin A; **MN:** Micronutrients; **LGG:** *Lactobacillus rhamnosus* –GG; **SB:** *Sacharomyces boulardii*; **ALL-PRB**: All probiotics (except LGG and SB); **SYM:** Symbiotics, **LCF:** Lactose-free formula; **YOG:** Yogurt; **PRE:** Prebiotics; **DM:** Diluted Milk; **LGG+SM**: LGG+Smectite; **ZN+PRB:** Zinc + Probiotics; **STND:** Standard treatment or Placebo**; CAO:** Kaolin-Pectin.

# Table J: Direct, Indirect and NMA estimates for Stool frequency at day 2 and GRADE Assessment

| **#** | **Comparison** | **Direct Estimates** OR(95%CrI) | -I^2^ | **Number of studies** | **Number of patients** | **Direct GRADE** | **Indirect**  **Estimates**  OR(95%CrI) | **Indirect GRADE** | **+ Direct GRADE that informs Indirect** | **NMA estimates**  OR(95%CrI) | **NMA GRADE** |
| --- | --- | --- | --- | --- | --- | --- | --- | --- | --- | --- | --- |
| **1** | **STND vs ZN+PRB** | 0.30 (-0.38; 0.99) | -- | 1 | 65 | Low^1,4^ | 0.89 (-0.71; 2.49) | Low^4^ | 7/6 | 0.89 (-0.71; 2.49) | Low^4^ |
| **2** | **STND vs RC** | 0.64 (-0.92; 2.18) | 21% | 2 | 163 | Low^1,1^ | 0.73  (1.53; 2.99) | Low | 7/4 | 0.60  (-0.69; 1.88) | Very Low^4^ |
| **3** | **CAO vs RC** | **3.28 (2.8; 3.76)** | **--** | 1 | 50 | Low^1,1^ | 0.60  (-0.69; 1.88) | Low^1,1^ | __ | 0.60  (-0.69; 1.88) | Low^1,1^ |
| **4** | **ZN vs RC** | -0.01 (-1.22; 1.2) | **--** | 1 | 120 | Low^1,1^ | 0.08  (-1.56; 1.73) | Very Low^6^ | 7/2 | 0.10  (-1.40; 1.58) | Very Low^4^ |
| **5** | **STND vs SM** | 1.01 (-1.08; 3.05) | **--** | 1 | 35 | Very Low^1,1,4^ | 0.98  (-1.90; 3.85) | Very Low^1,1,4^ | __ | 0.98  (-1.90; 3.85) | Very Low^1,1,4^ |
| **6** | **ZN+PRB vs ZN** | -1.06 (-2.14; 0.02) | **--** | 1 | 62 | Very Low^1,1,4^ | -0.39  (-2.04; 1.24) | Very Low^4^ | 7/1 | -0.39  (-2.04; 1.24) | Very Low^4^ |
| **7** | **STND vs ZN** | 0.73 (-0.76; 2.23) | 95% | 2 | 182 | Very Low^1,2,4^ | 0.64  (1.66; 2.95) | Low | 4/2 | 0.51  (-0.66; 1.64) | Very Low^4^ |
| **8** | **STND vs LGG** | 0.7 (-0.53; 1.96) | 46% | 4 | 1280 | Low^1,4^ | 0.70  (-0.47; 1.90) | Low^1,4^ | __ | 0.70  (-0.47; 1.90) | Low^1,4^ |
| **9** | **STND vs SB** | 0.77 (-0.46; 2.02) | 82% | 3 | 257 | Very Low^1,2,4^ | 0.77  (-0.41; 1.95) | Very Low^1,2,4^ | __ | 0.77  (-0.41; 1.95) | Very Low^1,2,4^ |
| **10** | **STND vs All-PRB** | 0.85 (0; 1.72) | 80% | 8 | 1003 | Very Low^1,2,4^ | 4.27  (0.19; 8.36) | Low | 11/14 | **0.96**  **(1.51; 1.76)** | **Very Low^4^** |
| **11** | **YOG vs All-PRB** | 0.74 (-0.63; 2.15) | **--** | 1 | 120 | Low^1,4^ | 0.38  (-1.00; 1.75) | Very Low^6^ | 10/14 | -0.08  (-1.48; 1.26) | Low^4^ |
| **12** | **STND vs SYM** | 1.14 (-0.48; 2.69) | 0% | 2 | 320 | Moderate^1^ | 1.13  (-0.33; 2.62) | Moderate^1^ | __ | 1.13  (-0.33; 2.62) | Moderate^1^ |
| **13** | **STND vs LCF** | 1.18 (-0.24; 2.61) | 58% | 4 | 329 | Very Low^1,2,4^ | 1.18  (-0.17; 2.55) | Very Low^1,2,4^ | __ | 1.18  (-0.17; 2.55) | Very Low^1,2,4^ |
| **14** | **STND vs YOG** | 1.06 (-0.2; 2.38) | 93% | 4 | 522 | Very Low^1,2,4^ | -1.04  (-4.71; 2.63) | Moderate | 11/10 | 1.04  (-0.11; 2.27) | Low^4^ |
| **15** | **SM vs RC** | NA | NA | NA | NA | NA | -0.4 (-3.52; 2.77) | Very low^4^ | 2/5 | -0.4 (-3.52; 2.77) | Very low^4^ |
| **16** | **ZN vs SM** | NA | NA | NA | NA | NA | 0.51 (-2.65; 3.57) | Very Low^4,6^ | 7/5 | 0.51 (-2.65; 3.57) | Very Low^4,6^ |
| **17** | **LGG vs RC** | NA | NA | NA | NA | NA | -0.1 (-1.87; 1.61) | Very Low^4,6^ | 2/8 | -0.1 (-1.87; 1.61) | Very Low^4,6^ |
| **18** | **LGG vs SM** | NA | NA | NA | NA | NA | 0.28 (-2.85; 3.4) | Very Low^4,6^ | 5/8 | 0.28 (-2.85; 3.4) | Very Low^4,6^ |
| **19** | **LGG vs ZN** | NA | NA | NA | NA | NA | -0.19 (-1.88; 1.46) | Very low^4^ | 7/8 | -0.19 (-1.88; 1.46) | Very low^4^ |
| **20** | **SB vs RC** | NA | NA | NA | NA | NA | -0.17 (-1.95; 1.56) | Very low^4^ | 2/9 | -0.17 (-1.95; 1.56) | Very low^4^ |
| **21** | **SB vs SM** | NA | NA | NA | NA | NA | 0.21 (-2.9; 3.31) | Very Low^4,6^ | 5/9 | 0.21 (-2.9; 3.31) | Very Low^4,6^ |
| **22** | **SB vs ZN** | NA | NA | NA | NA | NA | -0.26 (-1.96; 1.36) | Very low^4^ | 7/9 | -0.26 (-1.96; 1.36) | Very low^4^ |
| **23** | **SB vs LGG** | NA | NA | NA | NA | NA | -0.07 (-1.7; 1.63) | Very low^4^ | 8/9 | -0.07 (-1.7; 1.63) | Very low^4^ |
| **24** | **All-PRB vs RC** | NA | NA | NA | NA | NA | -0.37 (-1.87; 1.15) | Very low^4^ | 2/11 | -0.37 (-1.87; 1.15) | Very low^4^ |
| **25** | **All-PRB vs SM** | NA | NA | NA | NA | NA | 0.03 (-2.99; 3.03) | Very Low^4,6^ | 5/11 | 0.03 (-2.99; 3.03) | Very Low^4,6^ |
| **26** | **All-PRB vs ZN** | NA | NA | NA | NA | NA | -0.46 (-1.88; 0.95) | Very low^4^ | 7/11 | -0.46 (-1.88; 0.95) | Very low^4^ |
| **27** | **All-PRB vs LGG** | NA | NA | NA | NA | NA | -0.26 (-1.71; 1.18) | Low^4^ | 8/11 | -0.26 (-1.71; 1.18) | Low^4^ |
| **28** | **All-PRB vs SB** | NA | NA | NA | NA | NA | -0.19 (-1.63; 1.27) | Very low^4^ | 9/11 | -0.19 (-1.63; 1.27) | Very low^4^ |
| **29** | **SYM vs RC** | NA | NA | NA | NA | NA | -0.55 (-2.53; 1.37) | Very low^4^ | 2/12 | -0.55 (-2.53; 1.37) | Very low^4^ |
| **30** | **SYM vs SM** | NA | NA | NA | NA | NA | -0.14 (-3.36; 3.07) | Very low^4^ | 5/12 | -0.14 (-3.36; 3.07) | Very low^4^ |
| **31** | **SYM vs ZN** | NA | NA | NA | NA | NA | -0.63 (-2.51; 1.22) | Very low^4^ | 7/12 | -0.63 (-2.51; 1.22) | Very low^4^ |
| **32** | **SYM vs LGG** | NA | NA | NA | NA | NA | -0.44 (-2.28; 1.44) | Very Low^4,6^ | 8/12 | -0.44 (-2.28; 1.44) | Very Low^4,6^ |
| **33** | **SYM vs SB** | NA | NA | NA | NA | NA | -0.36 (-2.28; 1.5) | Very low^4^ | 9/12 | -0.36 (-2.28; 1.5) | Very low^4^ |
| **34** | **SYM vs All-PRB** | NA | NA | NA | NA | NA | -0.18 (-1.87; 1.51) | Moderate^4^ | 11/12 | -0.18 (-1.87; 1.51) | Moderate^4^ |
| **35** | **LCF vs RC** | NA | NA | NA | NA | NA | -0.59 (-2.46; 1.33) | Very Low^4,6^ | 2/13 | -0.59 (-2.46; 1.33) | Very Low^4,6^ |
| **36** | **LCF vs SM** | NA | NA | NA | NA | NA | -0.17 (-3.39; 2.99) | Very Low^4,6^ | 5/13 | -0.17 (-3.39; 2.99) | Very Low^4,6^ |
| **37** | **LCF vs ZN** | NA | NA | NA | NA | NA | -0.68 (-2.46; 1.12) | Very low^4^ | 7/13 | -0.68 (-2.46; 1.12) | Very low^4^ |
| **38** | **LCF vs LGG** | NA | NA | NA | NA | NA | -0.47 (-2.29; 1.35) | Very low^4^ | 8/13 | -0.47 (-2.29; 1.35) | Very low^4^ |
| **39** | **LCF vs SB** | NA | NA | NA | NA | NA | -0.41 (-2.18; 1.38) | Very low^4^ | 9/13 | -0.41 (-2.18; 1.38) | Very low^4^ |
| **40** | **LCF vs All-PRB** | NA | NA | NA | NA | NA | -0.22 (-1.82; 1.36) | Very low^4^ | 11/13 | -0.22 (-1.82; 1.36) | Very low^4^ |
| **41** | **LCF vs SYM** | NA | NA | NA | NA | NA | -0.05 (-2.06; 1.95) | Very Low^4,6^ | 12/13 | -0.05 (-2.06; 1.95) | Very Low^4,6^ |
| **42** | **YOG vs RC** | NA | NA | NA | NA | NA | -0.43 (-2.25; 1.23) | Very low^4^ | 2/14 | -0.43 (-2.25; 1.23) | Very low^4^ |
| **43** | **YOG vs SM** | NA | NA | NA | NA | NA | -0.05 (-3.19; 3.02) | Very low^4^ | 5/14 | -0.05 (-3.19; 3.02) | Very low^4^ |
| **44** | **YOG vs ZN** | NA | NA | NA | NA | NA | -0.53 (-2.24; 1.1) | Very low^4^ | 7/14 | -0.53 (-2.24; 1.1) | Very low^4^ |
| **45** | **YOG vs LGG** | NA | NA | NA | NA | NA | -0.34 (-2.06; 1.31) | Very low^4^ | 8/14 | -0.34 (-2.06; 1.31) | Very low^4^ |
| **46** | **YOG vs SB** | NA | NA | NA | NA | NA | -0.27 (-1.97; 1.36) | Very low^4^ | 9/14 | -0.27 (-1.97; 1.36) | Very low^4^ |
| **47** | **YOG vs SYM** | NA | NA | NA | NA | NA | 0.1 (-1.82; 1.95) | Very Low^4,6^ | 12/14 | 0.1 (-1.82; 1.95) | Very Low^4,6^ |
| **48** | **YOG vs LCF** | NA | NA | NA | NA | NA | 0.14 (-1.7; 1.92) | Very low^4^ | 13/14 | 0.14 (-1.7; 1.92) | Very low^4^ |
| **49** | **ZN+PRB vs RC** | NA | NA | NA | NA | NA | -0.29 (-2.28; 1.65) | Very low^4^ | 2/6 | -0.29 (-2.28; 1.65) | Very low^4^ |
| **50** | **ZN+PRB vs SM** | NA | NA | NA | NA | NA | 0.1 (-3.21; 3.4) | Very Low^4,6^ | 5/6 | 0.1 (-3.21; 3.4) | Very Low^4,6^ |
| **51** | **ZN+PRB vs LGG** | NA | NA | NA | NA | NA | -0.19 (-2.16; 1.81) | Very low^4^ | 8/6 | -0.19 (-2.16; 1.81) | Very low^4^ |
| **52** | **ZN+PRB vs SB** | NA | NA | NA | NA | NA | -0.12 (-2.11; 1.87) | Very low^4^ | 9/6 | -0.12 (-2.11; 1.87) | Very low^4^ |
| **53** | **ZN+PRB vs All-PRB** | NA | NA | NA | NA | NA | 0.07 (-1.73; 1.85) | Very low^4^ | 11/6 | 0.07 (-1.73; 1.85) | Very low^4^ |
| **54** | **ZN+PRB vs SYM** | NA | NA | NA | NA | NA | 0.25 (-1.94; 2.45) | Very Low^4,6^ | 12/6 | 0.25 (-1.94; 2.45) | Very Low^4,6^ |
| **55** | **ZN+PRB vs LCF** | NA | NA | NA | NA | NA | 0.29 (-1.84; 2.36) | Very Low^4,6^ | 13/6 | 0.29 (-1.84; 2.36) | Very Low^4,6^ |
| **56** | **ZN+PRB vs YOG** | NA | NA | NA | NA | NA | 0.14 (-1.81; 2.17) | Very low^4^ | 14/6 | 0.14 (-1.81; 2.17) | Very low^4^ |
| **57** | **CAO vs SM** | NA | NA | NA | NA | NA | 3.68 (-0.06; 7.44) | Very low^4^ | 5/3/2 | 3.68 (-0.06; 7.44) | Very low^4^ |
| **58** | **CAO vs ZN** | NA | NA | NA | NA | NA | **3.21 (0.73; 5.75)** | **Very Low^6^** | 7/3/2 | **3.21 (0.73; 5.75)** | **Very Low^6^** |
| **59** | **CAO vs LGG** | NA | NA | NA | NA | NA | **3.4 (0.79; 6.14)** | **Low** | 8/3/2 | **3.4 (0.79; 6.14)** | **Low** |
| **60** | **CAO vs SB** | NA | NA | NA | NA | NA | **3.46 (0.82; 6.2)** | **Low** | 9/3/2 | **3.46 (0.82; 6.2)** | **Low** |
| **61** | **CAO vs All-PRB** | NA | NA | NA | NA | NA | **3.66 (1.15; 6.23)** | **Low** | 11/3/2 | **3.66 (1.15; 6.23)** | **Low** |
| **62** | **CAO vs SYM** | NA | NA | NA | NA | NA | **3.83 (1.04; 6.65)** | **Low** | 12/3/2 | **3.83 (1.04; 6.65)** | **Low** |
| **63** | **CAO vs LCF** | NA | NA | NA | NA | NA | **3.88 (1.16; 6.7)** | **Very Low^6^** | 13/3/2 | **3.88 (1.16; 6.7)** | **Very Low^6^** |
| **64** | **CAO vs YOG** | NA | NA | NA | NA | NA | **3.73 (1.14; 6.5)** | **Very Low^6^** | 14/3/2 | **3.73 (1.14; 6.5)** | **Very Low^6^** |
| **65** | **CAO vs ZN+PRB** | NA | NA | NA | NA | NA | **3.58 (0.75; 6.45)** | **Low** | 6/3/2 | **3.58 (0.75; 6.45)** | **Low** |
| **66** | **CAO vs STND** | NA | NA | NA | NA | NA | **2.7 (0.32; 5.16)** | **Very Low^6^** | 2/3/2 | **2.7 (0.32; 5.16)** | **Very Low^6^** |

**+** This column displays the direct comparisons GRADE Assessments that informed the indirect estimates GRADE assessment. Numbers are showing the number ID of the direct comparison (which are all between 1-14)

**MD:** Mean difference; **95CrI%:** 95% Credible Intervals; **I^2^**: I-square statistic for heterogeneity in direct comparisons; **NMA:** Network Meta-analysis; **NA**: Non-applicable; In **Bold**, estimates that were statistically significant. **RC:** racecadotril; **SM:** Smectite; **ZN**: Zinc; **LGG:** *Lactobacillus rhamnosus* –GG; **SB:** *Sacharomyces boulardii*; **ALL-PRB**: All probiotics (except LGG and SB); **SYM:** Symbiotics, **LCF:** lactose-free formula; **YOG:** Yogurt; **ZN+PRB:** Zinc + Probiotics; **STND:** Standard treatment or Placebo**; CAO:** Kaolin-Pectin.

**GRADE Assessment:** Reasons for downgrading direct evidence(1 to 5), indirect (6) and Mixed estimates(4, 7): 1. Downgraded because of Risk of Bias; 2. Downgraded because of Inconsistency; 3. Downgraded because of Indirectness; 4. Downgraded because of Imprecision; 5. Downgraded because of Publication Bias; 6. Downgraded because of Intransitivity; 7. Downgraded because of Incoherence; NOTE: When a superscript is more than one for an estimate, means that the criterion was downgraded 2 points (-2), instead on one point (-1)

# Table K: Assessment of Incoherence for Indirect Comparisons (Stool frequency day 2)

| **Comparison** | **Z-value** | **P- value** |
| --- | --- | --- |
| STND vs RC | 0.06 | 0.948 |
| ZN vs RC | -0.06 | 0.948 |
| STND vs ZN | -0.06 | 0.948 |
| STND vs All-PRB | 1.62 | 0.106 |
| YOG vs All-PRB | -0.89 | 0.375 |
| STND vs YOG | 0.03 | 0.976 |
| Whole Network |  | 0.99 |

Incoherence between the direct and indirect estimates in the network was evaluated using both the global test random-effects design-by-treatment interaction model (Veroniki 2013) for the whole network and with the node-splitting method (Dias 2010) for local assessment.

# Fig F: NMA Forest plot of all interventions vs. STND for diarrhea at day 3

**– # 10,339 patients, # 12 treatments, # 46 studies –**

**OR:** Odds Ratio; **95CrI%:** 95% Credible Intervals; **SM:** Smectite, **LOP:** Loperamide; **ZN**: Zinc; **LGG:** *Lactobacillus rhamnosus* –GG; **SB:** *Sacharomyces boulardii*; **ALL-PRB**: All probiotics (except LGG and SB); **SYM:** Symbiotics, **LCF:** lactose-free formula; **YOG:** Yogurt; **ZN+MN:** Zinc + Micronutrients; **ZN+PRB:** Zinc + Probiotics; **STND:** Standard treatment or Placebo**;**

# Table L: Direct, Indirect and NMA Estimates for Diarrhea at Day 3 with the GRADE Assessment

| **#** | **Comparison** | **Direct Estimates** OR(95%CrI) | -I^2^ | **Number of studies** | **Number of patients** | **Direct GRADE** | **Indirect**  **Estimates**  logOR(95%CrI) | **Indirect GRADE** | **+ Direct GRADE that informs Indirect** | **NMA estimates**  OR(95%CrI) | **NMA GRADE** |
| --- | --- | --- | --- | --- | --- | --- | --- | --- | --- | --- | --- |
| **1** | **ZN+MN vs STND** | **0.75 (0.57; 0.99)** | **--** | **1** | **896** | High | 0.64  (-1.87; 3.15) | Low | 6/7 | 0.69 (0.17; 2.63) | Moderate^4^ |
| **2** | **STND vs SM** | **6.4 (2.18; 19.42)** | **92%** | **3** | **218** | Very Low^1,2,4^ | **6.31 (2.05; 20.13)** | **Very Low^1,2,4^** | __ | **6.31 (2.05; 20.13)** | **Very Low^1,2,4^** |
| **3** | **STND vs LOP** | 0.95 (0.24; 3.86) | -- | 1 | 65 | Very Low^1,2,4^ | -1.38  (-4.83; 2.06) | Very Low^6^ | 12/4 | 1.34 (0.22; 8.48) | Low^4^ |
| **4** | **All-PRB vs LOP** | 0.62 (0.14; 2.7) | -- | 1 | 70 | Very Low^1,4,4^ | 1.81  (-1.41; 5.03) | Very Low^6^ | 12/3 | 0.43 (0.07; 2.77) | Low^4^ |
| **5** | **ZN+PRB vs ZN** | 1.04 (0.37; 2.91) | -- | 1 | 64 | Very Low^1,2,4^ | 1.04 (0.16; 6.4) | Very Low^1,2,4^ | __ | 1.04 (0.16; 6.4) | Very Low^1,2,4^ |
| **6** | **STND vs ZN** | **1.75 (1.05; 2.95)** | **73%** | **10** | **5,401** | Low^1,2^ | -2.29  (-5.17; 0.59) | Moderate | 8/13 | **1.83 (1.1; 3.1)** | **Moderate** |
| **7** | **ZN+MN vs ZN** | 1.15 (0.86; 1.55) | -- | 1 | 889 | High | -0.50  (-3.00; 1.99) | Low | 6/1 | 1.26 (0.32; 4.8) | Moderate^4^ |
| **8** | **SYM vs ZN** | 1.95 (0.92; 4.26) | -- | 1 | 110 | Very Low^1,2,4^ | 1.08  (0.09; 2.07) | Low | 6/13 | 0.58 (0.23; 1.48) | Low^4^ |
| **9** | **STND vs LGG** | 2.81 (0.82; 9.99) | 61% | 2 | 306 | Very Low^1,2,4^ | 1.21  (-0.96; 3.39) | Low | 10/12 | 1.61 (0.53; 4.92) | Very low^4^ |
| **10** | **All-PRB vs LGG** | **0.09 (0.01; 0.44)** | **--** | **1** | **40** | **Moderate^4^** | 0.07  (-1.17; 1.31) | Very Low^6^ | 9/12 | 0.51 (0.16; 1.66) | Moderate^4^ |
| **11** | **STND vs SB** | **5.55 (3.09; 10.11)** | **78%** | **8** | **1,549** | **Low^1,2^** | **5.55 (2.99; 10.24)** | **Low^1,2^** | __ | **5.55 (2.99; 10.24)** | **Low^1,2^** |
| **12** | **STND vs All-PRB** | **2.87 (1.78; 4.55)** | **53%** | **14** | **1,384** | **Low^1,3^** | **-3.18**  **(-5.61; -0.76)** | **Moderate** | 4/3 | **3.1 (1.92; 5.02)** | **Low^4^** |
| **13** | **STND vs SYM*** | **3.97 (1.68; 9.37)** | **24%** | **4** | **530** | **Moderate^1^** | 1.47  (-1.12; 4.05) | Very Low^6^ | 6/8 | **3.17 (1.39; 7.32)** | **Low^4,7^** |
| **14** | **STND vs LCF** | 0.9 (0.4; 2.07) | -- | 1 | 115 | Very Low^1,4,4^ | 0.91 (0.16; 5.15) | Very Low^1,4,4^ | __ | 0.91 (0.16; 5.15) | Very Low^1,4,4^ |
| **15** | **STND vs YOG** | 1.5 (0.56; 4.42) | 89% | 3 | 310 | Very Low^1,2,4^ | 1.55 (0.53; 4.73) | Very Low^1,2,4^ | __ | 1.55 (0.53; 4.73) | Very Low^1,2,4^ |
| **16** | **LOP vs SM** | NA | NA | NA | NA | NA | 4.68  (0.55; 40.27) | Very Low^6^ | 3/2 | 4.68  (0.55; 40.27) | Very Low^6^ |
| **17** | **ZN vs SM** | NA | NA | NA | NA | NA | 3.46  (1.0; 12.11) | Very Low^4^ | 6/2 | 3.46  (1; 12.11) | Very Low^4^ |
| **18** | **ZN vs LOP** | NA | NA | NA | NA | NA | 0.73  (0.11; 4.95) | Very Low^6^ | 6/3 | 0.73  (0.11; 4.95) | Very Low^6^ |
| **19** | **LGG vs SM** | NA | NA | NA | NA | NA | 3.91  (0.81; 19.1) | Very Low^4^ | 2/9 | 3.91  (0.81; 19.1) | Very Low^4^ |
| **20** | **LGG vs LOP** | NA | NA | NA | NA | NA | 0.84  (0.1; 7.02) | Very Low^4^ | 3/9 | 0.84  (0.1; 7.02) | Very Low^4^ |
| **21** | **LGG vs ZN** | NA | NA | NA | NA | NA | 1.13  (0.34; 3.97) | Very Low^4^ | 6/9 | 1.13  (0.34; 3.97) | Very Low^4^ |
| **22** | **SB vs SM** | NA | NA | NA | NA | NA | 1.14  (0.31; 4.14) | Very Low^4^ | 2/11 | 1.14  (0.31; 4.14) | Very Low^4^ |
| **23** | **SB vs LOP** | NA | NA | NA | NA | NA | 0.24  (0.04; 1.69) | Very Low^6^ | 3/11 | 0.24  (0.04; 1.69) | Very Low^6^ |
| **24** | **SB vs ZN** | NA | NA | NA | NA | NA | **0.33  (0.15; 0.74)** | **Low** | 6/11 | **0.33  (0.15; 0.74)** | **Low** |
| **25** | **SB vs LGG** | NA | NA | NA | NA | NA | 0.29  (0.08; 1.03) | Very Low^4^ | 9/11 | 0.29  (0.08; 1.03) | Very Low^4^ |
| **26** | **All-PRB vs SM** | NA | NA | NA | NA | NA | 2.03  (0.6; 7.08) | Very Low^4^ | 2/12 | 2.03  (0.6; 7.08) | Very Low^4^ |
| **27** | **All-PRB vs ZN** | NA | NA | NA | NA | NA | 0.59  (0.29; 1.2) | Very Low^4^ | 6/12 | 0.59  (0.29; 1.2) | Very Low^4^ |
| **28** | **All-PRB vs SB** | NA | NA | NA | NA | NA | 1.78  (0.83; 3.94) | Very Low^4^ | 11/12 | 1.78  (0.83; 3.94) | Very Low^4^ |
| **29** | **SYM vs SM** | NA | NA | NA | NA | NA | 1.99  (0.49; 8.17) | Low^4^ | 2/13 | 1.99  (0.49; 8.17) | Low^4^ |
| **30** | **SYM vs LOP** | NA | NA | NA | NA | NA | 0.42  (0.06; 3.13) | Very Low^4,6^ | 3/13 | 0.42  (0.06; 3.13) | Very Low^4,6^ |
| **31** | **SYM vs LGG** | NA | NA | NA | NA | NA | 0.51  (0.13; 2) | Low^4^ | 9/13 | 0.51  (0.13; 2) | Low^4^ |
| **32** | **SYM vs SB** | NA | NA | NA | NA | NA | 1.75  (0.61; 4.93) | Low^4^ | 11/13 | 1.75  (0.61; 4.93) | Low^4^ |
| **33** | **SYM vs All-PRB** | NA | NA | NA | NA | NA | 0.98  (0.37; 2.57) | Low^4^ | 12/13 | 0.98  (0.37; 2.57) | Low^4^ |
| **34** | **LCF vs SM** | NA | NA | NA | NA | NA | 6.94  (0.86; 56.97) | Low^4^ | 2/14 | 6.94  (0.86; 56.97) | Low^4^ |
| **35** | **LCF vs LOP** | NA | NA | NA | NA | NA | 1.47  (0.12; 19.44) | Low^4^ | 3/14 | 1.47  (0.12; 19.44) | Low^4^ |
| **36** | **LCF vs ZN** | NA | NA | NA | NA | NA | 2.03  (0.33; 12.64) | Very Low^6^ | 6/14 | 2.03  (0.33; 12.64) | Very Low^6^ |
| **37** | **LCF vs LGG** | NA | NA | NA | NA | NA | 1.76  (0.22; 14.23) | Very Low^6^ | 9/14 | 1.76  (0.22; 14.23) | Very Low^6^ |
| **38** | **LCF vs SB** | NA | NA | NA | NA | NA | 6.12  (0.97; 38.27) | Very Low^6^ | 11/14 | 6.12  (0.97; 38.27) | Very Low^6^ |
| **39** | **LCF vs All-PRB** | NA | NA | NA | NA | NA | 3.41  (0.57; 21.05) | Very Low^6^ | 12/14 | 3.41  (0.57; 21.05) | Very Low^6^ |
| **40** | **LCF vs SYM** | NA | NA | NA | NA | NA | 3.49  (0.51; 24.26) | Very Low^4,6^ | 13/14 | 3.49  (0.51; 24.26) | Very Low^4,6^ |
| **41** | **YOG vs SM** | NA | NA | NA | NA | NA | 4.06  (0.85; 19.4) | Very Low^4^ | 2/15 | 4.06  (0.85; 19.4) | Very Low^4^ |
| **42** | **YOG vs LOP** | NA | NA | NA | NA | NA | 0.86  (0.1; 7.13) | Very Low^6^ | 3/15 | 0.86  (0.1; 7.13) | Very Low^6^ |
| **43** | **YOG vs ZN** | NA | NA | NA | NA | NA | 1.18  (0.34; 3.87) | Very Low^4^ | 6/15 | 1.18  (0.34; 3.87) | Very Low^4^ |
| **44** | **YOG vs LGG** | NA | NA | NA | NA | NA | 1.03  (0.21; 4.81) | Very Low^4^ | 9/15 | 1.03  (0.21; 4.81) | Very Low^4^ |
| **45** | **YOG vs SB** | NA | NA | NA | NA | NA | **3.59  (1.01; 11.9)** | **Low** | 11/15 | **3.59  (1.01; 11.9)** | **Low** |
| **46** | **YOG vs All-PRB** | NA | NA | NA | NA | NA | 2.00  (0.59; 6.55) | Very Low^4^ | 12/15 | 2.00  (0.59; 6.55) | Very Low^4^ |
| **47** | **YOG vs SYM** | NA | NA | NA | NA | NA | 2.05  (0.51; 7.82) | Very Low^4^ | 13/15 | 2.05  (0.51; 7.82) | Very Low^4^ |
| **48** | **YOG vs LCF** | NA | NA | NA | NA | NA | 0.59  (0.07; 4.48) | Very Low^4^ | 14/15 | 0.59  (0.07; 4.48) | Very Low^4^ |
| **49** | **ZN+PRB vs SM** | NA | NA | NA | NA | NA | 3.52  (0.38; 33.04) | Very Low^6^ | 2/6/5 | 3.52  (0.38; 33.04) | Very Low^6^ |
| **50** | **ZN+PRB vs LOP** | NA | NA | NA | NA | NA | 0.75  (0.05; 10.65) | Very Low^6^ | 3/6/5 | 0.75  (0.05; 10.65) | Very Low^6^ |
| **51** | **ZN+PRB vs LGG** | NA | NA | NA | NA | NA | 0.92  (0.1; 8.05) | Very Low^6^ | 9/6/5 | 0.92  (0.1; 8.05) | Very Low^6^ |
| **52** | **ZN+PRB vs SB** | NA | NA | NA | NA | NA | 3.12  (0.42; 22.71) | Very Low^4^ | 11/6/5 | 3.12  (0.42; 22.71) | Very Low^4^ |
| **53** | **ZN+PRB vs All-PRB** | NA | NA | NA | NA | NA | 1.73  (0.25; 12.32) | Very Low^4^ | 12/6/5 | 1.73  (0.25; 12.32) | Very Low^4^ |
| **54** | **ZN+PRB vs SYM** | NA | NA | NA | NA | NA | 1.79  (0.23; 13.86) | Very Low^4^ | 13/6/5 | 1.79  (0.23; 13.86) | Very Low^4^ |
| **55** | **ZN+PRB vs LCF** | NA | NA | NA | NA | NA | 0.51  (0.04; 6.94) | Very Low^6^ | 14/6/5 | 0.51  (0.04; 6.94) | Very Low^6^ |
| **56** | **ZN+PRB vs YOG** | NA | NA | NA | NA | NA | 0.88  (0.1; 7.83) | Very Low^6^ | 15/6/5 | 0.88  (0.1; 7.83) | Very Low^6^ |
| **57** | **STND vs ZN+PRB** | NA | NA | NA | NA | NA | 1.77  (0.27; 12.08) | Very Low^6^ | 5/6 | 1.77  (0.27; 12.08) | Very Low^6^ |
| **58** | **ZN+MN vs SM** | NA | NA | NA | NA | NA | 4.35  (0.74; 25.49) | Very Low^4^ | 2/7 | 4.35  (0.74; 25.49) | Very Low^4^ |
| **59** | **ZN+MN vs LOP** | NA | NA | NA | NA | NA | 0.93  (0.09; 8.79) | Low^4^ | 3/7 | 0.93  (0.09; 8.79) | Low^4^ |
| **60** | **ZN+MN vs LGG** | NA | NA | NA | NA | NA | 1.11  (0.18; 6.39) | Very Low^4^ | 9/7 | 1.11  (0.18; 6.39) | Very Low^4^ |
| **61** | **ZN+MN vs SB** | NA | NA | NA | NA | NA | 3.83  (0.85; 16.51) | Very Low^6^ | 11/7 | 3.83  (0.85; 16.51) | Very Low^6^ |
| **62** | **ZN+MN vs All-PRB** | NA | NA | NA | NA | NA | 2.14  (0.49; 8.8) | Very Low^4^ | 12/7 | 2.14  (0.49; 8.8) | Very Low^4^ |
| **63** | **ZN+MN vs SYM** | NA | NA | NA | NA | NA | 2.2  (0.44; 10.42) | Low^4^ | 13/7 | 2.2  (0.44; 10.42) | Low^4^ |
| **64** | **ZN+MN vs LCF** | NA | NA | NA | NA | NA | 0.62  (0.07; 5.78) | Very Low^4,6^ | 14/7 | 0.62  (0.07; 5.78) | Very Low^4,6^ |
| **65** | **ZN+MN vs YOG** | NA | NA | NA | NA | NA | 1.06  (0.19; 6.23) | Very Low^6^ | 15/7 | 1.06  (0.19; 6.23) | Very Low^6^ |
| **66** | **ZN+MN vs ZN+PRB** | NA | NA | NA | NA | NA | 1.21  (0.13; 12.2) | Very Low^4,6^ | 7/5 | 1.21  (0.13; 12.2) | Very Low^4,6^ |

**+** This column displays the direct comparisons GRADE Assessments that informed the indirect estimates GRADE assessment. Numbers are showing the number ID of the direct comparison (which are all between 1-15)

** Comparison with Incoherence between Direct and indirect evidence (p=0.039). Direct GRADE assessment displayed includes the Precision assessment;*

**OR:** Odds Ratio; **95CrI%:** 95% Credible Intervals; **I^2^**: I-square statistic for heterogeneity in direct comparisons; **NMA:** Network Meta-analysis; **NA:** Non-applicable; In **Bold** are estimates that were statistically significant. **SM:** Smectite, **LOP:** Loperamide; **ZN**: Zinc; **LGG:** *L. rhamnosus* –GG; **SB:** *Sacharomyces boulardii*; **ALL-PRB**: All probiotics (except LGG and SB); **SYM:** Symbiotics, **LCF:** lactose-free formula; **YOG:** Yogurt; **ZN+MN:** Zinc + Micronutrients; **ZN+PRB:** Zinc + Probiotics; **STND:** Standard treatment or Placebo**; GRADE Assessment:** Reasons for downgrading direct evidence(1 to 5), indirect (6) and Mixed estimates(4, 7): 1. Downgraded because of Risk of Bias; 2. Downgraded because of Inconsistency; 3. Downgraded because of Indirectness; 4. Downgraded because of Imprecision; 5. Downgraded because of Publication Bias; 6. Downgraded because of Intransitivity; 7. Downgraded because of Incoherence; NOTE: When a superscript is more than one for an estimate, means that the criterion was downgraded 2 points (-2), instead on one point (-1)

# Table M: Assessment of incoherence for indirect comparisons (Diarrhea day 3)

| **Comparison** | **Z-value** | **P- value** |
| --- | --- | --- |
| ZN+MN vs STND | -0.25 | 0.802 |
| STND vs LOP | 0.71 | 0.478 |
| All-PRB vs LOP | -0.71 | 0.478 |
| STND vs ZN | 1.18 | 0.237 |
| ZN+MN vs ZN | 0.25 | 0.802 |
| SYM vs ZN | -1.91 | 0.056 |
| STND vs LGG | -1.72 | 0.085 |
| All-PRB vs LGG | 1.72 | 0.085 |
| STND vs All-PRB | 1.72 | 0.085 |
| **STND vs SYM** | **-2.05** | **0.0408** |
| Whole Network |  | 0.23 |

Incoherence between the direct and indirect estimates in the network was evaluated using both the global test random-effects design-by-treatment interaction model (Veroniki 2013) for the whole network and with the node-splitting method (Dias 2010) for local assessment.

# Fig G: NMA Forest plot of all interventions vs. STND for Vomiting

**– # 5671 patients, # 16 treatments, # 23 studies–**

**
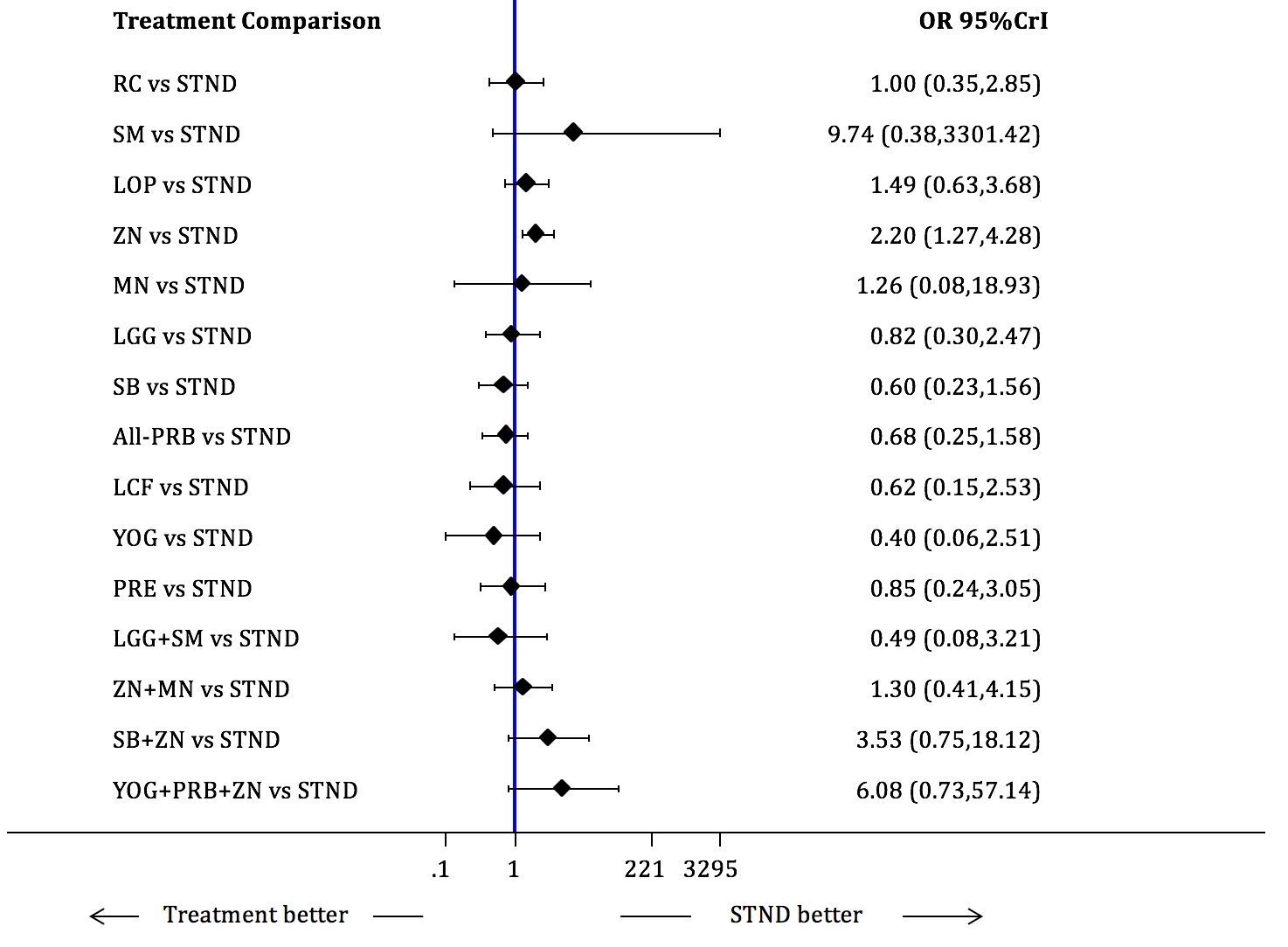
**

**OR:** Odds Ratio; **95CrI%:** 95% Credible Intervals; **NMA:** Network Meta-analysis; **NA:** Non-applicable; In **Bold** are estimates that were statistically significant. **RC:** Racecadotril; **SM:** Smectite, **LOP:** Loperamide; **ZN**: Zinc; **MN:** Micronutrients **LGG:** *Lactobacillus rhamnosus* –GG; **SB:** *Sacharomyces boulardii*; **ALL-PRB**: All probiotics (except LGG and SB); **LCF:** lactose-free formula; **PRE:** prebiotics; **LGG+SM**: LGG+Smectite; **STND:** Standard treatment or Placebo**; ZN+MN:** Zinc + micronutrients; **SB+ZN:** *S. boulardii* + Zinc; **YOG+PRB+ZN:** yogurt + probiotics +zinc.

# Table N: Direct, Indirect, and NMA Estimates for Vomiting with the GRADE Assessment

| **#** | **Comparison** | **Direct Estimates** OR (95%CrI) | **I^2^** | **## studies** | **# Patients** | **Direct GRADE** | **Indirect**  **Estimates**  OR (95%CrI) | **Indirect GRADE** | **+ Direct GRADE that informs Indirect** | **NMA estimates**  OR (95%CrI) | **NMA GRADE** |
| --- | --- | --- | --- | --- | --- | --- | --- | --- | --- | --- | --- |
| 1 | **STND vs PRE** | 1.18  (0.59; 2.35) | -- | 1 | 144 | Moderate^4^ | 1.18  (0.33; 4.12) | Moderate | ---- | 1.18  (0.33; 4.12) | Moderate |
| 2 | **ZN+MN vs STND** | 1.31  (0.80; 2.16) | -- | 1 | 266 | Moderate^4^ | 1.30  (0.41; 4.15) | Moderate | ---- | 1.30  (0.41; 4.15) | Moderate |
| 3 | **YOG+PRB+ZN vs SB+ZN** | 1.72  (0.58; 5.14 | -- | 1 | 55 | Very Low^1,1,4^ | 1.73  (0.39; 7.62 | Very Low | / | 1.73  (0.39; 7.62 | Very Low |
| 4 | **STND vs RC** | 1.03  (0.52; 2.00) | -- | 1 | 135 | Very Low^1,1,4^ | 0.07  (-1.74; 1.89) | Low | 4/6 | 1.00  (0.35; 2.88) | Very Low |
| 5 | **LOP vs RC** | 1.40  (0.33; 6.44) | -- | 1 | 97 | Very Low^1,1,4^ | -0.42  (-1.78; 0.94) | Very Low^6^ | 3/6 | 1.51  (0.47; 4.90) | Very Low |
| 6 | **STND vs SM** | 0.11  (0.00; 2.10) | -- | 1 | 64 | Very Low^1,1,4^ | 0.10  (0.00; 2.64) | Very Low | ---- | 0.10  (0.00; 2.64) | Very Low |
| 7 | **STND vs LOP** | 0.65  (0.24; 1.72) | 0% | 3 | 402 | Low^1,4^ | 0.28  (-1.60; 2.16) | Very Low^6^ | 4/3 | 0.67  (0.27; 1.58) | Low |
| 8 | **STND vs ZN** | **0.45**  **(0.23; 0.80)** | 49% | 6 | 2,624 | **High** | **0.45**  **(0.23; 0.79)** | High | ---- | **0.45**  **(0.23; 0.79)** | High |
| 9 | **SB+ZN vs ZN** | 1.56  (0.53; 4.67) | -- | 1 | 70 | Low^1,4^ | 1.58  (0.36; 6.95) | Low | ---- | 1.58  (0.36; 6.95) | Low |
| 10 | **ZN+MN vs MN** | 1.06  (0.11; 10.26) | -- | 1 | 937 | Low^1,4^ | 1.03  (0.09; 12.31) | Low | ---- | 1.03  (0.09; 12.31) | Low |
| 11 | **LGG+SM vs LGG** | 0.60  (0.21; 1.69) | -- | 1 | 81 | Very Low^1,1,4^ | 0.60  (0.14; 2.61) | Very Low | ---- | 0.60  (0.14; 2.61) | Very Low |
| 12 | **STND vs LGG** | 1.30  (0.72; 2.39) | -- | 1 | 192 | Low^1,4^ | 0.00  (-2.36; 2.36) | Low | 13/15 | 1.22  (0.40; 3.38) | Low |
| 13 | **SB vs LGG** | 0.79  (0.42; 1.48) | -- | 1 | 191 | Low^1,4^ | 0.46  (-1.81; 2.74) | Low^6^ | 14/11 | 0.73  (0.24; 2.02) | Low |
| 14 | **All-PRB vs LGG** | 0.91  (0.29; 2.29) | 56% | 2 | 431 | Very Low^1,2,4^ | 1.67  (-1.61; 4.96) | Very Low^6^ | 16/11 | 0.82  (0.27; 1.92) | Very Low |
| 15 | **STND vs SB** | 1.69  (0.65; 4.48) | 0 | 2 | 224 | Low^1,4^ | 14.10  (-16.99;45.19) | Low | 15/16 | 1.66  (0.64; 4.37) | Low |
| 16 | **YOG vs SB** | 0.67  (0.19; 2.28) | -- | 1 | 42 | Very Low^1,1,4^ | 0.67  (0.13; 3.24) | Very Low | / | 0.67  (0.13; 3.24) | Very Low |
| 17 | **All-PRB vs SB** | 1.20  (0.44; 3.00) | 28% | 2 | 423 | Low^1,4^ | 2.86  (-2.91; 8.63) | Low | 14/16 | 1.12  (0.42; 2.60) | Low |
| 18 | **STND vs All-PRB** | 1.36  (0.56; 3.60) | 57% | 2 | 423 | Very Low^1,2,4^ | -3.79  (-9.56; 1.99) | Low^6^ | 15/14 | 1.48  (0.63; 4.07) | Very Low |
| 19 | **STND vs LCF** | 1.60  (0.64; 4.27) | -- | 1 | 80 | Low^1,4^ | 1.61  (0.39; 6.58) | Low | ---- | 1.61  (0.39; 6.58) | Low |
| **20** | **SM vs RC** | NA | NA | NA | NA | NA | 9.9  (0.33; 3723) | Low | 4/6 | 9.9  (0.33; 3723) | Very Low^4^ |
| **21** | **LOP vs SM** | NA | NA | NA | NA | NA | 0.15  (0; 4.55) | Very Low^6^ | 7/6 | 0.15  (0; 4.55) | Very Low^4,6^ |
| **22** | **ZN vs RC** | NA | NA | NA | NA | NA | 2.24  (0.69; 7.81) | Very Low^6^ | 4/8 | 2.24  (0.69; 7.81) | Very Low^4,6^ |
| **23** | **ZN vs SM** | NA | NA | NA | NA | NA | 0.23  (0; 6.1) | Very Low^6^ | 6/8 | 0.23  (0; 6.1) | Very Low^4,6^ |
| **24** | **ZN vs LOP** | NA | NA | NA | NA | NA | 1.49  (0.52; 4.35) | Moderate | 7/8 | 1.49  (0.52; 4.35) | Low^4^ |
| **25** | **MN vs RC** | NA | NA | NA | NA | NA | 1.26  (0.07; 24.07) | Low | 4/2 | 1.26  (0.07; 24.07) | Very Low^4^ |
| **26** | **MN vs SM** | NA | NA | NA | NA | NA | 0.12  (0; 8.66) | Low | 6/2 | 0.12  (0; 8.66) | Very Low^4^ |
| **27** | **MN vs LOP** | NA | NA | NA | NA | NA | 0.84  (0.05; 15) | Moderate | 7/2 | 0.84  (0.05; 15) | Low^4^ |
| **28** | **MN vs ZN** | NA | NA | NA | NA | NA | 0.57  (0.03; 9.14) | Moderate | 8/2 | 0.57  (0.03; 9.14) | Low^4^ |
| **29** | **LGG vs RC** | NA | NA | NA | NA | NA | 0.83  (0.2; 3.86) | Low | 4/12 | 0.83  (0.2; 3.86) | Very Low^4^ |
| **30** | **LGG vs SM** | NA | NA | NA | NA | NA | 0.08  (0; 2.62) | Very Low^6^ | 6/12 | 0.08  (0; 2.62) | Very Low^4,6^ |
| **31** | **LGG vs LOP** | NA | NA | NA | NA | NA | 0.55  (0.14; 2.27) | Moderate | 7/12 | 0.55  (0.14; 2.27) | Low4 |
| **32** | **LGG vs ZN** | NA | NA | NA | NA | NA | 0.37  (0.11; 1.26) | Moderate | 8/12 | 0.37  (0.11; 1.26) | Low4 |
| **33** | **LGG vs MN** | NA | NA | NA | NA | NA | 0.65  (0.04; 12.67) | Moderate | 2/12 | 0.65  (0.04; 12.67) | Low4 |
| **34** | **SB vs RC** | NA | NA | NA | NA | NA | 0.6  (0.15; 2.5) | Low | 4/15 | 0.6  (0.15; 2.5) | Very Low^4^ |
| **35** | **SB vs SM** | NA | NA | NA | NA | NA | 0.06  (0; 1.76) | Very Low^6^ | 6/15 | 0.06  (0; 1.76) | Very Low^4,6^ |
| **36** | **SB vs LOP** | NA | NA | NA | NA | NA | 0.4  (0.11; 1.44) | Moderate | 7/15 | 0.4  (0.11; 1.44) | Low^4^ |
| **37** | **SB vs ZN** | NA | NA | NA | NA | NA | **0.27  (0.08; 0.81)** | **Low^6^** | 8/15 | **0.27  (0.08; 0.81)** | Very Low^4^ |
| **38** | **SB vs MN** | NA | NA | NA | NA | NA | 0.48  (0.03; 8.43) | Low^6^ | 10/15 | 0.48  (0.03; 8.43) | Very Low^4^ |
| **39** | **All-PRB vs RC** | NA | NA | NA | NA | NA | 0.68  (0.16; 2.56) | Low | 4/18 | 0.68  (0.16; 2.56) | Very Low^4^ |
| **40** | **All-PRB vs SM** | NA | NA | NA | NA | NA | 0.07  (0; 2) | Low | 6/18 | 0.07  (0; 2) | Very Low^4^ |
| **41** | **All-PRB vs LOP** | NA | NA | NA | NA | NA | 0.45  (0.11; 1.49) | Low | 7/18 | 0.45  (0.11; 1.49) | Very Low^4^ |
| **42** | **All-PRB vs ZN** | NA | NA | NA | NA | NA | **0.31  (0.09; 0.82)** | **Low** | 8/18 | **0.31  (0.09; 0.82)** | Very Low^4^ |
| **43** | **All-PRB vs MN** | NA | NA | NA | NA | NA | 0.53  (0.03; 9.4) | Low | 2/18 | 0.53  (0.03; 9.4) | Very Low^4^ |
| **44** | **LCF vs RC** | NA | NA | NA | NA | NA | 0.62  (0.11; 3.64) | Low | 4/19 | 0.62  (0.11; 3.64) | Very Low^4^ |
| **45** | **LCF vs SM** | NA | NA | NA | NA | NA | 0.06  (0; 2.2) | Very Low^6^ | 6/19 | 0.06  (0; 2.2) | Very Low^4,6^ |
| **46** | **LCF vs LOP** | NA | NA | NA | NA | NA | 0.41  (0.08; 2.22) | Low^6^ | 7/19 | 0.41  (0.08; 2.22) | Very Low^4,6^ |
| **47** | **LCF vs ZN** | NA | NA | NA | NA | NA | 0.28  (0.06; 1.26) | Low^6^ | 8/19 | 0.28  (0.06; 1.26) | Very Low^4,6^ |
| **48** | **LCF vs MN** | NA | NA | NA | NA | NA | 0.49  (0.02; 10.58) | Moderate | 2/19 | 0.49  (0.02; 10.58) | Low^4^ |
| **49** | **LCF vs LGG** | NA | NA | NA | NA | NA | 0.75  (0.12; 4.01) | Low^6^ | 12/19 | 0.75  (0.12; 4.01) | Very Low^4,6^ |
| **50** | **LCF vs SB** | NA | NA | NA | NA | NA | 1.02  (0.19; 5.65) | Moderate | 15/19 | 1.02  (0.19; 5.65) | Low^4^ |
| **51** | **LCF vs All-PRB** | NA | NA | NA | NA | NA | 0.92  (0.18; 5.08) | Low | 18/19 | 0.92  (0.18; 5.08) | Very Low^4^ |
| **52** | **YOG vs RC** | NA | NA | NA | NA | NA | 0.4  (0.05; 3.32) | Very Low^6^ | 4/15 | 0.4  (0.05; 3.32) | Very Low^4,6^ |
| **53** | **YOG vs SM** | NA | NA | NA | NA | NA | 0.04  (0; 1.76) | Very Low^6^ | 6/15 | 0.04  (0; 1.76) | Very Low^4,6^ |
| **54** | **YOG vs LOP** | NA | NA | NA | NA | NA | 0.27  (0.03; 2.05) | Very Low^6^ | 7/15 | 0.27  (0.03; 2.05) | Very Low^4,6^ |
| **55** | **YOG vs ZN** | NA | NA | NA | NA | NA | 0.18  (0.02; 1.21) | Very Low^6^ | 8/15 | 0.18  (0.02; 1.21) | Very Low^4,6^ |
| **56** | **YOG vs MN** | NA | NA | NA | NA | NA | 0.32  (0.01; 8.26) | Very Low^6^ | 2/15/10 | 0.32  (0.01; 8.26) | Very Low^4,6^ |
| **57** | **YOG vs LGG** | NA | NA | NA | NA | NA | 0.49  (0.07; 3.16) | Low | 12/15 | 0.49  (0.07; 3.16) | Very Low^4^ |
| **58** | **YOG vs All-PRB** | NA | NA | NA | NA | NA | 0.6  (0.1; 3.81) | Low | 18/15 | 0.6  (0.1; 3.81) | Very Low^4^ |
| **59** | **YOG vs LCF** | NA | NA | NA | NA | NA | 0.65  (0.06; 6.44) | Very Low^6^ | 19/15 | 0.65  (0.06; 6.44) | Very Low^4,6^ |
| **60** | **PRE vs RC** | NA | NA | NA | NA | NA | 0.84  (0.17; 4.56) | Low | 4/1 | 0.84  (0.17; 4.56) | Very Low^4^ |
| **61** | **PRE vs SM** | NA | NA | NA | NA | NA | 0.09  (0; 2.89) | Very Low^6^ | 6/2 | 0.09  (0; 2.89) | Very Low^4,6^ |
| **62** | **PRE vs LOP** | NA | NA | NA | NA | NA | 0.57  (0.12; 2.63) | Low^6^ | 7/1 | 0.57  (0.12; 2.63) | Very Low^4,6^ |
| **63** | **PRE vs ZN** | NA | NA | NA | NA | NA | 0.38  (0.09; 1.49) | High | 8/1 | 0.38  (0.09; 1.49) | Moderate |
| **64** | **PRE vs MN** | NA | NA | NA | NA | NA | 0.67  (0.03; 13.34) | Moderate | 2/1 | 0.67  (0.03; 13.34) | Low^4^ |
| **65** | **PRE vs LGG** | NA | NA | NA | NA | NA | 1.03  (0.2; 5.17) | Low^6^ | 12/1 | 1.03  (0.2; 5.17) | Very Low^4,6^ |
| **66** | **PRE vs SB** | NA | NA | NA | NA | NA | 1.4  (0.3; 7.05) | Low^6^ | 15/1 | 1.4  (0.3; 7.05) | Very Low^4,6^ |
| **67** | **PRE vs All-PRB** | NA | NA | NA | NA | NA | 1.24  (0.29; 6.64) | Low | 18/1 | 1.24  (0.29; 6.64) | Very Low^4^ |
| **68** | **PRE vs LCF** | NA | NA | NA | NA | NA | 1.36  (0.21; 9.06) | Moderate | 19/1 | 1.36  (0.21; 9.06) | Low^4^ |
| **69** | **PRE vs YOG** | NA | NA | NA | NA | NA | 2.1  (0.23; 21.37) | Very Low^6^ | 15/1 | 2.1  (0.23; 21.37) | Very Low^4,6^ |
| **70** | **LGG+SM vs RC** | NA | NA | NA | NA | NA | 0.49  (0.07; 4.15) | Low^6^ | 4/12 | 0.49  (0.07; 4.15) | Very Low^4,6^ |
| **71** | **LGG+SM vs SM** | NA | NA | NA | NA | NA | 0.05  (0; 2.1) | Low^6^ | 6/12 | 0.05  (0; 2.1) | Very Low^4,6^ |
| **72** | **LGG+SM vs LOP** | NA | NA | NA | NA | NA | 0.33  (0.04; 2.62) | Moderate | 7/12 | 0.33  (0.04; 2.62) | Low^4^ |
| **73** | **LGG+SM vs ZN** | NA | NA | NA | NA | NA | 0.22  (0.03; 1.53) | Moderate | 8/12 | 0.22  (0.03; 1.53) | Low^4^ |
| **74** | **LGG+SM vs MN** | NA | NA | NA | NA | NA | 0.39  (0.02; 10.57) | Moderate | 10/12/10 | 0.39  (0.02; 10.57) | Low^4^ |
| **75** | **LGG+SM vs SB** | NA | NA | NA | NA | NA | 0.81  (0.14; 5.45) | Moderate | 15/12 | 0.81  (0.14; 5.45) | Low^4^ |
| **76** | **LGG+SM vs All-PRB** | NA | NA | NA | NA | NA | 0.73  (0.14; 4.78) | Moderate | 18/12 | 0.73  (0.14; 4.78) | Low^4^ |
| **77** | **LGG+SM vs LCF** | NA | NA | NA | NA | NA | 0.79  (0.09; 8.33) | Low^6^ | 19/12 | 0.79  (0.09; 8.33) | Very Low^4,6^ |
| **78** | **LGG+SM vs YOG** | NA | NA | NA | NA | NA | 1.23  (0.12; 14.74) | Low | 15/12/16 | 1.23  (0.12; 14.74) | Very Low^4^ |
| **79** | **LGG+SM vs PRE** | NA | NA | NA | NA | NA | 0.58  (0.06; 5.63) | Moderate | 10/12 | 0.58  (0.06; 5.63) | Low^4^ |
| **80** | **STND vs MN** | NA | NA | NA | NA | NA | 0.8  (0.05; 11.79) | Moderate | 2/10 | 0.8  (0.05; 11.79) | Low^4^ |
| **81** | **STND vs YOG** | NA | NA | NA | NA | NA | 2.49  (0.4; 16.68) | Low | 16/15 | 2.49  (0.4; 16.68) | Very Low^4^ |
| **82** | **STND vs LGG+SM** | NA | NA | NA | NA | NA | 2.03  (0.31; 11.75) | Very Low | 11/12 | 2.03  (0.31; 11.75) | Very Low^4^ |
| **83** | **ZN+MN vs RC** | NA | NA | NA | NA | NA | 1.32  (0.27; 6.19) | Low | 4/2 | 1.32  (0.27; 6.19) | Very Low^4^ |
| **84** | **ZN+MN vs SM** | NA | NA | NA | NA | NA | 0.13  (0; 4.1) | Low | 6/2 | 0.13  (0; 4.1) | Very Low^4^ |
| **85** | **ZN+MN vs LOP** | NA | NA | NA | NA | NA | 0.88  (0.2; 3.83) | Moderate | 7/2 | 0.88  (0.2; 3.83) | Low^4^ |
| **86** | **ZN+MN vs ZN** | NA | NA | NA | NA | NA | 0.59  (0.15; 2.05) | Moderate^6^ | 8/2 | 0.59  (0.15; 2.05) | Low^4^ |
| **87** | **ZN+MN vs LGG** | NA | NA | NA | NA | NA | 1.59  (0.31; 7.48) | Moderate | 12/2 | 1.59  (0.31; 7.48) | Low^4^ |
| **88** | **ZN+MN vs SB** | NA | NA | NA | NA | NA | 2.17  (0.49; 10.07) | Moderate | 15/2 | 2.17  (0.49; 10.07) | Low^4^ |
| **89** | **ZN+MN vs All-PRB** | NA | NA | NA | NA | NA | 1.91  (0.48; 9.46) | Low | 18/2 | 1.91  (0.48; 9.46) | Very Low^4^ |
| **90** | **ZN+MN vs LCF** | NA | NA | NA | NA | NA | 2.11  (0.34; 13.23) | Moderate | 19/2 | 2.11  (0.34; 13.23) | Low^4^ |
| **91** | **ZN+MN vs YOG** | NA | NA | NA | NA | NA | 3.25  (0.38; 30) | Low | 15/2 | 3.25  (0.38; 30) | Very Low^4^ |
| **92** | **ZN+MN vs PRE** | NA | NA | NA | NA | NA | 1.54  (0.28; 8.39) | Moderate | 10/2 | 1.54  (0.28; 8.39) | Low^4^ |
| **93** | **ZN+MN vs LGG+SM** | NA | NA | NA | NA | NA | 2.69  (0.29; 21.97) | Moderate | 12/2 | 2.69  (0.29; 21.97) | Low^4^ |
| **94** | **SB+ZN vs RC** | NA | NA | NA | NA | NA | 3.55  (0.54; 24.96) | Very Low^6^ | 4/8 | 3.55  (0.54; 24.96) | Very Low^4,6^ |
| **95** | **SB+ZN vs SM** | NA | NA | NA | NA | NA | 0.35  (0; 14.05) | Very Low^6^ | 6/8 | 0.35  (0; 14.05) | Very Low^4,6^ |
| **96** | **SB+ZN vs LOP** | NA | NA | NA | NA | NA | 2.37  (0.38; 15.34) | Low | 7/8 | 2.37  (0.38; 15.34) | Very Low^4^ |
| **97** | **SB+ZN vs MN** | NA | NA | NA | NA | NA | 2.82  (0.12; 64.11) | Low | 2/8/10 | 2.82  (0.12; 64.11) | Very Low^4^ |
| **98** | **SB+ZN vs LGG** | NA | NA | NA | NA | NA | 4.27  (0.62; 29.68) | Low | 12/8 | 4.27  (0.62; 29.68) | Very Low^4^ |
| **99** | **SB+ZN vs SB** | NA | NA | NA | NA | NA | 5.85  (0.92; 40.64) | Very Low^6^ | 15/8 | 5.85  (0.92; 40.64) | Very Low^4,6^ |
| **100** | **SB+ZN vs All-PRB** | NA | NA | NA | NA | NA | 5.24  (0.89; 37.61) | Very Low^6^ | 18/8 | 5.24  (0.89; 37.61) | Very Low^4,6^ |
| **101** | **SB+ZN vs LCF** | NA | NA | NA | NA | NA | 5.67  (0.7; 48.8) | Very Low^6^ | 19/8 | 5.67  (0.7; 48.8) | Very Low^4,6^ |
| **102** | **SB+ZN vs YOG** | NA | NA | NA | NA | NA | 8.76  (0.79; 113.6) | Very Low^6^ | 15/8/16 | 8.76  (0.79; 113.6) | Very Low^4,6^ |
| **103** | **SB+ZN vs PRE** | NA | NA | NA | NA | NA | 4.15  (0.56; 33.08) | Low | 10/8 | 4.15  (0.56; 33.08) | Very Low^4^ |
| **104** | **SB+ZN vs LGG+SM** | NA | NA | NA | NA | NA | 7.19  (0.62; 80.91) | Low | 12/8 | 7.19  (0.62; 80.91) | Very Low^4^ |
| **105** | **SB+ZN vs STND** | NA | NA | NA | NA | NA | 3.53  (0.75; 18.12) | Low | 9/8 | 3.53  (0.75; 18.12) | Very Low^4^ |
| **106** | **SB+ZN vs ZN+MN** | NA | NA | NA | NA | NA | 2.69  (0.39; 20.27) | Low | 2/8/10 | 2.69  (0.39; 20.27) | Very Low^4^ |
| **107** | **YOG+PRB+ZN vs RC** | NA | NA | NA | NA | NA | 6.13  (0.57; 71.02) | Very Low^6^ | 4/8 | 6.13  (0.57; 71.02) | Very Low^4,6^ |
| **108** | **YOG+PRB+ZN vs SM** | NA | NA | NA | NA | NA | 0.6  (0; 33.74) | Very Low^6^ | 6/8 | 0.6  (0; 33.74) | Very Low^4,6^ |
| **109** | **YOG+PRB+ZN vs LOP** | NA | NA | NA | NA | NA | 4.08  (0.4; 45.85) | Very Low | 7/8 | 4.08  (0.4; 45.85) | Very Low^4^ |
| **110** | **YOG+PRB+ZN vs ZN** | NA | NA | NA | NA | NA | 2.75  (0.34; 22.7) | Very Low^6^ | 3/8 | 2.75  (0.34; 22.7) | Very Low^4,6^ |
| **111** | **YOG+PRB+ZN vs MN** | NA | NA | NA | NA | NA | 4.79  (0.16; 154.2) | Very Low | 2/8/10 | 4.79  (0.16; 154.2) | Very Low^4^ |
| **112** | **YOG+PRB+ZN vs LGG** | NA | NA | NA | NA | NA | 7.42  (0.64; 83.72) | Very Low | 12/8 | 7.42  (0.64; 83.72) | Very Low^4^ |
| **113** | **YOG+PRB+ZN vs SB** | NA | NA | NA | NA | NA | 10.02  (0.96; 117.8) | Very Low^6^ | 15/8 | 10.02  (0.96; 117.8) | Very Low^4,6^ |
| **114** | **YOG+PRB+ZN vs All-PRB** | NA | NA | NA | NA | NA | 9.02  (0.92; 111.3) | Very Low^6^ | 18/8 | 9.02  (0.92; 111.3) | Very Low^4,6^ |
| **115** | **YOG+PRB+ZN vs LCF** | NA | NA | NA | NA | NA | 9.79  (0.77; 136.5) | Very Low^6^ | 19/8 | 9.79  (0.77; 136.5) | Very Low^4,6^ |
| **116** | **YOG+PRB+ZN vs YOG** | NA | NA | NA | NA | NA | 15.15  (0.88; 300.8) | Very Low^6^ | 15/8/16 | 15.15  (0.88; 300.8) | Very Low^4,6^ |
| **117** | **YOG+PRB+ZN vs PRE** | NA | NA | NA | NA | NA | 7.19  (0.58; 96.79) | Very Low | 10/8 | 7.19  (0.58; 96.79) | Very Low^4^ |
| **118** | **YOG+PRB+ZN vs LGG+SM** | NA | NA | NA | NA | NA | 12.44  (0.72; 217.2) | Very Low | 12/8 | 12.44  (0.72; 217.2) | Very Low^4^ |
| **119** | **YOG+PRB+ZN vs STND** | NA | NA | NA | NA | NA | 6.08  (0.73; 57.14) | Very Low | 9/8 | 6.08  (0.73; 57.14) | Very Low^4^ |
| **120** | **YOG+PRB+ZN vs ZN+MN** | NA | NA | NA | NA | NA | 4.64  (0.41; 58.03) | Very Low | 9/8 | 4.64  (0.41; 58.03) | Very Low^4^ |

**+** This column displays the direct comparisons GRADE Assessments that informed the indirect estimates GRADE assessment. Numbers are showing the number ID of the direct comparison (which are all between 1-18)

**OR:** Odds Ratio; **95CrI%:** 95% Credible Intervals; **I^2^**: I-square statistic for heterogeneity in direct comparisons; **NMA:** Network Meta-analysis; **NA:** Non-applicable; In **Bold** are estimates that were statistically significant. **RC:** Racecadotril; **SM:** Smectite, **LOP:** Loperamide; **ZN**: Zinc; **MN:** Micronutrients **LGG:** *Lactobacillus rhamnosus* –GG; **SB:** *Sacharomyces boulardii*; **ALL-PRB**: All probiotics (except LGG and SB); **LCF:** lactose-free formula; **PRE:** prebiotics; **LGG+SM**: LGG+Smectite; **STND:** Standard treatment or Placebo**; ZN+MN:** Zinc + micronutrients; **SB+ZN:** *S. boulardii* + Zinc. **YOG+PRB+ZN:** yogurt + probiotics +zinc

**GRADE Assessment:** Reasons for downgrading direct evidence(1 to 5), indirect (6) and Mixed estimates(4, 7): 1. Downgraded because of Risk of Bias; 2. Downgraded because of Inconsistency; 3. Downgraded because of Indirectness; 4. Downgraded because of Imprecision; 5. Downgraded because of Publication Bias; 6. Downgraded because of Intransitivity; 7. Downgraded because of Incoherence; NOTE: When a superscript is more than one for an estimate, means that the criterion was downgraded 2 points (-2), instead of one point (-1)

# Table O: Assessment of Incoherence for indirect comparisons (Vomiting)

| **Comparison** | **Z-value** | **P- value** |
| --- | --- | --- |
| STND vs RC | -0.1 | 0.921 |
| LOP vs RC | 0.1 | 0.921 |
| STND vs LOP | 0.1 | 0.921 |
| STND vs LGG | -0.2 | 0.840 |
| SB vs LGG | -0.19 | 0.853 |
| All-PRB vs LGG | -0.95 | 0.341 |
| STND vs SB | -0.92 | 0.358 |
| All-PRB vs SB | -1.03 | 0.304 |
| STND vs All-PRB | 1.17 | 0.240 |
| Whole Network |  | 0.60 |

Incoherence between the direct and indirect estimates in the network was evaluated using both the global test random-effects design-by-treatment interaction model (Veroniki 2013) for the whole network and with the node-splitting method (Dias 2010) for local assessment.

# Fig H: NMA Forest plots of all interventions vs. STND for Side effects

**– # 4419 patients, # 10 treatments, # 17 studies–**

**
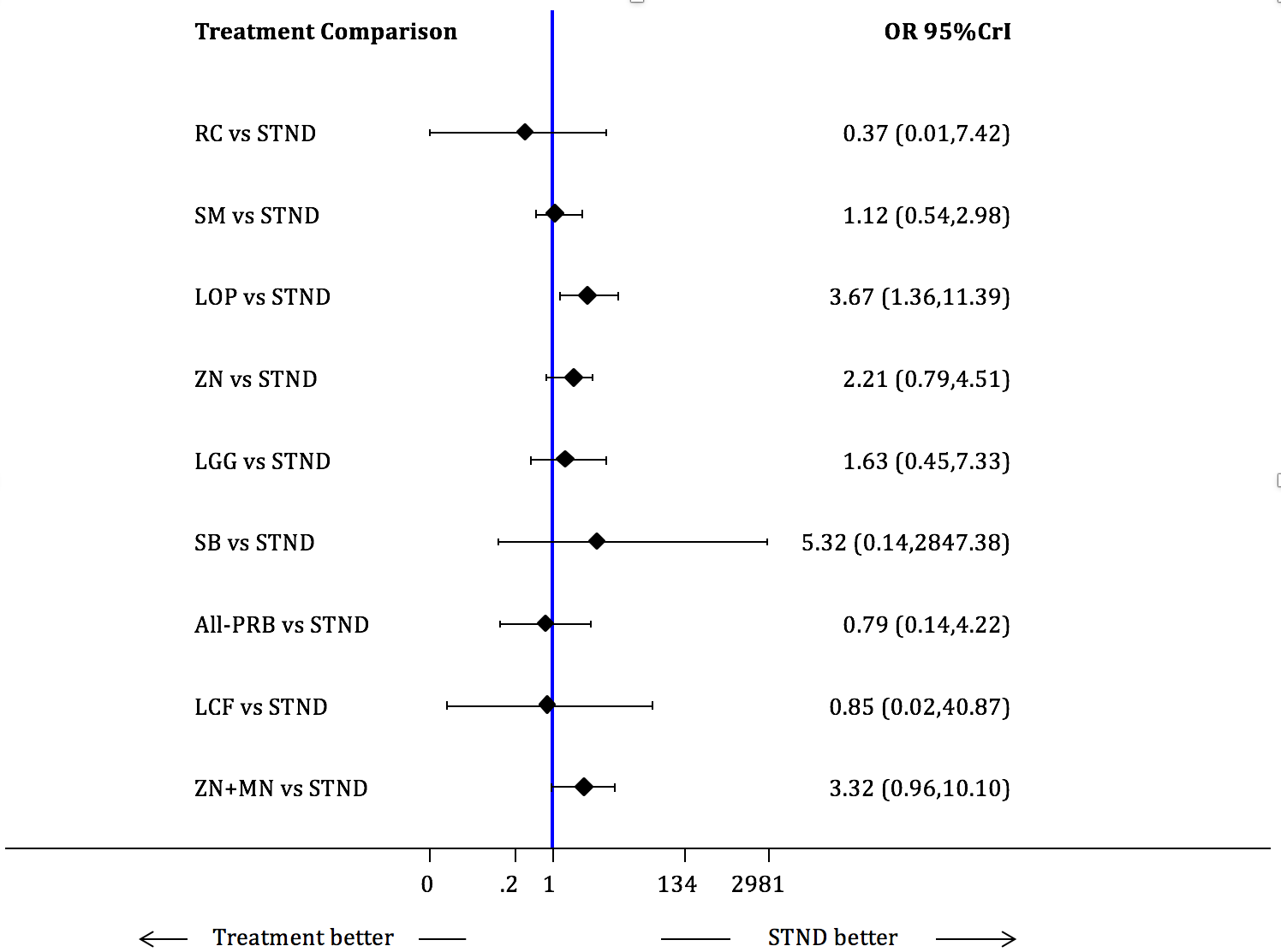
**

**OR:** Odds Ratio; **95CrI%:** 95% Credible Intervals; **RC:** Racecadotril; **SM:** Smectite, **LOP:** Loperamide; **ZN**: Zinc; **LGG:** *Lactobacillus rhamnosus*–GG; **SB:** *Sacharomyces boulardii*; **ALL-PRB**: All probiotics (except LGG and SB); **LCF:** lactose-free formula; **STND:** Standard treatment or Placebo**; ZN+MN:** Zinc + micronutrients.

# Table P: Direct, Indirect, and NMA Estimates for Side effects with the GRADE Assessment

| **#** | **Comparison** | **Direct Estimates** OR (95%CrI) | **I^2^** | **Number of**  **studies** | **Number of Patients** | **Direct GRADE** | **Indirect**  **Estimates**  OR (95%CrI) | **Indirect GRADE** | **+ Direct GRADE that informs Indirect** | **NMA estimates**  OR (95%CrI) | **NMA GRADE** |
| --- | --- | --- | --- | --- | --- | --- | --- | --- | --- | --- | --- |
| **1** | **STND vs RC** | 2.52  (0.21; 71.94) | -- | 1 | 179 | Very Low^1,1,4^ | 2.68  (0.13; 93.92) | Very Low^4^ | -- | 2.68  (0.13; 93.92 | Very Low^1,1^ |
| **2** | **STND vs SM** | 0.90  (0.32; 1.87) | 0% | 3 | 670 | Low^1,4^ | 0.89  (0.34; 1.86) | Low^,4^ | -- | 0.89  (0.34; 1.86) | Low^4^ |
| **3** | **STND vs LOP** | **0.27**  **(0.09; 0.74)** | 0% | 4 | 666 | **Moderate^1^** | **0.27**  **(0.09; 0.74)** | **Moderate** | -- | **0.27**  **(0.09; 0.74)** | **Moderate** |
| **4** | **ZN+MN vs ZN*** | 1.20  (0.86; 1.67) | -- | 1 | 889 | High | **-2.54**  **(-4.66; -0.42)** | **Moderate** | 5/10 | **1.47**  **(0.52; 5.35)** | **Low** |
| **5** | **STND vs ZN** | 0.45  (0.22; 1.27) | 72% | 3 | 1204 | Moderate^2^ | 0.45  (0.22; 1.27) | Moderate | 4/10 | 0.45  (0.22; 1.27) | Moderate |
| **6** | **STND vs LGG** | 0.61  (0.13; 2.27) | 0% | 2 | 712 | Moderate^4^ | 0.61  (0.14; 2.24) | Moderate^4^ | -- | 0.61  (0.14; 2.24) | Moderate^4^ |
| **7** | **STND vs SB** | 0.19  (0.00; 6.12) | -- | 1 | 202 | Low^1,4^ | 0.19  (0.00; 7.39) | Low^4^ | -- | 0.19  (0.00; 7.39) | Low^4^ |
| **8** | **STND vs All-PRB** | 1.26  (0.24; 7.10) | 0% | 2 | 274 | Low^1,4^ | 1.26  (0.24; 7.05) | Low^4^ | -- | 1.26  (0.24; 7.05) | Low^4^ |
| **9** | **STND vs LCF** | 1.11  (0.03; 50.57) | -- | 1 | 73 | Moderate^4^ | 1.18  (0.02; 49.36) | Moderate^4^ | -- | 1.18  (0.02; 49.36) | Moderate^4^ |
| **10** | **ZN+MN vs STND*** | 4.44  (2.87; 7.12) | -- | 1 | 896 | Moderate^4^ | 0.52  (-1.22; 2.25) | Moderate | 4/5 | 3.32  (0.96; 10.1) | Low |
| **11** | **SM vs RC** | NA | NA | NA | NA | NA | 3.11  (0.14; 116.1) | Very Low^4,6^ | 2/1 | 3.11  (0.14; 116.1) | Very Low^4,6^ |
| **12** | **LOP vs RC** | NA | NA | NA | NA | NA | 10.05  (0.43; 406.7) | Very Low^4,6^ | 3/1 | 10.05  (0.43; 406.7) | Very Low^4,6^ |
| **13** | **LOP vs SM** | NA | NA | NA | NA | NA | 3.21  (0.83; 12.64) | Low^4^ | 2/3 | 3.21  (0.83; 12.64) | Low^4^ |
| **14** | **ZN vs RC** | NA | NA | NA | NA | NA | 5.7  (0.25; 222.5) | Very Low^4,6^ | 1/5 | 5.7  (0.25; 222.5) | Very Low^4,6^ |
| **15** | **ZN vs SM** | NA | NA | NA | NA | NA | 1.98  (0.44; 5.21) | Low^4^ | 2/5 | 1.98  (0.44; 5.21) | Low^4^ |
| **16** | **ZN vs LOP** | NA | NA | NA | NA | NA | 0.59  (0.12; 2) | Very Low^4,6^ | 3/5 | 0.59  (0.12; 2) | Very Low^4,6^ |
| **17** | **LGG vs RC** | NA | NA | NA | NA | NA | 4.52  (0.17; 207.4) | Very Low^4^ | 1/6 | 4.52  (0.17; 207.4) | Very Low^4^ |
| **18** | **LGG vs SM** | NA | NA | NA | NA | NA | 1.45  (0.28; 7.49) | Very Low^4,6^ | 2/6 | 1.45  (0.28; 7.49) | Very Low^4,6^ |
| **19** | **LGG vs LOP** | NA | NA | NA | NA | NA | 0.45  (0.08; 2.65) | Low^4^ | 3/6 | 0.45  (0.08; 2.65) | Low^4^ |
| **20** | **LGG vs ZN** | NA | NA | NA | NA | NA | 0.75  (0.17; 4.95) | Very Low^4,6^ | 5/6 | 0.75  (0.17; 4.95) | Very Low^4,6^ |
| **21** | **SB vs RC** | NA | NA | NA | NA | NA | 16.27  (0.14; 17210) | Very Low^4,6^ | 1/7 | 16.27  (0.14; 17210) | Very Low^4,6^ |
| **22** | **SB vs SM** | NA | NA | NA | NA | NA | 4.66  (0.1; 2759) | Low^4^ | 2/7 | 4.66  (0.1; 2759) | Low^4^ |
| **23** | **SB vs LOP** | NA | NA | NA | NA | NA | 1.45  (0.03; 854.6) | Low^4^ | 3/7 | 1.45  (0.03; 854.6) | Low^4^ |
| **24** | **SB vs ZN** | NA | NA | NA | NA | NA | 2.55  (0.06; 1720) | Low^4^ | 5/7 | 2.55  (0.06; 1720) | Low^4^ |
| **25** | **SB vs LGG** | NA | NA | NA | NA | NA | 3.38  (0.06; 2196) | Very Low^4,6^ | 6/7 | 3.38  (0.06; 2196) | Very Low^4,6^ |
| **26** | **All-PRB vs RC** | NA | NA | NA | NA | NA | 2.15  (0.07; 102.9) | Very Low^4,6^ | 1/8 | 2.15  (0.07; 102.9) | Very Low^4,6^ |
| **27** | **All-PRB vs SM** | NA | NA | NA | NA | NA | 0.7  (0.1; 4.06) | Very Low^4,6^ | 2/8 | 0.7  (0.1; 4.06) | Very Low^4,6^ |
| **28** | **All-PRB vs LOP** | NA | NA | NA | NA | NA | 0.21  (0.03; 1.56) | Very Low^4,6^ | 3/8 | 0.21  (0.03; 1.56) | Very Low^4,6^ |
| **29** | **All-PRB vs ZN** | NA | NA | NA | NA | NA | 0.36  (0.06; 2.63) | Very Low^4,6^ | 5/8 | 0.36  (0.06; 2.63) | Very Low^4,6^ |
| **30** | **All-PRB vs LGG** | NA | NA | NA | NA | NA | 0.48  (0.05; 3.87) | Very Low^4,6^ | 6/8 | 0.48  (0.05; 3.87) | Very Low^4,6^ |
| **31** | **All-PRB vs SB** | NA | NA | NA | NA | NA | 0.14  (0; 7.9) | Very Low^4,6^ | 7/8 | 0.14  (0; 7.9) | Very Low^4,6^ |
| **32** | **LCF vs RC** | NA | NA | NA | NA | NA | 2.34  (0.02; 365) | Very Low^4,6^ | 1/9 | 2.34  (0.02; 365) | Very Low^4,6^ |
| **33** | **LCF vs SM** | NA | NA | NA | NA | NA | 0.73  (0.02; 38.83) | Low^4^ | 2/9 | 0.73  (0.02; 38.83) | Low^4^ |
| **34** | **LCF vs LOP** | NA | NA | NA | NA | NA | 0.23  (0; 12.53) | Low^4^ | 3/9 | 0.23  (0; 12.53) | Low^4^ |
| **35** | **LCF vs ZN** | NA | NA | NA | NA | NA | 0.4  (0.01; 21.74) | Low^4^ | 5/9 | 0.4  (0.01; 21.74) | Low^4^ |
| **36** | **LCF vs LGG** | NA | NA | NA | NA | NA | 0.51  (0.01; 31.44) | Low^4,6^ | 6/9 | 0.51  (0.01; 31.44) | Low^4,6^ |
| **37** | **LCF vs SB** | NA | NA | NA | NA | NA | 0.13  (0; 33.67) | Low^4^ | 7/9 | 0.13  (0; 33.67) | Low^4^ |
| **38** | **LCF vs All-PRB** | NA | NA | NA | NA | NA | 1.08  (0.02; 74.76) | Very Low^4,6^ | 8/9 | 1.08  (0.02; 74.76) | Very Low^4,6^ |
| **39** | **ZN+MN vs RC** | NA | NA | NA | NA | NA | 8.85  (0.36; 351.9) | Very Low^4,6^ | 1/10 | 8.85  (0.36; 351.9) | Very Low^4,6^ |
| **40** | **ZN+MN vs SM** | NA | NA | NA | NA | NA | 2.99  (0.55; 10.5) | Low^4^ | 2/10 | 2.99  (0.55; 10.5) | Low^4^ |
| **41** | **ZN+MN vs LOP** | NA | NA | NA | NA | NA | 0.91  (0.16; 3.77) | Low^4^ | 3/10 | 0.91  (0.16; 3.77) | Low^4^ |
| **42** | **ZN+MN vs LGG** | NA | NA | NA | NA | NA | 2.03  (0.28; 10.68) | Low^4,6^ | 6/10 | 2.03  (0.28; 10.68) | Low^4,6^ |
| **43** | **ZN+MN vs SB** | NA | NA | NA | NA | NA | 0.61  (0; 27.65) | Low^4^ | 7/10 | 0.61  (0; 27.65) | Low^4^ |
| **44** | **ZN+MN vs All-PRB** | NA | NA | NA | NA | NA | 4.17  (0.53; 31.7) | Very Low^4,6^ | 8/10 | 4.17  (0.53; 31.7) | Very Low^4,6^ |
| **45** | **ZN+MN vs LCF** | NA | NA | NA | NA | NA | 3.89  (0.07; 177.5) | Moderate^4^ | 9/10 | 3.89  (0.07; 177.5) | Moderate^4^ |

** Comparisons with Incoherence between Direct and indirect evidence (p=0.039).*

**+** This column displays the direct comparisons GRADE Assessments that informed the indirect estimates GRADE assessment. Numbers are showing the number ID of the direct comparison (which are all between 1-18)

**OR:** Odds Ratio; **95CrI%:** 95% Credible Intervals; **NMA:** Network Meta-analysis; **NA:** Non-applicable; In **Bold** are estimates that were statistically significant. **RC:** Racecadotril; **SM:** Smectite, **LOP:** Loperamide; **ZN**: Zinc; **LGG:** *Lactobacillus rhamnosus*–GG; **SB:** *Sacharomyces boulardii*; **ALL-PRB**: All probiotics (except LGG and SB); **LCF:** lactose-free formula; **STND:** Standard treatment or Placebo**; ZN+MN:** Zinc + micronutrients.

**GRADE Assessment:** Reasons for downgrading direct evidence(1 to 5), indirect (6) and Mixed estimates(4, 7): 1. Downgraded because of Risk of Bias; 2. Downgraded because of Inconsistency; 3. Downgraded because of Indirectness; 4. Downgraded because of Imprecision; 5. Downgraded because of Publication Bias; 6. Downgraded because of Intransitivity; 7. Downgraded because of Incoherence; NOTE: When a superscript is more than one for an estimate, means that the criterion was downgraded 2 points (-2), instead on one point (-1)

# Table Q: Assessment of Incoherence for Indirect Comparisons (Side Effects)

| **Comparison** | **Z-value** | **P- value** |
| --- | --- | --- |
| ZN+MN vs ZN | 2.08 | 0.037 |
| ZN+MN vs STND | -2.08 | 0.037 |
| **Whole Network** |  | **0.009** |

Incoherence between the direct and indirect estimates in the network was evaluated using both the global test random-effects design-by-treatment interaction model (Veroniki 2013) for the whole network and with the node-splitting method (Dias 2010) for local assessment.

# Table R: Sensitivity Analyses & SUCRA for Secondary outcomes

| **Intervention** | **Stool Frequency at day 2** | | **Diarrhea at day 3** | | **Vomiting** | | **Side Effects** | |
| --- | --- | --- | --- | --- | --- | --- | --- | --- |
|  | **All studies** | **RCTs** | **All studies** | **RCTs** | **All studies** | **RCTs** | **All studies** | **RCTs** |
| **RC** | 0.45 (0.09; 1.00) | 0.40 (0.00; 1.00) | -- | -- | 0.53 (0.13; 0.93) | 0.54 (0.15; 1.00) | 0.89 (0.11; 1.00) | 0.89 (0.00; 1.00) |
| **SM** | 0.64 (0.09; 1.00) | 0.60 (0.00; 1.00) | 0.91 (0.45; 1.00) | 1.00 (0.45; 1.00) | 0.00 (0.00; 0.93) | 0.00 (0.00; 0.92) | 0.67 (0.22; 0.89) | 0.67 (0.22; 0.89) |
| **LOP** | -- | -- | 0.27 (0.00; 1.00) | 0.27 (0.00; 1.00) | 0.33 (0.07; 0.80) | 0.31(0.08; 0.77) | 0.11 (0.00; 0.56) | 0.22 (0.00; 0.78) |
| **ZN** | 0.09 (0.09; 0.91) | 0.50 (0.00; 1.00) | 0.45 (0.18; 0.73) | 0.45 (0.18; 0.73) | 0.20 (0.07; 0.47) | 0.15 (0.00; 0.46) | 0.33 (0.11; 0.78) | 0.33 (0.11; 0.78) |
| **MN** | -- | -- | -- | -- | 0.40 (0.00; 1.00) | 0.38 (0.00; 1.00) | -- | -- |
| **LGG** | 0.55 (0.09; 1.00) | 0.50 (0.00; 1.00) | 0.36 (0.00; 0.82) | 0.36 (0.00; 0.91) | 0.60 (0.20; 0.93) | 0.62 (0.15; 0.92) | 0.44 (0.00; 0.89) | 0.44 (0.00; 0.89) |
| **SB** | 0.55 (0.09; 1.00) | 0.50 (0.00; 1.00) | 0.91 (0.64; 1.00) | 0.91 (0.55; 1.00) | 0.80 (0.33; 1.00) | 0.77 (0.31; 1.00) | 0.11 (0.00; 1.00) | 0.11 (0.00; 1.00) |
| **All-PRB** | 0.64 (0.27; 1.00) | 0.60 (0.20; 1.00) | 0.73 (0.45; 0.91) | 0.73 (0.36; 0.91) | 0.73 (0.23; 1.00) | 0.77 (0.31; 1.00) | 0.78 (0.11; 1.00) | 0.78 (0.11; 1.00) |
| **SYM** | 0.73 (0.09; 1.00) | 0.70 (0.00; 1.00) | 0.73 (0.27; 1.00) | 0.73 (0.27; 1.00) | -- | -- | -- | -- |
| **LCF** | 0.73 (0.18; 1.00) | 0.70 (0.10; 1.00) | 0.09 (0.00; 0.91) | 0.09 (0.00; 0.91) | 0.80 (0.20; 1.00) | 0.77 (0.15; 1.00) | 0.78 (0.00; 1.00) | 0.78 (0.00; 1.00) |
| **YOG** | 0.73 (0.18; 1.00) | 0.70 (0.10; 1.00) | 0.36 (0.00; 0.82) | 0.36 (0.00; 0.91) | 0.93 (0.20; 1.00) | -- | -- | -- |
| **PRE** | -- | -- | -- | -- | 0.60 (0.13; 1.00) | 0.62 (0.15; 1.00) | -- | -- |
| **LGG+SM** | -- | -- | -- | -- | 0.87 (0.13; 1.00) | 0.92 (0.15; 1.00) | -- | -- |
| **ZN+PRB** | 0.64 (0.09; 1.00) | 0.20 (0.00; 1.00) | 0.45 (0.00; 1.00) | 0.45 (0.00; 1.00) | -- | -- | -- | -- |
| **STND** | 0.18 (0.09; 0.45) | 0.10 (0.00; 0.40) | 0.18 (0.00; 0.36) | 0.18 (0.00; 0.36) | 0.53 (0.33; 0.80) | 0.54 (0.31; 0.77) | 0.67 (0.44; 0.89) | 0.67 (0.44; 0.89) |
| **ZN+MN** | -- | -- | 0.36 (0.00; 0.91) | 0.36 (0.00; 0.91) | 0.40 (0.07; 0.87) | 0.38 (0.08; 0.92) | 0.22 (0.00; 0.67) | 0.22 (0.00; 0.67) |
| **CAO** | 0.0 (0.00; 0.09) | -- | -- | -- | -- | -- | -- | -- |
| **SB+ZN** | -- | -- | -- | -- | 0.13 (0.00; 0.67) | 0.08 (0.00; 0.69) | -- | -- |
| **YOG+PRB+ZN** | -- | -- | -- | -- | 0.07 (0.00; 0.67) | -- | -- | -- |
| **Heterogeneity**  **Tau^2^ (95%CrI)** | 0.90 (0.39; 2.03) | 0.90 (0.38; 2.1) | 0.57 (0.29; 1.10) | 0.63 (0.32; 1.25) | 0.18 (0.02; 0.92) | 0.19 (0.03; 0.93) | **0.19 (0.00; 1.48)** | **0.21 (0.00; 1.56)** |

SUCRA values are presented along with the 95%CrI. **All Studies:** Analysis based on all the studies that reported the outcome. **RCTs;** Analyses when excluding qRCTs**.** Global heterogeneity assessed with the common within-network between-study variance (Tau2; 95%CrI). **RC:** racecadotril; **SM:** Smectite, **LOP:** Loperamide; **ZN**: Zinc; **MN:** Micronutrients; **LGG:** *Lactobacillus rhamnosus* –GG; **SB:** *Sacharomyces boulardii*; **ALL-PRB**: All probiotics except SB and LGG; **SYM:** Symbiotics, **LCF:** lactose-free formula; **YOG:** Yogurt; **PRE:** prebiotics; **LGG+SM**: LGG+Smectite; **ZN+PRB:** Zinc + Probiotics; **STND:** Standard treatment or Placebo**; ZN+MN:** Zinc + micronutrients; **CAO:** Kaolin-Pectin; **SB+ZN:** *S. boulardii* + Zinc; **YOG+PRB+ZN:** Yogurt + Probiotics + Zinc

# Fig I: Rank Heat Plot

**
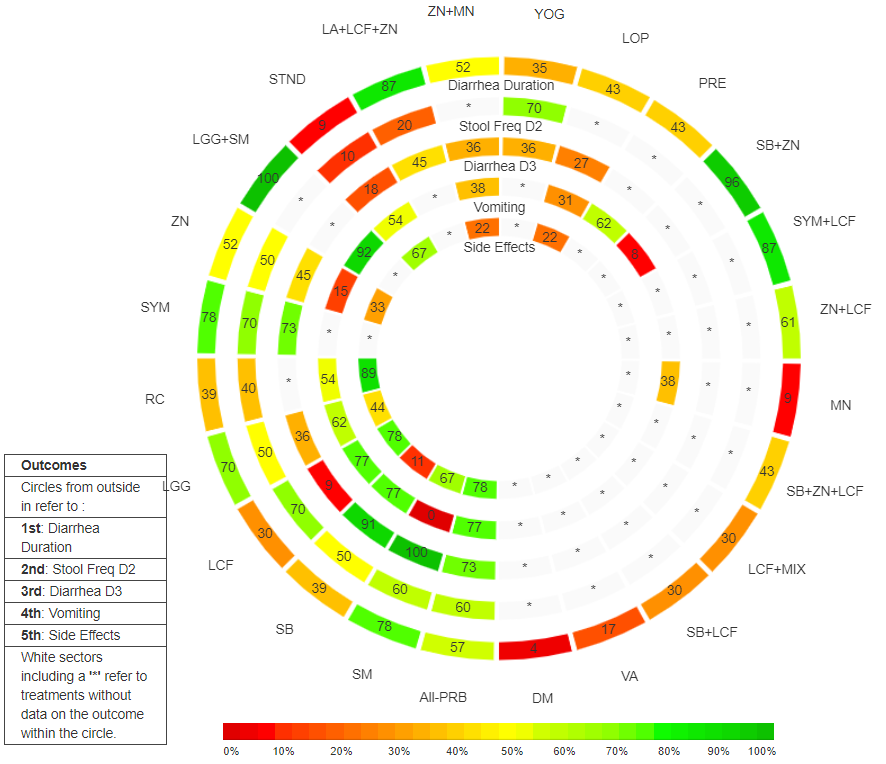
**

Each concentric circle represents a different outcome (as labeled), with the outermost circle representing the diarrhea duration, and the innermost circle representing the side effects. The scale bar represents the ranking statistic for each intervention using the P-scores, where 0% (red) indicates the lowest possible rank (worst treatment), and 100% (green) represents the highest possible rank (best treatment). Each rectangle represents an intervention and is coded using a letter outside the outmost circle (see treatment legend). The number within each rectangle represents the ranking statistic of the intervention for the particular outcome circle. See Table 1 for the coding guide of the treatments.

# Table S: Results for Diarrhea at day 7

| **First author** | **Year** | **Interventions** | **Definition of treatment failure** |
| --- | --- | --- | --- |
| Allen | 1994 | Group 1 LCF Group 2 STND | Diarrhea > 7 days: Soy milk: 5/39, Cow's milk 13/34 |
| Bahl | 2002 | Group 1 ZN Group 2 ZN2 Group 3 STND | Number of episodes lasting >= 7 days: Control group 28/401, ZincORs: 17/402; Zinc Syrup: 19/404 |
| Bhatnagar | 2004 | Group 1 ZN+MN Group 2 STND | Number of episodes lasting >= 7 days: Zinc 1/132, Placebo: 9/134 |
| Fayad | 1999 | Group 1 STND Group 2 SYM | Diarrhea on day 7: Soy formula 0/100; Sucrose formula: 1/100 |
| Guarino | 2001 | Group 1 SM Group 2 STND | Diarrhea Lasting more than 7 days: Smectite 44/406; Placebo: 72/398 |
| Henker | 2008 | Group 1 AL-PRB Group 2 STND | Response (cure) on day 7: Ecoli-Nissle 59/75, Placebo 45/76 |
| Patel | 2009 | Group 1 ZN Group 2 ZN2 Group 3 STND | Number and Proportion with diarrhea of > 7 days from Onset: Placebo; 14/ 217; Zinc+Copper 8/273; Zinc 10/264 |
| Patro | 2010 | Group 1 ZN Group 2 STND | The proportion of children with diarrhea lasting >7 days: Zinc 1/69; placebo 3/69 |
| Polat | 2003 | Group 1 ZN Group 2 STND | Cases of diarrhea post-enrollement duration of diarrhea > 7 days: zinc 5/40, placebo 16/20. RV+: zinc 8/52, placebo 20/54 |
| Santos | 2009 | Group 1 RC Group 2 STND | Resoluction fo diarrhea After day 7: Racecadotril 35/50; Placebo 30/53 |
| Sazawal | 1995 | Group 1 ZN+MN Group 2 MN | Episodes lasting >7 days (% of children): Zinc. 70/456, Placebo: 90/481 |
| Simakachorn | 2004 | Group 1 LCF Group 2 STND | Diarrhea not resolved after 7 days of treatment: Lactose Free-formula 0/40: Infant formula: 3/40 |
| Strand | 2002 | Group 1 ZN Group 2 ZN+MN Group 3 STND | Cases with postenrollment duration > 7 days: zinc: RR 0.57 (CI. 0.38; 0.86); zinc + vit A: RR 0.53 (CI 0.35; 0.81); placebo; 58/ |
| Szymanski | 2006 | Group 1 AL-PRB Group 2 STND | Diarrhea lasting over 7 days: number of patients: L. Rhamnossus 4/68; Placebo: 8/58 |
| Villarruel | 2007 | Group 1 SB Group 2 STND | Prolonged diarrhea > 7 days: S. Boulardii 3/44, Placebo: 31244 |

# Table T: Results for Treatment Failure

| **First author** | **Year** | **Interventions** | **Definition of treatment failure** |
| --- | --- | --- | --- |
| Agustina | 2007 | Group 1 SYM+LCF Group 2 LCF | Diarrhea > Day 7, fecal output > 100 g/kg on D6, clinical evidence of dehydration again that required iIV, or vomited for > 3 times in an 8 hour period |
| Allen | 1994 | Group 1 LCF Group 2 STND | (1) at least three episodes of vomiting per 8-h period while on study formula resulting in the reinstitution of clear fluids (34); (2) refusal to accept the study formula; the need for iv fluid balance due to diarrhea or vomiting; (3) diarrhea persisting for more than seven days following enroll- ment (34) |
| Armitstead | 1989 | Group 1 LCF Group 2 DM Group 3 STND | More than 6 stools in 24 hours or without CHO intolerance or the return of severe vomiting requiring rehydration |
| Awasthi | 2006 | Group 1 VA Group 2 STND | Treatment failure=one or more than of following dehydration, need for hospitalization, need for intravenous fluids, death, at any time |
| Bhatnagar | 1998 | Group 1 SB+ZN Group 2 YOG | having either an episode of dehydration after an initial 24 hours on study diets or stool weight greater than 150 gm/kg body weight on any day |
| Boudraa | 2001 | Group 1 STND Group 2 YOG | Failure of enteral feeding was defined as a 5% loss of body weight in 24 hours, or liquid stools still present 7 days after study inclusion |
| Bowie | 1995 | Group 1 LOP Group 2 STND | ORS required to maintain Hydrat > 72h |
| Brown | 1991 | Group 1 LCF Group 2 STND | Recurrent DHT (after Rehydration)- Including Electrolytes disturbances. Severe Diarrhea (excrecion >350 gr/k/d), OR rolonged diarrhea D6: >100 g/kg&d) |
| Fayad | 1999 | Group 1 STND Group 2 LCF | Failure was defined as recurrent DHT and requiremnt of IV, + Diarrhea D7. However, it was specified, hence I used Day7, In the Diarrhea Duration Dichotomous Outcome (1/86 and 0/86), And in the Failure I only ised the children with IV necessity or Recurrent DHT |
| Guandalini | 2000 | Group 1 LGG Group 2 STND | Need to restart IV rehydration |
| Henker | 2008 | Group 1 ALL-PRB Group 2 STND | Day 7: sample size - treatment success |
| Henning | 1992 | Group 1 VA Group 2 STND | Requirement of IV fluid after Being hydrated |
| Khanna | 2005 | Group 1 ALL-PRB Group 2 STND | Requirement of > 200 ml/kg of IVF, and one of them also remained dehydrated beyond 72 hours. |
| Lozano | 1994 | Group 1 LCF Group 2 STND | Persistance of diarrhea >7d, increment or more than 50% of volume stools, persistence of vomiting in 2 consecutive opportunities after a second chance of fasting |
| Madkour | 1993 | Group 1 SM Group 2 STND | Aggravation of diarrhea, vomiting, or dehydration requiring IV. There were NO treatment failures |
| Mujawar | 2012 | Group 1 SM Group 2 STND | Diarrhea on 5th day or complication or coinfection or side effect |
| Quak | 1989 | Group 1 STND Group 2 LCF | Relapse of diarrhea (4 or more stools in 12h) |
| Ransome | 1984 | Group 1 STND Group 2 DM | Lactose malabsrption |
| Riaz | 2012 | Group 1 SB+ZN Group 2 ZN | DHT >72 h, Consumption of ORS consumption >8 L OR Need for IV unscheduled >200 ml/kg t |
| Salazar-Lindo | 2004 | Group 1 LGG Group 2 STND | proportion of patients in each study group who have recurrence or continued presence of more than 5% dehydration, worsening electrolyte abnormalities, no weight gain since admission, develop- ing of ileus or severe diarrhea defined as a purging rate in excess of 10 ml/Kg/hr in two consecutive 4-hour periods. |
| Sarker | 2005 | Group 1 ALL-PRB Group 2 STND | No. of children required unscheduled intravenous fluid |
| Simakachorn | 2004 | Group 1 LCF Group 2 STND | Number of children with Diarrhea AT D7 |
| Wall | 1994 | Group 1 LCF Group 2 LCF2 Group 3 STND | Continuing OR Increase severity of diarrhea, with Weight loss or Electrolyte fluid imbalance. NOT clear, given that was measured j the second study, not in the first STUDy (See the explanation at the end) |
| Widiasa | 2009 | Group 1 SM Group 2 STND | No recovery on day 5, complication before day 5, coinfection, adverse events or death |
| Chew | 1993 | Group 1 STND Group 2 DM | Recurrent DHT OR INCREASE Output |
| Conway | 1989 | Group 1 DM Group 2 LCF Group 3 STND Group 4 LCF2 | Continued or increased Severity of Diarrhea with WEIGHT loss or deterioration (fluid electrolyte imbalance OR BOTH |
| Dagan | 1984 | Group 1 LCF Group 2 STND | Persistence of Diarrhea OR Vomiting. HERE 2 consecutive failures |
| Dugdale | 1982 | Group 1 DM Group 2 STND | Worsening of diarrhea OR VOMITING |
| Leake | 1974 | Group 1 LCF Group 2 STND | failure was defined as six or more stools per day for three consecutive days. |
| Lifshitz | 1991 | Group 1 STND Group 2 LCF | FAILURE: induction of dehydration and the severity of the diarrhea. Dehydration that occurred during the time of refeeding was defined as a daily **weight loss >5%** of body weight that was not prevented by intravenous and oral rehydration fluids. **Severe diarrhea** was defined as liquid stool losses **>40 gm/ kg of body weight per day up to the third day of treatment.** |
| Naidoo | 1981 | Group 1 LCF Group 2 STND | Treatment was considered to have failed if the infant required another period of fasting or treatment by intravenous fluids or if there were 6 or more *watery* stools per day for 3 consecutive days |
| Placzek | 1984 | Group 1 STND Group 2 DM | Complicated: Recurrence of Severe vomiting or Watery diarrhea with 2% or Reducing Substances |
| Macedo-Prietsch | 1999 | Group 1 LCF Group 2 STND | Clinical Signs of DHT, Diarrhea >7d post Treatment OR Persistence of vomiting |
| Sutton | 1968 | Group 1 LCF Group 2 STND | Prsistent profused diarrhea after fasting |
| Zong | 1997 | Group 1 SM Group 2 LCF | Not clearly defined |
| Gu | 2011 | Group 1 ALL-PRB Group 2 SM | Not clearly defined |
| Su | 2014 | Group 1 ZN Group 2 LCF Group 3 ZN+LCF | Not clearly defined |
| Xu | 2014 | Group 1 ZN+PRB Group 2 ALL-PRB | Not clearly defined |

# Fig J: Funnel plots for all the outcomes (A, B, C, D, E)

**A. Diarrhea Duration**

**B. Stool frequency at day 2**

**C. Diarrhea at day 3**

**D. Vomiting**

**e. Side Effects**

# File D: Full references of included studies

1. Agustina R, Lukito W, Firmansyah A, Suhardjo HN, Murniati D, Bindels J. The effect of early nutritional supplementation with a mixture of probiotic, prebiotic, fiber and micronutrients in infants with acute diarrhea in Indonesia. *Asia Pacific Journal of Clinical Nutrition.* 2007;16(3):435-442.

2. Al-Sonboli N, Gurgel RQ, Shenkin A, Hart CA, Cuevas LE. Zinc supplementation in Brazilian children with acute diarrhoea. *Annals of Tropical Paediatrics.* 2003;23(1):3-8.

3. Allen UD, McLeod K, Wang EEL. Cow's milk versus soy-based formula in mild and moderate diarrhea: A randomized, controlled trial. *Acta Paediatrica, International Journal of Paediatrics.* 1994;83(2):183-187.

4. Anderson J. Double-blind comparison of loperamide HCl and placebo in the treatment of acute diarrhea in children. *Advances in Therapy.* 1984;1(1):14-18.

5. Anonymous. Loperamide in acute diarrhoea in childhood: results of a double blind, placebo controlled multicentre clinical trial. Diarrhoeal Diseases Study Group (UK). *Br Med J (Clin Res Ed).* 1984;289(6454):1263-1267.

6. Armitstead J, Kelly D, Walker-Smith J. Evaluation of infant feeding in acute gastroenteritis. *Journal of Pediatric Gastroenterology and Nutrition.* 1989;8(2):240-244.

7. Awasthi S. Zinc supplementation in acute diarrhea is acceptable, does not interfere with oral rehydration, and reduces the use of other medications: A randomized trial in five countries - INCLEN Childnet Zinc Effectiveness for Diarrhea (IC-ZED) Group. *Journal of Pediatric Gastroenterology and Nutrition.* 2006;42(3):300-305.

8. Bahl R, Bhandari N, Saksena M, et al. Efficacy of zinc-fortified oral rehydration solution in 6- to 35-month-old children with acute diarrhea. *Journal of Pediatrics.* 2002;141(5):677-682.

9. Basu S, Chatterjee M, Ganguly S, Chandra PK. Efficacy of Lactobacillus rhamnosus GG in acute watery diarrhoea of Indian children: A randomised controlled trial. *Journal of Paediatrics and Child Health.* 2007;43(12):837-842.

10. Basu S, Paul DK, Ganguly S, Chatterjee M, Chandra PK. Efficacy of high-dose Lactobacillus rhamnosus GG in controlling acute watery diarrhea in Indian children: A randomized controlled trial. *J Clin Gastroenterol.* 2009;43(3):208-213.

11. Bhandari N, Bahl R, Sazawal S, Bhan MK. Breast-feeding status alters the effect of vitamin A treatment during acute diarrhea in children. *Journal of Nutrition.* 1997;127(1):59-63.

12. Bhatnagar S, Singh KD, Sazawal S, Saxena SK, Bhan MK. Efficacy of milk versus yogurt offered as part of a mixed diet in acute noncholera diarrhea among malnourished children. *Journal of Pediatrics.* 1998;132(6):999-1003.

13. Bhatnagar S, Bahl R, Sharma PK, Kumar GT, Saxena SK, Bhan MK. Zinc with oral rehydration therapy reduces stool output and duration of diarrhea in hospitalized children: A randomized controlled trial. *Journal of Pediatric Gastroenterology and Nutrition.* 2004;38(1):34-40.

14. Billoo AG, Memon MA, Khaskheli SA, et al. Role of a probiotic (Saccharomyces boulardii) in management and prevention of diarrhoea. *World Journal of Gastroenterology.* 2006;12(28):4557-4560.

15. Boran P, Tokuc G, Vagas E, Oktem S, Gokduman MK. Impact of zinc supplementation in children with acute diarrhoea in Turkey. *Archives of Disease in Childhood.* 2006;91(4):296-299.

16. Boudraa G, Benbouabdellah M, Hachelaf W, Boisset M, Desjeux JF, Touhami M. Effect of feeding yogurt versus milk in children with acute diarrhea and carbohydrate malabsorption. *Journal of Pediatric Gastroenterology and Nutrition.* 2001;33(3):307-313.

17. Boulloche J, Mouterde O, Mallet E. Management of acute diarrhea in infants and toddlers. Controlled study of the antidiarrheal efficacy of killed Lactobacillus acidophilus (LB strain) versus a placebo and a reference agent (loperamide). [French]. *Annales de Pediatrie.* 1994;41(7):457-463.

18. Bowie MD, Hill ID, Mann MD. Loperamide for treatment of acute diarrhoea in infants and young children. A double-blind placebo-controlled trial. *South African Medical Journal.* 1995;85(9):885-887.

19. Brown KH, Perez F, Gastanaduy AS. Clinical trial of modified whole milk, lactose-hydrolyzed whole milk, or cereal-milk mixtures for the dietary management of acute childhood diarrhea. *Journal of Pediatric Gastroenterology and Nutrition.* 1991;12(3):340-350.

20. Burande M. Comparison of efficacy of Saccharomyces boulardii strain in the treatment of acute diarrhea in children: A prospective, single-blind, randomized controlled clinical trial. *Journal of Pharmacology and Pharmacotherapeutics.* 2013;4(3):205-208.

21. Canani RB, Cirillo P, Terrin G, et al. Probiotics for treatment of acute diarrhoea in children: Randomised clinical trial of five different preparations. *British Medical Journal.* 2007;335(7615):340-342.

22. Carrague-Orendain A. Randomized, double blind placebo- controlled trial on the efficacy and safety of lactobacillus (Infloran Berna capsules) in the treatment of acute non-bloody diarrhoea in children two to five years of age. *Not published.* 1999;NR(NR).

23. Cetina-Sauri G, Sierra Basto G. Evaluation of Saccharomyces boulardii for the treatment of acute diarrhea in pediatric patients. [French]. *Annales de Pediatrie.* 1994;41(6):397-400.

24. J.P C, Duhamel JF, Meyer M, et al. Efficacy and tolerability of racecadotril in acute diarrhea in children. *Gastroenterology.* 2001;120(4):799-805.

25. Chen CC, Kong MS, Lai MW, et al. Probiotics have clinical, microbiologic, and immunologic efficacy in acute infectious diarrhea. *Pediatr Infect Dis J.* 2010;29(2):135-138.

26. Chew F, Penna FJ, Peret Filho LA, et al. Is dilution of cows' milk formula necessary for dietary management of acute diarrhoea in infants aged less than 6 months? *Lancet.* 1993;341(8839):194-197.

27. Clemente YF, Tapia CC, Comino AL, López PL, Escrivá TP, González PJ. Lactose-free formula versus adapted formula in acute infantile diarrhea. *Anales españoles de pediatría.* 1993;39(4):309-312.

28. Cojocaru B, Bocquet N, Timsit S, et al. Benefit of racecadotril for acute diarrhoea treatment and emergency department visit. *Archives de Pediatrie.* 2002;9(8):774-779.

29. Conway S, Ireson A. Acute gastroenteritis in well nourished infants: comparison of four feeding regimens. *Archives of Disease in Childhood.* 1989;64(1):87-91.

30. Cordier MP, Mozziconacci G, Polonovski C. Indications of loperamide in acute infantile diarrhea. A randomized double blind trial. [French]. *Annales de Pediatrie.* 1987;34(8):653-658.

31. Correa NBO, Penna FJ, Lima FMLS, Nicoli JR, Filho LAP. Treatment of acute diarrhea with saccharomyces boulardii in infants. *Journal of Pediatric Gastroenterology and Nutrition.* 2011;53(5):497-501.

32. Costa-Ribeiro H, Ribeiro TCM, Mattos AP, et al. Limitations of probiotic therapy in acute, severe dehydrating diarrhea. *Journal of Pediatric Gastroenterology and Nutrition.* 2003;36(1):112-115.

33. Crisinel PA, Verga ME, Kouame KSA, et al. Demonstration of the effectiveness of zinc in diarrhoea of children living in Switzerland. *Eur J Pediatr.* 2015;174(8):1061-1067.

34. Czerwionka-Szaflarska M, Murawska S, Swincow G. Evaluation of influence of oral treatment with probiotic and/or oral rehydration solution on course of acute diarrhoea in children. [Polish]. *Przeglad Gastroenterologiczny.* 2009;4(3):166-172.

35. Dagan R, Gorodischer R, Moses S. Dietary treatment of acute diarrhea: comparison between cow's milk and a soy formula without disaccharides. *Journal of tropical pediatrics.* 1984;30(4):221-224.

36. Dalgic N, Sancar M, Bayraktar B, Pullu M, Hasim O. Probiotic, zinc and lactose-free formula in children with rotavirus diarrhea: are they effective? *Pediatrics International.* 2011;53(5):677-682.

37. Das S, Gupta PK, Das RR. Efficacy and safety of saccharomyces boulardii in acute rotavirus diarrhea: Double blind randomized controlled trial from a developing country. *Journal of Tropical Pediatrics.* 2016;62(6):464-470.

38. Dewan V, Patwari AK, Jain M, Dewan N. A randomized controlled trial of vitamin A supplementation in acute diarrhea. *Indian Pediatr.* 1995;32(1):21-25.

39. Dinleyici EC, Dalgic N, Guven S, et al. The effect of a multispecies synbiotic mixture on the duration of diarrhea and length of hospital stay in children with acute diarrhea in Turkey: Single blinded randomized study. *Eur J Pediatr.* 2013;172(4):459-464.

40. Dinleyici EC, Vandenplas Y. Lactobacillus reuteri DSM 17938 effectively reduces the duration of acute diarrhoea in hospitalised children. *Acta paediatrica (Oslo, Norway : 1992).* 2014;103(7):e300-e305.

41. Dinleyici EC, Dalgic N, Guven S, et al. Lactobacillus reuteri DSM 17938 shortens acute infectious diarrhea in a pediatric outpatient setting. *J Pediatr (Rio J).* 2015;91(4):392-396.

42. Dinleyici EC, Kara A, Dalgic N, et al. Saccharomyces boulardii CNCM I-745 reduces the duration of diarrhoea, length of emergency care and hospital stay in children with acute diarrhoea. *Benef Microbes.* 2015;6(4):415-421.

43. Dugdale A, Lovell S, Gibbs V, Ball D. Refeeding after acute gastroenteritis: a controlled study. *Archives of Disease in Childhood.* 1982;57(1):76.

44. Dupont C, Foo JLK, Garnier P, Moore N, Mathiex-Fortunet H, Salazar-Lindo E. Oral Diosmectite Reduces Stool Output and Diarrhea Duration in Children With Acute Watery Diarrhea. *Clinical Gastroenterology and Hepatology.* 2009;7(4):456-462.

45. Dutta P, Datta A, Niyogi S, et al. Impact of zinc supplementation in malnourished children with acute water diarrhoea. *Journal of Tropical Pediatrics.* 2000;46(5):259-263.

46. Dutta P, Mitra U, Dutta S, Naik TN, Rajendran K, Chatterjee MK. Zinc, vitamin A, and micronutrient supplementation in children with diarrhea: A randomized controlled clinical trial of combination therapy versus monotherapy. *Journal of Pediatrics.* 2011;159(4):633-637.

47. Dutta P, Mitra U, Dutta S, Rajendran K, Saha TK, Chatterjee MK. Randomised controlled clinical trial of Lactobacillus sporogenes (Bacillus coagulans), used as probiotic in clinical practice, on acute watery diarrhoea in children. *Tropical Medicine and International Health.* 2011;16(5):555-561.

48. El-Soud NHAS, R. N.;Mosallam, D. S.;Barakat, N. A. M.;Sabry, M. A. Bifidobacterium lactis in treatment of children with acute diarrhea. A randomized double blind controlled trial. *Macedonian Journal of Medical Sciences.* 2015;3(3):403-407.

49. Eren M, Dinleyici EC, Vandenplas Y. Clinical efficacy comparison of Saccharomyces boulardii and yogurt fluid in acute non-bloody diarrhea in children: A randomized, controlled, open label study. *American Journal of Tropical Medicine and Hygiene.* 2010;82(3):488-491.

50. Fajolu IB, Emokpae A, Oduwole AO, Silva BO, Abidoye RO, Renner JK. Zinc supplementation in children with acute diarrhoea. *Nigerian quarterly journal of hospital medicine.* 2008;18(2):101-103.

51. Fayad IM, Hashem M, Hussein A, Abou Zikri M, Abu Zikri M, Santosham M. Comparison of soy-based formulas with lactose and with sucrose in the treatment of acute diarrhea in infants. *Archives of Pediatrics and Adolescent Medicine.* 1999;153(7):675-680.

52. Francavilla R, Lionetti E, Castellaneta S, et al. Randomised clinical trial: Lactobacillus reuteri DSM 17938 vs. placebo in children with acute diarrhoea--a double-blind study. *Aliment Pharmacol Ther.* 2012;36(4):363-369.

53. Freedman SBS, P. M.;Willan, A.;Johnson, D.;Gouin, S.;Schuh, S. Emergency Department Treatment of Children With Diarrhea Who Attend Day Care: A Randomized Multidose Trial of a Lactobacillus helveticus and Lactobacillus rhamnosus Combination Probiotic. *Clinical pediatrics.* 2015;54(12):1158-1166.

54. Gharial J, Laving A, Were F. Racecadotril for the treatment of severe acute watery diarrhoea in children admitted to a tertiary hospital in Kenya. *BMJ Open Gastroenterology.* 2017;4(1):e000124.

55. Gilbert B, Lienhardt A, Palomera S, Barberis L, Borreda D. The efficacy of smectite in acute infantile diarrhea, compared to a placebo and loperamide. [French]. *Annales de pediatrie.* 1991;38(9):633-636.

56. Grandy G, Medina M, Soria R, Teran CG, Araya M. Probiotics in the treatment of acute rotavirus diarrhoea. A randomized, double-blind, controlled trial using two different probiotic preparations in Bolivian children. *BMC Infectious Diseases.* 2010;10(253).

57. Grandy G, Jose Z. Effect of probiotic yogurt in the management of acute diarrhea in children. *Pediatr Res.* 2012;72 (1):109.

58. Groothuis JR, Berman S, Chapman J. Effect of carbohydrate ingested on outcome in infants with mild gastroenteritis. *Journal of Pediatrics.* 1986;108(6):903-906.

59. Gu B. Efficacy of golden bifid in treatment of viral diarrhea in infants. *Zhongguo Weishengtaxixue Zazhi / Chinese Journal of Microecology; 2011.* 2011;23(9):841.

60. Guandalini S, Pensabene L, Zikri MA, et al. Lactobacillus GG administered in oral rehydration solution to children with acute diarrhea: A multicenter European trial. *Journal of Pediatric Gastroenterology and Nutrition.* 2000;30(1):54-60.

61. Guarino A, Berni Canani R, Spagnuolo MI, Albano F, Di Benedetto L. Oral bacterial therapy reduces the duration of symptoms and of viral excretion in children with mild diarrhea. *Journal of Pediatric Gastroenterology and Nutrition.* 1997;25(5):516-519.

62. Guarino A, Bisceglia M, Castellucci G, et al. Smectite in the treatment of acute diarrhea: A nationwide randomized controlled study of the Italian society of pediatric gastroenterology and hepatology (SIGEP) in collaboration with primary care pediatricians. *Journal of Pediatric Gastroenterology and Nutrition.* 2001;32(1):71-75.

63. Hafeez A, Tariq P, Ali S, Kundi ZU, Khan A, Hassan M. The efficacy of Saccharomyces boulardii in the treatment of acute watery diarrhea in children: A multicentre randomized controlled trial. *Journal of the College of Physicians and Surgeons Pakistan.* 2002;12(7):432-434.

64. Haffejee IE. Cow's milk-based formula, human milk, and soya feeds in acute infantile diarrhea: A therapeutic trial. *Journal of Pediatric Gastroenterology and Nutrition.* 1990;10(2):193-198.

65. Hegar B, Waspada IMI, Gunardi H, Vandenplas Y. A Double Blind Randomized Trial Showing Probiotics to be Ineffective in Acute Diarrhea in Indonesian Children. *Indian J Pediatr.* 2015;82(5):410-414.

66. Henker J, Laass M, Blokhin BM, et al. The probiotic Escherichia coli strain Nissle 1917 (EcN) stops acute diarrhoea in infants and toddlers. *Eur J Pediatr.* 2007;166(4):311-318.

67. Henker J, Laass MW, Blokhin BM, et al. Probiotic escherichia coli nissle 1917 versus placebo for treating diarrhea of greater than 4 days duration in infants and toddlers. *Pediatr Infect Dis J.* 2008;27(6):494-499.

68. Henning B, Stewart K, Zaman K, Alam AN, Brown KH, Black RE. Lack of therapeutic efficacy of vitamin A for non-cholera, watery diarrhoea in Bangladeshi children. *European Journal of Clinical Nutrition.* 1992;46(6):437-443.

69. Heydarian F, Kianifar HR, Ahanchian H, Khakshure A, Seyedi J, Moshirian D. A comparison between traditional yogurt and probiotic yogurt in non-inflammatory acute gastroenteritis. *Saudi Medical Journal.* 2010;31(3):280-283.

70. Hoekstra JH, Szajewska H, Zikri MA, et al. Oral rehydration solution containing a mixture of non-digestible carbohydrates in the treatment of acute diarrhea: a multicenter randomized placebo controlled study on behalf of the ESPGHAN working group on intestinal infections. *Journal of pediatric gastroenterology and nutrition.* 2004;39(3):239-245.

71. Hoque ABMM, Larson CP, Khan AM, Saha UR. Initiation of zinc treatment for acute childhood diarrhoea and risk for vomiting or regurgitation: A randomized, double-blind, placebo-controlled trial. *Journal of Health, Population and Nutrition.* 2005;23(4):311-319.

72. Htwe K, Khin SY, Tin M, Vandenplas Y. Effect of Saccharomyces boulardii in the treatment of acute watery diarrhea in Myanmar children: A randomized controlled study. *American Journal of Tropical Medicine and Hygiene.* 2008;78(2):214-216.

73. Huang YF, Liu PY, Hsieh KS. Clinical effectiveness of three-combination probiotics therapy in pediatric patients with salmonella and rotavirus gastroenteritis: Randomised clinical trial. *International Journal of Infectious Diseases.* 2012;16:e199.

74. Huang YF, Liu PY, Chen YY, et al. Three-combination probiotics therapy in children with salmonella and rotavirus gastroenteritis. *J Clin Gastroenterol.* 2014;48(1):37-42.

75. Islek A, Sayar E, Yilmaz A, Baysan BO, Mutlu D, Artan R. The role of Bifidobacterium lactis B94 plus inulin in the treatment of acute infectious diarrhea in children. *Turkish Journal of Gastroenterology.* 2014;25(6):628-633.

76. Isolauri E, Vesikari T, Saha P, Viander M. Milk versus no milk in rapid refeeding after acute gastroenteritis. *Journal of pediatric gastroenterology and nutrition.* 1986;5(2):254-261.

77. Isolauri E, Juntunen M, Rautanen T, Sillanaukee P, Koivula T. A human Lactobacillus strain (Lactobacillus casei sp strain GG) promotes recovery from acute diarrhea in children. *Pediatrics.* 1991;88(1):90-97.

78. Isolauri E, Kaila M, Mykkanen H, Ling WH, Salminen S. Oral bacteriotherapy for viral gastroenteritis. *Digestive Diseases and Sciences.* 1994;39(12):2595-2600.

79. Jiang C, Xu C, Yang C. Therapeutic effects of zinc supplement as adjunctive therapy in infants and young children with rotavirus enteritis. *Zhongguo dang dai er ke za zhi [Chinese journal of contemporary pediatrics].* 2017;18(9):826-830. <http://onlinelibrary.wiley.com/o/cochrane/clcentral/articles/304/CN-01263304/frame.html>.

80. Kang G, Thuppal S, Srinivasan R, et al. Racecadotril in the Management of Rotavirus and Non-rotavirus Diarrhea in Under-five Children: two Randomized, Double-blind, Placebo-controlled Trials. *Indian Pediatr.* 2017;53(7):595-600. <http://onlinelibrary.wiley.com/o/cochrane/clcentral/articles/161/CN-01307161/frame.html>.

81. Kaplan MA, Prior MJ, McKonly KI, DuPont HL, Temple AR, Nelson EB. A multicenter randomized controlled trial of a liquid loperamide product versus placebo in the treatment of acute diarrhea in children. *Clinical Pediatrics.* 1999;38(10):579-591.

82. Karamyyar M, Gheibi S, Noroozi M, Valeshabad AK. Therapeutic effects of oral zinc supplementation on acute watery diarrhea with moderate dehydration: A double-blind randomized clinical trial. *Iranian Journal of Medical Sciences.* 2013;38(2):93-99.

83. Karrar ZA, Abdulla MA, Moody JB. Loperamide in acute diarrhoea in childhood: Results of a double blind, placebo controlled clinical trial. *Annals of Tropical Paediatrics.* 1987;7(2):122-127.

84. Kassem AS, Madkour AAS, Massoud BZ, Mehanna ZM. Loperamide in acute childhood diarrhoea: A double blind controlled trial. *Journal of Diarrhoeal Diseases Research.* 1983;1(1):10-16.

85. Khan A, Javed T, Chishti AL. Clinical efficacy of use of probiotic "Saccharomyces boulardii" In children with acute watery diarrhea. *Pakistan Paediatric Journal.* 2012;36(3):122-127.

86. Khanna V, Alam S, Malik A. Efficacy of tyndalized Lactobacillus acidophilus in acute diarrhea. *Indian J Pediatr.* 2005;72(11):935-938.

87. Kianifar HR, Farid R, Ahanchian H, Jabbari F, Moghiman T, Sistanian A. Probiotics in the treatment of acute diarrhea in young children. *Iranian Journal of Medical Sciences.* 2009;34(3):204-207.

88. Kowalska-Duplaga K, Strus M, Heczko P, Krobicka B, Kurowska-Baran D, Mrukowicz J. Lactobif, a marketed probiotic product containing Bifidobacterium ruminantium was not effective in the treatment of acute rotavirus diarrhoea in infants. *J Pediatr Gastroenterol Nutr.* 2002;34(4).

89. Kurugol Z, Koturoglu G. Effects of Saccharomyces boulardii in children with acute diarrhoea. *Acta Paediatrica, International Journal of Paediatrics.* 2005;94(1):44-47.

90. Leake RD, Schroeder KC, Benton DA, Oh W. Soy-Based Formula in the Treatment of Infantile: Diarrhea. *American Journal of Diseases of Children.* 1974;127(3):374-376.

91. Lee DK, Park JE, Kim MJ, Seo JG, Lee JH, Ha NJ. Probiotic bacteria, B.longum and L.acidophilus inhibit infection by rotavirus in vitro and decrease the duration of diarrhea in pediatric patients. *Clinics and Research in Hepatology and Gastroenterology.* 2015;39(2):237-244.

92. Lei V, Friis H, Michaelsen KF. Spontaneously fermented millet product as a natural probiotic treatment for diarrhoea in young children: An intervention study in Northern Ghana. *International Journal of Food Microbiology.* 2006;110(3):246-253.

93. Lexomboon U, Harikul S, Lortholary O. Control randomized study of rehydration/rehydration with dioctahedral smectite in ambulatory Thai infants with acute diarrhea. *The Southeast Asian journal of tropical medicine and public health.* 1994;25(1):157-162.

94. Lifshitz F, Neto UF, Olivo CAG, Cordano A, Friedman S. Refeeding of infants with acute diarrheal disease. *The Journal of pediatrics.* 1991;118(4):S99-S108.

95. López Hernández C, Estrada Pineda E, Rojas Jiménez MI, Salas Lucena M. Ensayo clínico terapéutico sobre Saccharomyces boulardii en niños con diarrea aguda

Saccharomyces boulardii clinical therapeutics trial in children affected by acute diarrhea. *Rev enfermedades infecc ped.* 1998;11(43):87-89.

96. Lozano JM, Cespedes JA. Lactose vs. lactose free regimen in children with acute diarrhoea: a randomized controlled trial. *Archivos latinoamericanos de nutricion.* 1994;44(1):6-11.

97. Madkour AA, Madina EMH, El-Azzouni OEZ, Amer MA, El-Walili TMK, Abbass T. Smectite in acute diarrhea in children: A double-blind placebo-controlled clinical trial. *Journal of Pediatric Gastroenterology and Nutrition.* 1993;17(2):176-181.

98. Majamaa H, Isolauri E, Saxelin M, Vesikari T. Lactic acid bacteria in the treatment of acute rotavirus gastroenteritis. *Journal of Pediatric Gastroenterology and Nutrition.* 1995;20(3):333-338.

99. Manyal AK, Phadke VD. Role of probiotic Lactobacillus sporogenes in the treatment of acute diarrhoea in children. *International Journal of Pharmaceutical Sciences and Research.* 2015;6(2):767-771.

100. Mao M, Yu T, Xiong Y, et al. Effect of a lactose-free milk formula supplemented with bifidobacteria and streptococci on the recovery from acute diarrhoea. *Asia Pacific journal of clinical nutrition.* 2008;17(1):30-34.

101. Maudgal D, Bradshaw J, Wansbrough-Jones M, Lambert H. Management of acute gastroenteritis in children. *British medical journal (Clinical research ed).* 1985;290(6477):1287.

102. Melendez Garcia J, Rodriguez J. Racecadotril en el tratamiento de la diarrea aguda en ninos. *Rev Facultad Med (Guatemala).* 2007;4:25-28.

103. Michael SSA, Abdelhaleem Ali AM, Ezzat DA, Tayel SA. Evaluation of racecadotril in treatment of acute diarrhea in children. *Asian Journal of Pharmaceutical and Clinical Research.* 2014;7(4):227-230.

104. Milocco C, Bolis A, Rizzo V, et al. [Evaluation of diosmectite in acute diarrhea in children]. *La Pediatria medica e chirurgica: Medical and surgical pediatrics.* 1998;21(3):129-133.

105. Misra S, Sabui TK, Pal NK. A Randomized Controlled Trial to Evaluate the Efficacy of Lactobacillus GG in Infantile Diarrhea. *Journal of Pediatrics.* 2009;155(1):129-132.

106. Moal VLL, Sarrazin-Davila LE, Servin AL. An experimental study and a randomized, double-blind, placebo-controlled clinical trial to evaluate the antisecretory activity of Lactobacillus acidophilus strain LB against nonrotavirus diarrhea. *Pediatrics.* 2007;120(4):e795-e803.

107. Movahedi Z, Sokrollahei MR, Tabaraie Y, Nodoushan KHA. Effect of zinc sulfate use on acute diarrhea in children (a clinical trial). *Qom University of Medical Sciences Journal; 2008.* 2008;2(2):Pe33-Pe36.

108. Mujawar QM, Naganoor R, Ali MD, Malagi N, Thobbi AN. Efficacy of Dioctahedral Smectite in Acute Watery Diarrhea in Indian Children: A randomized clinical trial. *Journal of Tropical Pediatrics.* 2012;58(1):63-67.

109. Naidoo B, Chunterpurshad I, Mahyoodeen A, Pather G. The use of a soy isolate based formula in the treatment of infantile diarrhoea. *Journal of International Medical Research.* 1981;9(3):232-235.

110. Narayanappa D. Randomized double blinded controlled trial to evaluate the efficacy and safety of Bifilac in patients with acute viral diarrhea. *Indian J Pediatr.* 2008;75(7):709-713.

111. Narkeviciute I, Rudzeviciene O, Leviniene G, Mociskiene K, Eidukevicius R. Management of Lithuanian children's acute diarrhoea with Gastrolit solution and dioctahedral smectite. *European Journal of Gastroenterology and Hepatology.* 2002;14(4):419-424.

112. Negi R, Dewan P, Shah D, Das S, Bhatnagar S, Gupta P. Oral zinc supplements are ineffective for treating acute dehydrating diarrhoea in 5-12-year-olds. *Acta Paediatrica, International Journal of Paediatrics.* 2015;104(8):e367-e371.

113. Nixon AF, Cunningham SJ, Cohen HW, Crain EF. The effect of Lactobacillus GG on acute diarrheal illness in the pediatric emergency department. *Pediatric Emergency Care.* 2012;28(10):1048-1051.

114. Noreen A, Jalal A, Qudsia, Jabeen R, Qamar S. Comparison of lactose free with lactose containing formula milk in the management of acute watery diarrhoea in infants. *Pakistan Journal of Medical and Health Sciences.* 2016;10(4):1337-1339.

115. Oandasan M, Gatcheco F, Kapahmgan S. Randomized, double blind placebo-controlled clinical trial on the effi-cacy and safety of Infloran berna capsules in the treatment of acute non-bloody diarrhea in infants. *Niepublikowane (Cytowane za 4).* 1999.

116. Owens JR, Broadhead R, Hendrickse RG, Jaswal OP, Gangal RN. Loperamide in the treatment of acute gastroenteritis in early childhood. Report of a two centre, double-blind, controlled clinical trial. *Annals of Tropical Paediatrics.* 1981;1(3):135-141.

117. Ozkan TB, Sahin E, Erdemir G, Budak F. Effect of Saccharomyces boulardii in children with acute gastroenteritis and its relationship to the immune response. *Journal of International Medical Research.* 2007;35(2):201-212.

118. Pant AR, Graham SM, Allen SJ, et al. Lactobacillus GG and acute diarrhoea in young children in the tropics. *Journal of Tropical Pediatrics.* 1996;42(3):162-165.

119. Pashapour N, Iou SG. Evaluation of yogurt effect on acute diarrhea in 6-24-month-old hospitalized infants. *Turkish Journal of Pediatrics.* 2006;48(2):115-118.

120. Passariello A, Terrin G, Cecere G, et al. Randomised clinical trial: Efficacy of a new synbiotic formulation containing Lactobacillus paracasei B21060 plus arabinogalactan and xilooligosaccharides in children with acute diarrhoea. *Alimentary Pharmacology and Therapeutics.* 2012;35(7):782-788.

121. Patel A, Dibley MJ, Mamtani M, Badhoniya N, Kulkarni H. Zinc and copper supplementation in acute diarrhea in children: A double-blind randomized controlled trial. *BMC Medicine.* 2009;7(22).

122. Patel H, Shah R, Gajjar B. Evaluation of the role of zinc supplementation in treatment of diarrhoea in paediatric patients: a randomized open-label study. *Drugs & Therapy Perspectives.* 2015;31(1):34-38.

123. Patro B, Szymanski H, Szajewska H. Oral zinc for the treatment of acute gastroenteritis in polish children: A randomized, double-blind, placebo-controlled trial. *Journal of Pediatrics.* 2010;157(6):984-988.e981.

124. Phavichitr N, Puwdee P, Tantibhaedhyangkul R. Cost-benefit analysis of the probiotic treatment of children hospitalized for acute diarrhea in Bangkok, Thailand. *The Southeast Asian journal of tropical medicine and public health.* 2013;44(6):1065-1071.

125. Pieacik-Lech M, Urbanska M, Szajewska H. Lactobacillus GG (LGG) and smectite versus LGG alone for acute gastroenteritis: A double-blind, randomized controlled trial. *Eur J Pediatr.* 2013;172(2):247-253.

126. Placzek M, Walker-Smith J. Comparison of two feeding regimens following acute gastroenteritis in infancy. *Journal of pediatric gastroenterology and nutrition.* 1984;3(2):245-248.

127. Pociecha W, Balcerska A. Influence of the mucoprotective drugs on the clinical course of children's rotaviral gastroenteritis. [Polish]. *Gastroenterologia Polska.* 1998;5(6):533-542.

128. Polat TB, Uysalol M, Cetinkaya F. Efficacy of zinc supplementation on the severity and duration of diarrhea in malnourished Turkish children. *Pediatrics International.* 2003;45(5):555-559.

129. Quak SH, Low PS, Quah TC, Teo J. Oral refeeding following acute gastro-enteritis: a clinical trial using four refeeding regimes. *Annals of Tropical Paediatrics.* 1989;9(3):152-155.

130. Rafeey M, Ostadrahimi A, Boniadi M, Ghorashi Z, Alizadeh MM, Hadafey V. Lactobacillus acidophilus yogurt and supplement in children with acute diarrhea: A clinical trial. *Research Journal of Medical Sciences.* 2008;2(1):13-18.

131. Ransome OJ, Roode H. Early introduction of milk feeds in acute infantile gastro-enteritis. A controlled study. *South African Medical Journal.* 1984;65(4):127-128.

132. Raza S, Graham SM, Allen SJ, Sultana S, Cuevas L, Hart CA. Lactobacillus GG promotes recovery from acute nonbloody diarrhea in Pakistan. *Pediatr Infect Dis J.* 1995;14(2):107-111.

133. Rehman A, Ahmad M, Chaudhry TA, Tehseen SA. The Efficacy of Diosmectite in Admitted Children Having Acute Watery Diarrhea With Dehydration. *Pakistan Paediatric Journal.* 2013;37(2):91-94.

134. Rerksuppaphol S, Rerksuppaphol L. Lactobacillus acidophilus and Bifidobacterium bifidum stored at ambient temperature are effective in the treatment of acute diarrhoea. *Annals of tropical paediatrics.* 2010;30(4):299-304.

135. Riaz M, Alam S, Malik A, Ali SM. Efficacy and safety of saccharomyces boulardii in acute childhood diarrhea: A double blind randomised controlled trial. *Indian J Pediatr.* 2012;79(4):478-482.

136. Ritchie BK, Brewster DR, Tran CD, Davidson GP, McNeil Y, Butler RN. Efficacy of Lactobacillus GG in aboriginal children with acute diarrhoeal disease: A randomised clinical trial. *Journal of Pediatric Gastroenterology and Nutrition.* 2010;50(6):619-624.

137. Rosenfeldt V, Michaelsen KF, Jakobsen M, et al. Effect of probiotic Lactobacillus strains in young children hospitalized with acute diarrhea. *Pediatr Infect Dis J.* 2002;21(5):411-416.

138. Rosenfeldt V, Michaelsen KF, Jakobsen M, et al. Effect of probiotic Lactobacillus strains on acute diarrhea in a cohort of nonhospitalized children attending day-care centers. *Pediatr Infect Dis J.* 2002;21(5):417-419.

139. Roy SK, Tomkins AM, Akramuzzaman SM, et al. Randomised controlled trial of zinc supplementation in malnourished Bangladeshi children with acute diarrhoea. *Archives of Disease in Childhood.* 1997;77(3):196-200.

140. Sachdev HP, Mittal NK, Mittal SK, Yadav HS. A controlled trial on utility of oral zinc supplementation in acute dehydrating diarrhea in infants. *Journal of pediatric gastroenterology and nutrition.* 1988;7(6):877-881.

141. Salazar-Lindo E, Santisteban-Ponce J, Chea-Woo E, Gutierrez M. Racecadotril in the treatment of acute watery diarrhea in children. *New England Journal of Medicine.* 2000;343(7):463-467.

142. Salazar-Lindo E, Miranda-Langschwager P, Campos-Sanchez M, Chea-Woo E, Sack RB. Lactobacillus casei strain GG in the treatment of infants with acute watery diarrhea: A randomized, double-blind, placebo controlled clinical trial [ISRCTN67363048]. *BMC Pediatrics.* 2004;4(18).

143. Saneian H, Yaghini O, Modaresi M, Razmkhah N. Lactose-Free Compared with Lactose-Containing Formula in Dietary Management of Acute Childhood Diarrhea. *Iranian journal of pediatrics.* 2012;22(1):82.

144. Santos M, Maranon R, Miguez C, Vazquez P, Sanchez C. Use of Racecadotril as Outpatient Treatment for Acute Gastroenteritis: A Prospective, Randomized, Parallel Study. *Journal of Pediatrics.* 2009;155(1):62-67.

145. Sarker SA, Sultana S, Fuchs GJ, et al. Lactobacillus paracasei strain ST11 has no effect on rotavirus but ameliorates the outcome of nonrotavirus diarrhea in children from Bangladesh. *Pediatrics.* 2005;116(2):e221-228.

146. Sazawal S, Black RE, Bhan MK, Bhandari N, Sinha A, Jalla S. Zinc supplementation in young children with acute diarrhea in India. *New England Journal of Medicine.* 1995;333(13):839-844.

147. Shan Z. STUDY ON SMECTA THERAPY FOR ACUTE DIARRHEA IN INFANT. *JOURNAL OF TIANJIN MEDICAL UNIVERSITY.* 1997:04.

148. Shamir R, Makhoul IR, Etzioni A, Shehadeh N. Evaluation of a diet containing probiotics and zinc for the treatment of mild diarrheal illness in children younger than one year of age. *Journal of the American College of Nutrition.* 2005;24(5):370-375.

149. Shornikova AV, Casas IA, Isolauri E, Mykkanen H, Vesikari T. Lactobacillus reuteri as a therapeutic agent in acute diarrhea in young children. *Journal of Pediatric Gastroenterology and Nutrition.* 1997;24(4):399-404.

150. Shornikova AV, Casas IA, Mykkanen H, Salo E, Vesikari T. Bacteriotherapy with Lactobacillus reuteri in rotavirus gastroenteritis. *Pediatr Infect Dis J.* 1997;16(12):1103-1107.

151. Shornikova AV, Isolauri E, Burkanova L, Lukovnikova S, Vesikari T. A trial in the Karelian Republic of oral rehydration and Lactobacillus GG for treatment of acute diarrhoea. *Acta Paediatrica, International Journal of Paediatrics.* 1997;86(5):460-465.

152. Simakachorn N, Pichaipat V, Rithipornpaisarn P, Kongkaew C, Tongpradit P, Varavithya W. Clinical evaluation of the addition of lyophilized, heat-killed Lactobacillus acidophilus LB to oral rehydration therapy in the treatment of acute diarrhea in children. *Journal of Pediatric Gastroenterology and Nutrition.* 2000;30(1):68-72.

153. Simakachorn N, Tongpenyai Y, Tongtan O, Varavithya W. Randomized, double-blind clinical trial of a lactose-free and a lactose-containing formula in dietary management of acute childhood diarrhea. *Journal of the Medical Association of Thailand.* 2004;87(6):641-649.

154. Sindhu KNC, Sowmyanarayanan TV, Paul A, et al. Immune response and intestinal permeability in children with acute gastroenteritis treated with lactobacillus rhamnosus GG: A randomized, double-blind, placebo-controlled trial. *Clinical Infectious Diseases.* 2014;58(8):1107-1115.

155. Strand TA, Chandyo RK, Bahl R, et al. Effectiveness and efficacy of zinc for the treatment of acute diarrhea in young children. *Pediatrics.* 2002;109(5):898-903.

156. Su HP, Qian SH, Zhang L. Effects of lactose-free milk in adjunctive treatment of infants with rotavirus enteritis and lactose intolerance: An analysis 30 cases. [Chinese]. *World Chinese Journal of Digestology.* 2014;22(31):4853-4857.

157. Sutton RE, Hamilton J. Tolerance of young children with severe gastroenteritis to dietary lactose: a controlled study. *Canadian Medical Association Journal.* 1968;99(20):980.

158. Szymanski H, Pejcz J, Jawien M, Chmielarczyk A, Strus M, Heczko PB. Treatment of acute infectious diarrhoea in infants and children with a mixture of three Lactobacillus rhamnosus strains - A randomized, double-blind, placebo-controlled trial. *Alimentary Pharmacology and Therapeutics.* 2006;23(2):247-253.

159. Teran CG, Teran-Escalera CN, Villarroel P. Nitazoxanide vs. probiotics for the treatment of acute rotavirus diarrhea in children: a randomized, single-blind, controlled trial in Bolivian children. *International Journal of Infectious Diseases.* 2009;13(4):518-523.

160. Torabi S, Abbaszadeh R, Ahmadiafshar A. The effect of oral zinc sulfate on the management of acute gastroenteritis in children. *Journal of Zanjan University of Medical Sciences and Health Services.* 2011;19(76).

161. Torrez J, Soria R, Grandy G. RACECADOTRIL, A GOOD ALTERNATIVE IN THE MANAGEMENT OF ACUTE WATERY DIARRHEA IN CHILDREN. Paper presented at: PEDIATRIC RESEARCH2013.

162. Touhami M, Boudraa G, Adlaoui M, et al. Should milk be diluted in benign acute diarrheas in eutrophic infants. [French]. *Archives Francaises de Pediatrie.* 1989;46(1):25-30.

163. Turck D, Berard H, Fretault N, Lecomte JM. Comparison of racecadotril and loperamide in children with acute diarrhoea. *Alimentary Pharmacology and Therapeutics, Supplement.* 1999;13(6):27-32.

164. Upadhyay A, Shah D, Teotia N, Agarwal A, Jaiswal V. Lactobacillus GG for treatment of acute childhood diarrhoea: an open labelled, randomized controlled trial. *The Indian journal of medical research.* 2014;139(3):379-385.

165. Urganci N, Polat T, Uysalol M, Cetinkaya F. Evaluation of the efficacy of Saccharomyces boulardii in children with acute diarrhoea. *Archives of Gastroenterohepatology.* 2001;20(3-4):81-83.

166. Vandenplas Y, De Hert SG. Randomised clinical trial: The synbiotic food supplement Probiotical vs. placebo for acute gastroenteritis in children. *Alimentary Pharmacology and Therapeutics.* 2011;34(8):862-867.

167. Villarruel G, Rubio DM, Lopez F, et al. Saccharomyces boulardii in acute childhood diarrhoea: A randomized, placebo-controlled study. *Acta Paediatrica, International Journal of Paediatrics.* 2007;96(4):538-541.

168. Vivatvakin B, Jongpipatvanich S, Harikul S, Eksaengri P, Lortholary O. Control study of oral rehydration solution (ORS)/ORS + dioctahedral smectite in hospitalized Thai infants with acute secretory diarrhea. *Southeast Asian J Trop Med Public Health.* 1992;23(3):414-419.

169. Vivatvakin B, Kowitdamrong E. Randomized control trial of live Lactobacillus acidophilus plus Bifidobacterium infantis in treatment of infantile acute watery diarrhea. *Journal of the Medical Association of Thailand = Chotmaihet thangphaet.* 2006;89 Suppl 3:S126-133.

170. Wall CR, Webster J, Quirk P, et al. The nutritional management of acute diarrhea in young infants: Effect of carbohydrate ingested. *Journal of Pediatric Gastroenterology and Nutrition.* 1994;19(2):170-174.

171. Widiasa AM, Soetjiningsih KP. Efficacy of dioctahedral smectite in infants with acute diarrhea: a double blind randomized controlled trial. *Paediatr Indones.* 2009;49(1):49.

172. Xu Y. Curative effect of Clostridium Butyricum Powder combined with Zinc Gluconate on children with acute diarrhea. *Zhongguo Weishengtaxixue Zazhi / Chinese Journal of Microecology.* 2014;26(5):546-548.

173. Yazar AS, Güven Ş, Dinleyici EÇ. Effects of zinc or synbiotic on the duration of diarrhea in children with acute infectious diarrhea. *Turk J Gastroenterol.* 2016;27:537-540.

174. Yurdakok K, Ozmert E, Yalcin SS, Laleli Y. Vitamin A supplementation in acute diarrhea. *Journal of Pediatric Gastroenterology and Nutrition.* 2000;31(3):234-237.
